# Supplementary material for: Covariation of Amino Acid Substitutions in the HIV-1 Envelope Glycoprotein gp120 and the Antisense Protein ASP Associated with Coreceptor Usage
Source: Viruses. 2025 Feb 26;17(3):323. doi: 10.3390/v17030323 (PMC11946160; doi:10.3390/v17030323)
Supplement: Supplementary file 1 [file viruses-17-00323-s001.zip › Supplementary File S1.pdf]

Supplementary File S1

Training dataset of 1838 amino acid sequences of the V3 loop domain with known tropism: 1701 sequences (1229 of genotype B and 472 of genotype C) are CCR5-tropic (R5) and 137 sequences (96 of genotype B and 41 of genotype C) are CXCR4-tropic (X4).

NCBI ac. number	Genotype	Tropism	V3 amino acid sequence	length (aa)
HQ644967	B	CCR5	CTRPNNNTRKSIHIGPGRAFYATGDIIGDIRKAHC	35
AJ418502	B	CCR5	CTRLNNNTRKSIHMGPGRAFYTTGEIIGDIRQAHC	35
JN687773	B	CCR5	CTRPYNNTRRSIPIGPGRAFYATGEVIGNIRKAYC	35
DQ061525	B	CCR5	CIRPNNNTRKSIHIGPGRAFYATGEIIGDIRQAHC	35
HQ377462	B	CCR5	CTRPNNNTRKSISMGPGRAFYATGGIIGNIRQAHC	35
AF541040	B	CCR5	CTRPNNNTRKSIHIGPGRAFYTTGEIIGDIRQAHC	35
DQ061827	B	CCR5	CTRPNNNTRKGIHMGPGKVFYATGQIIGDIRQAHC	35
EF600077	B	CCR5	CTRPNNNTRKSIHIGPGRAFYATGDIVGDIRQAHC	35
EU604591	B	CCR5	CTRPNNNTRKSINIGPGRAWYATGQIIGDIRQAYC	35
EF600098	B	CCR5	CTRPNNNTRKGIHIGPGRAFYATGDIIGDIRQAHC	35
JN687814	C	CCR5	CTRPGNNTRKSIRIGPGQTFYATGEIIGDIRQAHC	35
AY835439	B	CCR5	CTRPGNNTRKSIHIGPGRAFYATGDIIGDIRQAHC	35
EU293448	C	CCR5	CTRPGNNTRQSIRIGPGQAFYTNNIIGDIRQAHC	34
JX973238	C	CCR5	CTRPGNNTRKSIRIGPGQTFFATGDVIGDIREAHC	35
FJ977092	C	CCR5	CTRPNNNTRKSIRIGPGQTFYATGDIIGDIRQAHC	35
EF643670	B	CCR5	CTRPSNNTRKSITIGPGRAFYATGEIIGDIRKAHC	35
DQ002184	B	CCR5	CTRPNNNTRRSITIGPGRAFYATDIIGDIRQAHC	34
FJ653401	B	CCR5	CTRPNNNTRKSIHIAPGRAFYATGDIIGDIRQAHC	35
HQ377491	B	CCR5	CTRPGNNTRKSIPIGPGRAFYATGDIIGDIRKAHC	35
KC156221	C	CCR5	CTRVGNNTRKSVRIGPGQTFYATGDIIGDIREAHC	35
AF259038	B	CCR5	CTRPGNNTRKGIHIGPGRAFYTTGQIIGNIRQAHC	35
GU455514	B	CCR5	CTRPNNNTRKGIHIGPGRAFYTTGQIIGDIRQAHC	35
JN687721	C	CCR5	CTRPNNNTRKSVRIGPGQAFYATNGIVGDIRQAHC	35
HQ644912	B	CCR5	CMRPNNNTRKSIHIGPGRAFYTTGDIIGDIRQAHC	35
JF896843	B	CCR5	CTRPNNNTRKSIHIGPGRAFYATGQIIGNIRQAHC	35
KC113010	B	CCR5	CTRPNNNTRKIHIGPGKTFFATEVIGIRKAHC	32
JN001998	B	CCR5	CTRPNNNTRKSIHITPGRAFYATGEKIGDIRQAHC	35
KF770337	C	CCR5	CIRPGNNTRKSMRIGPGQTFYATGEIIGDIRQAHC	35
KC312501	B	CCR5	CTRPNNNTRKGIHIGPGRTFYVTGQIIGDIRQAHC	35
HM215411	C	CCR5	CTRPNNNTRRSIRIGPGQTFFAHGEIIGDIRQAHC	35
HQ708060	C	CCR5	CTRPNNNTRKSVRIGPGQTFYATGIIGNIRQAYC	34
KC156283	C	CCR5	CTRPNNNTRKSMRIGPGQTFYATGAIIGNIRQAYC	35
KC156440	C	CCR5	CIRPNNNTRKSVRIGPGQTFYATGEIIGDIREAYC	35
AY010823	B	CCR5	CTRPNNNTRKGIHIGPGSAIYATGDIIGDIRQAHC	35
KC156432	C	CCR5	CTRPNNNTRRSVRIGPGQTFYATGEIIGDIREAYC	35
DQ002206	B	CCR5	CTRPNNNTRKSIPIGPGRAFYATGEIIGDIRQAYC	35
JN002047	B	CCR5	CTRPNNNTRKSIPIGPGRAIYTTGGIIGDIRQAHC	35
EU744095	B	CCR5	CTRLNNNTRKSITFGPGRAFYATGDIIGNIRQAQC	35
EU578419	B	CCR5	CTRPNNNTRRGVTIGPGRVFYTGEVTGDIRQAHC	34
EU577028	B	CCR5	CTRPNNNTRKGITIGPGSVFYTGEIIGDIRQAHC	34
JQ779187	C	CCR5	CTRPGNNTRRSVRIGPGQTFYATGQIIGNIREAHC	35
AF153133	C	CCR5	CTRPNNNTRKSMRIGPGQTFYATGDIIGDIRQAHC	35
AF541049	B	CCR5	CTRPSNNTRKSIPMGPGKAFYATGDITGDIRKAHC	35
AF541010	B	CCR5	CTRPNNNTRKSIPMGPGKAFYATGDIIGDIRKAHC	35
JX972948	C	CCR5	CTRPNNNRRRSVRIGPGQSFYAYNDIIGNIREAYC	35
AF153144	C	CCR5	CTRPNNNTRKSIRIGPGQTFYANNAIIGNIRQAHC	35
DQ235642	C	CCR5	CTRPNNNTRKSIRIGPGQAFYATNDIIGDIRQAHC	35
DQ061825	B	CCR5	CTRPNNNTRKGIHMGSGAVFYATGQIIGDTRQAHR	35
HQ377375	B	CCR5	CTRPNNNTRKGIHLGPGGAFYTTGEIIGDIRKAHC	35
KC312591	B	CCR5	CVRPHNNTRKSIRIGPGSTFYATGEVIGDIRQAHC	35
HQ708066	C	CCR5	CIRPYNNTRKSMRIGPGQTFYATEEVIGDIRQAYC	35
AY010777	B	CCR5	CIRPNNNTRRSIHMGPGKAFYATGDIIGDIRQAYC	35
AF153152	C	CCR5	CTRPYNNTRKSMRIGPGQTFYATGDIIGDIRQAHC	35
EU744114	B	CCR5	CTRPNNNTRKSIHIGPGRAFYATGEIIGNIRQAHC	35
JF896859	B	CCR5	CTRPNNNTRRSIGIGPGRAFYATGIIGDIRQAHC	34
HM179798	C	CCR5	CIRPNNNTRTSIRIGPGQAFFATNGIIGNIRQAYC	35
AY253308	C	CCR5	CTRPGNNKRRSVRIGPGQTFYATGEIIGNIREAHC	35
HM179719	C	CCR5	CTRPNNNTRKSMRIGPGQAFYATGEIIGNIREAHC	35
FJ653102	B	CCR5	CTRPNNNTRKSINIGPGRAFYAATDIIGDIRQAHC	35
DQ061505	B	CCR5	CTRPSNNTRKSITIGPGRAFYTTGEILGEIRQAHC	35
HQ644953	B	CCR5	CTRPNNNTRKSIHMGPGRAFYATGEIIGDIRQAHC	35
HQ644892	B	CCR5	CTRPNNNTRKGIHIGPGRAFYTTGEITGDIRKAHC	35

FJ375997	C	CCR5	TRPNNNTRKSVRIGPGQTFYATGDIIGDIRQAHC	34
DQ235622	C	CCR5	CARPNNNTRKSIRIGPGQAFYATGEIIGNIRQAHC	35
EU744023	B	CCR5	CTRPSNNTRKSIHIGPGRAFYATGEIIGNIRQAHC	35
EU604569	B	CCR5	CTRPSNNTRKSINMGPGRAFYTTGEIIGDIRQAHC	35
HQ644821	B	CCR5	CTRPNNNTRKSIHIGPGRAFYATGEIIGDIRQAHC	35
DQ869033	B	CCR5	CTRPNNNTRKGVHIGPGRAFYATGEIIGDIRKAHC	35
U08717	B	CCR5	CTRPNNNTRKSIPLGPGQAWYTTGQIIGDIRQAHC	35
DQ002106	B	CCR5	CTRPNNNTRKSITIGPGRAFYATGDIIGDIRQAHC	35
AF541016	B	CCR5	CTRPNNNTRKSIPIGPGRAFYTTGEIIGDIRQAHC	35
EU744048	B	CCR5	CTRPSNNTRKGIHIGPGRALYATGEIIGDIRQAHC	35
AF384315	B	CCR5	CTKPYKKKKSRRIHIGPGRTFHTTGSIGDIRRAHC	35
HQ377411	B	CCR5	CTRPNNNTRRSIHIGPGSAFYATGDIIGDIRQAHC	35
EU744165	B	CCR5	CTRPNNNTRRSIHMGPGRALYTTGAIIGNIRQAHC	35
AF384271	B	CCR5	CTRPGNNTRRSIRIGPGSAFYATGDIIGDIRKAHC	35
JF508028	B	CCR5	CTRPNNNTRKSINIGPGRAFYTTGEIIGNIRQAHC	35
KF770409	C	CCR5	CTRPGNNTRKSMRIGPGQTFYATGDIIGDIRKAHC	35
DQ061444	B	CCR5	CTRPSNNTRKSIHIGPGRAFYTTGEIIGDIRQAHC	35
HQ708056	C	CCR5	CIRPNNNTRKSVRIGPGQTFYATGDIIGDIRRAYC	35
DQ516156	B	CCR5	CTRPNNNTRKDIHIGPGRAFYATGDIIGDIRQAHC	35
AF199039	B	CCR5	CIRPNNNTRQGIHIGPGKALYTTIIGNIRQAHC	33
EU272303	B	CCR5	CTRPNNNTRKSIHLGPGRAWYATGEIIGNIRQAHC	35
DQ235621	C	CCR5	CVRPNNNTRKSIRIGPGQTFYATGDIIGNIRQAHC	35
JX508865	C	CCR5	CTRPNNNTRKSFRIGPGQTFYATGDIIGDIRQAHC	35
DQ002248	B	CCR5	CTRPNNNTRKSIHIAPGRAFYATGEIIGDIRQAHC	35
AM156917	B	CCR5	CTRPSNNTRKGIHIGPGRAFYATGEIIGDIRQAHC	35
FJ653141	B	CCR5	CTRFNNNTRKSIHIGPGRAFYATGEIIGNIRQASC	35
JX140657	B	CCR5	CIRPGNNTRKSITMGPGRAFYATGEIIGNIRQAHC	35
DQ002070	B	CCR5	CIRPNNNTRKSIHIGPGRAFYTTGDIIGDIRQAHC	35
DQ002035	B	CCR5	CTRPHNNTRKSIPMGPGRAFYTAGQIIGDIRQAHC	35
AJ418494	B	CCR5	CTRPNNNTRKSISFGPGSAMYATGAIIGDIRQAHC	35
DQ002144	B	CCR5	CTRPNNNTRRGIHIGPGRAFYTTGEIIGDIRQAYC	35
EF600078	B	CCR5	CTRPNNNTRKSIHIGPGRALYATGDIIGDIRQAHC	35
KC312489	B	CCR5	CTRPNNNTRKGIHIGPGRTFYATGEIIGDIRQAHC	35
AF540999	B	CCR5	CTRPNNNTRKSIPIGPGRALYATGEIIGQIRRAYC	35
EU272203	B	CCR5	CTRPNNNTRKSIHLGQGRAWYATGEIIGDIRQAHC	35
KC312390	B	CCR5	CTRPNNNTRKSIHIGPGSAFYTTGEIIGDIRQAHC	35
EF657940	B	CCR5	CIRPNNNTRKSIHMGPGGAFYATGGIIGNIRQAHC	35
JQ779286	C	CCR5	CIRPNNNTRKSVRIGPGQTFYATGDIIGDIREAYC	35
JQ779910	C	CCR5	CTRPGNNTRKSVRIGPGQTYFSTGEIIGNIRQAHC	35
HQ644825	B	CCR5	CTRPNNNTRRSIHIGPGKAFFATGDIIGDIRQAHC	35
AF541011	B	CCR5	CTRPNNNTGKSIPIGPGRAFYATGEIIGDIRQAHC	35
AF541008	B	CCR5	CTRPSNNTRKSIPMGPGKAFYATGDIIGDIRKAHC	35
KC312576	B	CCR5	CVRPHNNTRKGIHIGPGSTFYATGEVIGDIRQAHC	35
HM179933	C	CCR5	CTRPGNNTRKSMWIGPGQAFYATGDIIGDIRQAYC	35
FJ653234	B	CCR5	CTRPSNNTSGSIHIGPGRAFDATKTITGDIRQAHC	35
JQ779139	C	CCR5	CTRPGNNTRKSVRIGPGQTFYATGEIIGDIRKAHC	35
DQ002082	B	CCR5	CTRPNNNTRKSIHIGPGRAFYTTGSIIGDIRQAHC	35
AJ810480	B	CCR5	CTRPSNNTRKSVHIGPGRALYTTDIIGDIRKAYC	34
JX972470	C	CCR5	CTRPNNNTRKSVRIGPGQTFYATGEIIGDIRQAYC	35
EU744039	B	CCR5	CTRPSNNTRKGIHIGPGRAFYTTGEIIGDIRQAHC	35
AF022263	B	CCR5	CTRPNNNTRRSISIGPGRAFYTTGEIIGNIRQAHC	35
EU293445	C	CCR5	CIRTGNNTRKSVRIGPGQTFYATDGIIGDIRKAYC	35
DQ061432	B	CCR5	CTRPNNNTRKSITIGPGRAFYTTGEIIGDIRQAHC	35
EF579980	B	CCR5	CERPGNNTSKGIHIGPGRAFYATENIIGDIRKAHC	35
EU272329	B	CCR5	CTRPNNNTRKSIHLGQGRAWYTTGQIIGDIRQAHC	35
KC156238	C	CCR5	CIRPGNNTRRSMRIGPGQTFYATGDIIGDIRKAHC	35
EU576584	B	CCR5	CTRPNNNTRKGIHIGPGKAFYTTGEIIGNIRQAHC	35
EU744156	B	CCR5	CTRPNNNTRRSIHIGPGRAFYATGDIIGDIRQAHC	35
HQ644881	B	CCR5	CTRPSNNTRKSINIGPGRAFYATGDIIGDIRKAYC	35
HM239634	B	CCR5	CIRPNNNTRKSIHVGPGSALYTTKIIGNIRQAHC	34
HM239547	B	CCR5	CTRPNNNTRKSIPMGPGQALYATGEIIGDIRQAHC	35
EU744150	B	CCR5	CTRPNNNTRKSIHIGPGRAFYTTGGIIGDIRQAHC	35
HQ644861	B	CCR5	CTRPSNNTRKSIHMGPGRAFYVTGDVIGDIRQAHC	35
AF258965	B	CCR5	CTRPNNNTRKSIHLGPGRAFYTTGGIVGNIRQAHC	35
HM368250	B	CCR5	CIRPHNNTRKSIHIGPGRTFYATGDIIGDIRKAHC	35
AF541112	B	CCR5	CVRPNNNTRRGIHIGLGRRFYTTEIVGDIRKAYC	34
AF543913	C	CCR5	CTRPSNNTRKSVRIGPGQTFFATGDIIGDIRQAHC	35
HQ644883	B	CCR5	CTRPNNNTRKSIHIGPGGAFYATGEIIGDIRQAHC	35
JN687752	B	CCR5	CVRPNNNTRTSIHMGPGKAFYAAGEVIGDIRRAYC	35
U08771	B	CCR5	CTRPNNNTRKSIHMGWGRAFYATGEIIGNIRQAHC	35

FJ653124	B	CCR5	CTRFYNNTRKSIHIGPGRAFYATGEIIGNIRQASC	35
AF491742	B	CCR5	CTRPNNNTRKSIPMGPGKAFYATGDIIGDIRQAHC	35
AF384280	B	CCR5	CIRPNNNTRTSIPMGPGRAWYAMGDIIGDIRQAHC	35
KF384808	B	CCR5	CTRPGNNTRKGITIGPGRAFYATGKIIGDIRQAHC	35
DQ061418	B	CCR5	CTRPNNNTRKSINIGPGRAFYTTGEIIGDIRQAHC	35
JN687784	C	CCR5	CTRPGNNTRRSVRIGPGQTFYATGDIIGNIRQAHC	35
JX972406	B	CCR5	CVRPGNNTRKSITIGPGRAFYATGEIIGDIRKAHC	35
JX972238	B	CCR5	CTRPNNNTRKSIHLGPGSAIYATGQIIGDIRQAHC	35
U08711	B	CCR5	CTRPNNNTRKSITIGPGRAFYATGEIIGDIRQAHC	35
KF770437	C	CCR5	CIRPGNNTSRSIRIGPGQAFYATGRIVGDIRQAHC	35
HM246239	B	CCR5	CTRPSNNTRKSISIGPGRAFYATGDIIGDIRQAHC	35
JF508012	B	CCR5	CTRPNTNTRKSINIGPGRAFYTTGEIIGNIRQAHC	35
AY010852	B	CCR5	CTRPSNNTRKSIYIGPGRAFYATGSIIGDIRQAHC	35
EU744106	B	CCR5	CTRPNNNTRKSINIGPGRALYTTGEIIGNIRQAHC	35
DQ516243	B	CCR5	CTRPNNNTRKGIHIGPGRAFYTTGEIIGDIRQAYC	35
JF896862	B	CCR5	CTRPGNNTRKGIHMGPGKAFFTTETVIGNVRQAHC	35
EF579987	B	CCR5	CERPGNNTSKGIHIGPGRAFYATENIIGDIRKARC	35
AF541098	B	CCR5	CIRPNNNTKRSIHLGLGRRFYTTEIVGDIRKAYC	34
KF770305	C	CCR5	CTRPDNNTRRSVRIGPGQVFYTNDIIGDIRRAHC	34
DQ061835	B	CCR5	CTRPNNNTRKGIHMGPGKVFYATGQIIGDIRQAYC	35
FJ653296	C	CCR5	CTRPNNNTRKSIRIGPGQTFFATEIVGNIRQAHC	34
AF384307	B	CCR5	CTRPNNNTRKSIHIGPGRAFYATGDIIGDIRQAHC	35
U08716	B	CCR5	CTRPNNNTRKSIHLGPGRAWYTTGQIIGDIRQAHC	35
JX973081	C	CCR5	CIRPGNNTRKSVRIGPGQAFYATGDIIGDIRQAHC	35
DQ235628	C	CCR5	CTRPNNNTRKSIRIGPGQTFYATNEIIGDIRQAHC	35
HQ708084	C	CCR5	CTRPNTNKRTSYTIGPGRAFFATGDIIGDIRRADC	35
JF507865	B	CCR5	CTRPGNNTRRSINIGPGRAFYTTGDIIGNIRQAHC	35
HM246216	B	CCR5	CTRPNNNTRKSIHIAPGGAFYATGDIIGDIRQAHC	35
AJ418500	B	CCR5	CTRLNNNTRKSIHMGPGRAFFATGEIIGDIRQAHC	35
EU272197	B	CCR5	CTRPNNNTRKSINLGPGQAWYTTGQIIGDIRQAHC	35
JN687771	B	CCR5	CTRPNNNTRKSINIGPGRAWYATGEIIGNIRQAHC	35
HQ708028	C	CCR5	CTRPDNNTRKSVRIGPGQTFYTTGDIIGNIRQAYC	35
HQ708094	C	CCR5	CTRPNNNIRKSYTIGPGRAFFATGDIIGDIRQADC	35
DQ061797	B	CCR5	CIRPNNNTRKGIHIGPGRAFYATGEIIGNIRQAHC	35
KF770354	C	CCR5	CTRPNNNTSQSIRIGPGQTFYAMGRIIGDIRQAHC	35
DQ061529	B	CCR5	CTRPNNNTRRSIHIAPGSTFFATGDIIGDIRQAHC	35
JX508864	C	CCR5	CTRPNNNTRQSFRIGPGQTFYATGDIIGDIRQAHC	35
DQ061693	B	CCR5	CIRPNNNTRKGVHLGPGGALYATGAIIGDIRQAYC	35
HQ708047	C	CCR5	CTRPNNNTRKSIRIGPGQTFYATGAVTGDIRKAYC	35
EF688444	B	CCR5	CTRPNNHTRKRMTLGPGRVYYTTGEIVGDIRQAHC	35
FJ653195	B	CCR5	CTRPNNNTRKGIHIGPGRAFYATEKITGDIRQAHC	35
KF770374	C	CCR5	CTRPGNNTSRSIRIGPGQTFYATGRITGNIRQAHC	35
HM179822	C	CCR5	CTRPGNNTRQSMRIGPGQTFYATGDIIGDIRPAHC	35
KF770306	C	CCR5	CTRPDNNTRRSVRIGPGQVFYTNDIIGDIRQAHC	34
EF600079	B	CCR5	CTRPNDNTRKSIHIGPGRALYATGDIIGDIRQAHC	35
DQ002072	B	CCR5	CIRPNNNTRKSIHIGPGRVFYTTGDIIGDIRQTHC	35
HQ644954	B	CCR5	CTRPNNNTRRSINIGPGRAFYATGEIIGDIRQAHC	35
JF508059	B	CCR5	CTRPNNNTRKSIHIGPGRAFYATGEIIGNIRQASC	35
AF180900	B	CCR5	CTRPNNNTRRSIHIGPGRAFYTTGQIIGDIRQAYC	35
DQ002105	B	CCR5	CTRLNNNTRRSINIGPGRAWYTTGEVIGDIRKANC	35
AJ418531	B	CCR5	CTRPHNNTRKSIHIGPGRAFYATGEIIGDIRQAHC	35
HQ645007	B	CCR5	CTRPSNNTRKSINFGPGRAIYTTGQIIGDIRQAHC	35
HM239583	B	CCR5	CTRPNNNTRKSINIGPGKALYATGDIIGDIRQAHC	35
EF657913	B	CCR5	CMRPNNNTRKSIHMGPGRAFYATGEIIGNIRQAHC	35
AF021510	B	CCR5	CTRPNNNTRKSIHIGPGRAFYTTGQIIGDIRQAYC	35
JF507860	B	CCR5	CTRPNNNTRKSINIGPGRAFYTTGQIIGDIRQAHC	35
JF508121	B	CCR5	CTRPNNNTRKSIHIGPGRTFYATGEIIGDIRQAHC	35
HQ644851	B	CCR5	CTRPNNNTRRSITIGPGRAFYTTDIIGDIRQAYC	34
DQ235636	C	CCR5	CTRPGNNTRKSVRIGPGQTFYATGEIIGDIRRAHC	35
DQ002324	B	CCR5	CTRPNNNTRRSIHMGPGRAFYTTGEIVGDIRQAYC	35
HM179926	C	CCR5	CTRPNNNIRKSIRIGPGQTFYATGDIIGDIRQAYC	35
JF896850	B	CCR5	CTRPNNNTRKSIHMGPGKAFYTAGDIIGDIRKAHC	35
AF391249	C	CCR5	CTRPNNNTRRSMRIRPGQTFYATGEIIGDIRQAYC	35
DQ516119	B	CCR5	CTRPNNNTIKSIHIGPGRAFYTTGQIIGNIRQAHC	35
DQ002304	B	CCR5	CTRPNNNTRKGIHIGPGRAFYATGDIIGDIRKAHC	35
AF540997	B	CCR5	CTRPNNNTRKSIPIGPGRAFYATGDIIGDIRQAHC	35
AY010871	B	CCR5	CTRPNNNTRTSIPMGPGRAMYATGDIIGNIRQAYC	35
DQ061462	B	CCR5	CTRPNNNTRKGIHIGPGRASYTTGEIIGDIRKAHC	35
DQ061538	B	CCR5	CTRPNNNTRRGIHIAPGSAFYATGEIIGDIRQAHC	35
JF896815	B	CCR5	CTRNNNRRGHVGPGGALFTNHIIGDIRQAHC	31

AY669739	C	CCR5	CTRPNNNTRRSIRIGPGQVFYANNDIIGDIRQAHC	35
KF770352	C	CCR5	CTRPGNNTRRSVRIGPGQAFYATGDIIGDIRAAHC	35
AF153185	C	CCR5	CIRPGNNTRKSVRIGPGQAFFATGDIIGDPRQAHC	35
EU272291	B	CCR5	CTRPSNNTRKSINLGPGTAWYTTGQIIGDIRQAHC	35
AY887888	C	CCR5	CTRPGNNTRKSMRIGPGQTFYATGAIIGDIRQAHC	35
HQ644811	B	CCR5	CTRPNNNTRRSINIGPGRAIFTTGEIIGDIRQAHC	35
JF508074	B	CCR5	CTRPNNNTRKSIHIGPGKAFYTTGEIIGDIRQAHC	35
HM239611	B	CCR5	CTRPNNNTRKSIHIGPGRAFYATGGIIGNIRHAYC	35
EF579995	B	CCR5	CERPGNNTSKGIHIGPGRAFYATENIVGDIRKAHC	35
JF507729	B	CCR5	CTRPNNNTRKGIHIGPGRTFFATGEIIGDIRRAHC	35
JQ779248	C	CCR5	CIRPGNNTRKSIRIGPGQVFFAATDIIGNIREAHC	35
DQ002306	B	CCR5	CTRPNNNTRKSINIGPGRAWYATGDIIGDIRKAHC	35
HQ678254	B	CCR5	CTRPNNNTRKGIHMGPGGAFYTRGDIIGDIRKAHC	35
FJ653236	B	CCR5	CTRPSNNTSQSIHIGPGRAFDATKTITGDIRQAHC	35
JX972841	C	CCR5	CTRPNNNTRTSIRIGPGQTFYATGDIIGEIRQAYC	35
GQ372988	B	CCR5	CTRPNNNTRKSIHVGWGRALYTTGQIIGDIRKAHC	35
EU578319	B	CCR5	CTRPNNNTRKSIHIGPGRAFYTGEIIGDIRQAHC	34
AY010797	B	CCR5	CIRPNNNTRKSIHMGPGKAFYATGDIIGDIRQAYC	35
DQ422948	C	CCR5	CTRPGNNTRKSVRIGPGQTFYATGAIIGDIRKAHC	35
HM239629	B	CCR5	CTRPNNNTRKGIPVGPGKAIYATGEIIGNIRQAHC	35
AY842838	B	CCR5	CIRPNNNTRKSINIGPGRAMYATEQIIGDIRQAHC	35
HM179732	C	CCR5	CARPNNNTRKSMRIGPGQAFYATGEIIGNIREAHC	35
HQ644828	B	CCR5	CTRPNNNTRRSIHIGPGRAFFATGDIIGDIRQAHC	35
HQ644920	B	CCR5	CMRPQNNTRKSIPIGPGRAFYTTGDIIGDIRQAHC	35
EF600086	B	CCR5	CIRPNNNTRQGIHIGPGKALYTTKIIGNIRQAHC	34
KC473834	B	CCR5	CTRPNNNTRKSINIGPGRAFYTTGDIIGDIRQAHC	35
KF770320	C	CCR5	CTRPNNNTRKSIRIGPGQTFFATDDIIGDIRKAYC	35
DQ235640	C	CCR5	CTRPNNNTRKSIRIGPGQTFYATNDIIGDIRQAHC	35
HM246201	B	CCR5	CTRPNNNTRKSINIGPGRAFYVAGEIIGDIRQAHC	35
JF507887	B	CCR5	CTRPNNNTKKSVNIGPGRAIYTTDIIGDIRKAYC	34
EU575490	B	CCR5	CTRPNNNTRRSIHIGPGRAFYTTGEVIGDIKQAHC	35
KC156324	C	CCR5	CTRPNTNIRKSMRIGPGQTFYATGDIIGDIRQAHC	35
JN002017	B	CCR5	CTRPNNNTRKGIHIGPGRAFYATGDIVGDIRQAHC	35
KC312494	B	CCR5	CTRPNNNTRKGIHIGPGRTFYATGEVIGNIRQAHC	35
HM179941	C	CCR5	CTRPGNNTRKSMWIGPGQVFYATGDIIGDIRQAYC	35
JX140664	C	CCR5	CTRPNNNTRKSIRIGPGQAFYATGDIIGNIREAHC	35
DQ061734	B	CCR5	CTRPNNNTRRSINLGPGRAYYATGDIIGDIRQAHC	35
KC156117	C	CCR5	CTRPSNNTRKSVRIGPGQTFYATGRIIGDIREAHC	35
EU272299	B	CCR5	SRPNNNTRKSIHLGLGRAWYATGEIIGNIRQAHC	34
DQ002334	B	CCR5	CTRPSNNTRKSIHIAPGRAFYATGDIIGNIRQAYC	35
DQ061412	B	CCR5	CTRPNNNTRKSITIGPGRAFYTAGEIIGDIRQAHC	35
AY010854	B	CCR5	CTRPSNNTRKSIHIGPGRAFYTTGAIIGDIRQAHC	35
EU293447	C	CCR5	CIRPNNNTRESIRIGPGQAFYATGGIIGDIRQAHC	35
JN002023	B	CCR5	CTRPNNNTRKSIHIGPGRAFYATGDIVGDIREAHC	35
HQ678294	B	CCR5	CTRPSNNTRRSIHMGPGKAYYTTDIIGDIRQAHC	34
KF384814	B	CCR5	CVRPNNNTRRSIHIGPGSAFYTTDIIGDIRQAHC	34
DQ061768	B	CCR5	CTRPSNNTRKGIHIGPGRAFYATGEIIGNIRQAHC	35
KC156321	C	CCR5	CNRPHNNTRKSMRIGPGQAFYATGDVTGDIRQAHC	35
AF153135	C	CCR5	CTRPNNNTRKSMRIGPGQTFYATGEIIGDIRQAHC	35
AY529676	C	CCR5	CTRPNNNTRQSVRIGPGQVFYATNDIIGDIRQAYC	35
DQ061537	B	CCR5	CTRPNNNTRRSIHIAPGSAFYATGEIIGDIRQAHC	35
AF541025	B	CCR5	CTRPNNNTRKSIHIGPGRAFYTTGEIIGNIRQAHC	35
DQ061583	B	CCR5	CMRPNNNTRKSIPIGPGRAFYTTGEIIGDIRQAHC	35
DQ388514	C	CCR5	CTRPNNNTRKSIRIGPGQTFYATGEIIGKIREAHC	35
AF153157	C	CCR5	CERPGNNTRKSVRIGPGQTFYATGEIIGNIRQAHC	35
HQ644853	B	CCR5	CTRPGNNTRKSINIGPGRALYTTGDIIGDIRQAHC	35
JF896836	B	CCR5	CTRPNNTKSIHIGPGRAFYATGEIIGDIQAHC	32
HQ377436	B	CCR5	CTRPNNNTRKSIHIGPGKAFYATGDIIGDIRQAHC	35
HQ377417	B	CCR5	CTRPNNNTRKSIHIGPGSAFYATGEIIGDIRQAHC	35
FJ375979	C	CCR5	ARPNNNTRKSVRIGPGQTFATGEIIDIRQAHC	32
KF770294	C	CCR5	CIRPNNNTRKSVRIGPGQTFYATGEVIGDIRKAHC	35
HQ377483	B	CCR5	CTRPGNNTRKSIPIGPGRAFYATGDIIGDVRKAHC	35
HQ644873	B	CCR5	CTRPNNNTRKGIHIGPGRAFYATGEIIGDIRQAHC	35
AY842814	B	CCR5	CTRPNNNTRKSIPIGPGRAMYATGDIIGDIRQAHC	35
EU744061	B	CCR5	CTRPNNNTRKSIHVGPGKTLYATGDIIGDIRQAHC	35
DQ869029	B	CCR5	CTRPNNNTRKSIHMGPGKAFYATGEIIGDIRKAYC	35
HM368238	B	CCR5	CTRPNNNTRRGIHIGPGGAFYSTGDIIGDIRQAHC	35
DQ061501	B	CCR5	CTRPSNNTRKSIHIGPGRAVYTTGEIIGRIRQAHC	35
DQ177208	B	CCR5	CTRLNNNTRKSIPMGPGRAFYATGDIIGDIRKAHC	35
EU744004	B	CCR5	CTRPNNNTRKSIPIGPGRAFYATGDIIGDIRKAHC	35

EU744147	B	CCR5	CTRPNNNTRKGIHIGPGRAFYTTGEIIGDIRQAHC	35
DQ002187	B	CCR5	CTRPGNNTRKSIHIGPGRAFYATDIIGDIRQAYC	34
AY010805	B	CCR5	CTRPNNNTRKGIHLGPGSAFYATGDIIGDIRQAHC	35
AF082837	B	CCR5	CTRPNNNTRKSIHIGPGRAFYATGDNIGDIRQAHC	35
EU744084	B	CCR5	CTRLNNNTRKSITLGPGRAFYATGDIIGNIRQAQC	35
HM179838	C	CCR5	CTRPGNNTRQSMRIGPGQTFYATGDIIGDIRQAHC	35
HM246191	B	CCR5	CTRPSNNTRKSIPIGPGRAFYATGDIIGDIRQAHC	35
AY510062	C	CCR5	CTRPGNNTRKSVRIGPGQTFYATGAIIGDIRQAHC	35
AF153158	C	CCR5	CTRPNNNTRKGVRIGPGQTFYATGDIIGDIRQAHC	35
AY570011	B	CCR5	CTRPNNNTRKSINLGPGRALYTTGEITGDIRQAHC	35
DQ002323	B	CCR5	CTRPNNNTRRSIHMGPGRAFYTTGEIIGDIRQAYC	35
JF508007	B	CCR5	CTRPNNNTRKSINIGPGRAFYTTGTIIGDIRQAHC	35
KC156401	C	CCR5	CIRPGNNTRKSIRIGPGQTDFSTGEIIGNIRQAHC	35
AF384268	B	CCR5	CTRPDNNTRKSINIGPGRAFFATGDIIGDIRQAHC	35
DQ061772	B	CCR5	CTRPDNNTRKGIHIGPGRAFYATGEIIGNIRQAHC	35
DQ382371	C	CCR5	CARPGNNTRRSVRIGPGQAFYATGEIIGDIRKAHC	35
HM239626	B	CCR5	CTRPNNNTRRGIHIGPGRTLFATDIIGDIRQAHC	34
KF770366	C	CCR5	CIRPGNNTRKSVRIGPGQTFYATGDIIGDIRQAHC	35
AY010851	B	CCR5	CTRPNNNTRKSIHLGPGSAFYATGDIIGDIRQAHC	35
AF384246	B	CCR5	CTRPNNNTRKSIHIAPGRTYYATGDIIGDIRQAHC	35
AF153155	C	CCR5	CTRPGNNTRKSIRIGPGQAFYATGDIIGDIRQAHC	35
HQ644905	B	CCR5	CTRPTNNTRKGIHIGPGRAFYTTGEIIGDIRKAHC	35
DQ002233	B	CCR5	CTRPSNNTRKSIPIGPGRAFYATGDIIGDIRKAHC	35
JF896847	B	CCR5	CTRPNNNTRKSIPLGPGSAFYATDRIGDIRQAYC	34
HQ644832	B	CCR5	CTRPNNNTRRSITIGPGRAFYTTGEIIGDIRQAHC	35
AF258968	B	CCR5	CTRPNNNTRKGIHLGPGRAFYATGGIVGNIRQAHC	35
JQ779902	C	CCR5	CIRPGNNTRKSVRIGPGQTYFSTGEIIGNIRQAHC	35
HQ644902	B	CCR5	CTRPNNNTKKSIHIGPGRAFYTTGDIIGDIRKAHC	35
DQ516262	B	CCR5	CTRPNNNTRKGIHMGPGRAFFTTGAIIGDIRQAHC	35
DQ002288	B	CCR5	CIRPNNNTRKSINIGPGRAFYATGDIIGDIRQAHC	35
EU272192	B	CCR5	CTRPNNNTRKSINLGPGRAWYTTGQIIGDIRQAHC	35
AF384331	B	CCR5	CIRPNNNTRKGIHIGPGRTFYATGEIIGNIRQAHC	35
KF770449	C	CCR5	CTRPGNNTRRSVRIGPGQAFYTTGEIIGDIRAAHC	35
HQ644993	B	CCR5	CIRPNNNTRKSIPIGPGSAFYATGDIIGDIRQAHC	35
FJ376018	C	CCR5	CIRPNNNTRESIRIGPGQTFYATDIIGIRQAHC	33
EU272255	B	CCR5	CTRPNNNTRKRVSLGPGRVWYTTGQIVGDIRQALC	35
KF770379	C	CCR5	CTRPGNNTRKSIRIGPGQTFYATGDIIGDIRQAHC	35
HQ644857	B	CCR5	CIRPGNNTRKSIHIGPGRAFYATGDIIGDIRQAHC	35
DQ002336	B	CCR5	CTRPNNNTRKSIHIAPGRAFYATGAIIGNIRQAYR	35
FJ998109	B	CCR5	CTRPNNNTRRGIHMGPGRAFYATDIIGDIRQAHC	34
EU744051	B	CCR5	CTRPSNNTRKDIHIGPGRAFYATGEIIGDIRQAHC	35
HM239569	B	CCR5	CTRPNNNTRKGINIGPGRAFYATTDIIGDIRQAHC	35
AJ418508	B	CCR5	CTRLNNNTRKSIHMGPGRAFYATGEIIGDIRQAHC	35
AF541012	B	CCR5	CTRPNNNTRKSIPMGPGRAFYATGEIIGDIRQAHC	35
KC312506	B	CCR5	CTRPNNNTRKGIHIGPGRTFYATGEIIGNIRQAHC	35
AY010893	B	CCR5	CTRPNNNTRTSIHMGPGRALYATGDIIGDIRHAYC	35
AF307756	C	CCR5	CTRPNNNTRESIRIGPGQTFYATGDIIGDIRQAHC	35
JX973174	C	CCR5	CTRPGNNTRKSIRIGPGQTFFATNDIIGDIRQAYC	35
DQ109997	B	CCR5	CTRPNNNTRSIHIPGRAFYTGKIIGIRQAHC	31
DQ002318	B	CCR5	CTRPNNNTRKSIHMGPGRAFYTTGEIIGDIRQAYC	35
HM246198	B	CCR5	CTRPNNNTRKSINIGPGRAFYAIGDIIGDIRQAHC	35
AY010890	B	CCR5	CTRPNNNTRTSIHMGPGRAMYATGDIIGNIRQAYC	35
AY253304	C	CCR5	CVRPGNNTRKSVRIGPGQTFYATGDIIGDIRQAHC	35
U27443	B	CCR5	CTRPNNNTRKSITIGPGRAFYTTGQIIGDIRQAYC	35
AY842810	B	CCR5	CTRPNNNTRKSIHMGPGRAMYATGDIIGNIRQAHC	35
JF507953	B	CCR5	CTRPNNNTAKGIHLGPGRAMYATQRIVGDIRRAHC	35
HM179833	C	CCR5	CTRPGTNTRQSMRIGPGQTFYATGDIIGDIRQAHC	35
DQ061688	B	CCR5	CIRPNNNTRKGIHLGPGGALYATGAIIGDIRQAYC	35
DQ177190	B	CCR5	CTRPNNNTRRSINIGPGRAFYATGEIIGDIRQAYC	35
HM239541	B	CCR5	CTRPNNNTRRSINIGPGRAVYATGEIIGDIRVAHC	35
JN002002	B	CCR5	CTRPNNNTRKSIHIAPGRAFYATGEKIGDIRQAHC	35
FJ653402	B	CCR5	CTRPNNNTRKSIPIGPGRAFFATGDIIGDIRQAHC	35
DQ002167	B	CCR5	CTRPNDNTRKSIPMGPGRAFYATGAIIGDIRQAHC	35
AY510059	C	CCR5	CTRPGNNTRKSMRIGPGQAFYTTGDIIGDIRQAHC	35
DQ516164	B	CCR5	CTRPNNNTRRGIHIGPGRAFYAGDVTGDIRQAHC	34
U08802	B	CCR5	CTRPNNNTRKSIPLGPGQAWYTTGQIIGNIRQAHC	35
EF643684	B	CCR5	CIRPNNNTRKSIHIGPGRAFYATGEIIGNIRQAHC	35
DQ358761	C	CCR5	CTRPNNNTRESIRIGPGQTFYATGEIIGDIRQAYC	35
JQ779182	C	CCR5	CTRPGNNTRRSVRIGPGQTFYATGAIIGNIREAHC	35
KF716494	B	CCR5	CIRPNNNTRKGIHIGPGRVFYATGDIIGDIRQAHC	35

HQ678290	B	CCR5	CIRPGNNTRKSISIGPGRAFYATGGIIGDIRQAHC	35
JQ777168	C	CCR5	CIRPGNNTRRSVRIGPGQTFYATGDIIGDIRKAHC	35
AF153176	C	CCR5	CTRPSNNTRKSVRIGPGQTFYATGDIIGDIRQAHC	35
HQ377382	B	CCR5	CTRPNNNTRKGIHIGPGRAFYTTGDIIGDIRQAHC	35
DQ382361	C	CCR5	CTRPNNNTRESIRIGPGQTFFATGDIIGDIRQAYC	35
EF657896	B	CCR5	CIRPNNNTRKSIPMGPGKAFYATGGIIGNIRQAHC	35
JQ779231	C	CCR5	CIRPGNNTRKSIRIGPGQVFFTATDIIGNIREAHC	35
EF688449	B	CCR5	CTRPNNNTRRSINIGPGRAFYTTGEIIGDIRQAHC	35
GU945308	C	CCR5	CIRPNNNTRKSIRIGPGQTFYATGDIIGDIRQASC	35
JF508049	B	CCR5	CTRPNNNTRKSIHIGPGRAFYTTGEIIGNIRQASC	35
EU744052	B	CCR5	CTRPSNNTRRGIHIGPGRAFYTTGEIIGDIRQAHC	35
DQ002282	B	CCR5	CIRPNNNTRKSINIGPGRAFYATRDIIGDIRQAHC	35
JQ779091	C	CCR5	CTRPGNNTRKSVRIGPGQTFFATGDIIGDIRKAYC	35
FJ375995	C	CCR5	CTRPNNNTRKSIRIGPGQTLFATGAIIGNIRQAHC	35
DQ002339	B	CCR5	CTRPSNNTRKSIHLGLGRAFYATGEIIGDIRQAHC	35
AY887873	C	CCR5	CTRPNNNTRKSIRIGPGQTFYATGDIIGNIRQAHC	35
HM239567	B	CCR5	CTRPNNNTRRSIHIAPGRAFYATGEIIGNIRQAHC	35
HM239610	B	CCR5	CTRPNNNTRRSINIAPGRAFYATGDVIGDIRQAHC	35
EF600096	B	CCR5	CTRLNNNTRKGIHIGPGRAFYATGDIIGDIRQAHC	35
JN001997	B	CCR5	CTRPNNNTRKSIHITPGRAFYATGEIIGDIRQAHC	35
DQ002224	B	CCR5	CTRPSNNTRKSITIGPGKAFYATGEIIGDIRKAHC	35
HM246205	B	CCR5	CTSNNTRKSIHIGPGRAFYATGEIIGNIRQAHC	33
DQ061699	B	CCR5	CTRSNNNTRKGIHMGPGRAFYATGNIIGDIRQAYC	35
AY010847	B	CCR5	CTRPNNDTRKSIHLGPGSAFYAPGDIIGDIRQAHC	35
AF384326	B	CCR5	CTRPNNNTRKSISIGPGRAFYAHGDIVGDIRQAHC	35
KF770403	C	CCR5	CTRPGNNTRKSIRIGPGQTFYATGDIIGDIRKAHC	35
AF153131	C	CCR5	CTRPDNNTRKSMRIGPGQTFYATGDIIGNIRQAHC	35
HQ678259	B	CCR5	CTRPNNNTSQSIHIGPGRAFYATGRIIGDIRQAHC	35
AY833059	C	CCR5	CARPNNNTRKSMRIGPGQTFYATGDIIGDIRQAHC	35
DQ061483	B	CCR5	CTRPHNNTRKSIHIGPRRAFYTTEEIIRNIRQAHC	35
KC156338	C	CCR5	CTRPNTNIRKSMRIGPGQTFYATGDIIGNIRQAHC	35
KF770308	C	CCR5	CTRPDNNTRRSVRIGPGQAFYTNDIIGDIRQAHC	34
HM239528	B	CCR5	CERTNNNTRKSIHIGPGQALYATTNIIGDIRKAHC	35
HQ644886	B	CCR5	CIRPNNNTRKSIHIGPGRAFYATGDIIGNIRQAHC	35
AF153161	C	CCR5	CTRPNNNTRKSVRIGPGQTFYATGEIIGDIRQAHC	35
AF384248	B	CCR5	CTRPNNNTRKGIHIGPGRAFYTTGEIIGDIRKAHC	35
FJ653136	B	CCR5	CTRFNNNTRKSMHIGPGRAFYATGEIIGNIRQASC	35
JQ779094	C	CCR5	CTRPGNNTRKSVRIGPGQTFFATGDIIGDIRQAYC	35
AM156918	B	CCR5	CTRPSNNTRKGIHIGPGRAFYATEEITGDIRQAHC	35
HM239526	B	CCR5	CTRPSNNTRRGIHMAVGRAFYATGQIIGDIRQAHC	35
EU272330	B	CCR5	CTRPNNNTRKSIHLGQGRVWYTTGQIIGDIRQAHC	35
HQ678271	B	CCR5	CIRPNNNTRKSISIGPGRAFVTTGQIIGNIRQAHC	35
HQ644815	B	CCR5	CTRPNNNTRRSINIGPGRAFFATGDIIGDIRQAHC	35
DQ002098	B	CCR5	CTRPSNNTRKSITIGPGRAFYTAEVIGNIRAAYC	34
HQ644818	B	CCR5	CTRPNNNTRKSINIGPGRAFYATGEIIGDIRQAHC	35
GU945309	C	CCR5	CIRPNNNTRKSIRIGPGQTFYATGDVIGDIRQASC	35
AF384320	B	CCR5	CTRPNNNTRKGIHIRPGGAIYATGAITGDIRQAHC	35
U08780	B	CCR5	CTRPNNNTRKSIHLGWGRAFYATGEIVGDIRQAHC	35
JF507746	B	CCR5	CIRPNNNTRKGIHIGPGRTFFATGEIIGDIRRAYC	35
DQ516264	B	CCR5	CTRPNNNTRKGIHMGPGRAFYTTGAIIGDIRQAHC	35
HQ644938	B	CCR5	CTRPNNNTRKSINIGPGRAIYATGEIIGDIRQAHC	35
EU576867	B	CCR5	CTRPNNNTRRGIHMGPGKAYFTGEIIGDIRQAHC	34
DQ382374	C	CCR5	CTRPNNNTRKSMRIGPGQAFYAMGDIIGDIRQAHC	35
AF082381	B	CCR5	CTRPNNNTRKGIHIGPGRAFYATDIIGDIRQAHC	34
KF770344	C	CCR5	CTRPGNNTRRSIRIGPGQAFYATGGIIGDIRQAHC	35
KC473833	B	CCR5	CVRPNNNTRQGIHMGPGRTFYTTGGIIGDIRQAYC	35
EU744161	B	CCR5	CTRPNNNTRRSIHIGPGRAFYATGDIIGNIRQAHC	35
AF541079	B	CCR5	CTRPSNNTRKGIHIGLGRRFYTTEIVGDIRKAYC	34
FJ653098	B	CCR5	CTRPNNNARKSINIGPGRAFYAATDIIGDIRQAHC	35
AF153141	C	CCR5	CVRPNNNTRKSIRIGPGQTFYANDIIGDIRQAHC	34
AF153156	C	CCR5	CVRPSNNTRKSVRIGPGQTFYATKDIIGDIRQAHC	35
AY010895	B	CCR5	CTRPNNNTGKSMPMGPGRAFYSTGEIMGHLRKTYS	35
KC312574	B	CCR5	CERPNNNTRKSIRIGPGSTFYATGEIIGNIRQAHC	35
HQ708048	C	CCR5	CTRPNNNTRKSVRIGPGQTFYATGGIIGDIRRAYC	35
DQ516285	B	CCR5	CTRPNNNTRKSIHMGPGRAFYATGEIIGNIRQAHC	35
EU272261	B	CCR5	CTRPNNNTRKSINLGPGRAWYATGQIIGDIRQAHC	35
EU272281	B	CCR5	CTRPNNNTRKSIPLGPGKAWYTTGDIIGDIRQAHC	35
DQ061481	B	CCR5	CTRPNNNTRKSISIGPGRAFYTTGEIIGEIRQAYC	35
AY510057	C	CCR5	CTRPNNNTRKSVRIGPGQAFYATGDIIGNIRQAYC	35
KF384804	B	CCR5	CTRPGNNTSKGIHIGPGRALHVTRTRGKIIGDIRQA	38

HQ377487	B	CCR5	CTRPGNNTRKSIPIGPGRAFYATGDVIGDVRKAHC	35
HM179824	C	CCR5	CTRPGNNTRQSMRIGPGQTFYATGNIIGDIRQAHC	35
EU786680	B	CCR5	CTRPNNNTRKSIHIGPGRALYAQDIIGDIRQAHC	34
EF657898	B	CCR5	CIRPNNNTRKSIPMGPGKAFYATGSIIGNIRQAHC	35
HM179924	C	CCR5	CTRPNNNTRKSIRIGPGQTFYATGDIIGDIRQAYC	35
M26727	B	CCR5	CTRPNNNTRNRISIGPGRAFHTTKQIIGDIRQAHC	35
AF543910	C	CCR5	CIRPNNNTRKSIRIGPGQTFYATNDIIGDIRQAHC	35
FJ376028	C	CCR5	CTRPGNNTRESIRIGPGQTFYATGDIIGNTRQAYC	35
EU577210	B	CCR5	CTRPNNNTRKSVHIGPGKVFYAGEIIGDIRQAHC	34
EF688438	B	CCR5	CTRPNNNTRKAIHLGPGRAFYTTGGIIGDIRQAHC	35
DQ904344	C	CCR5	CTRPNNNTRKSVRIGPGQTFYATGDIIGDIREAHC	35
AJ418520	B	CCR5	CTRPDNNTRKSIHIGPGRAFYATGDIIGNIRQAHC	35
AY887878	C	CCR5	CTRPNNNTRQSMRIGPGQTFYATGDIIGDIRQAHC	35
EU272290	B	CCR5	CTRPSNNTRKSINLGPGRAWYTTGQIIGDIRQAHC	35
EU908220	C	CCR5	CTRPNNNTRQSIRIGPGQVFYATGAIIGDIRQAYC	35
AY842792	B	CCR5	CTRPNNNTRKSIHMGPGSVWYATGEIIGDIRQAHC	35
EU744005	B	CCR5	CTRPNNNTRKSISIGPGRAFYATGDIVGDIRKAHC	35
AY124978	B	CCR5	CTRPSNNTRKSINIGAGRAIYATGDIIGDIRQAYC	35
KC312586	B	CCR5	CVRPNNNTRKSIHLGPGSAFYATGKIIGNIRQAHC	35
HQ678282	B	CCR5	CTRLNNNTRKSIRIGPGSTFYTSNIIGDIRQAYC	34
DQ002340	B	CCR5	CTRPNNNTRKSIHIAPGRAFYATGDIIGNIRQAYC	35
DQ002281	B	CCR5	CTRPNNNTKRSVNIVPGRAIYTTDIIGDIRQAHC	34
KC156120	C	CCR5	CTRPNNNTRKSIRIGPGQAFFATGDIIGDIREAHC	35
U52953	C	CCR5	CTRPNNNTRKSIRIGPGQAFYATGEIIGDIRQAHC	35
AY842817	B	CCR5	CTRPNNNTRKSIPMGPGRAMYATGDIIGDIRKANC	35
AF258977	B	CCR5	CTRPNNNTRKGIHLGPGRAFYATGGIVGDIRQAHC	35
KC156437	C	CCR5	CTRPNNNTRKSVRIGPGQTFYATGEIIGDIREAYC	35
DQ435683	C	CCR5	CIRPGNNTRRSIRIGPGQAFYAMGDIIGNIREAHC	35
EF688450	B	CCR5	CTRPNNNTRKSIHIGPGRAFYGTDIIGDIRQAHC	34
HQ708025	C	CCR5	CTRPNNNTRKSVRIGPGQTFYATGDIIGDIRQAYC	35
JF896853	B	CCR5	CTRPNNNTRKSIHMGPGKTFFATGDIIGDIRQAHC	35
DQ516304	B	CCR5	CTRPNNNTRKSIHIGPGRALYTTGEIIGNIRQAHC	35
EF688457	B	CCR5	CTRPNNNTRKSIPMGPGRAFYTTGEIIGDIRQAHC	35
EF643662	B	CCR5	CTRPNNNTRRSISIGPGRAFYATGEVIGDIRQAHC	35
EU744065	B	CCR5	CTRPNNNTRKSIHMGPGKTFYATGDIIGDIRQAHC	35
JF507805	B	CCR5	CMRPGNNTRKSIAIGPGRAFYAGEIIGDIRKAHC	34
AJ418534	B	CCR5	CARPHNNTRKSIHIGPGRAFYATGQITGDIRQAYC	35
AF541039	B	CCR5	CTRPSNNTRKSIPMGPGKAFYATGDIIGDIRQAHC	35
AF384294	B	CCR5	CTRPNNNTRKSIHIGPGRAVYATGQIIGDIRQAHC	35
AY842805	B	CCR5	CTRPNNNTRRSIHMGPGSVLYATGEIIGDIRQAHC	35
DQ002149	B	CCR5	CTRPGNNTRRGIHIGPGRAFYTTGEIIGDIRQAHC	35
HM239579	B	CCR5	CTRPNNNTRKSIIGPGRAYFATGIIGDIRKAHC	33
AF391245	C	CCR5	CTRPNNNTRRSIRIGPGQAFYTNDIIGDIRQAHC	34
AY887865	C	CCR5	CIRPGINKRKRIRIGLRYAFFATDNRIGKAHC	32
FJ670531	B	CCR5	CIRPNNNTRKSIHMGPGRAFYATGDVIGDIRKAYC	35
EU744008	B	CCR5	CTRPNNNTRKSISIGPGRAFYATGDIIGDIRKAHC	35
DQ869022	B	CCR5	CTRPGNNTRKSIHIAPGRAFYATGDIIGDIRQAHC	35
DQ002231	B	CCR5	CTRPSNNTRKSIPIGSGRAFYATGDIIGDIRKAHC	35
HM239613	B	CCR5	CTRPNNNTRKGITMGPGKAFYVTGGIVGDIRQAHC	35
AF199036	B	CCR5	CTRPNNNTRKSIHIGPGRALYATGDIVGDIRQAHC	35
DQ904347	C	CCR5	CTRPNNNTRKSIRIGPGQTFYATGDIIGNIREAHC	35
AY842807	B	CCR5	CTRPNNNTRKSIHMGPGSVLYATGEIIGDIRQAHC	35
DQ002264	B	CCR5	CTRPNNNTRKSIPIGPGRAFYTTGEIIDIRQAHC	34
HQ644868	B	CCR5	CTRPGNNTRKSIPMGPGRAFYATGDIIGDIRKAHC	35
U08455	C	CCR5	CTRPNNNTRKSVRIGPGQTFYATGAIIGDIRQAHC	35
AY010794	B	CCR5	CIRPNNNTRKSIQMGPGKAFYATGDIIGDIRQAYC	35
HQ644921	B	CCR5	CTRPNNNTRKSIPIGPGRAFYTTGDIIGDIRQAHC	35
JN002053	B	CCR5	CTRPNNNTRKSIHMGPGKAFYTTGVIGDIRQAHC	34
U45299	B	CCR5	CTRPNNNTRRGVHIGPRRGFYTTEIIGNIRQAHC	34
U23487	B	CCR5	CTRPSNNSRKSIYIGPGRRFHVTRAVTGDIRQAHC	35
FJ653358	B	CCR5	CTRPNNNTRRGIHIGPGRAFYATDIIGDIRQAHC	34
HM246193	B	CCR5	CIRPNNNTRKSIHIAPGRAFYATGNIIGDIRKAYC	35
U08712	B	CCR5	CTRPNNNTRKSIHMGWGRTFYATGEIIGDIRQAHC	35
HM239588	B	CCR5	CTRPNNNTRKSIHIGPGRAFYATGDIIGEIRQAYC	35
DQ061678	B	CCR5	CIRPNNNTRKGIHLGPGGALYATGGIIGDIRRAYC	35
DQ002026	B	CCR5	CIRPHNNTRKSIPIGPGRAFYTTGEIIGDIRQAHC	35
AF355727	B	CCR5	CTRPNNNTRKSIPLGPGRAFYATGDIIGNIRKAHC	35
EU575305	B	CCR5	CTRPGNNTRKSITIGPGRAFYATGDIIGDIRQAHC	35
U79719	B	CCR5	CTRPNNNTRKGIHIGPGAAFYATGEIIGDIRQAHC	35
AF310127	B	CCR5	CTRPNNNTRKSIHLGAGRALYTGEIIGDIRQAHC	34

AY887886	C	CCR5	CIRPGNNTRKSVRIGPGQTIYATGDIIGDIRQAHC	35
AF022258	B	CCR5	CTRPNNNTRKSISIGPGRAFYTTGEIIGDIRQAHC	35
AJ418505	B	CCR5	CTRPNNNTRKSIPIGPGRAFYTTGEIIGDIRKAHC	35
EF657905	B	CCR5	CIRPNNNTRKSIPMGPGRAFYATGDIIGNIRQAHC	35
KC156318	C	CCR5	CTRPNDNIRKSMRIGPGQTFYATGDIIGNIRQAHC	35
AF153172	C	CCR5	CVRPNNNTRKSIRIGPGQTFYATNGIIGDIREAHC	35
HM239595	B	CCR5	CTRPNNNTRRSIRIGPGSYFATGDIIGDIRKAYC	34
AJ418480	B	CCR5	CTRPNNNTRKSIPIGPGRALYATGDIIGEIRQAFC	35
KF770391	C	CCR5	CIRPGNNTRKSMRIGPGQTFYATGDIIGDIRKAHC	35
HM246188	B	CCR5	CTRPNNNTRKSIHIVPGGAFYATGDIIGDIRQAHC	35
EU744075	B	CCR5	CIRYNNNTRKSIPLGPGRAFYATGDIIGNIRQAQC	35
GU945313	C	CCR5	CTRPGNNTRKSIRIGPGQTFYATGGIIGNIRQAHC	35
HM246190	B	CCR5	CSRPNNNTKSIPIGPGAFYATGDIIGDIRQAHC	33
AF153164	C	CCR5	CIRPGNNTRKSIRIGPGQAFYATGDIIGDIRQAHC	35
DQ061463	B	CCR5	CTRPNNNTRKSIHIGPGRAFYTTGEIIGDIRKAHC	35
AB553911	B	CCR5	CERPNNNTRRSIQIGPGRAWFEAEDIIGDIRKAHC	35
DQ516149	B	CCR5	CTRPNNNTRRDIHIGPGRAFYATGGIIGNIRQAHC	35
DQ002342	B	CCR5	CTRPNNNTRKSIHIGPGSAFYATGDIIGNIRQAYC	35
AF355726	B	CCR5	CTRPSNNTRKSIHLGPGRAFYATGDIIGNIRKAHC	35
DQ061452	B	CCR5	CTRPNNNTRKSIHIGPGRAFYTTGEIMGDIRKAHC	35
AF258994	B	CCR5	CTRPNNNTRKGIHLGPGRAFYATGEIVGDIRKAHC	35
EU272217	B	CCR5	CIRPNNNTRKSIPLGPGKAWYTTGQIIGDIRQAHC	35
HQ708068	C	CCR5	CIRPSNNTRTSMRIGPGQTFFATGDVTGDIRQAYC	35
EU744015	B	CCR5	CTRPNNNTRKSIHIGPGRAFYTTGEIIGEIRQAHC	35
GU455522	B	CCR5	CIRPNNNTRKSIPMGPGKAFYTTGDIIGDIRQAHC	35
JQ779893	C	CCR5	CIRPGNNTRKSIRIGPGQTYFSTGEIIGNIRQAHC	35
KF766537	C	CCR5	CIRPNNNTRKSIRIGPGQTFYATGEIIGDIRQAHC	35
KF770295	C	CCR5	CIRPNNNTRKSVRIGPGQTFYATGEIIGDIRKAHC	35
DQ002148	B	CCR5	CTRPGNNTRRGIHIGPGRAFYTTGEIIGNIRQAHC	35
KC156326	C	CCR5	CNRPHNNTRKSMRIGPGQAFYATGDITGDIRKAHC	35
EU744078	B	CCR5	CTRYNNNTRKSIHLGPGRAFYATGDIIGDIRQAQC	35
EU293450	C	CCR5	CTRPSNNTRKSVRIGPGQAFFATGEIIGDIRQAHC	35
EU272222	B	CCR5	CIRPNNNTRKSIPIGPGKAWYTTGQIIGDIRQAHC	35
DQ516241	B	CCR5	CTRPNNNTRKGIHIGPGRAFYTTGEIIGDIRQAYR	35
DQ061524	B	CCR5	CTRPNNNTRRSIHIAPGSAFYATGDIIGDIRQAHC	35
HQ644904	B	CCR5	CTRPNNNTRKSIPIGPGRAFYTTGDIIGDIRKAHC	35
JN002050	B	CCR5	CTRPDNNTRKGIHLGPGGTFFATGAIIGDIRQAHC	35
EU576298	B	CCR5	CTRPNNNTRKGIHIGPGKTFYTTGGIIGDIRQAYC	35
DQ869026	B	CCR5	CTRPNNNTRKSIHIAPGRPFYATGDIIGDIRQAHC	35
AF153138	C	CCR5	CTRPGNNTRKSVRIGPGQTFYATGEIIGNIRQAHC	35
DQ411850	C	CCR5	CTRPNNNTRKSIRIGPGQTVYATNDIIGDIRQAHC	35
HQ644927	B	CCR5	CMRPNNNTRKSIPIGPGRAFYTTGDIIGDIRQAHC	35
KC473843	B	CCR5	CTRPNNNTRRGIQMGPGRAVYATGDIIGDIRQAHC	35
EF688452	B	CCR5	CTRPNDNTRKGINIGPGRAFYATGEIIGDIRQAHC	35
AF254783	C	CCR5	CTRPGNNTRKSIRIGPGQAFFATGAIIGDIRKAYC	35
HM239529	B	CCR5	CTRPSNNTRKSITIGPGRAFYTTGQIIGNIRQAHC	35
KC473841	B	CCR5	CTRHSNNTRRSIRIGPGQAFYATGEIIGDIRKAHC	35
FJ653126	B	CCR5	CTRFNNNTRKSIHIGPGRAFYATGDIIGNIRQASC	35
KC156124	C	CCR5	CTRPNNNTRTSIRIGPGQTFYATGDIIGDIRQAYC	35
HM246228	B	CCR5	CTRPNNNTRKSIHIAPGRAFYATGDIIGDIRQAYC	35
AY887879	C	CCR5	CMRPNNNTRKSVRIGPGQTFYATGDIIGNIRQAHC	35
HQ644887	B	CCR5	CTRPNNNTRKSIHIGPGRAFYTTGEIIGKIRQAHC	35
DQ061752	B	CCR5	CTRPNNNTRRSITLGPGRAYYATGDIIGDIRQAHC	35
JF508128	B	CCR5	CTRPNNNTRRSIYIGPGRTFYATGEIIGDIRQAHC	35
HM239566	B	CCR5	CTRPSNNTRKGIHMAVGRAFYTTGQIIGNIRQAHC	35
DQ388517	C	CCR5	CTRPNNNTRKSIRIGPGQSFYATGEIVGNIREAHC	35
AF153181	C	CCR5	CIRPSNNTRRSVRIGPGQTFYATGEITGDIRQAHC	35
JF508018	B	CCR5	CTRPNNNRRKSINIGPGRAFYTTGAIIGDIRQAHC	35
AY010831	B	CCR5	CTRPNSNTRKGIHIGPGSAIYATGDIIGDIRQAHC	35
JX140654	B	CCR5	CTRPGNNTRRSITMGPGKVFYTNEIIGNIRRAYC	34
JF507854	B	CCR5	CTRPGNNTRRSINIGPGRAFYTGEIIGDIRQAHC	34
HQ644992	B	CCR5	CTRPNNNTRKSIPIGPGRAFYATGEIIGDIRQAHC	35
HM179723	C	CCR5	CTRPNNNTRKSMRIGPGQTFYATGEIIGNIREAHC	35
HQ708049	C	CCR5	CTRPNNNTRKSIRIGPGQAFYATGAVTGDIRKAYC	35
AY736825	C	CCR5	CTRPGDNRRRSVRIGPGQTFYATGEIIGNIREAHC	35
AY255825	C	CCR5	CTRPNNNTRRSIRIGPGQTFYATGEIIGNIREAHC	35
KC156396	C	CCR5	CIRPGNNTRRSVRIGPGQTYFSTGEIIGNIRQAHC	35
JF507916	B	CCR5	CTRPNNNTRKSISIGPGRAFYATGEIIGDIRQAHC	35
AF384291	B	CCR5	CTRPNNNTRKSIPIGPGRAFYARGDIIGDIRQAHC	35
EU272220	B	CCR5	CIKPNNNTRKSIPIGPGKAWYTTGQIIRDIRRAHC	35

JN002015	B	CCR5	CTRPNNNTRKGIHIGPGRAFYATGDIVGDIKQAHC	35
HQ645006	B	CCR5	CTRPNNNTRRSISIGPGRAFYATGDIIGDIRRAQC	35
DQ235634	C	CCR5	CTRPNNNTRKSVRIGPGQAFYATNDIIGDIRQAHC	35
AF259010	B	CCR5	CTRPNNNTRKGIHMGPGRAFYATGEIVGDIRKAHC	35
EU161645	C	CCR5	CTRPNNNTRTSTRIGPGQAFYATGDIIGDIRQAHC	35
DQ177198	B	CCR5	CTRPNNNTRKSINIGPGRAFYSTGAIIGNIRQAHC	35
EF117268	C	CCR5	CTRPNNNTRKSIRIGPGQTFYATGDIIGNIRQAYC	35
HQ699981	C	CCR5	CTRPNNNTRKSVRIGPGQAFYATGDIIGNIREAHC	35
EF643664	B	CCR5	CTRPNNNTRKGISIGPGRAFYTTGEVIGDIRQAHC	35
KF770347	C	CCR5	CTRPNNNTRRSIRIGPGQAIYTTGGIIGDIRQAHC	35
AF021502	B	CCR5	CTRPNNNTRKSIPIGPGRAFYTTGQIIGDIRQAYC	35
AF310109	B	CCR5	CTRPNNNTRKSIHIGAGKALYTGEIIGDIRQAHC	34
AF153173	C	CCR5	CTRPNNNTRKSIRIGPGQTFYANDIIGDIRQAHC	34
JN002045	B	CCR5	CTRPNNNTRKSIPIGPGGAFYATGDIIGDIRQAHC	35
DQ110002	B	CCR5	CIRPNNNTRKSIPMGPGRAFYATGDIIGDIRQAHC	35
JN002019	B	CCR5	CTRPNNNTRKGIHIGPGRAFYATGDIVGDMRQAHC	35
HQ644936	B	CCR5	CTRPNNNTRKSINIGPGRALYTTGEIIGDIRQAHC	35
JQ779245	C	CCR5	CIRPGNNTRKSIRIGPGQVLFATTDIIGNIREAHC	35
DQ435682	C	CCR5	CRRPNNNTRKSIRIGPGQAFYATNDIIGDIRQAHC	35
AF258959	B	CCR5	CIRPSNNTRTSIHLGPGQAVYATGEIIGNIRQAHC	35
EU786676	B	CCR5	CTRPNNNTRKSITIGPGRAFYATGDVIGDIRQAHC	35
DQ382376	C	CCR5	CTRPGNNTRKSVRIGPGQVFYATGDIIGDIRQAHC	35
AF022281	B	CCR5	CTRPNNNTRKSISIGPGRAFYATGDIIGDIRQAHC	35
U08699	B	CCR5	CTRPNNSTRKSIHMGWGRAFYATGEIIGNIRQAHC	35
KC156458	C	CCR5	CTRPNNNTRKSVRIGPGQTFYATGEIIGNIREAYC	35
DQ002312	B	CCR5	CTRPNNNTRKSINIGPGRAWYTTGDIIGDIRKAHC	35
HQ644915	B	CCR5	CTRPNNNTRKSIHIGPGRAFYTTEIIGDIRKAYC	34
HQ644803	B	CCR5	CTRPNNNTRRSINIGPGRAFFATGEIIGDIRQAHC	35
EU786677	B	CCR5	CTRPNNNTRKSIRIGPGQAFYTTGEIIGDIRQAHC	35
AF259020	B	CCR5	CTRPGNNTRKSIHIGPGRAFYTTGQIIGKIRQAHC	35
HQ708020	C	CCR5	CIRPDNNTRKSIRIGPGQTFYATGDIIGNIRKAYC	35
HQ644793	B	CCR5	CTRPNNNTRRSIHIGPGRAFYTGEIIGNIRQAHC	34
JX140655	B	CCR5	CTRPNNNTRKGIHIGPGKTFFATDIIGDIRQAHC	34
KF770439	C	CCR5	CIRPGNNTSRSIRIGPGQTFYATGRIVGDIRQAHC	35
HM239515	B	CCR5	CTRPSNNTRKSIHMGPGRVLFATGEIIGNIRQAHC	35
DQ222217	B	CCR5	CTRPNNNTRKSIHVGPGKAIYTTEIIGNIRQAHC	34
U08450	B	CCR5	CIRPNNNTRRSIHMGLGRAFYATGDIIGDIRQAHC	35
HQ644979	B	CCR5	CTRPNNNTRRSINIGPGRAIYATGEIIGDIRQAHC	35
HQ644805	B	CCR5	CIRPNNNTRRSINIGPGRAIFTTGEIIGDIRQAHC	35
DQ061742	B	CCR5	CTRPNNNTRRSISLGPGRAYYATGDIIGDIRQAHC	35
JX140668	C	CCR5	CIRPNNNTRKSIRIGPGQTFYANNIIGDIRQAYC	34
AY887867	C	CCR5	CTRPNNNTRRSVRIGPGQAFYGTDIIGDIRQAYC	34
EU272262	B	CCR5	CTRPNNNTRKSINLGPGAWYATGQTIGDIRQAHC	34
HM239577	B	CCR5	CTRPNNNTRRSIHIAPGKTFYATGAIVGDIRQAYC	35
JQ779230	C	CCR5	CTRPNNNTRKSIRIGPGQAFFATTGIIGNIRQASC	35
HQ644882	B	CCR5	CTRPNNNTRKSINIGPGRAFYATGDIIGNIRQAHC	35
DQ002137	B	CCR5	CTRPNNNTRKSIHIRPGKAFYATGDIIGDIRQAHC	35
DQ061685	B	CCR5	CIRPNNNTRKGIHLGPGGALYATGGIIGDIRQAYC	35
JF507888	B	CCR5	CTRPSNNTRKSIHIGPGRALYATDIIGDIRKAYC	34
EF657900	B	CCR5	CIRPNNNTRKSIPMGPGKAFYATGDIIGNIRQAHC	35
AY010808	B	CCR5	CTRPNNNTRKGILLGPGSAFYATGDIIGDIRQAHC	35
AY887866	C	CCR5	CTRPGNNTRKSMRIGPGQTFYATGDIIGDIRQAHC	35
AY010886	B	CCR5	CTRPNNNTRTRIHMGPGRAMYATGDIIGNIRQAYC	35
DQ235620	C	CCR5	CTRPNNNTRKSMRIGPGQTFFATGEIIGDIRQAHC	35
EU578364	B	CCR5	CTRPNNNTRKSITIGPGSVFYTGEIIGDIRRAHC	34
AF391231	C	CCR5	CTRPNNNTRKSVRIGPGQAFYATNDVIGNIRQAHC	35
DQ061817	B	CCR5	CTRPNNNTRKGIHMGSGKVFYATGQIIGDIGQAHC	35
AF384312	B	CCR5	CTRPYEKKKSRRIRIGPGRTFHTTGSIGDIRRAHC	35
HQ645010	B	CCR5	CTRPNNNTRKSIPIGPGRAIYTTGQIIGDIRQAHC	35
EU575924	B	CCR5	CTRPGNNTRKSIHIAPGRTFYATGEIIGDIRRAHC	35
AF384274	B	CCR5	CTRPNNNTRKSIHMGPGGSFYATGDIIGNIRQAHC	35
GU945317	C	CCR5	CIRPNNNTRQSIRIGPGQVFYATGDIIGDIRQAYC	35
DQ061561	B	CCR5	CVRPNNNTRKSIPIGPGRAFYTTGEIIGDIRQAHC	35
DQ382380	C	CCR5	CTRPNNNTRTSVRIGPGQAFYATNGIIGDIRQAHC	35
DQ002203	B	CCR5	CTRPSNNTRKSIPIGPGRAFYATGEIIGDIRQAHC	35
HQ644926	B	CCR5	CTRPNNNTRKSIHIGPGKALYTTEIIGNIRQAHC	34
AY669741	C	CCR5	CTRPNNNTRKSMRIGPGQTFYATGDIIGDIRQAYC	35
AJ888842	B	CCR5	CTRPNNNTRKSITIGPGRAFYTTGEIIGDIRKAHC	35
AY835434	B	CCR5	CTRPNNNTRKSIHMGPGAAFYARGEVIGDIRQAHC	35
AY043175	C	CCR5	CTRPNNNTRKSVRIGPGQTFYATGEIIGDIREAHC	35

JF507936	B	CCR5	CTRPNNNTAKGIHIGPGRAMYATERIVGNIRRAHC	35
EF688446	B	CCR5	CTRPNNNTRKSIRIGPGSAFYATGDIIGDIRQAHC	35
AM156920	B	CCR5	CTRPNNNTRRSIHIGPGRAFYTRDIIGNIRQAHC	34
DQ002165	B	CCR5	CTRPNNNTRKSISMGPGRAFYATGAIIGDIRQAHC	35
DQ061780	B	CCR5	CTRPNNNTRKGIHIGPGRAFYAAGEIIGNIRQAHC	35
JQ779243	C	CCR5	CIRPGNNTRKSIRIGPGQVFFATDIIGDIRKAHC	34
AJ418484	B	CCR5	CTRPNNNTRKGIHIGPGRALYATGDIIGKIRQAHC	35
EF579976	B	CCR5	CERPGNNTSKGIHIGPGRAFYATVNIIGDIRKAHC	35
DQ235623	C	CCR5	CTRPNNNTRKSVRIGPGQTFYATGDIIGNIRQAHC	35
JF896841	B	CCR5	CTRPNNNTRKSIPMGPGKAFYATGIIGDIRQAHC	34
JF507878	B	CCR5	CTRPSNNTRQGIHIGPGRAIYTTDIIGDIRKAYC	34
HM246187	B	CCR5	CTRPNNNTRKSISIGPGRAFYTTGDIIGNIRQAYC	35
HQ678292	B	CCR5	CTRPNNNTRKSIRIGRGRAFYAPGEIIGDIRKAYC	35
AF021523	B	CCR5	CTRPNNNTRKSITIGPGRAFYTTGQIIGDIRKAYC	35
KF770371	C	CCR5	CIRPGNNTRRSVRIGPGQTFYATGDIIGDIRQAHC	35
EU575170	B	CCR5	CVRPNNNTRRSITIGPGRAFYTGEIIGNIRKAYC	34
JN687739	B	CCR5	CTRPSDNTRKSIHMGWGRAFYATGEITGDIRQAHC	35
HM215421	C	CCR5	CTRPNNNTRKSIRIGPGQTFYATGEIIGDIRQAHC	35
DQ869025	B	CCR5	CTRPNNNTRKGIHIGPGRAFYATGEIIGNIRQAYC	35
EU272207	B	CCR5	CTRPNNNTRKSIHIGPGRAWYATGEIIGDIRQAHC	35
HQ708021	C	CCR5	CIRPNNNTRKSVRIGPGQTFYATGDIIGDIRQAYC	35
AY842793	B	CCR5	CTRPNNNTRKSIHMGPGRVWYTTGGIIGDIRQAHC	35
DQ002156	B	CCR5	CTRPNNNTRKSIPMGPGRAFYATGAIIGDIRQAHC	35
DQ061496	B	CCR5	CTRPSNNTRKSIHIGPGRAFYTTGEIIGNIRQAHC	35
AF153139	C	CCR5	CVRPNNNTRKSVRIGPGQTFYATGDIIGDIRQAHC	35
DQ235641	C	CCR5	CIRPNNNTRKSIRIGPGQAFYATGDIIGDIRKAYC	35
HQ644922	B	CCR5	CIRPNNNTRKSIHIGPGRAFYTTEIIGNIRKAYC	34
HM368253	B	CCR5	CTRPNNNTRKSIHIQPGRAFYATDIIGDIRQAHC	34
AF384251	B	CCR5	CTRPNNNTRKSIHMGPGRAFYTTGNIIGDIRKAHC	35
FJ375982	C	CCR5	CTRPNNNTRKSVGMGPGRAIYATGDIIGDIRQAHC	35
JF507901	B	CCR5	CTRPSNNTRQGIHIGPGRAIYTTNIIGDIRKAYC	34
FJ376013	C	CCR5	CTRNNNTRKSIRIGPGQYATGDIIGDIRQAHC	32
FJ853622	B	CCR5	CTRPNNNTRKSIHLGAGKAIYTTGAIIGNIRQAHC	35
EU744025	B	CCR5	CTRPNNNTRKSIHIGPGRALYATGEIIGNIRQAHC	35
AF082354	B	CCR5	CTRPNNNTRRSINIGPGRAFYTTGDIIGDIRQAHC	35
DQ002041	B	CCR5	CIRPHNNTRKSIHIGPGRAFYTTGEIIGDIRQAHC	35
KF770329	C	CCR5	CTRPNNNTRKSVRIGPGQTFYATGEIIGDIRRAHC	35
HQ644855	B	CCR5	CTRPGNNTRKSINIGPGRAIYATGDIIGNIRQAHC	35
EU575457	B	CCR5	CTRPSNNTRRSINIGPGKAFYATGEIIGNIRQAHC	35
HQ377469	B	CCR5	CIRPNNNTRKSISMGPGRAFFATGEIIGNIRQAHC	35
AY124975	B	CCR5	CTRPNNNTRKGIHMGPGGALYATGAIIGNIRQAHC	35
AY158533	C	CCR5	CTRPNNNTRKSMRIGPGQTFFATGDIIGNIRQAHC	35
EF643657	B	CCR5	CTRLNNNTRRSINIGPGRAWYTTGEVIGDIRKAHC	35
DQ002228	B	CCR5	CTRPSNNTRRSITIGPGRAFYATGEIIGDIRKAHC	35
HM239594	B	CCR5	CTRPNNNTRKSIPIGPGSAFYATGDIIGDIRQAHC	35
HM246197	B	CCR5	CTRLNNNTRKSIHIGPGQAFYATGAIIGIRQAHC	34
FJ653220	B	CCR5	CTRPGNNTSKSIHIGPGRAFDATKTITGDIRQAHC	35
EF117265	C	CCR5	CTRPNNNTRKSIRIGPGQTFYATGEIIGNIRQAHC	35
JN002044	B	CCR5	CTRPNNNTRKSIPMGPGKAFYATGDIIGDMRQAHC	35
AF258982	B	CCR5	CTRPNNNTRKGIHLGPGRAFYATGEIIGNIRQAYC	35
DQ109995	B	CCR5	CTRPNNNTRKSINIGPGRAWYTTGQIIGDIRQAHC	35
HM179818	C	CCR5	CTRPGNNTRQSMKIGPGQTFYATGDIIRDIRQAHC	35
HM239558	B	CCR5	CTRPNNNTRKSIHIGPGRAFYATGEIGDIRQAHC	34
AF310112	B	CCR5	CTRPNNNTRKSIHIGPGKALYTGEIIGDIRQAHC	34
AY426112	B	CCR5	CTRPNNNTRKSIHIGPGRALYTTGKIIGDIRQAHC	35
DQ869024	B	CCR5	CTRPNNNTRKGIHMGPGKTLYATGEIIGDIRQAHC	35
DQ235633	C	CCR5	CTRPNNNTRQSIRIGPGQAFFAAKDIIGDIREAHC	35
DQ002179	B	CCR5	CTRPNNNTRRSITIGPGRAFYATDIIGDIRQAQC	34
KC312595	B	CCR5	CVRPHNNTRKSIHIGPGRTFYATGEVIGDIRQAHC	35
AY173955	B	CCR5	CIRPNNNTRKSIHIGPGRAFYATGDIIGDIRQAHC	35
FJ653210	B	CCR5	CTRPNNNTRKGIHIGPGRAYYATEKITGDIRQAHC	35
AY043173	C	CCR5	CTRPNNNTRKSIRIGPGQTFYATGEIIGNIREAHC	35
KC312481	B	CCR5	CTRPNNNTRKGIHIGPGRTFYATGQIIGNIRQAHC	35
AF384329	B	CCR5	CTRPNNNTRKGIAIGPGRAVYATEKIVGDIRQAHC	35
HM215397	B	CCR5	CTRPNNNTRKSISLGPGRAWYATGQIIGNIRQAHC	35
FJ653122	B	CCR5	CTRFNNNTRKGIHIGPGRAFYATGDIIGNIRQASC	35
HQ678284	B	CCR5	CTRPNNNTRKSIHIAPGRAFYATGAIIGDIRKAYC	35
HQ708079	C	CCR5	CTRPNNNIRKGYTIGPGRAFFATGDIIGDIRQADC	35
AF384270	B	CCR5	CTRPGNNTRKSIHIAPGSAFYATGVIIGDIRKAHC	35
HQ644814	B	CCR5	CIRPNNNTRRSINIGPGRAIFRTGEVIGDIRQAHC	35

AY124979	B	CCR5	CTRPSNNTRKSINIGAGRAIYAAGDIIGNTRQAYC	35
HQ644989	B	CCR5	CTRPNNNTRKSIPIGPGSAFYATGEIIGDIRQAHC	35
HQ708064	C	CCR5	CTRPHNNTRKSMRIGPGQTFYATEEVIGDIRQAYC	35
DQ235629	C	CCR5	CTRPSNNTRTSVRIGPGQTFYATGDIIGDIRQAHC	35
KC156284	C	CCR5	CTRPNNNTRQSMRIGPGQTFYATGAIIGNIRQAHC	35
AJ418536	B	CCR5	CTRPHNNTRKSIHIGPGRAFYATGQITGDIRQAYC	35
AF258962	B	CCR5	CTRPNNNTRKSIHLGPGRAFFTTGEIIGNIRQAYC	35
FJ376029	C	CCR5	TRGNNTRSIRIGPGQTFYATGDIIGDRQAHC	31
AF543920	C	CCR5	CTRPNNNTRKSVRIGPGQTFYATGAIIGDIREAHC	35
DQ516181	B	CCR5	CTRPNNNTRKGIHIGPGRAFYTTGAIIGNIRHAHC	35
EU575508	B	CCR5	CTRPNNNTRKSIHIGPGQAFYTTGAIIGDIRQAYC	35
JF507977	B	CCR5	CTRPNNNTKRDIPIGPGRAFYATDIVGDIRQAHC	34
JF896845	B	CCR5	CTRPNNNTRKSIGIGPGRAFYATGDIIGDIRQAHC	35
JF896873	B	CCR5	CTRPNNNTRKSIPLGPGSAFYATETIIGDIRQAHC	35
AF153130	C	CCR5	CARPNNNTRKSIRIGPGQAFYATGDIIGDIRQAHC	35
HQ644974	B	CCR5	CTRPNNNTRKSITIGPGRAFYATGNIIGDIRQAHC	35
DQ516289	B	CCR5	CTRPNNNTRKGIHIGPGRAFYTTGGIIGDIRQAHC	35
FJ376002	C	CCR5	CTRPNNNTRRVRIGPGQAFYATGDIIGDIRQAHC	34
EU293449	C	CCR5	CSRIGNNTRTSIGIGPGQAFFATGDIIGNIRKAHC	35
EU272293	B	CCR5	GTRPSNNTRKSINLGPGRAWYTTGQIIGDIRQAHC	35
EU604600	B	CCR5	CTRPSNNTRKSINVGPGRAWYATGQIIGDIRQAYC	35
EU744133	B	CCR5	CTRPNNNTRKSIHIGPGRAFYATGEIVGNIRQAHC	35
EU744080	B	CCR5	CTRYNNNTRRSIHMGPGRTFYATGDIIGDIRQAQC	35
JF896848	B	CCR5	CTRPNNNTRKSIHIAPGRAFYATENIVGDIRKAYC	35
HM246236	B	CCR5	CTRPNNNTRKSIHIGPGRAFYTTGDIIGDIRKAHC	35
EU744074	B	CCR5	CTRYNNNTRRSIPLGPGRAFYATGDIIGDIRQAQC	35
KF716498	B	CCR5	CTRPNNNTRRSIHLGPGQTLYATGDIIGDIRQAHC	35
KC312497	B	CCR5	CTRPNNNTRKGIHIGPGRTFYATGQIIGDIRQAHC	35
DQ002313	B	CCR5	CTRPNNNTRKGINIGPGRAWYTTGDIIGDIRKAHC	35
AY010784	B	CCR5	CIRPNNNTRKSIHIGPGKAFYATGDIIGDIRQAYC	35
DQ061429	B	CCR5	CTRPSNNTGKSITIGPGRAFYTTGEIIGDIRQAHC	35
EU272312	B	CCR5	YTRPNNNTRKSIPLGPGRAWYTTGQIIGDIRQAHC	35
AY228556	C	CCR5	CTRPNNNTRKSIRIGPGQTFYATNIIGDIRQAYC	34
U08784	B	CCR5	CTRPNNNTRKSIPLGPGQAWYTTGQILGDIRQAHC	35
GQ372990	B	CCR5	CLRPNNNTRKGIHIGPGRAFYTTGEIIGDIRQAHC	35
KC473830	B	CCR5	CTRPNNNTRKSIHIAPGRAFYATGAITGDIRQAHC	35
DQ110000	B	CCR5	CTRPNNNTRRSIHIAPGRAFYATGKIIGDIRQAHC	35
HM246227	B	CCR5	CTRPNNNTRKVQVGPGKALYITGSIIGDIRQAHC	34
DQ061776	B	CCR5	CTRPNNNTRKGIHIGPGRAFYATGEVIGNIRQAHC	35
HQ708091	C	CCR5	CTRPNNNTRRSYTIGPGRVLYATGSIIGDIRQAYC	35
HQ377420	B	CCR5	CTRPNNNTRKSIHIGPGSAFYATGEIIGDMRQAHC	35
FJ376004	C	CCR5	CTRPNNTKSVRIPGQTFYATGDIIGDIRQAHC	32
AF082372	B	CCR5	CTGPNNNTRRSISIGPGGAFYTTGDIIGDIRQAYC	35
AF153142	C	CCR5	CTRPNNNTRKSIRIGPGQTFYATGVIIGDIRQAHC	35
DQ061447	B	CCR5	CTRPNNNTRKSIHIGPGRAFYTTGEIIGDIRKAYC	35
JN002038	B	CCR5	CTRPNNNTRKSIHMGPGKAFYATGDIIGDIRRAHC	35
AF384255	B	CCR5	CIRPSNNTRKSIHMGPGRAWYATGSITGDIRQAHC	35
EU744132	B	CCR5	CTRPTNNTRKSIHIGPGRAFYATGDIIGDIRQAHC	35
AY585271	C	CCR5	CTRPHNNTRQSVRIGPGQAFFATREIIGDIRQAHC	35
FJ977094	C	CCR5	CARPGNNTRKSVRIGPGQAFYATGDIIGDIRQAHC	35
AF541062	B	CCR5	CVRPNNNTRRSIHIGLGRRFYTTEIVGDIRKAYC	34
AF384324	B	CCR5	CTRPNNNTRKGIHIGPGGAIYATGAIIEDIRQAHC	35
EU786673	C	CCR5	CTRPGNNTRKSMRIGPGQTFFATGDIIGDIRQAHC	35
HM246209	B	CCR5	CTRPSNNTRKSIPIGPGRAFYATGDIIGNIRQAHC	35
AY010842	B	CCR5	CTRPNNNTRKGIHIGPGSAFYATGDIIGDIRKAHC	35
DQ002115	B	CCR5	CTRPNNNTRKSITIGPGRAFYATGDIIGNIRQAHC	35
AF153136	C	CCR5	CTRPNNNTRKSIRIGPGQAFYTTGEIIGDIKQAHC	35
HQ644914	B	CCR5	CIRPNNNTRKSIHIGPGRAFYTTEIIGDIRKAYC	34
EU743981	B	CCR5	CTRPNNNTRKSIHIGPGRAFYATGEIIGDIRQAYC	35
HQ708065	C	CCR5	CIRPYNNTRKSMRIGPGQTFYATEEVIGNIRQAYC	35
AY170662	C	CCR5	CARPNNNTRKSVRIGPGQTFYATGGIIGDIRQAHC	35
FJ376014	C	CCR5	CTRPGNNTRRSVRIGPGQVFYATGEIIGDIRQAHC	35
HM179942	C	CCR5	CTRPGNNTRKGMWIGPGQAFYATGDIIGDIRQAYC	35
HQ708071	C	CCR5	CTRPNNNTRTSVRIGPGQTFFATGDVTGDIRQAFC	35
HQ644809	B	CCR5	CIRPNNNTRRSINIGPGRAIFRTGEIIGDIRQAHC	35
DQ061751	B	CCR5	CTKPNNNTRRSISLGPGRAYYATGDIIGDIRQAHC	35
HQ678272	B	CCR5	CTRPNNNTRKSIPMGPGKAFFTTDIIGEIRRAYC	34
GU204942	B	CCR5	CIRPNNNTRKSIHIAPGRAFYATGDIIGDIRQAHC	35
KF770290	C	CCR5	CIRPNNNTRRSVRIGPGQTFYATGAIIGDIRKAYC	35
AF491741	B	CCR5	CSRPNNNTRKSIPMGPGKAFYATGDIIGDIRQAHC	35

DQ002096	B	CCR5	CTRPSNNTRRGIHIGPGRAFYTTGEVIGNIRAANC	35
HM246204	B	CCR5	CRPNNNTRKSIPMGPGQALYATGEIIGDIRQAHC	34
AY010860	B	CCR5	CTRPNNNTGTSIHMGPGRAVYATGDIIGNIRQAYC	35
AF391238	C	CCR5	CPRPNHNTRRSIRIGPGQAFYATGDIIGDIRQAHC	35
DQ002308	B	CCR5	CTRPNNNTRKSINIGPGRAWYTTGAIIGDIRKAHC	35
DQ002159	B	CCR5	CTRPNNNTRKSIHMGPGRAFYATGQIIGDIRQAHC	35
HQ644910	B	CCR5	CTRPNNNTRKSIHIGPGRAFYATGEIIGDIRRAYC	35
AF391237	C	CCR5	CTRPGNNTRQSIRIGPGQTFYATGDIIGDIRQAHC	35
EF657916	B	CCR5	CIRPNNNTMKSIHMGPGRAFYATGEIIGNIRQAHC	35
GU204920	B	CCR5	CARPNNNTRKSIHIAPGRAFHTTGSIIGDIRKAYC	35
KC156328	C	CCR5	CTRPNNNIRKSMRIGPGQTFYATGGIIGDIRQAHC	35
AF153183	C	CCR5	CTRPHNNTRKSVRIGPGQTFYATGDIIGDIRQAHC	35
FJ376012	C	CCR5	CIRPNNNTRKSMRIGPGQTFYATGDIIGDIRQAYC	35
KF770328	C	CCR5	CIRPNNNTRRSMRIGPGQAYFTTGEIIGNIRQAHC	35
KF384810	B	CCR5	CTRHNNNTRKSIHIGPGSAFYATGAIIGDIRQAHC	35
HM239534	B	CCR5	CTRLNNNTRKSIHIGPGQAFATGAIIGIRQAHC	33
EU272317	B	CCR5	CTRPNNNTRKSIPLKPGRAWYTTGQIIGDIRQAHC	35
AY736822	C	CCR5	CTRPGNNTRKSVRIGPGQAFYATNDIIGDIRQAYC	35
DQ235645	C	CCR5	CIRPQNNTVKSVRIGPGQTFYTTGQVVGDIRKAHC	35
JN687737	C	CCR5	CTRPNNNTRKSIRIGPGQAFYATGDIVGDIRQAHC	35
HM239592	B	CCR5	CTRPNNNTMKSITIGPGRAFYATGSIIGDIRQAHC	35
EU578309	B	CCR5	CTRPNNNTRKSIAIGPGRAFYTGEIIGDIRQAHC	34
EU576240	B	CCR5	CTRPNNNTRRSIPMGPGKVFYTADIIGDIRQAHC	34
AF067154	C	CCR5	CVRPNNNTRESIRIGPGQTFYATGEIIGDIRQAHC	35
FJ375969	C	CCR5	CTRPGTRKSIRIGPGQSFYATGIIGDIRQAHC	32
JF508129	B	CCR5	CTRPNNNTRKGIHIGPGRTFYATGEIVGDIRQAHC	35
JF507835	B	CCR5	CMRPGNNTRRSITIGPGRAFYAGEIIGDIRKAHC	34
EU578351	B	CCR5	CTRPNNNTRKSIHMGAGRAFYTGEIIGDIRQAHC	34
KC156335	C	CCR5	CNRPHNNTRKSMRIGPGQTFYATGDVTGDIRKAHC	35
EU744041	B	CCR5	CTRPSNNTRRGIHIGPGRAFYATGEIIGDIRQAHC	35
AF259023	B	CCR5	CTRPGNNTRKSIHIGPGRAFYTTGEIIGKIRQAHC	35
HM239635	B	CCR5	CTRPNNNTRKGIHLGPGKAFYATGDIIGNIRQAHC	35
AY887852	C	CCR5	CIRPGNNTRKSIRIGPGQTFYATGDIIGDIRQAHC	35
HM179915	C	CCR5	CTRPNNNTRKSIRIGPGQTFYATGDIIGDIRQAYR	35
JF896868	B	CCR5	CTRLNNNTRKSIFAPGRTFYAMGDIIGDIRKASC	34
HQ377470	B	CCR5	CTRPNNNTRKSIHMGPGGAVYATGAIIGNIRQAHC	35
AY887854	C	CCR5	CTRPGNNTRKSVRIGPGQTFFATGDIIGDIRQAHC	35
AY887872	C	CCR5	CTRPGNNTRRSVRIGPGQTFYATGDIIGDIRQAHC	35
JN687705	C	CCR5	CTRPNNNTRKSVRIGPGQTFYATGGIIGDIKQAHC	35
AY842816	B	CCR5	CTRPNNNTRKSIPMGPGRAMYATGDIIGDIRQAHC	35
EU576299	B	CCR5	CTRPNNNTRKSIHIGPGRTFYTTGDIIGDIRQAYC	35
AF254773	C	CCR5	CTRPNNNTRKSIRIGPGQTFYATNDIIGDIRQAYC	35
KC156240	C	CCR5	CIRPGNNTRRSVRIGPGQTFYATGDITGDIRKAHC	35
AF022266	B	CCR5	CTRPNNNTRKSISIGPGRAFYTTGEIIGNIRQAHC	35
FJ998107	B	CCR5	CTRPNNNTRRGIRIGPGRAFYATAIIGDIRQAHC	34
DQ061518	B	CCR5	CTRPNNNTRKSIHITPGRTFYATGEIIGDIRQAHC	35
EU577329	B	CCR5	CSRPNNNTRRSVHIGPGRAWYTGEIIGDIRQAHC	34
DQ061749	B	CCR5	CTRPNNNTRRSISLGPGRAYYATGDIIGDIRRAHC	35
AY842789	B	CCR5	CTRPSNNTRKSIHMGPGRVLYTTGGIIGDIRQAHC	35
DQ061531	B	CCR5	CTRPNNNTRRSIYIAPGSAFYATGEIIGDIRQAHC	35
AJ418521	B	CCR5	CTRPSNNTRKSIPIGPGRAFYTTGEIIGDIRQAHC	35
DQ002152	B	CCR5	CTRPNNNTRRNIHIGPGKAIYTTGEIIGDIRQAHC	35
DQ516124	B	CCR5	CTRPNNNTIKSIHIGPGRAFYTTGQIIGDIRQAHC	35
JF507907	B	CCR5	CTRPSNNTRQSVHIGPGRALYTTTKIIGDIRKAYC	35
AF022274	B	CCR5	CTRPNNNTRKSISIGPGRAFYATGEIIGDIRQAYC	35
KF770330	C	CCR5	CIRPNNNTRRSMRIGPGQALFTTGEIIGNIRQAHC	35
JX973084	C	CCR5	CIRPGNNMRKSVRIGPGQAFYATGDIIGDIRQAHC	35
HM239590	B	CCR5	CTRPNNNTRKSIPIGPGKAFYTTGDIIGDIRQAHC	35
EU744070	B	CCR5	CIRPNNNTRKSIHLGPGRAFYATGDIIGDIRQAQC	35
AY887853	C	CCR5	CIRPGNNTRKSVRIGPGQTFFATGDIIGDIRKAHC	35
AF199041	B	CCR5	CTRPNNNTRKSINMGQGRAWYTTGQIIGDIRQAHC	35
HQ644865	B	CCR5	CTRPGNNTRKSIPIGPGRAFYATGDIIGDIRQAHC	35
JF507846	B	CCR5	CVRPGNNTRRSITIGPGRAFYAGEIIGDIRKAHC	34
JF507941	B	CCR5	CTRPNNNTRKDIHIGPGRAFYATDIVGDIRQAHC	34
DQ110003	B	CCR5	CIRPNNNTRSIMGPGAFYATGDIIGDIRQAHC	32
JF507788	B	CCR5	CERPNNNTRKGIHIGPGRTFFATGEIIGDIRRAYC	35
EU744056	B	CCR5	CTRPNNNTRKSIHVGPGKTLYATGDIIGNIRQAHC	35
AF541018	B	CCR5	CIRPNNNTRKSIHIGPGRAFYTTGEIIGDIRQAHC	35
FJ653282	C	CCR5	CTRPNNNTRKSIRIGPGQTFFATEIVGNIREAHC	34
HM179735	C	CCR5	CTRPSNNTRKSMRIGPGQAFYATGEIIGNIREAHC	35

KC473846	B	CCR5	CTRPSNNTRRGIHIGPGQAFYTTGQIIGDIRQAYC	35
AF153149	C	CCR5	CTRPINNTRWSVRIGPGQTFYATGDIIGDIRQAHC	35
AF153174	C	CCR5	CTRPNNNTRKSVRIGPGQAFYATDGIIGDIRQAHC	35
HQ708077	C	CCR5	CTRPNNNTRTSMRIGPGQTFYATGKVTGDIRQAYC	35
JF507950	B	CCR5	CTRPNNNTAKGIHIGPGRAMYATERIVGDIRRAHC	35
HM239570	B	CCR5	CERPNNNTRKSIHIAPGRTFYATGEIIGNIRQAHC	35
AB553913	B	CCR5	CTRPNNNTRKGIHMGPGRAIYTTDIIGDIRQAHC	34
AY887863	C	CCR5	CIRPGNNTRKSIRIGPGQAFYATGSIIGDIRQAHC	35
EU744021	B	CCR5	CTRPNNNTRKGIHIGPGRAFYTTGEIVGDIRQAHC	35
HM239617	B	CCR5	CTRPNNNTRRSVHIAPGAAFYATGAIIGQIRQAHC	35
AF541063	B	CCR5	CIRPNNNTRRGIHIGLGRRFYTTEIVGDIRKAYC	34
EU575330	B	CCR5	CIRPNNNTRKRITMGPGKVYYTTGQIIGDIRQAHC	35
AF391240	C	CCR5	CTRPNNNTRKSIRIGPGQTFFATNDIIGDIRQAYC	35
FJ798403	B	CCR5	CTRPNNNTRKGISIGPGRAFYATGDIIGDIRKAHC	35
FJ977091	C	CCR5	CARPGNNTKKSVRIGPGQTFYATGDIIGDIRQAHC	35
AF541031	B	CCR5	CTRPSNNTRKSIPIGPGRAFYATGEITGDIRQAHC	35
JN687717	C	CCR5	CTRPNNNTRKSIRIGPGQSFYATGDIIGDIRQAHC	35
DQ061843	B	CCR5	CTRPNNNTRKGIHVGPGAVFYATGQIIGDIRQAYC	35
HQ644913	B	CCR5	CTRPNNNTRKSIPIGPGRAFYTTEIIGNIRQAHC	34
AY253322	C	CCR5	CTRPGNNTRKSIRIGPGQAFFATGEVIGDIRQAHC	35
AY010803	B	CCR5	YKTQQLYKKSIHMGPGRAFYTTGDIIGDIRQAHC	34
EU272301	B	CCR5	CIRPNNNTRKSIHLGPGRAWYATGEIIGNIRQAHC	35
HQ644980	B	CCR5	CTRPNNNTRRSIHIGPGRAFYATGEIIGDIRQAHC	35
DQ061420	B	CCR5	CTRPNNNTRKSITIGPGRAFYTTGEIIGNIRQAHC	35
AF082360	B	CCR5	CTRPNNNTRKSIHIGPGRAFYTTGDIVGDIRQAHC	35
FJ653231	B	CCR5	CTRPSNNTSGSIHIGPGRAFDATKTITGDIRQVHC	35
DQ002279	B	CCR5	CTRPNNDTKRSVNIVPGRAIYTTDIIGDIRQAHC	34
AF021499	B	CCR5	CTRPNNNTRKSIHIGPGGAFYTTGQIIGDIRQAYC	35
HM239552	B	CCR5	CTRPNNNTRKSIIGPGRAFYATDIIGDIRQAHC	33
HQ708031	C	CCR5	CTRPNNNTRKSTRIGPGQSFYATGDIIGNIRQAHC	35
DQ061844	B	CCR5	CTRPNNNTRKGIHMGPGAVFYATGQIIGDIRQAYC	35
DQ002237	B	CCR5	CTRPSNNTRKSIHIAPGRAFYATGEIIGDIRQAHC	35
AF541060	B	CCR5	RVRPNNNTRRSIHIGLGRRFYTTEIVGDIRKAYC	34
HQ644789	B	CCR5	CTRPNNNTRRGIHIGPGRAFYTTGEIVGDIRQAHC	35
AY124977	B	CCR5	CTRLNNNTRKSINIGPGRAFYATGDIIGDIRQAHC	35
GU204943	B	CCR5	CIRPNNNTRKSIHIPGRAFATGDIIGDIRQAHC	33
AY835447	B	CCR5	CTRHNNNTRKSINIGPGRAFYATGKIIGDIRQAHC	35
KF770377	C	CCR5	CIRPGNNTSRSIRIGPGQTFYATGRIVGDIRLAHC	35
HQ644985	B	CCR5	CTRPNNNTRKSIRIGPGSAFYATGEIIGDIRQAHC	35
EF580005	B	CCR5	CERPGNNTSKGIHIGPGRAFYATEDIIGDIRKAHC	35
DQ645382	B	CCR5	CTRPNNNTRKSIHLGPGKTLYATDIIGNIRQAHC	34
GU362881	B	CCR5	CTRPNNNTRKGIHIGPGRTLYATGEIIGDIRKAHC	35
HQ644838	B	CCR5	CTRPNNNTRKSITIGPGRAFYTTGDIIGDIRQAYC	35
AF254782	C	CCR5	CTRPNNNTRRSMRIGPGQTFYATGEIIGDIRQAYC	35
HQ644917	B	CCR5	CIRPNNNTRKSIHIGPGRALYATEIIGDIRKAYC	34
JF896834	B	CCR5	CTRLNNNTRKGIHIGPGRAFYATTDIIGDVRQAHC	35
AY010861	B	CCR5	CTRPNNNTRTSIHMGPGRALYATGDIIGNIRQAYC	35
JF507905	B	CCR5	CTRPSNNTRQSVHIGPGRALYTTNIIGDIRKAYC	34
JF896832	B	CCR5	CTRPNNNTRKSIPIGPGRAFYATGHIIGNIRQAHC	35
AF153184	C	CCR5	CTRPNNNTRKSIRIGPGQAFYATNAIIGNIRQAHC	35
EU576418	B	CCR5	CTRPNNNTRKSIPMGPGKAFYARGDITGDIRKAYC	35
AY010785	B	CCR5	CIRPNNNTRKSIHMGPGKAFYATGDMIGDIRQAYC	35
JF507966	B	CCR5	CTRPNNNTRKDIHIGPGRAMYATGEIVGDIRRAHC	35
FJ375986	C	CCR5	CTRPNNNTRTSMRIGGQTFYATGDIIGDIRQAHC	34
AF384265	B	CCR5	CTRPNNNTRKSINIGPGRAFFATGDIIGDIRQAHC	35
JF507870	B	CCR5	CTRPGNNTRRSINIGPGRAFYTGEIIGNIRQAHC	34
DQ177201	B	CCR5	CTRPNNNTRKSIPMGPGKAFYATGEIIGDIRQAHC	35
DQ002272	B	CCR5	CTRPNINTKRSVNIVPGRAIYTTDIIGDIRQAHC	34
EU743975	B	CCR5	CTRPNNNTRKGIHIGPGKAFYATGEIIGDIRQAHC	35
DQ061803	B	CCR5	CIRPNNNTRKGIHIGPGRAFYATGATIGNIRQARC	35
EU744174	B	CCR5	CTRPNNNTRRSIHMGPGGALYTTGAIIGNIRQAHC	35
DQ869021	B	CCR5	CTRPGNNTRRSIPMGPGRAWYAIGEITGNIRKAHC	35
HM368242	B	CCR5	CTRPNNNTRRGIHIGPGGAFYSTGDIIGNIRQAYC	35
KF770393	C	CCR5	CTRPSNNTRRSVRIGPGQAFYTTGEIIGDIRQAHC	35
GU362883	B	CCR5	CTRPNNNTRRSIHIAPGRAFYATGEIIGDIRQAHC	35
HQ644799	B	CCR5	CTRPNNNTRKSINIGPGRAIYTTGEIIGDIRQAHC	35
JF507918	B	CCR5	CIRPNNNTRKDIHIGPGRAFYATGIVGDIRQAHC	34
FJ376022	C	CCR5	CTRPGNNTKSFRIGPGQTFFATGEIIGNIRQAHC	34
AY713408	B	CCR5	CTRPNNNTRKSIQLGPGRAWYTTGQIIGDIRQAHC	35
JX508874	C	CCR5	CTRPNNNTRRSIRIGPGQTFYATGEIIGNIREAYC	35

KC312596	B	CCR5	CVRPNNNTRKSIHLGPGSAFYATGEIIGNIRQAHC	35
EU744071	B	CCR5	CTRPHNNTRKSIHLGPGRAFYATGDVIGNIRQAHC	35
DQ002168	B	CCR5	CTRPNNNTRKSIHMGPGRALYATGAIIGDIRQAHC	35
EU576838	B	CCR5	CTRPNNNTRRDITIGPGRVFYTGEIIGDIRQAHC	34
AF384334	B	CCR5	CTRPNNNTRKSIPLGAGRAWYATGDIIGDIRQAHC	35
GU945319	C	CCR5	CTRPNNNTRQSIRIGPGQVFYATGDIIGDIRQAYC	35
DQ177199	B	CCR5	CIRPNNNTRRSIPIGPGRAFYATGGIIGDIRKAYC	35
EU744138	B	CCR5	CTRPTNNTRKSIHIGPGRAFYATGDIVGNIRQAHC	35
KC312394	B	CCR5	CTRPNNNTRKSIHIGPGSAFYTTGAIIGDIRQAHC	35
HQ708029	C	CCR5	CTRPNNNTRKSTRIGPGQSFYATGDIIGEIRQAHC	35
HM239559	B	CCR5	CTRPSNNTRKGIHMGPGKAFYATGEIRGDIRQAHC	35
AF153153	C	CCR5	CPRPINNTRWSVRIGPGQTFYATGDIIGDIRQAHC	35
HQ708030	C	CCR5	CTRPNNNTRKSTRIGPGQSFYATGEIIGNIRQAHC	35
JF507730	B	CCR5	CIRPNNNTRKSIHIGPGRTFFATGEIIGDIRRAHC	35
AF355728	B	CCR5	CTRPSNNTRKSIPLGPGRAFYATGDIIGNIRKAHC	35
EF688453	B	CCR5	CTRPNNNTRKGIHIGPGRAWYTTGEIIGDIRQAHC	35
HQ645012	B	CCR5	CTRPNNNTRKSINLGPGRALYTTGNIIGNIRQAHC	35
AF259012	B	CCR5	CTRPSNNTRKSIHIGPGRAFYTTGEVTGDIRQAHC	35
AJ418517	B	CCR5	CTRPNNNTRKSIHIGPGRAFYTTGDIIGNIRQAHC	35
HM179948	C	CCR5	CTRPGNNTRESMWIGPGQAFYATGDIIGDIRQAYC	35
EU744017	B	CCR5	CTRPNNNTRKSIHMGPGRAFYTTGEIIGDIRQAHC	35
HM239562	B	CCR5	CIRPNNNTRKGIHIGPGKAFYTTGSIIGDIRQAHC	35
HM239556	B	CCR5	CTRPSNNTRKGIHMAVGRAFYATGQIIGDIRQAYC	35
JN687824	B	CCR5	CTRPNNNTRKSINMEPGRAWYATGEIIGNIRQAHC	35
DQ235625	C	CCR5	CTRPNNNTRRSMRIGPGQAFYATGDIIGDIRQAHC	35
AY887881	C	CCR5	CVRPNNNTRKSIRIGPGQTFYATGNIIRDIRQAHC	35
EU272251	B	CCR5	CIRPNNNTRKSIPLRPGKAWYTTGQIIGDIRQAHC	35
FJ375988	C	CCR5	CTRPNNNTRQSIRIGPGQAFYATQGIIGDIRKAYC	35
KC473842	B	CCR5	CTRPGNNTRKSISLGPGRVFYATGDIIGDIRQAYC	35
AX455929	C	CCR5	CTRPGNNTRKSVRIGPGQAFYATGDIIGDIRQAHC	35
KF770319	C	CCR5	CTRPSNNTRKSIRIGPGQTFFATDDIIGDIRKAYC	35
AF541013	B	CCR5	CTRPNNNTRKSIPIGPGRALYATGDIIGDIRKAHC	35
EU744137	B	CCR5	CTRPTNNTRKSIHLGPGRAFYATGDIVGNIRQAHC	35
DQ061810	B	CCR5	CIRPNNNTRKGIHIGPGRAFYATGAIIGNIRQAHC	35
HQ377468	B	CCR5	CTRPNNNTRKGIHIGPGGAVYATGAIIGNRRQAHC	35
AF384249	B	CCR5	CTRPNNNTRKSIHMGPGKAFYTTGNIIGDIRKAHC	35
EF657926	B	CCR5	CIRPNNNTRKSIHMGPGRAFYTTGEIIGNIRQAHC	35
HM215428	B	CCR5	CTRPSNNTRKSIHLGQGRAWYTTGKIIGDIRQAHC	35
AF310131	B	CCR5	CTRPNNYTRKHIHLGARKAFYTGEIIGDIRQAHC	34
EF688447	B	CCR5	CTRPNNNTRKSIPIGPGRAFFTTGDIIGDIRQAHC	35
HQ644820	B	CCR5	CKRPNNNTRRSIHIGPGRAFFATGDIIGDIRQAHC	35
GU455519	B	CCR5	CTRPNNNTKKSISIGPGRAFYTTGQIIGDIRQAHC	35
EU576991	B	CCR5	CTRPNNNTRKSIPIGPGRAFYATGEIIGNIRQAHC	35
EU578539	B	CCR5	CTRPNNNTRRSIHLGPGAAFITGEIIGDIRQAYC	34
AY510063	C	CCR5	CTRPSNNTRKSVRIGPGQTFYATGIIIGDIRQARC	35
HM239550	B	CCR5	CTRSNNTRKSIHIGPGRAFYATGEIIGNIRQAHC	34
KC473828	B	CCR5	CTRPNNNTRKSIHIAPGGAFFATGDIIGDIRKAHC	35
DQ869020	B	CCR5	CTRPSNNTRKSITIGPGRAFYATGDIIGDIRQAHC	35
AF541065	B	CCR5	CIRPNNNTKRSIHIGLGRRFYTTEIVGDIRKAYC	34
AF384318	B	CCR5	CTRPNNNTRKGIHIGPGGAFYATGAITGDIRQAHC	35
DQ002255	B	CCR5	CTRPNNNTRKSIPIGPGRAFYTTGEIIGNIRQAHC	35
JN687762	B	CCR5	CTRPNNNTRKSIHMGPGRAFYTTGDIIGDIRQANC	35
EF657930	B	CCR5	CIRPNNNTRKSIPMGPGRAFYATGGIIGDIRQAHC	35
AY173952	B	CCR5	CTRPSNNTRKSIHIGPGRAFYTTGNIIGDIRQAHC	35
EU576936	B	CCR5	CTRPGNNTRKSIPIGPGRAFYTTGDIIGDIRKAHC	35
HQ377459	B	CCR5	CTRPNNNTRKGIHIGPGGAVYATGAIIGNIRQAHC	35
AF384278	B	CCR5	CIRPNNNTRTSMPMGPGRAFYAMGDIIGDIRQAHC	35
DQ061506	B	CCR5	CTRPNNNTRKSITIGPGRAFYTTGEIIGEIRQAHC	35
AJ418530	B	CCR5	CTRPSNNTRKSIPIGPGRAFYTTGEIIGDIRKAHC	35
U16217	B	CCR5	CTRPNNNTRKGIHMGCGRTFYATGEIIGDIRQAHC	35
FJ375977	C	CCR5	CTRPNNNTRKSVRIGPGQTFYATGDIIGREAHC	33
EF657911	B	CCR5	CIRPNNNTKKSIPMGPGRAFYATGDIIGNIRQAHC	35
DQ061807	B	CCR5	CIRPNNNTRKGIHIGLGRAFYATGEIIGNIRQAHC	35
HM246202	B	CCR5	CRPNNNTRKGIHMGFGKTLYATGEIVGNIRQAHC	34
HQ678289	B	CCR5	CTRPNNNTRKSIPMGPGRAFYTTGQIIGDIRKAYC	35
AF180902	B	CCR5	CTRPNNNTRRSIHIGPGRALYTTGQIIGDIRQAYC	35
FJ376016	C	CCR5	CTRPSNNTRTSIRIGPGQAFYATGAITGDIRQAHC	35
JN687726	C	CCR5	CTRPGNNTRKSTRIGPGQAFFTAEIIGDIRQAHC	34
DQ904348	C	CCR5	CIRPNNNTRKSIRIGPGQTFYATNAIIGDIRQAHC	35
FJ653132	B	CCR5	CTRFNNNTRKSVHIGPGRAFYATGDIIGNIRQASC	35

JF508037	B	CCR5	CARPNNNTRKGIHIGPGRAFYTTGEIIGDIRQAHC	35
KC312499	B	CCR5	CTRPNNNTRKGIHIGPGKTFYATGQIIGDIRQAHC	35
AY835449	B	CCR5	CTRPNNNTRKSIHIAPGRAFYATGEIIGDIRKAYC	35
AY887892	C	CCR5	CIRPGNNTRKSVRIGPGQAFYATGEIIGDIRQAHC	35
EU578432	B	CCR5	CTRPNNNTRKSIHLGPGSVFYTGEIIGDIRQAHC	34
DQ002100	B	CCR5	CTRPNNNTRRGIHIGPGRAFYTTGEVIGNIRAANC	35
HQ644837	B	CCR5	CTRPNNNTRRSITIGPGRAFYTTGDIIGDIRQAYC	35
HQ644998	B	CCR5	CIRPNNNTRKSIPIGPGRAFYATGDIIGDIRQAHC	35
EU744136	B	CCR5	CTRPNNNTRKGIHIGPGRAFYATGDIIGNIRQAHC	35
HM239585	B	CCR5	CTRPNNNTRTSIHMGPGKAFYTTGDIIGDIRKAYC	35
AF384283	B	CCR5	CTRPNNNTRKSIHIGPGRAFYTTGQIVGDVRKAHC	35
FJ375998	C	CCR5	CRPNNTRKMRIGPGQTYATGDIIGIRAHC	29
HQ678251	B	CCR5	CTRPNNNTRKRINLGPGKTFYATGEIIGDIRQAHC	35
AY835448	B	CCR5	CTRPNNNTSKSIHMGPGGAFFATGRIIGDIRKAYC	35
L48068	C	CCR5	CIRPGNNTRKSVRIGPGQTFFATGEIIGKIREAHC	35
AF082361	B	CCR5	CIRPNNNTRKSIHIGPGRAFYAAGDIIGDIRQAHC	35
FJ376031	C	CCR5	CTRGNNTRKSIRIGPGQTLYATGEIIGDIRAHC	33
HQ708081	C	CCR5	CTRPNNNTRRSYTIGPGRAFYATGAIIGDIRQAYC	35
AY887859	C	CCR5	CTRPGNNTRQSIRIGPGQTFYATGEVIGDIRQAHC	35
AF194975	B	CCR5	CTRPNNNTRRSINIGPGRAFYATGDIIGDIRQAHC	35
HM239612	B	CCR5	CTRPNNNTRKSIPIGPGRAYVATGDIIGDIRKAHC	35
AF541075	B	CCR5	CVRPSNNTRRGIHIGLGRRFYTTEIVGDIRKAYC	34
JF896854	B	CCR5	CTRPNNNTRKSIHMGPGAFYATGDIIGDIRQAHC	34
FJ376030	C	CCR5	CTRPYNNTRRSVRIGPGQAFYTTGEVIGDIRQAYC	35
AY123278	C	CCR5	CVRPHNNTRKSIRIGPGQAFYATESIIGDIRQAHC	35
KC473832	B	CCR5	CTRPGNNTRKSISIGPGRAFYATGDIIGDIRQAHC	35
HM239545	B	CCR5	CTRPSNNTRGIHMAVGRAFYATGQIIGNIRQAHC	34
AY426110	B	CCR5	CTRPNNNTRKSIHIGPGRALYTTGEIIGDIRQAHC	35
EU744139	B	CCR5	CTRPTNNTRKSIHIGPGRAFYATGDIIGNIRQAHC	35
DQ061740	B	CCR5	CTRPNNNTRRSISLGPGRAYYATGDIIGDIRQARC	35
JF507922	B	CCR5	CTRPNNNTRKSISIGPGRAFYATGGIIGDIRQAHC	35
AF541007	B	CCR5	CTRPSNNTRKSIPMGPGRAFYTTGEIIGNIRQAHC	35
JF896839	B	CCR5	CTRLSNNTRKGIHIGPGRAFYTTGEIIGDIRQAHC	35
EU578625	B	CCR5	CTRPNNNTRKSVHIGPGKVFYTGEIIGDIRQAHC	34
U79720	B	CCR5	CTRPNNNTRKSVSLGPGSAWYATGDIIGDIRQAHC	35
JF507748	B	CCR5	CTRPNNNTRKSIHIGPGRTFFATGEIIGDIRRAYC	35
HM239599	B	CCR5	CTRPSNNTRKGIHAWGRAFYATGQIIGDIRQAHC	34
AF153187	C	CCR5	CTRPGNNTRKSIRIGPGQTFYATNDIIGDIRSAHC	35
U04909	B	CCR5	CTRPNNNTRKSIPMGPGKAMYATGEIIGDIRKAYC	35
AJ418485	B	CCR5	CTRPNNNTRKGIHIGPGKALYATGDIIGKIRQAHC	35
HQ644856	B	CCR5	CTRPGNNTRKSIHIGPGRAFYTTGDIIGDIRQAHC	35
EF579975	B	CCR5	CGRPGNNTSKGIHMGPGRAFYATENIIGDIRKAHC	35
U08700	B	CCR5	CTRPNNNTRKSIHMGWGRAFFSTGELIGNIRQAHC	35
AY170661	C	CCR5	CTRPSNNTRKSIRIGPGQAFYATNEIIGDIRQAHC	35
HM239520	B	CCR5	CSRPNNNTRKSIPIGPGRAFYATGDIIGDIRQAHC	35
DQ002173	B	CCR5	CTRPNNNTRRSITIGPGRAFYGTDITGDIRQAHC	34
FJ376020	C	CCR5	CTRNNNTRKSIRIGPGQTFYATGDIIGNIRQAHC	34
EF688445	B	CCR5	CTRPNNNTSKGIHMGPGKAFYATGKIIGDIRQAHC	35
EU272322	B	CCR5	CTRPNNNTRKSIPLGPGRAWYTTGQIIGDIRQAHC	35
AF384253	B	CCR5	CTRPNNNTRKSIHMGPGRAFYTTGEIIENIRQAHC	35
KC473829	B	CCR5	CIRPNNNTRKGIHIGPGRAFYATGDIIGNIRKAHC	35
HM239630	B	CCR5	CTRPNNNTRKSISIGPGRAFYATGEVIGIRQAC	33
HQ708074	C	CCR5	CTRPNNNTRTSMRIGPGQTFYATGEVTGDIRQAYC	35
DQ235616	C	CCR5	CTRPNNNTRQSIRIGPGQTFFATKGIIGDIRQAYC	35
EF688458	B	CCR5	CTRPNNNTRKGIPIGPGKAFYATGEIIGDIRQAHC	35
FJ375975	C	CCR5	CTRPNNNTRKSRIGPGQAFYATDIIGDIREAHC	33
KF770359	C	CCR5	CTRPNNNTRESIRIGPGQTYYAMGDIIGDIRQAHC	35
HQ678262	B	CCR5	CIRPNNNTRKSIPMGPGKAFFTTNIIGDIRQAHC	34
DQ382364	C	CCR5	CARPGNNTRKSVRIGPGQTFFATGDIIGDIRKAHC	35
KC312582	B	CCR5	CVRPNNNTRKSIRIGPGSAFYAAGEIIGNIRQAHC	35
U79721	B	CCR5	CTRPNNNTRRSINIGPGRALYATGEIIGDIRQAHC	35
AY010802	B	CCR5	CTRPNNNTRTSIPMGPGRALYATGDIIGNIRQAYC	35
HM239553	B	CCR5	CIRPNNNTRKGIHMGFGKTLYATGEIVGNIRQAHC	35
AY835436	B	CCR5	CTRPNNNTRKSINIGPGRAFVATGAIIGDIRQAHC	35
EF643659	B	CCR5	CTRPNNNTRKSIHIGPGRAFYATGAIIGNIRQAHC	35
FJ653221	B	CCR5	CTRPSNNTSTSIHIGPGRAFDATKTIIGDIRQAHC	35
HM368227	B	CCR5	CTRPNNNTRKSIHIAPGRTFYTTGEIIGDIRQAHC	35
DQ061809	B	CCR5	CTRPSNNTRKGIHIGPGRAFYATGEITGDIRQAHC	35
HQ644800	B	CCR5	CTRPNNNTRKSINIGPGRAFFATGEIIGDIRQAHC	35
HM239582	B	CCR5	CTRPNNNTRKSIPLGPGRAFYTTGDIIGDIRKAYC	35

AF259013	B	CCR5	CTRPSNNTRKSINIGPGRAWYTTGQITGDIRQAHC	35
HQ644919	B	CCR5	CIRPNNNTRKSIHIGPGRALYTTEIIGDIRKAYC	34
AY887862	C	CCR5	CIRPNNNTRQSIRIGPGQAFFATGDIIGDIRQAYC	35
HM179830	C	CCR5	CTRPGNNTRQSMRIGPGQTFYATGDIIGDIRQTHC	35
KC596067	B	CCR5	CTRPGNNTRRSISIGPGRAFYTMGQIIGNIREAHC	35
HQ644961	B	CCR5	CTRPNNNTRKSITIGPGRAFYATGEIIGDIRKAHC	35
KF770399	C	CCR5	CTRPGNNTRKSMRIGPGQTFYATGDIVGDIRKAHC	35
EF657929	B	CCR5	CMRPNNNTRKSIHMGPGRAFYTTGEIIGNIRQAHC	35
JN687741	B	CCR5	CTRPSNNIRKSIHMGWGRAFYATGEITGDIRQAHC	35
AF153167	C	CCR5	CVRPNNNTRRSVRIGPGQTFYATGDIIGDIRQAHC	35
EF600088	B	CCR5	CIRPNNNTRQGMHIGPGKALYTTNIIGNIRQAHC	34
HQ645004	B	CCR5	CTRPNNNTRRSITIGPGRAFYATGDIIGDIRRAQC	35
EF175209	B	CCR5	CIRPNNNTRKSIPMGPGKAFYATGDIIGNIRLAYC	35
AY426116	B	CCR5	CTRPNNNTRKSIHIGPGRAIYTTGKIIGDIRQAHC	35
EU272323	B	CCR5	CTRPNNNTRKSIPLGPGRAWYTTGIIGDIRQAHC	34
HQ644863	B	CCR5	CTRPNNNTRKSIHMGPGRAFYATEDVIGDIRQAHC	35
HM215400	B	CCR5	CIRPNNNTRKSIPLGQGRAWYTTGQIIGDIRQAHC	35
EF600084	B	CCR5	CIRPNNNSRQGIHIGPGKALYTTKIIGNIRQAHC	34
EU576214	B	CCR5	CTRPSNNTRKGIHIGPGRAFYATDIIGEIRQAHC	34
KC473825	B	CCR5	CTRPGNNTRKSIHIGPGRAFYATGDIIGDIRKAHC	35
GU455525	B	CCR5	CIRPNNNTRKGIHIGPGRAFYTTGQIIGDIRQAHC	35
AY510064	C	CCR5	CTRPNNNTRKSARIGPGQTFYAMGDIIGDIRQAHC	35
HM246225	B	CCR5	CTRPGNNTRKGIHIGPGRAFYATGDIIGDIRQAHC	35
AF199032	B	CCR5	CTRPNNNTRKSIPIGPGRAFYTTGQIIGDIRQAHC	35
EF600093	B	CCR5	CTRPNNNTRKSINIGPGRALYTTGDIIGDIRQAHC	35
FJ670521	C	CCR5	CTRPGNNTRKSMRIGPGQTFFATGDIIGNIRQAHC	35
DQ061480	B	CCR5	CIRPNNNTRKSITIGPGRAFYTTGEIIGEIRQAHC	35
DQ516122	B	CCR5	CIRPNNNTIKSIHIGPGRAFYTTGQIIGDIRQAHC	35
EU575728	B	CCR5	CMRPNNNTRKGIHIGPGGAFYATGDIIGNIRQAHC	35
KC156340	C	CCR5	CTRPHNNTRKSMRIGPGQAFYATGDVTGDIRKAHC	35
AF541073	B	CCR5	CIRPNNNTRRGIHIGLGRRFYATEIVGDIRKAYC	34
AY842811	B	CCR5	CTRPNNNTRKSIHMGPGRAMYATGDIIGDIRQAHC	35
EF657903	B	CCR5	CIRPNNNTRKSIPMGPGRAFYATGEIIGNIRQAHC	35
AJ418498	B	CCR5	CTRPSNNTRKSINIGPGRAFYTTGEIIGDIRKAHC	35
DQ516259	B	CCR5	CTRPNNNTRKGIHMGPGRALYTTGAIIGDIRQAHC	35
JF507945	B	CCR5	CTRPNNNTRRNIHIGPGRAMYATGQIIGNIRQAHC	35
AY010801	B	CCR5	YKTQQQYKTSIPMGPGRAMYATGDIIGNIRQAYC	34
AY713412	B	CCR5	CTRPNNNTRKSIHMGPGRAFYTTGDIIGDIRKAHC	35
AF112548	B	CCR5	CTRPNNNTRKGIHIGPGRTFYTTGEIIGDIRQAHC	35
AF194976	B	CCR5	CTRPNNNTRRSISIGPGRAFYATGDIIGDIRQAHC	35
U08698	B	CCR5	YKTQQQYKKKYTYGMGESIYATGEIIGNIRQAHC	34
EU744083	B	CCR5	CTRHHNNTRRSIHLGPGRAFYATGDVIGDIRQAQC	35
EF600089	B	CCR5	CIRPNNNTRQGIHIGPGRALYTTNIIGNIRQAHC	34
AY010843	B	CCR5	CTRPNNNTRKGIHIGPGSAFYATGDIIGDIRQAHC	35
JQ779253	C	CCR5	CTRPGNNTRKSIRIGPGQVFFTATDIIGNIREAHC	35
AF543911	C	CCR5	CTRPNNNTRKSIRIGPGQTFYATDAIIGNIREAHC	35
HM239571	B	CCR5	CIRPNNNTRRSIQMGPGKTFFTTGDIIGDIRRAHC	35
AY835442	B	CCR5	CTRPNNNTRKSIHIGPGRAFYATGGVIGDIRQAHC	35
JF507986	B	CCR5	CTRPNNNTRKSINIGPGRAFYTTGAIIGDIRQAHC	35
HQ644900	B	CCR5	CTRPNNNTRKSIHIGPGRAFYTTGDITGDIRKAYC	35
HQ377464	B	CCR5	CTRPNNNTRKSISMGPGRAFFATGEIIGNIRQAHC	35
DQ869031	B	CCR5	CTRPNNNTRKGIHMGPGRTFYATGEIIGDIRQAHC	35
HM246208	B	CCR5	CTRPNNNTRRSISIGPGRAFFATDIIGDIRQAHC	34
KC156272	C	CCR5	CTRPNNNTRKSMRIGPGQTFYATGAIIGDIRQAHC	35
DQ110004	B	CCR5	CRPNNNTRKSIMGPGRAFYATGDIIGDIRQAHC	33
KF766538	C	CCR5	CIRPNNRKSIRIGPGQTFYATGEIIGDIRQAHC	33
AF254771	C	CCR5	CTRPGNNTRKSVRIGPGQAFFATNDIIGDIRQAHC	35
U08454	C	CCR5	CTRPNNNTRKSIRIGPGQTFYATNEIIGNIREAHC	35
AF541078	B	CCR5	CIRPNNNTKRSIHLGLGRRFYTTEIIGDIRKAYC	34
KC156223	C	CCR5	CIRPGNNTRRSMRIGPGQTFYATGDITGDIRKAHC	35
AY010883	B	CCR5	CTRPNNNTRTSIPMGPGRAMYATGDIIGNIRHAYC	35
GU204924	B	CCR5	CIRPNNNTRKSIHIGPGRAFYTTGEIIGDIRQAYC	35
AY713417	C	CCR5	CTRPNNNTRQSIRIGPGQTFYATGEIIGDIRQAHC	35
HQ678269	B	CCR5	CTRPSNNTRKGIHMGPGRAFYATTNIAGDIRKAHC	35
JF896840	B	CCR5	CIRPGNNTRKSIQMGFGRAIYTTGDIIGIRQAHC	34
EU744040	B	CCR5	CTRPSNNTRKGIHMGPGRAFYATGEIIGDIRQAHC	35
DQ002143	B	CCR5	CTRPNNNTRRGIHIGPGRAFYTTGEIIGNIRQAYC	35
AF153190	C	CCR5	CTRPNNNTRKSIRIGPGQAFFATNEIIGDIRQAHC	35
DQ358757	C	CCR5	CTRPNNNTRKSIRIGPGRTFYATGDIIGNIRKAYC	35
DQ235618	C	CCR5	CIRTGNNTRKSVRIGPGQTFYATGAIIGDIRQAHC	35

JF896828	B	CCR5	CTRPNNNTRKSIHIAPGRAFYATGEIIGNIRAHC	34
EU744162	B	CCR5	CTRPNNNTRRSIHIGPGRAFYTSGGIIGDIRQAHC	35
EF657924	B	CCR5	CIRPNNNTRKSIHMGPGRAFYATGEIIGNIRQAHC	35
DQ235619	C	CCR5	CTRPGNNTRKSVRIGPGQTFFATGDIIGNIRLAHC	35
FJ375974	C	CCR5	CTRPNNNRKSIRIGPGQAFYATGDIIGDIRQAHC	34
EU575892	B	CCR5	CSRPNNNTRRSIHIGPGRAFYATGDITGDIRKAHC	35
HQ644908	B	CCR5	CIRPNNNTRKGIHIGPGRAFYTTGEIIGNIRQAYC	35
JF896851	B	CCR5	CIRPNNNTRKSIHIGPGRAFYATGQIIGNIRQAHC	35
GU455515	B	CCR5	CTRPNNNTRKSIPMGPGKAFYTTGEIIGDIRQAHC	35
HQ708070	C	CCR5	CIRPSNNTRTSMRIGPGQTFYATGDVTGDIRQAYC	35
DQ222214	B	CCR5	CTRPNNNTSKSIPLGPGRAFHTTGRIIGDIRQAHC	35
DQ002226	B	CCR5	CTRPSNNTRKSITIGPGRAFYATGEIIGDVRKAHW	35
FJ375971	C	CCR5	CRGNNTRKSIRIGPGQAFHATGAIIGDIRAHC	32
AY158535	C	CCR5	CIRPGNNTRQSIRIGPGQTFFATGDIIGDIRQALC	35
AY123262	C	CCR5	CTRPNNNTRSVRIGPGQAFYATKDIIGDIRQAHC	34
DQ061765	B	CCR5	CTRPSNNTRKGIHIGPGRAFYATGEIIGDIRKAHC	35
JF896867	B	CCR5	CVRPNNNTRKIHMGPGKAFATGDIIGDIRQAHC	33
EU578352	B	CCR5	CTRPNNNTRRSIHLGAGKALYTGEIIGDIRQAHC	34
EF117272	C	CCR5	CIRPNNNTRKSIRIGPGQTFYATGDIVGDIRQAYC	35
AF384303	B	CCR5	CTRPNNNTRKSIPIGPGRAVYATGQMIGDIRQAHC	35
KC473827	B	CCR5	CTRPGNNTRKSIHLGQGRAWYATGDIIGDIRQAHC	35
AF286234	C	CCR5	CTRPGNNTRKSVRIGPGQTFYTNDIIGDIRQAYC	34
HQ644797	B	CCR5	CTRPNNNTRRSINIGPGRAIYTTGEIIGDIRQAHC	35
HQ708059	C	CCR5	CTRPNNNTRKSVRIGPGQTFYATGAVTGDIRKAYC	35
EU744141	B	CCR5	CTRPNNNTRKSIHIGPGKTFYATGEIIGNIRQAHC	35
FJ376003	C	CCR5	CARPGNNTRKSRIGPGQSFHATGEIIGNIRAHC	33
HM239618	B	CCR5	CIRPGNNTRKSIHIGPGRAFYATGNIIGDIRQAHC	35
AF384269	B	CCR5	CTRPGNNTRRSIHIAPGRAFYATGDIIGDIRKAHC	35
EU744094	B	CCR5	CTRPHNNTRKSIHLGPGRAFYATGDIIGNPRQAYC	35
HQ645000	B	CCR5	CTRPNNNTRRSIRIGPGSAFYTTGDIIGDIRRAHC	35
FJ376034	C	CCR5	CTRPGNNTRSIRIGPGQTFYATIIGIRQAHC	31
DQ061758	B	CCR5	CTRPNNNTRKSISLGPGRAYYATGDIIRNIQAHC	34
AY010882	B	CCR5	CTRPNNNTRTSIPMGLGRAMYATGDIMGNIRQAYC	35
JF508116	B	CCR5	CTRPNNNTRRSIHIGPGRAFFTAGEIIGNIRQAHC	35
EF117271	C	CCR5	CARPSNNTRTSIRIGPGQTFYATGAITGDIRQAHC	35
AY835451	B	CCR5	CTRPGNNTRRSINIGPGRAFYATGAIIGDIRKAHC	35
EF657902	B	CCR5	CIRPNNNTRKSIPMGPGKAFYATGGIIGDIRQAHC	35
AY170665	C	CCR5	CTRPNNNTTTSVRIGPGQTFYATGDIIGNIRAAHC	35
FJ653219	B	CCR5	CTRPSNNTSGSIHIGPGKAFDATKTITGDIRQAHC	35
AY835446	B	CCR5	CIRPNNNTRKGIHIGPGRAFYTTGDIIGDIRQAHC	35
DQ382367	C	CCR5	CTRPSNNTRKSVRIGPGQTFFATGEIIGDIRQAHC	35
EU575786	B	CCR5	CMRPNNNTRKSINIGPGRAFYATGDIIGDIRQAHC	35
DQ061479	B	CCR5	CTGPHNNTRKSIHIGPRRAFYTTEEIIRNIRQAHC	35
KF770356	C	CCR5	CTRPNNNTSQSIRIGPGQTYYAMGRIIGDIRQAHC	35
EU272334	B	CCR5	CTRPNNNTRKSIHIGPGQAWYATGEIIGDIRQAHC	35
AF384257	B	CCR5	CIRPGNNTGKSIPMGPGRAWYATGSIIGDIRQAHC	35
FJ977095	C	CCR5	CTRPNNNTRQSIRIGPGQAFYATGDIIGDIRQAHC	35
EU272325	B	CCR5	CTRPNNNTRKSIHIGPGQAWYTTGQIIGDIRQAHC	35
U08772	B	CCR5	STRPGNNTRKGIPIGPGGSFYATERIIGDIRQAHC	35
EU744073	B	CCR5	CTRYNNNTRKSIPLGPGRAFYATGDIIGDIRQAQC	35
JF896865	B	CCR5	CTRNNNTRRSIPMGPGAFYTTIIGDIRQAHC	31
EU272269	B	CCR5	CIRPNNNTRKSIHLGLGRAWYATGEIIGNIRQAHC	35
HM368228	B	CCR5	CTRPNNNTRRSIHIAPGRTFYTTGEIIGDIRQAHC	35
DQ061527	B	CCR5	CTRPNNNTRKSIHIAPGRAFYATGEIVGDIRQAHC	35
EU578657	B	CCR5	CTRLSNNTRKGVHLGPGSAMYATGEIIGDIRQAHC	35
AY170659	C	CCR5	CIRPNNNTSKSIRIGPGQTFYATGRIIGDIRQAHC	35
AY510066	C	CCR5	CTRPGNNTRKSVRIGPGQAFYATGAIIGDIRQAHC	35
JX140669	C	CCR5	CTRHNNNTRKSIRIGPGQTFYATGDIIGDIRQAYC	35
AY887857	C	CCR5	CTRPGNNTRNSIRIGPGQTFFATGEIIGDIRQAHC	35
JN687761	B	CCR5	CIRPSNNTRKSIHMGPGRVLYATGEIIGDIRQAHC	35
JF508108	B	CCR5	CTRPNNNTRRSIHIGPGRALFTAGEIIGNIRQAHC	35
AY010884	B	CCR5	CTRPNNNTRTSIPIGPGRAIYATGDIIGNIRQAYC	35
DQ002333	B	CCR5	CTRPNNNTRKSIHIAPGSAFYATGDIIGNIRQAYC	35
AF384264	B	CCR5	CTRPNNNTRKGIHVGPGRAFYATGEIIGDIRQAHC	35
JX140658	B	CCR5	CTRPSNNTRKGISLGQGGVFYTTGDIIGNIRQAHC	35
DQ002103	B	CCR5	CTRPSNNTRKSITIGPGRAFYTTEVIGNIRAAYC	34
AY570012	B	CCR5	CTRPNNNTRKSINLGPGRALYTTGEIIGDIRQAHC	35
JF896823	B	CCR5	CTRLNNNTRQSIHMGPGRALYTTDIVGDIRAHC	33
JF507942	B	CCR5	CTRPNNNTRKDIHIGPGRAMYATGQIIGNIRQAHC	35
AY010774	B	CCR5	CLRPNNNTRKSIHMGPGKAFYATGDIIGDIRQAYC	35

DQ904338	C	CCR5	CTRPNNNTRKSMRIGPGQSFYATGEIIGDIRQAHC	35
FJ853620	B	CCR5	CIRPNNNTRKSINIGPGRAFYAAGEIIGDIRQAHC	35
KC596066	B	CCR5	CVRPNNNTRKSIHLAAGKALYATGDIIGDIRQAHC	35
DQ904349	C	CCR5	CTRPNNNTRKGIGIGPGQTFYATNAIIGDIRQAHC	35
HQ699979	B	CCR5	CTRPNNNTRKSIHLGPGQAWYTTGEIIGDIRQAHC	35
DQ516159	B	CCR5	CTRPNNNTRKDIHIGPGRAFYATGDIIEDIRQAHC	35
JN687723	C	CCR5	CTRPNNNTRKSVRIGPGQAFYATNGIVGNIRQAHC	35
DQ002059	B	CCR5	RIRPNNNTRKSIHIGPGRAFYTTGDVIGDIRQAHC	35
DQ516121	B	CCR5	CIRPNNNTIKSIHIGPGRAFYTTGQIIGNIRQAHC	35
HM246196	B	CCR5	CTRPNNNTRKSIHIAPGRTFYATGDIIGDIRQAHC	35
HQ644787	B	CCR5	CTRPNNNTRRSIHIGPGRALYTTGEIIGDIRQAYC	35
HQ644903	B	CCR5	CTRPGNNTRKSIHIGPGRAFYTTGDITGDIRKAHC	35
JX140665	C	CCR5	CIRPGNNTRKSVRIGPGQAFYATGEIIGDIRKAHC	35
HQ708022	C	CCR5	CTRPNNNTRKSTRIGPGQTFYATGGIIGDIRQAHC	35
AF259047	B	CCR5	CTRPGNNTRKGIHIGPGRAFYTTGQIIGDIRQAHC	35
HM239564	B	CCR5	CTRPNNNTRKSINIGPGRAFYATGAIIGDIRQAHC	35
AY158534	C	CCR5	CTRPHNNTRKSVRIGPGQAFYATGDIIGNIRQAHC	35
DQ002178	B	CCR5	CTRPNNNTRRSITIGPGRAFYGTDIIGDIRQAHC	34
JN002060	B	CCR5	CTRPNNNTRKSIPIGPGRAIYTTGGIIGDMRQAHC	35
AF153175	C	CCR5	CTRPNNNTRKSVRIGPGQAFYATNEIIGDIRQAHC	35
FJ653109	B	CCR5	CTRPNNNTRKSINIGPGGAFYAATDIIGDIRQAHC	35
HM239565	B	CCR5	CTRPNNNTRKGVHIGPGRTFFYTGDIIGDIRQAHC	35
HQ644834	B	CCR5	CTRPNNNTRRSINIGPGRAIYTTGQIIGDIRQAHC	35
EF600087	B	CCR5	CIRPNNNTRQGIHIGPGKALYTTNIIGNIRQAHC	34
DQ904337	C	CCR5	CTRPNNNTRKSIRIGPGQTFYATGAIIGNIREAHC	35
JF507850	B	CCR5	CTRPGNNTRRSINIGPGRAFYTGEIVGDIRQAHC	34
AY835452	B	CCR5	CTRPNNNTSKSITIGPGRAFYATGRIIGDIRKAHC	35
AY887870	C	CCR5	CIRPGNNTRKSVRIGPGQAFYATGDIIGDIRKAHC	35
DQ002066	B	CCR5	CIRPNNNTRKSIHIGPGRVFYTTGDIIGDIRQAHC	35
HM239575	B	CCR5	CTRPNNNTRKSIPMGPGKAWYATGEIIGDIRQAYC	35
HM179917	C	CCR5	CTRPNNNTRKSIRIGPGQTFYATGDIMGDIRQAYC	35
DQ002326	B	CCR5	CTRPSNNTRKSIHMGPGRAFYTTGEIIGDIRQTHC	35
HQ678274	B	CCR5	CTRPNNNTRKGIHIGPGRVFYATGEIIGDIRQAHC	35
HQ644864	B	CCR5	CTRPGNNTRKSIHMGPGRAFYVTEDVIGDIRQAHC	35
DQ235615	C	CCR5	CIRPNNNTRQSVRIGPGQTFFANDIIGDIRQAHC	34
HM246240	B	CCR5	CTRPNNNTRKGVHIGPGRAFYATGIIGDIRKAYC	34
AY887885	C	CCR5	CTRPGNNTRKSVRIGPGATFYATGDIIGDIRQAHC	35
EU575529	B	CCR5	CTRPNNNTRKGIHIGLGRALYATGDIIGDIRQAHC	35
DQ061416	B	CCR5	CTRPNNNIRKSINIGPERAFYTTGEIIGDIRQAHC	35
AY887861	C	CCR5	CTRPGNNTRKSIRIGPGQAFYATGDIIGDIRKAHC	35
EU578397	B	CCR5	CTRPNNNTRRSIHMGAGKALYTGEIIGDIRQAHC	34
EU578272	B	CCR5	CTRPNNNTRKSIHMGWGRAFYATGQIIGDIRQAHC	35
EF688454	B	CCR5	CTRPNNNTRRSIHIGPGRAFHATGEIVGNIRQAHC	35
FJ653100	B	CCR5	CTRPNNNTRKSINIGPGRAFYAATGIIGDIRQAHC	35
DQ061684	B	CCR5	CIRPNNNTRKGIHLGPGGAFYATGGIIGGIRQAYC	35
AY736823	C	CCR5	CTRPNNNTRRSIRIGPGQTFYATGEIIGDIRQAHC	35
AJ418514	B	CCR5	CTRPNNNTRKSIHIGPGRAFYTTGGIIGNIRQAHC	35
DQ382365	C	CCR5	CTRPGNNTRKSVRFGPGQAFYATGDIIGDIRQAHC	35
JQ779237	C	CCR5	CTRPNNNTRKSIRIGPGQAFFATTGIIGNIRQAYC	35
FJ376035	C	CCR5	CIRPNNNTKSRIGPGQFATDIIGNIRQAHC	30
AY170666	C	CCR5	CARPNNNTRTSVRIGPGQAFYATNDIIGKIRQAHC	35
AF153165	C	CCR5	CIRPGNNTRKGMRIGPGQTFYATGDIIGDIRQAHC	35
DQ382363	C	CCR5	CTRPNNNTRKSVRIGPGQTFYATGEIIGNIRQAHC	35
DQ061805	B	CCR5	CTRPSNNTRKGIHIGPGRAFYATGAIIGNIRQAHC	35
KF384807	B	CCR5	CTRPSNNTIKGIHMGPGRAFYATEQVIGDIRQAHC	35
AY505002	C	CCR5	CTRPGNNPRKSVRIGPGQAFYATGDIIGDIRQAYC	35
DQ002211	B	CCR5	CTRPNNNTRKSIHIGPGRAFYTTGEIIGDIRQAYC	35
AY010857	B	CCR5	CTRPNNNTGTSIPMGPGRAVYATGDIIGNIRHAYC	35
JF507914	B	CCR5	CTRPNNNTRKSISIGPGRAFYATGQIIGDIRQAHC	35
DQ358770	C	CCR5	CTRPNNNTRKSIRIGPGQTFYATRGIIGDIREAHC	35
HQ644888	B	CCR5	CTRPNNNTRKGIHIGPGRAFYTTGQIIGNIRLAHC	35
KF770311	C	CCR5	CTRPDNNTRRSVRIGPGQVFYTNDIIGDIRQAYC	34
AF153188	C	CCR5	CTRPGNNTRTSIRIGPGQTFFANNIIGDIRQAHC	34
DQ516305	B	CCR5	CTGPNNNTRKSIHIGPGRAFYTTGGIIGDIRQAHC	35
FJ376025	C	CCR5	CTRPGNNKRKSMRIGPGQTFYATGDVIGDIRKAQC	35
AF384321	B	CCR5	CTRPNNNTRKGIHIGPGGAIYATGAITGDIRQAHC	35
DQ002311	B	CCR5	CTRPNNITRKSINIGPGRAWYTTGAIIGDIRKAHC	35
AY842837	B	CCR5	CIRPNNYTRKSINIGPGRAMYATEQITGDIRQAHC	35
HM239603	B	CCR5	CTRPNNNTRKGVQVGPGKALYITGSIIGDIRQAHC	35
DQ235639	C	CCR5	CIRPNNNTRKSIRIGPGQAFYATNDIIGDIRQAHC	35

AY835443	B	CCR5	CERPNNNTIKSIHLGPGRAWHATGQIIGDIRKAFC	35
AF384289	B	CCR5	CTRPNNNTRKSIIGPGRAFYATGDIIGDIRQAHC	34
EU744063	B	CCR5	CTRPHNNTRRSIHLGPGKTFYATGDIIGNIRQAHC	35
EU272196	B	CCR5	YKTPQQSRKSINLGPGRAWYTTGQIIGDIRQAHC	34
EF688428	B	CCR5	CTRPNNNTRKGIHIGPGRAFYTTGEIIGNIRQAHC	35
AF384293	B	CCR5	YTRPNNNTRKSIPIGPGRAFYARGDIIGDIRQAHC	35
DQ061821	B	CCR5	CTRPNNNTRKGIHMGPGAVFYATGQIIGDIRKAHC	35
AF153150	C	CCR5	CTRPGNNTRKSVRIGPGQAFYATGEIIGDIRQAHC	35
DQ177200	B	CCR5	CTRPNNNTRKGIHMGPGRAYYATGDIIGNIRQAHC	35
DQ002101	B	CCR5	CTRPSNNTRRGIHIGPGKAFYTTGGVIGDIRKANC	35
EU293446	C	CCR5	CIRPNNNTRKSIRIGPGQSFHATGEIIGNIRQAHC	35
HM239627	B	CCR5	CTRPNNNTRKIHIGPGRAFYATGDIGDIRQAHC	33
AF384245	B	CCR5	CTRPNNNTRKSIHIAPGRAYYATGDIIGDIRQAHC	35
DQ061750	B	CCR5	CTRPNNNTRRSISLGPGRAYYATGDIIGNIRQAHC	35
JF896849	B	CCR5	CTRPNNTRKSIPMGPGKVFYATEIIGDIRQAHC	33
KF384813	B	CCR5	CTRPNNNTRKSISIGPGRAFYTTGEVIGDIRQAHC	35
FJ376027	C	CCR5	CTRPNNNTRKSVRIGPGQTFYATGDIIGDRQAYC	34
EU578380	B	CCR5	CTRPGNNTRKGITIGPGSVFYTGEIIGDIRQAHC	34
AF180901	B	CCR5	CTRPNNNTRKSIPIGPGRAFYATGQIIGDIRQAYC	35
DQ061431	B	CCR5	CTRPNNNTRKSIHIGPGRAFYTAGEIIGDIRQAHC	35
AF254775	C	CCR5	CTRPNNNTRKSVRIGPGQTFYATGEIIGNIREAHC	35
DQ002194	B	CCR5	CTRPGNNTRKSIRIGPGRAFYATDIIGDIRQAYC	34
HM179921	C	CCR5	CTRPNNNTRKSIRIGPGQAFYATGDIIGDIRQAYC	35
JF507899	B	CCR5	CTRPNNNTRQSVHIGPGRALYTTNIIGDIRKAYC	34
AY887871	C	CCR5	CTRPANNTRRSIRIGPGQTFYATGEIIGDIRQAHC	35
JN687759	B	CCR5	CTRPNNNTRRSIPMGPGRMFTTKIVGDIRQAHC	33
HQ644835	B	CCR5	CTRPNNNTRRSITIGPGRAFYTTGDIIGDIRQAHC	35
JN002057	B	CCR5	CTRPDNNTRKGIHLGPGGTFFATGAKIGDIRQAHC	35
AF384323	B	CCR5	CTRPNNNTRKGIHTGPGGAIYATGAIIGDIRQAHC	35
EU272266	B	CCR5	CTRPNNNTRKSINLGPGRAWYATGQIIGDIRRAHC	35
HM239633	B	CCR5	CIRPNNNTIKSIPIGPGRAFYATGKIVGDIRKAYC	35
AM156921	B	CCR5	CIRPNNNTRRSIHIGPGRAFYTTDIIGNIRQAHC	34
AF541009	B	CCR5	CTRPSNNTRKSIPIGPGRAFYATGEITGDIRKAHC	35
EF657925	B	CCR5	CMRPNNNTRKSIPMGPGRAFYATGEVIGNIRQAHC	35
EU786679	B	CCR5	CTRPNNNTRRSITIGPGRAFYATGEIIGDIRKAYC	35
AF355729	B	CCR5	CTRPCNNTRKSIPLGPGRAFYATGDIIGNIRKAHC	35
HQ644791	B	CCR5	CTRPNNNTRRSIHIGPGRAFYTTGEIIGDIRQAHC	35
DQ061400	B	CCR5	CTGPNNNTRKSIHIGPGRAFYTTGEIIGDIRQAHC	35
HM246237	B	CCR5	CTRPNNNTRKSINLGPGRTIYATGDIIGDIRQAHC	35
EU786678	B	CCR5	CIRPNNNTRKSINIGPGRAFYTTGAITGDIRQAHC	35
JF896817	B	CCR5	CIRPNNNTRKSIHMGPGRAYYTTDIIGDIRKAYC	34
AF384252	B	CCR5	CTRPNNNTRKSIHMGPGRAFYTTGEIIGNIRQAHC	35
U08714	B	CCR5	CTRPSNNTRKGIHIGPGRAFYATGDIIGDIRQAHC	35
AF199033	B	CCR5	CTRPSNNTKSIHIGPGRAFYTTGEITGDIRQAHC	34
EU744118	B	CCR5	CTRPNNNTRKSINIGPGRAIYTTGEIIGNIRQAHC	35
AF082384	B	CCR5	CTRLNNYTKEVVSMGPGRAFFTTGDIIGAIRRAHC	35
DQ061523	B	CCR5	CTRPNNNTRKSIPIGPGRAFYATGETIGDIRQAHC	35
AF384254	B	CCR5	CTRPSNNTRKSIHMGPGRAWYATGSITGDIRQAHC	35
EF688437	B	CCR5	CTRPNNNTRKSIYIGPGRAFHTTGRIIGDIRQAHC	35
AF153154	C	CCR5	CTRPNNNTRKSIRIGPGQAFYATGDIIGDIRQAHC	35
EF688448	B	CCR5	CTRPNNNTRKSIPIGPGRAFYTTGDIIGNIRQAHC	35
GU945314	C	CCR5	CTRPGNNTRKSIRIGPGQTFYATGGIIGDIRQAHC	35
AF153132	C	CCR5	CTRPGNNTRKSVRIGPGQTFYATDDIIGDIRKAHC	35
AF153179	C	CCR5	CTRPNNNTRQSMRIGPGQTFYATGDIIGNIRQAHC	35
DQ061398	B	CCR5	CTRPNNNTRKSIHIGPGSAFYTTGEIIGNIRQAHC	35
DQ002301	B	CCR5	CTRPSNNTRKGIHIGPGRAFYATGDIIGDIRKAHC	35
JF508035	B	CCR5	CTRPNNNTRKGIHIGPGRAFYTTGEIIGNIRQASC	35
KF770342	C	CCR5	CTRPGNNTRRSIRIGPGQSFYATGGIIGDIRQAHC	35
AY170657	C	CCR5	CTRPSNNTRKSIRVGPGQSFHATGEIIGDIRQAHC	35
AY010894	B	CCR5	CIRPNNNTRKSIPMGPGKAFYATGDIIGDMRDACW	35
AY842786	B	CCR5	CTRPNNNTRKSIHMGPGRVLYTTGGITGDIRQAHC	35
AF199040	B	CCR5	CTRPNNNTRKSINMGPGRAWYTTGQIIGDIRQAHC	35
FJ977083	C	CCR5	CIRPGNNTRKSIRIGPGQTFFATGDIIGDIRRAHC	35
DQ177191	B	CCR5	CTRPNNNTRKSIHIAPGRAFHATGDIIGNIRQAHC	35
DQ002302	B	CCR5	CTRPNNNTRKGIHIGPGKAFYATGDIIGDIRKAHC	35
AF355732	B	CCR5	CTRPNNNTRKSIHLGPGRAFYATGDIIGNIRKAHC	35
KF384811	B	CCR5	CERPNNNTRRSIPIGPGRVFFTSEIIGDIRQAYC	34
JF896820	B	CCR5	CTRPNNNTRKSIHIAPGRSFYATGDIIGDIRQAHC	35
FJ375978	C	CCR5	CTRPNNNTRKSVRIGPGQTFYAGGIIGDIRQAYC	34
HM239546	B	CCR5	CTRPNNNTRKSIHITPGRAFYATGDIIGDIRQAHC	35

FJ375967	C	CCR5	CTPNNNTSVRIGPGQTFYATNDIIGNIRQAC	31
DQ235647	C	CCR5	CTRPNNNTRQGIGIGPGQTFYAHTNIIGDIRQAHC	35
HQ644794	B	CCR5	CTRPNNNTRRSIHIGPGSAFYTGEIIGNIRQAHC	34
HQ678273	B	CCR5	CTRPSNNTSKSIPIGPGRAFYATGRIIGDIRQAHC	35
U45485	C	CCR5	CTRPSNNTRKSMRIGPGQTFYATGDIIGDIRQAHC	35
DQ235638	C	CCR5	CTRPGNNTRKSIRIGPGQTFYANDIIGDIRQAYC	34
DQ061439	B	CCR5	CTRPSNNTRKSIHIGPGRAFYTTGEVIGDIRQAHC	35
HQ644925	B	CCR5	CVRPNNNTRKSIPIGPGRAFYTTGDIIGDIRQAHC	35
HM239637	B	CCR5	CTRPNNNTRKSISIGPGRAFFATGDIIGDIRQAHC	35
DQ061802	B	CCR5	CIRPNNNTRKGIHIGPGRAFYATGAIIGNIIQAHC	35
DQ516111	B	CCR5	CTRPNNNTVKSIHIGPGRAFYTTGQIIGNIRQAHC	35
HM246221	B	CCR5	CTRPNNNTRKSIIGPGRAYFATGDIIGDIRKAHC	34
AY529663	C	CCR5	CPRPNNNTRKSIRIGPGQTFYATNDIIGDIRQAHC	35
AF310108	B	CCR5	CTRPNNNTRKSIHIEAGKALYTGEIIGDIRQAHC	34
DQ061436	B	CCR5	CARPNNNTRKSIHIGPGRAFYTTGEIIGDIRQAHC	35
AY713410	B	CCR5	CTRPNNNTRKGIHMGPGRAFYATGTIIGDIRQAHC	35
U08671	B	CCR5	CTRPGNNTRKGIPIGPGGSFYATERIIGDIRQAHC	35
DQ002219	B	CCR5	CTRPSNNTRKSITIGPGRAFYATGDIIGDIRKAHC	35
DQ235648	C	CCR5	CTRPGSNTRRSIRIGPGQAFYTQDIIGDIRQAHC	34
AF541036	B	CCR5	CTRPSNNTRKSIPMGPGKAFYTTGEIIGNIRQAHC	35
DQ002227	B	CCR5	CARPSNNTRKSITIGPGRAFYATGDIIEDIRKAHC	35
KF770455	C	CCR5	CTRPGNNTRRSVRIGPGQAFYTTGEIIGDIRVAHC	35
AF153170	C	CCR5	CTRPNNNTRQSIRIGPGQVFYATGDIIGDIRQAHC	35
AF153129	C	CCR5	CTRPNNNTRKSIRIGPGQTFYATGGIIGNIREAHC	35
KF716495	B	CCR5	CTRPSNNTMKSIHIGPGRAFYTTEQVIGDIRKAHC	35
AY170667	C	CCR5	CTRPGNNTRKSVRIGPGQAFYATGDIIGDIRKAYC	35
U08688	B	CCR5	CTRPNNNTKSIHIGPGSAFYATGDIIGDIRQAHC	34
HM239527	B	CCR5	CTRPNNNTRKSIQGPGKAIYATGEIIGDIRKAHC	34
AY842809	B	CCR5	CTRPNNNTRKSIHMGPGRAMYATGDIIGDMRQAHC	35
DQ002104	B	CCR5	CTRPNNNTRRSITIGPGKAFYTTGGVIGDIRKANC	35
EU575611	B	CCR5	CTRPNNNTRKSIHIGPGGAFYAAGGIIGNIRQAHC	35
KC312573	B	CCR5	CERPNNNTRKSIRIGPGSAFYAAGEIIGNIRQAHC	35
EU575279	B	CCR5	CTRPNNNTIKGIHIGPGRAFYTTGQVIGDIRKAYC	35
AF082379	B	CCR5	CTRPNNNTRKSIPMGPGRAFYATGQIIGDIRQAYC	35
HM239614	B	CCR5	CTRPNNNTRRSVHFAPGRAFYATGDIIGDIRQAHC	35
JF507766	B	CCR5	CTRPNNNTRKGIHIGPGRVFYATEGIIGDIRRAYC	35
JQ779247	C	CCR5	CIRPNNNTRKSIRIGPGQAFFATTGIIGNIRQAYC	35
HM246194	B	CCR5	CTRPSNNTRRGIHMAGRAFYATGQIIGDIRQAHC	34
DQ002327	B	CCR5	CTRPNNNTRRSIHMGPGKAFYTTGEIVGNIRQAYC	35
AY887877	C	CCR5	CTRPGNNTRKSIRIGPGQTFYATGDIIGDIRRAYC	35
DQ382366	C	CCR5	CTRHNNNTRKSVRIGPGQTFYATGDIIGDIRQAHC	35
AF180906	B	CCR5	CTRPNNNTRKSINIGPGRAFYTTGQVIGDIRKAHC	35
DQ061753	B	CCR5	CTRPNNNTRRSISLGPGRAYYATGDIVGDIRQAHC	35
FJ375996	C	CCR5	CTRPNNNTRRGIGIGPGQTFFATDAIIGDIRQAHC	35
JF507980	B	CCR5	CTRPNNNTRKSINIGPGRAFYTTGAIIGNIRQAHC	35
AY010876	B	CCR5	CTRPNNNTRTSIPMGSGRAMYATGDIIGNIRQAYC	35
DQ869016	B	CCR5	CTRPNNNTRKSISIAPGRAWYATGDIIGDIRQAHC	35
FJ798398	B	CCR5	CTRPNNNTRKGISIGPGRAFYATGGIIGDIRKAHC	35
JF508097	B	CCR5	CTRPNNNTRKGIHIGPGRTFHVTGEIIGDIRQAHC	35
HQ678266	B	CCR5	CSRPNNNTRKSIHIAPGRTFYATGDIIGDIRQAHC	35
HM239535	B	CCR5	CVRPNNNTRRSIPLGPGKTFYAGEVIGDIRQAHC	34
DQ235631	C	CCR5	CIRPGNNTSKSIRIGPGQTFYATGDVIGNIRQAHC	35
HM246195	B	CCR5	CTRPNNNTRKSIPMGPGAAIYATGAIIGDIRQAHC	35
FJ977086	C	CCR5	CTRPGNNTRKSVRIGPGQTFYATGDIIGNIRQAHC	35
EU272248	B	CCR5	CIRPNNNTRKSIPIGPGRAWYTTGQIIGDIRQAHC	35
JF507750	B	CCR5	CTRPNNNTRKSIPIGPGRVFFATGGIIGDIRRAHC	35
JX140656	B	CCR5	CTRPNNNTRKGIQMGPGRAFYATGDIIGDIRQAHC	35
HQ644790	B	CCR5	CTRPNNNTRRSIHIGPGRAFYTTGEIVGDIRQAHC	35
GU204921	B	CCR5	CRPNNNTRKSIHLTPGAFHTTGSIIGDIRAC	31
JF507983	B	CCR5	CTRPNNNIRKSINIGPGRAFYTTGAIIGDIRQAHC	35
DQ516307	B	CCR5	CTRPNNNTRKSIHIGPGRALYTTGGIIGDIRQAHC	35
JN002043	B	CCR5	CTRPNNNTRKSIPIGPGRAFYATGDIIGNIRQAHC	35
HM215423	B	CCR5	CTRPNNNTLKSIQLGLGRAWHATGQIIGDIRQAHC	35
AY426113	B	CCR5	CTRPNNNTRKSIHIGPGRKIYTTGKIIGDIRQAHC	35
DQ002241	B	CCR5	CTRPNNNTRKSIHIAPGRAFYATGEIIGDIGQAHC	35
HQ644897	B	CCR5	CTRPNNNTRKSIHIGPGRAFYTTGDIIGDIRKAYC	35
HM239593	B	CCR5	CTRPNNNTRRSVPLGPGRAVYATGAIIGDIRQAYC	35
DQ002087	B	CCR5	CTRPNNNTRKSIHIGPGRAFYATGNIIGNIRQAHC	35
U08689	B	CCR5	CTRPGNNTRRSIHMGWGRAFYATGDIIGDIRQAHC	35
AY887864	C	CCR5	CIRPGNNTRKSIRIGPGQTFYATGDIIGDIRKAHC	35

DQ382375	C	CCR5	CTRPNNNTRRSIRIGPGQTFYTNDIIGDIRQAYC	34
AF541084	B	CCR5	CVRPNNNTRRGIHIGLGRRFYTTEIVGDIRRAYC	34
DQ388515	C	CCR5	CVRPNNNTRKSVRIGPGQTFFATGEIIGDIRQAHC	35
JN002040	B	CCR5	CTRPNNNTRKSIHMGPGKAFYATGDIIGDMRRAHC	35
HM239605	B	CCR5	CTRPNNNTRRSIHIAPGKAFYATGDVIGDIRQAHC	35
DQ516215	B	CCR5	CTRPNNNTRKGIHIGPGSAFYTTGAIIGNIRQAHC	35
KC156330	C	CCR5	CARPHNNTRKSMRIGPGQAFYATGDVTGDIRQAHC	35
DQ516225	B	CCR5	CTRPNNNTRKGIHIGPGRAFYTTGAIIGSIRQAHC	35
AY669749	C	CCR5	CTRPNNTRKSIRIGPGQTFYATNEIIGNREAHC	33
DQ061848	B	CCR5	CTRPNNNTRKGIHMGPGKMFYATGQIIGDIRQAYC	35
EU576774	B	CCR5	CTRPNNNTRKSIPIGPGSVFYTGEIIGDIRQAHC	34
EF117274	C	CCR5	CTRPGNNTRKSIRIGPGQTFYATGEIIGNIRQAHC	35
JQ779236	C	CCR5	CTRPNNNTRQSIRIGPGQAFFATTGIIGNIRQASC	35
EU744170	B	CCR5	CTRPNNNTRRSIHIGPGRAIYATGDIIGDIRKAHC	35
HQ708057	C	CCR5	RIRPNNNTRKSVRIGPGQTFYATGGIIGDIRRAYC	35
JX140659	B	CCR5	CTRPNNNTRKGIHIGPGKTFFATEVIGDIRKAHC	34
JF507793	B	CCR5	CMRPGNNTRKSITIGPGRAFYAGEIIGNIRQAHC	34
JN188292	C	CCR5	CTRPGNNTRKSVRIGPGQTFFATGEIIGKIREAHC	35
EU577152	B	CCR5	CTRPNNNTRRSITFGPGAAFYTGEIIGDIRQAYC	34
JF896831	B	CCR5	CTRPGNNTRKSIHMGPGRAFYTTGEIIGDIRLAHC	35
DQ177210	B	CCR5	CTRPNNNTRRSITIGPGRAFYATGDIIGDIRQAHC	35
JX140667	C	CCR5	CTRPHNNTRKSVRIGPGQTFYATGEIIGNIRQAHC	35
JF507803	B	CCR5	CMRPGNNTKKSITIGPGKAFYAGEIIGDIRKAHC	34
KF770389	C	CCR5	CTRPGNNTRKSMRIGPGQTFYATGDIIGNIRQAHC	35
HM239524	B	CCR5	CTRPSNNTRKSIPMGPGKAFYATGEIVGDIRQAHC	35
AF153180	C	CCR5	CTRPNNNTRRSYGIGPGQAFRATTNIIGDIRKAHC	35
HM239576	B	CCR5	CTRPNNNTRKGIHMGPGGAFYATGEIIGNIRQAHC	35
EU744066	B	CCR5	CIRPNNNTRKSIHLGPGRAFYATGDIIGDIRQAHC	35
AF384332	B	CCR5	CIRPNNNTRKGIHIGPGRTFYGTGEIIGNIRQAHC	35
AB553914	B	CCR5	CTRPNNNTRKGIHFGPGQALYTTGAIIGDIREAHC	35
EF657907	B	CCR5	CIRPSNNTRKSIPMGPGRAFYATGDITGNIRQAHC	35
HQ644867	B	CCR5	CTRPGNNTRKSIHMGPGRAFFVTDDVIGDIRQAHC	35
FJ977079	C	CCR5	CTRPGNNTRTSIRIGPGQTFYATGDIIGDIRQAHC	35
KF716466	C	CCR5	CTRPNNNTRKNVRIGPGQAFYATNGIIGDIRQAYC	35
AF541024	B	CCR5	CTRPNNNTRKGINIGPGRAFYTTGEIIGDIRQAHC	35
KC473831	B	CCR5	CTRPNNNTRRSIHLGPGKAIYTTGEIIGDIRRAHC	35
JF896842	B	CCR5	CVRPNNNTRKGIHIGPGRAFYATGEIIGNIRQAHC	35
HQ645001	B	CCR5	CTRPNNNTRRSITMGPGKAFYTTGDIIGDIRRAHC	35
U04925	B	CCR5	CTRPSNNTRKSVHIGPGRAFYTTGEIIGDIRQAHC	35
EF688440	B	CCR5	CTRPHNNTRKGIHIGPGRAFYTAEKIVGDIRQAHC	35
DQ061683	B	CCR5	CIRPNNNTRKGIHLGPGGAFYATGGIIGDIRQAYC	35
AF391233	C	CCR5	CTRPNNNTRKSIRIGPGQTFYATNGIIGNIRQAHC	35
EU575091	B	CCR5	CTRPNNNTRKSIGMGPGRAFYATGDIIGDIRQAHC	35
AF254766	C	CCR5	CTRPNNNTRKSVRIGPGQAFYATNDVIGDIRQAHC	35
HQ644852	B	CCR5	CTRPGNNTRKSITIGPGRAFYTTGDIIGDIRQAHC	35
DQ061478	B	CCR5	CTRPNNNTGKSISIGPGRAFYTTGEIIGEIRQAYC	35
AF254778	B	CCR5	CTRPNNNTRKSINLGQGRAWYATGAIIGDIRQAHC	35
DQ002092	B	CCR5	CTRPSNNTRRGIHIGPGRAFYTTGEVIGDIRAANC	35
AF384296	B	CCR5	CTRPNNNTKKSIHIGPGRAVYATGQIIGDIRQAHC	35
HQ678286	B	CCR5	CTRPNNNTRKSIHMGPGGAFYATGDIIGDIRQAHC	35
AY842824	B	CCR5	CIRPNNNTRKSINIGPGRAMYATEQITGDIRQAHC	35
EU744082	B	CCR5	CTRLNNNTRKSIPLGPGRAFYATGDIIGDIRKAQC	35
EF579978	B	CCR5	CERPGNNTSKGIHIGPGRAFCATENIIGDIRKAHC	35
U04908	B	CCR5	CTRPNNNTRKGIHIGPGRAFYTTGEVIGNIRQAHC	35
U04917	B	CCR5	CIRPNNNTRKSIHIGPGRAFYTTGDIIGDIRKAHC	35
AF384258	B	CCR5	CIRPSNNTRKSIPMGPGRAWYATGSIIGDIRQAHC	35
AF384282	B	CCR5	CTRPNNNTRKSIHIGPGRAFYTTGQIVGDIRKAHC	35
EU578561	B	CCR5	CTRPNNNTRRSIHMGPGKALYTGDIIGDIRQAHC	34
AB553912	B	CCR5	CTRPNDNTRKSINIAPGRAFYATGDIIGDIRQAHC	35
JF896824	B	CCR5	CTRPNNNTRRSISIGPGRAFFATGEVIGDIRKAYC	35
KC156277	C	CCR5	CTRPNNNTRKSMRIGPGQTFYATGDIIGNIRQAHC	35
DQ869019	B	CCR5	CTRPSNNTRKGIHIGPGRAFYTTGDIIGDIRQAHC	35
AY887891	C	CCR5	CTRPSNNTRTSIRIGPGQTFYATGEIIGDIRKAHC	35
AF153145	C	CCR5	CTRPNNNTRKSIRIGPGQTFFATNAIIGDIRQAHC	35
DQ061540	B	CCR5	CIRPNNNTRKSIHIAPGRAFYATGEIIGDIRQAHC	35
KC312477	B	CCR5	YKTQQQYKERYTYRTRENIYATGEIIGDIRQAHC	34
EU744166	B	CCR5	CTRPNNNTRKGIHIGPGRAFYTSGGIIGDIRQAHC	35
AF384273	B	CCR5	CTRPNNNTRKSIPMGPGKFYATGEIIGNIRQAHC	34
JF896844	B	CCR5	CTRPNNNTRKGIHMGPGRAFYATEIIGNIRQAHC	34
JN002009	B	CCR5	CTRPHNTRKSIHIGPGRAFYATGDIVGDIREAHC	34

EU575538	B	CCR5	CTRPSNNTRKSIHMGPGGAFYATGSIIGDIRQAHC	35
FJ375981	C	CCR5	CTRPNNNTRKSRIGPGQTFYATGDIIGDIRQAHC	34
KF770313	C	CCR5	CIRPDNNTRRSIRIGPGQVFYANDIIGDIREASC	34
AY887855	C	CCR5	CTRPSNNTRKGIRIGPGQTFYATGDVIGDIRQAHC	35
KC156320	C	CCR5	CNRPHNNTRKSMRIGPGQAFYATGDVTGDIRKAHC	35
AY835435	B	CCR5	CTRPNNNTRKSIHMGPGKVFYTTGEIIGDIRQAHC	35
DQ002118	B	CCR5	CTRPNNNTRKSITIGPGGAFYATGDIIGDIRQAHC	35
AY887868	C	CCR5	CTRPNNNTRQSVRIGPGQTFYATNIIGDIRQAYC	34
JF507855	B	CCR5	CTRPGNNTRRSINIGPGRAFYTGEMIGDIRQAHC	34
HQ644839	B	CCR5	CIRPNNNTRRSINIGPGRAFYTTGDIIGDIRQAHC	35
HM239539	B	CCR5	CTRPNNNTRRSIPIGPGRAFWATGDIIGDIRQAHC	35
EU575025	B	CCR5	CTRPNNNTRKGIHIGPGRAFYATGQIIGDIKRAYC	35
AY835438	B	CCR5	CTRPNNNTRKSINLGPGRAFYATGDIIGDIRQAHC	35
JF508106	B	CCR5	CTGPNNNTRKSIHIGPGRTFYATGEIIGDIRQAHC	35
DQ002170	B	CCR5	CTRPNNNTRRSITIGPGGAFYATDIIGDIRQAHC	34
FJ670524	B	CCR5	CIRPNNNTRKSIHIGPGRAFYAQDIIGDIRQAHC	34
EU272300	B	CCR5	CTRPNNNTRKSIHLGLGRAWYATGEIIGNIRQAHC	35
AF153160	C	CCR5	CTRPNNNTRKSVRIGPGQTFYATGEVIGNIRQAHC	35
HQ644817	B	CCR5	CIRPNNNTRRSIHIGPGRAFFATGDIIGDIRQAHC	35
HM215413	B	CCR5	CTRPNNNTRKSMTLGPGRAWYTTGQIIGDIRKAHC	35
DQ061815	B	CCR5	CTRPNNNTRKGMHMGPGKVFYATGQIIGDIRQAHC	35
KC312590	B	CCR5	CERPNNNTRKSIRIGPGSAFYATGEIIGNIRQAHC	35
JQ779233	C	CCR5	CIRPGNNTRKSIRIGPGQVFYATNIIGDIREAHC	34
AY010787	B	CCR5	CIRPNNNTRKSIHVGPGKAFYATGDIIGDIRQAYC	35
DQ061422	B	CCR5	CTRPNNNTRKSMHIGPGRAFYTTGEIIGDIRQAHC	35
AF384308	B	CCR5	CTRPNNNTRKSIHIGPGRAFYATGDIIGDIIQAHC	35
DQ061425	B	CCR5	RTRPNNNTRKSIHIGPGRAFYATGEIIGDIKQAHC	35
DQ235617	C	CCR5	CTRPSNNTRKSIRIGPGQAFFATGEIIGDIRQAHC	35
EU908221	C	CCR5	CTRPDNNTRKSIRIGPGQTFYATGDIIGDIRQAHC	35
JF896837	B	CCR5	CTRPNNTRRSINIGPGRAWYTTGEIVGDIRQAHC	34
DQ388516	C	CCR5	CMRPGNNTRRSVRIGPGQTFYATGEIIGDIRQAHC	35
FJ375985	C	CCR5	CTRPGNNTRRSVRIGPGQAFYATGDIIGDPRQAHC	35
EU908225	C	CCR5	CTRPNNNTRRSIRIGPGQTFYATGDIIGNIRQAYC	35
DQ002337	B	CCR5	CTRPSSNTRKSIHLGLGRAFYATGEIIGDIRQAHC	35
FJ375984	C	CCR5	TRPNNNTRKSIRIGPGQAFYATKDIIGDIRKAHC	34
DQ061819	B	CCR5	CTRPNNNTRKGIHMGPGKVFYATGQIIGNIRQAHC	35
AY510060	C	CCR5	CTRPNNNTRRGIRIGPGQTFYATGGIIGDIRQAHC	35
JX140666	C	CCR5	CTRPNNNTRKSIRIGPGQTFYATNDIIGDIREAHC	35
AF541028	B	CCR5	CTRPNNNTRKSIHIGPGRAFYTTGEIIGDIKQAHC	35
AY159664	B	CCR5	CTRPNNNTRKGIHIGPGAALYATGAIIGNIRQAHC	35
DQ869017	B	CCR5	CIRPNNNTRRSIPIGPGRAFYATGDIIGDIRQAHC	35
AF153140	C	CCR5	CTRPGNNTRKSMRIGPGQTFFATGEIIGDIRQAHC	35
AY010798	B	CCR5	CMRPSNNTRKSISIGPGRAFYTTGEIIGDIRQAHC	35
HM368243	B	CCR5	CTRPNNNTRRGIHIGPGGAFYSTGDIIGNIRQAHC	35
HQ644951	B	CCR5	CIRPNNNTRKSIHIGPGRAFYATGEIIGDIRQAYC	35
EU578340	B	CCR5	CTRPNNNTRRSTHLGAGRALYTGEIIGDIRQAHC	34
EU744122	B	CCR5	CTRPNNNTRKSIHIGPGKAFYATGEIIGNIRQAHC	35
DQ061829	B	CCR5	CTRPDNNTRKGIHMGPGKVFYATGQIIGDIRQAHC	35
EU272264	B	CCR5	CTRPNNNTKKSINLGPGRAWYATGQIIGDIRQAHC	35
EU578358	B	CCR5	CTRPNNNTRKSIHIGPGSVFYTGEIIGNIRQAHC	34
HQ678276	B	CCR5	CTRPNNNTRKSIHIGPGRAFYATGEIIGDIRKAHC	35
AF153177	C	CCR5	CTRPNNNTRKSIRIGPGQTFYATNEIIGNIRQAHC	35
KC312395	B	CCR5	TRPNNNTRKSIHIGPGSAFYTTGEIIGDIRQAHC	34
EU744087	B	CCR5	CTRPHNNTRKSIHLGPGRAFYATGDIIGNIRQAYC	35
DQ235624	C	CCR5	CIRPNNNTRKSIRIGPGQTFYATGDIIGDIRKAHC	35
EU744069	B	CCR5	CTRPHNNTRRSIHMGPGKTFYATGDIIGDIRQAHC	35
HM239574	B	CCR5	CTRPSNNTRKSINIGPGRAFYATGDIIGDIRQAHC	35
HM239584	B	CCR5	CTRPSNNTRKDIHIGPGRAFYTTGEIIGDIRKAHC	35
AF112565	B	CCR5	CTRLNNNTRKSIHIGPGRAFYATGDIIGDIRQAHY	35
JF507871	B	CCR5	CTRPGNNTRRSINIGPGRAFYTGEIIGNLRQAHC	34
HQ708053	C	CCR5	CIRPNNNTRKSVRIGPGQTFYATGGIIGDIRRAYC	35
JF507737	B	CCR5	CTRPNNNTRKSIHIGPGRTFFATGEIIGDIRRAHC	35
EU272288	B	CCR5	CTRANNNTRKSIPLGPGKAWYTTGDIIGDIRQAHC	35
DQ235646	C	CCR5	CTRPGNNTRKSIRIGPGQTFYATGDIIGDIRKAYC	35
HQ644795	B	CCR5	CTRPNNNTRRSIHIGPGRAFYTGEIIGDIRKAHC	34
DQ235626	C	CCR5	CTRPNNNTRRSIRIGPGQTFYATGDIIGDIRQAHC	35
DQ177207	B	CCR5	CTRPNNNTRKSITLTPGRAFYATGDIIGDIRQAHC	35
JF896818	B	CCR5	CTRPNNNTRKGIHIGPGRAIYATGAIIGDIRQAHC	35
HM239607	B	CCR5	CRPNNNTRKSIHIAPGRAFYATGDIIGDIQAYC	33
HQ708061	C	CCR5	CTRPNNNTRKSVRIGPGQTFYATGGIIGNIRQAYC	35

HQ377465	B	CCR5	CTRPNNNTRKSISMGPGRAFYATGEIIGNIRQAHC	35
DQ061757	B	CCR5	CTRPNDNTRRSISLGPGRAYYATGDIIGDIRQAHC	35
AF194979	B	CCR5	CTRPNNNTRRSIHIGPGKAFYTGRNNRNIRQAHC	34
JF507858	B	CCR5	CTRPGNNTGRSINIGPGRAFYTGEIIGDIRQAHC	34
AF310125	B	CCR5	CTRPNNNTRKSIHLGAGRALYTREIIGDIRQAHC	34
EF688456	B	CCR5	CTRPNNNTRKSINMGPGRAFYATGDIIGDIRQAHC	35
AF153163	C	CCR5	CTRPGNNTRRSVRIGPGQAFYATGEIIGNIRRAHC	35
AY510058	C	CCR5	CTRPGNNTRKGIWIGPGQAFYATGDIIGDIRQAHC	35
DQ869023	B	CCR5	CARPNNSTRKGIHIGPGRAFYAAADIIGDIRQAHC	35
EF117267	C	CCR5	CTRPNENRRKSIRIGPGQAFYATGDIIGDIRQARC	35
AY010881	B	CCR5	CTRPNNNTRTGIPMGPGRAMYATGDIIGNIRQAYC	35
AY669715	B	CCR5	CTRPNNNTRKSIHMGGRFYATGEIIGNIRQAHC	33
GU204932	B	CCR5	CTRPNNNTRRSISLGPGRSIYTTGQIIGDIRQAHC	35
EU577288	B	CCR5	CTRPNNNTRRGIHVGPGQALYTGDIIGDIRQAHC	34
HQ644866	B	CCR5	CTRPGNNTRKSIHMGPGRAFYATEDVIGDIRQAHC	35
EU604557	B	CCR5	CTRPSNNTRKSINMGPGRAFYTTGEIIGNIRQAHC	35
HQ678287	B	CCR5	CIRPNNNTRKSINIGPGRAFYATGDIIGDIRRAHC	35
JF508061	B	CCR5	CTRPNNNTRKSIHIGPGRAFYTTGEIIGNIRQAFC	35
DQ869014	B	CCR5	CTRPNNNTRKSIPIGPGRAFFATDIIGDIRQAHC	34
EU578424	B	CCR5	CTRPNNNTRRGVTIGPGRVFYTGQVIGDIRQAHC	34
KC473835	B	CCR5	CTRPNNNTRKGIHIAPGRAFYATGDIIGDIRQAHC	35
HM239538	C	CCR5	CTRPNNNTRESIRIGPGQTFYATGDIIGDIRQAYC	35
AJ418522	B	CCR5	CTRPNNNTRKSIPIGPGGAFYTTGEIIGDIRKAHC	35
HQ678293	B	CCR5	CIRPGNNTRKSIHIAPGRAFYATGDIIGDIRQAHC	35
FJ375972	C	CCR5	CTRNNTRKSRIGPGQTFYATGIIGIRQAHC	30
AF194978	B	CCR5	CTRPNNNTRRSIHIGPGKAFYTGEIIRNIRQAHC	34
HQ644885	B	CCR5	CTRPNNNTRKSINIGPGRAIYATGDIIGNIRQAHC	35
FJ375994	C	CCR5	CTRPGNNTRTSIRIGPGQTFYANNPIIGDIRQAYC	35
AF307750	C	CCR5	CTRPNNNTRKSIRIGPGQVFYATGDIIGDIRQAHC	35
HQ644895	B	CCR5	CTRPNNNTRKSIHIGPGRALYTTGDIIGDIRKAYC	35
DQ002128	B	CCR5	CTRSNNNTRKSIHIEPGRAFYATGDIIGDIRQAHC	35
U66221	B	CCR5	CTRPNNNTRRSVRIGPGGAMFRTGDIIGDIRQAHC	35
HQ708063	C	CCR5	CTRPNNNTRKSMRIGPGQTFYATEEVIGDIRQAYC	35
AY887884	C	CCR5	CTRPGNNTRKGIGIGPGQTFYAPRGIIGDIRQAHC	35
DQ061495	B	CCR5	CTRPNNNTRKSIHIGPGRAFYTTGGIIGKIRQAHC	35
HQ644909	B	CCR5	CTRPNNNTRKSIHIGPGRAFYATGEIIGDIRKAYC	35
AF384328	B	CCR5	CTRPNNNTRKSISIGPGRAFYAHGEIIGDIRQAHF	35
AF082840	B	CCR5	CTRPNNNTRKSIHIGPGKAFYATGDVIGDIRKAHC	35
DQ002089	B	CCR5	CTRPNNNTRKSIHIGPGRAFYATGNMIGNIRQAHC	35
AY510065	C	CCR5	CTRPNNSTRKSVRIGPGQAFYATGDIIGDIRQAHC	35
JN002026	B	CCR5	CTRPNNNTRKSIPMGPGKAFYATGDIIGNIRQAHC	35
HQ644972	B	CCR5	CTRPNNNTRRSIHIGPGRAFYATGDIIGDIRKAHC	35
AF153182	C	CCR5	CTRPGNNTRKSVRIGPGQTFFATGEIIGDIRQAHC	35
AF384286	B	CCR5	CTRPNNNTRKSIHIGPGRAFYARGDIIGDIRQAHC	35
DQ002315	B	CCR5	CTRPNNNTRRSIHMGPGKAFYTTGEIVGDIRQAYC	35
FJ375991	C	CCR5	CTRPNNNTRQSIRIGPGQTYATGDIIGDIRQAHC	34
AY835441	B	CCR5	CTRPNNNTRKGITIGPGRVFYTGEIVGDIRQVHC	34
DQ061584	B	CCR5	CIRPNNNTRKSIRIRPGSAFYTTGEIIGDIRQAHC	35
HM215403	C	CCR5	CTRPSNNTRKSIRIGPGQTFYATGDIIGDIRQAHC	35
AF384263	B	CCR5	CTRPNNNTRRGIHIGPGRAFYATGEIIGDIRQAHC	35
DQ516161	B	CCR5	CTRPNNNTRKDIRIGPGRAFYATGDIIGDIRQAHC	35
AY713415	C	CCR5	CTRYANNTRKSVRIGPGQTFYTNDIIGDIRQAHC	34
JN687734	C	CCR5	CTRPGNNTRKSIRIGPGQAFYATNDIIGDIRQAHC	35
DQ061482	B	CCR5	CTRPNNNTRKSISIGPGRAFYTTGEIIGETRQAYC	35
KF770339	C	CCR5	CIRPGNNTRKSMRIGPGQTFYATGEIIGDIRRAHC	35
HM239533	B	CCR5	CIRPNNNTRKGIHMGMGRAFYATGEVIGNIRQAHC	35
AF112563	B	CCR5	CTRPTNNTRKSIHIAPGSAFYATGDIIGDIRQAHC	35
DQ002074	B	CCR5	CTRPNNSTRKSIHIGPGRAFYATGEIIGNIRQAHC	35
EF688430	B	CCR5	CTRPNNNTRQGINIGPGRAFYTTGEVIGDIRQAHC	35
EU578323	B	CCR5	CTRPNNNTRKSISIGPGRAFYTGEIIGDIRQAHC	34
JF508118	B	CCR5	CTRPNNNTRKGIHIGPGRTFYATGEIIGDMRQAHC	35
HM239628	B	CCR5	CTRPNNTRKSINIGPGRAFYATGDIIGNIRQAHC	34
JX140653	B	CCR5	CIRPNNNTRKSIHMGPGGAFYATGDVIGDIRKAYC	35
HQ708033	C	CCR5	CTRPNNNIRKSVRIGPGQTFYATGDIIGDIRQAYC	35
JF896821	B	CCR5	CTRPNNNTRKSIHLTPGGAYATGDIIGDIRKAC	33
AY835450	B	CCR5	CTRPHNNTRKSIHIGPGRAWYATGDIIGDIRKAYC	35
AF153168	C	CCR5	CPRPINNTRRSVRIGPGQTFYATGEIIGNIRQAHC	35
FJ376015	C	CCR5	CTRPSNNTRESIRIGPGQTFYATGDIIGDIRQAYC	35
DQ869015	B	CCR5	CTRPNNNTRRSIPIGPGRAFYATDIIGDIRQAHC	34
KF716496	B	CCR5	CTRPSNNTRKSINIGPGRAFYTTGEIIGDIRQAHC	35

EU578312	B	CCR5	CTRPNNNTRKSITIGPGRAFYTGEIIGDIRQAHC	34
GU204919	B	CCR5	CARPNNNTRKSIHIAPGRAFYTTGSIIGDIRQAHC	35
AY123275	C	CCR5	CRPGNNTRESRIGPGQAFFATGVIGDIRKAYC	32
AY887874	C	CCR5	CARPNNNTRKSIRIGPGQTFYATNDIIGNIRQAHC	35
U04918	B	CCR5	CIRPNNNTRRSIHMGPGRAFYATGDIIGDIRQAHC	35
FJ376024	C	CCR5	CTRPNNNTRKSIRIGPGQAFWATGDIIGDIRQAC	34
JF896872	B	CCR5	CTRPNNNTRKSIHGPGSSFETTGEIIGNIRQAHC	34
EU575201	B	CCR5	CTRPSNNTSKSIPIGPGRAFYTTDRIVGDIRQAHC	35
KC473826	B	CCR5	CTRPNNNTRKSIHIAPGKAFYATGDIIGNIRQAHC	35
HM179801	C	CCR5	CIRPNNNTRTSMRIGPGQAFFATNGIIGNIRQAYC	35
EU744062	B	CCR5	CTRPNNNTRKSIHVGPGKTLYATGDIIGDIGQAHC	35
HM179793	C	CCR5	CIRPNNNTRTSIRIGPGQALFATNGIIGNIRQAYC	35
FJ376017	C	CCR5	CIRPGNNTRKSVRIGPGQTFYVNNIIGDIRQAC	33
DQ061677	B	CCR5	CIRPNNNTRKGIHLGPGGALYATGGIIGAIRQAYC	35
AF153146	C	CCR5	CTRPNNNTRKSVRIGPGQTFYATGGIIGDIREAHC	35
HQ377416	B	CCR5	CTRPNNNTRRSIHIGPGSAFYATGEIIGDIRQAHC	35
EU575795	B	CCR5	CTRPGNNTRKSIHIAPGRAFYTTGDIIGDIRQAHC	35
FJ977090	C	CCR5	CTRPNNNTRKSVRIGPGQTFYATGDIIGNTRQAYC	35
JQ779251	C	CCR5	CIRPNNNTRKSIRIGPGQAFFATTGIIGNIRQAHC	35
EU576666	B	CCR5	CTRPNNNTRKGIHIGPGKTLYATGEIIGDIRQAHC	35
DQ061771	B	CCR5	CTRPNDNTRKGIHIGPGRAFYATGEIIGNIRQAHC	35
AY010858	B	CCR5	CTRPNNNTGTCIPMGPGRAVCATGDIIGNIRQAYC	35
KC473824	B	CCR5	CIRPNNNTRKSIHLGPGRAFYATGEIIGDIRKAHC	35
AY887882	C	CCR5	CVRPNNNTRKSMRIGPGQTFYATGEIIGDIRQAHC	35
JF508043	B	CCR5	CTRPNNNTRKSIPIGPGKAFYTTGEIIGDIRQAHC	35
AY010859	B	CCR5	CTRPNNNTGTSIHMGPGRAVYATGDIIGNIRHAYC	35
EU576296	B	CCR5	CIRPNNNTRKGIHIGPGRTFYTTGDIIGDIRQAYC	35
EU578541	B	CCR5	CTRPNNNTRRSIPLGPGAAFFTGEIIGDIRQAYC	34
DQ061451	B	CCR5	RTRPNNNTRKSIHIGPGRAFYTTGEIIGDIRKAYC	35
AF153159	C	CCR5	CTRPNNNTRTSIRIGPGQTFYATGDIIGDIRQAHC	35
JF896838	B	CCR5	CTRPNNNTRKSIPMGPGKAFYATGAIIGIRQAHC	34
DQ177195	B	CCR5	CTRPGNNTRKSIHIGPGRAFYTTGSIIGDIRKAHC	35
DQ235644	C	CCR5	CIRPNNNTRKSIRIGPGQVFYANNDIIGDIRQAHC	35
DQ516173	B	CCR5	CTRPNNNTRKGIHIGPGRAFFTTGAIIGNIRQAHC	35
HM239573	B	CCR5	CTRPNNNTRRSIHLGPGRTIFATGTVIGEIRRAHC	35
HQ678260	B	CCR5	CTRPNNNTRKRITIGPGKVFYATGDIIGDIRQAHC	35
DQ002183	B	CCR5	CARPNNNTRRSITIGPGRAFYATDIIGDIRKAHC	34
AF254779	B	CCR5	CTRPGNNTRKSISMGPGRAFYATGDIIGDIRQAYC	35
AF153169	C	CCR5	CTRPNNNTRKSVRIGPGQTFYATGSIIGDIRQAHC	35
DQ178989	B	CCR5	CTRPNNNTRRSIPIGPGRAFYATGDIIGDIRKAHC	35
DQ002307	B	CCR5	CTRPNSNTRKSINIGPGRAWYTTGDIIGDIRKAHC	35
AF384256	B	CCR5	CTRPSNNTRKGIHIGPGRAWYATGSITGDIRQAHC	35
JQ779177	C	CCR5	CTRPGNNTRKSVRIGPGQTFYATGQIIGNIREAHC	35
AF384275	B	CCR5	CTRPNNNTRKSIHMGPGGSFYATGDIIGNIRQEHC	35
HM239597	B	CCR5	CTRPNNNTRKSINIAPGRAFYATGDVIGDIRQAHC	35
EU575376	B	CCR5	CTRPNNNTRKSITFGPGRAFYTTGDIIGDIRKAYC	35
FJ670525	B	CCR5	CTRPNNNTRQGIHIGPGRAFYATTDIVGNIRKAHC	35
DQ002186	B	CCR5	CTRPGNNTRKSIHIGPGRAFYATDIIGDLRQAYC	34
AY010850	B	CCR5	CTRPNNNTRKSIHLGPGSAIYATGDIIGDIRQAHC	35
JQ779204	C	CCR5	CTRPGNNTRRSVRIGPGQTFYATEEIIGDIREAHC	35
AY010855	B	CCR5	CTRPNNNTRKRIRIGPGRAFYTTGAIIGDIRQAHC	35
AF153166	C	CCR5	CTRPINNTRRSVRIGPGQTFYATGGIIGNIRQAHC	35
AF384250	B	CCR5	CTRPNNNTRKSIHMGPGRAFYTTGNIIGDIRQAHC	35
HM239620	B	CCR5	CTRPNNNTRKSINIGPGRAFFTTGDIIGDIRQAHC	35
HQ644996	B	CCR5	CTRPNNNTRKSIPMGPGRAFYATGDIIGDIRQAHC	35
AJ418483	B	CCR5	CTRPNNNTRKSIHVGPGRALYATGDIIGEIRQAFC	35
AF021494	B	CCR5	CTRPNNNTRRSIHIGPGRAFYTTGRIIGDIRQAYC	35
HM368255	B	CCR5	CTRPNNNTRRGIHIQPGGAFYATDRIIGDIRQAHC	35
EF688443	B	CCR5	CTRPNNNTKRSIYMGPGRTVHTKGRIIGDIRQAHC	35
AF153137	C	CCR5	CIRPNNNTRKSVRIGPGQTFYATEIIGEIRQAHC	34
AY170663	C	CCR5	CTRPSNNTRTSVRIGPGQTFFATNDVIGDIRQAHC	35
DQ002254	B	CCR5	CTRPNNNTRKSIPIGPGRAFYTTGGIIGDIRQAHC	35
HQ644846	B	CCR5	CTRPNNNTRRSINIGPGRAFYTTGDIIGDIRQAYC	35
EU786674	B	CCR5	CTRPGNNTRRSISMGPGRAIYATGEIIGDIRQAYC	35
FJ376019	C	CCR5	CMRPGNNTRKSIIGPGRAFYAGDIIGDIRQAHC	33
JF508032	B	CCR5	CTRPNNNTRKSINIGPGRAFYTTGEIIGNIKQAHC	35
JN002013	B	CCR5	CTRPNNNTRKSIHIGPGRAFYATGEIVGDIREAHC	35
DQ235635	C	CCR5	CIRPGNNTRRSVRIGPGQTFFATGDIIGDIRQAHC	35
HQ678265	B	CCR5	CTRPNNNTRRSIPMGPSKAFYATGDIIGNIRQAHC	35
EU575148	B	CCR5	CERPNNNTRRSIHIGPGRAFYAGEIIGNIRKAYC	34

HM239608	B	CCR5	CIRPNNNTRKSINIGPGKAFYATGGIIGDIRQAHC	35
KF384798	B	CCR5	CTKHSINKRKRVTIGPGRVYYSTKEIIGDIRKAHC	35
AF153178	C	CCR5	CTRPNNNTRKSVRIGPGQTFYATDIIGDIRQAHC	34
HM239555	B	CCR5	CTRPSNNTRKIPIGPGRAFYATGDIIGNIRQAHC	34
HQ708023	C	CCR5	CIRPNNNTRKSVRIGPGQTFYATGDIIGNIRKAYC	35
DQ061697	B	CCR5	CTRPNNNTRKGIHMGPGKAFYATGDIIGNIRQAHC	35
AY510061	C	CCR5	CTRPNNNTRKSVRIGPGQALYATGGIIGDIRQAHC	35
AF254772	C	CCR5	CTRPNNNTRRSIRIGPGQAFYATGDIIGDIRQAHC	35
FJ653125	B	CCR5	CTKFNNNTRKSIHIGPGRAFYATGDIIGNIRQASC	35
JF896816	B	CCR5	CTRPNNNTRKSIHMGPGRAYATGEIIGIRQAC	32
EU743997	B	CCR5	CTRPNNNTRKSISIGPGRAFYATGEIIGNIRQAHC	35
DQ516311	B	CCR5	CTRPDNNTRKSIHIGPGRAFYTTGGIIGDIRQAHC	35
FJ375968	C	CCR5	CTPNNTRKSIRIGPGQTFYTNIIGDIRKAHC	31
AF355644	B	CCR5	CTRPNNNTRKSINIGPGRAWYATGKIIGNIRQAHC	35
U08703	B	CCR5	CTRPNNNTRKGIHMGWGRAFYATGEIIGNIRQAHC	35
GU945312	C	CCR5	CIRPNNNTRKSIGIGPGQTFYATGDIIGDIRQASC	35
KF770331	C	CCR5	CIRPGNNTRKGMRIGPGQTFYATGEIIGDIRQAHC	35
HQ678257	C	CCR5	CIRPNNNTRRSVRIGPGQTFYATGDIIGDIRKAYC	35
AF384284	B	CCR5	CTRPNSNTRKGIHIGPGRAFYTTGEIVGDIRQAHC	35
JF507909	B	CCR5	CTRPNNNTRQSVHIGPGRALYTTDIIGDIRKAYC	34
AY736826	C	CCR5	CTRPNNNTRKSIRIGPGQTFYATDIIGDIRQAYC	34
KF716467	C	CCR5	CVRPNNNTRKSLRIGPGQTFYATGDIIGDIRQAHC	35
AY010853	B	CCR5	CTRPSNNTRKSIHIGPGRAFYTTGSIIGDIRQAHC	35
U08704	B	CCR5	CTRPNNNTRKSIHMGWGRAFYATGDIIGDIRQAHC	35
EU576909	B	CCR5	CTRPNNNTRKGIHIGPGKVFYTGEIVGDIRQAHC	34
AY887880	C	CCR5	CTRPANNTRKSVRIGPGQTFYATGAIIGDIRQAHC	35
DQ061536	B	CCR5	CTRPNNNTRKSIPIGPGRASYATGDIIGDIRQAHC	35
EF657904	B	CCR5	CIRPNNNTRKSIPMGPGRAFYATGAIIGNIRQAHC	35
AF384262	B	CCR5	CTRPNNNTRKGIHIGPGRAFYATGEIIGDIRQAHF	35
FJ846652	C	CXCR4	CTRPNNNTRKSMRIGIGRGHAFYTTGKVIGNIRQAH	37
FJ375993	C	CXCR4	CARPNYTRQRIGIGRGQALFTARRIIGNIKQAHC	34
AF021617	B	CXCR4	CTRPNNNTRRRIHIGPGRAVYTTGQIIGDIRKAYC	35
FJ653149	B	CXCR4	CKRPNNNARRRIHIGPGRAFYATDIIGNIRQAYC	34
EU604549	B	CXCR4	CTRPSNHTRKRVTLGPSRVYYTTGEITGDIRRAHC	35
KF770414	C	CXCR4	CMRPGNNTRRRVRIGPGQTFYATGNIIGDIRQAHC	35
DQ382378	C	CXCR4	CTRPGKGKRTRVRIGPGRTFYATGAVTGDIRKAHC	35
FJ846632	C	CXCR4	CTRPDNKISMKRIKIGPGRAFVATKGIKGDIRQAYC	36
AF146728	B	CXCR4	CMRPNNNTRKGIYVGPGRHIYATEKIVGDIRQAHC	35
FJ846648	C	CXCR4	CTRPNNNTRKSVRIGIGRGHAFYTTGKVIGNIRQAH	37
AF259007	B	CXCR4	CTRPNKTIRKGLRLGPGRAFYTMGRIEGYIRQAHC	35
X01762	B	CXCR4	CTRPNNNTRKSIRIQRGPGRAFVTIGKIGNMRQAHC	36
KF770412	C	CXCR4	CTRPNINNVRNVRIGPGRALFKTGKMTGDIRQASC	35
AY842799	B	CXCR4	CTRPNNNTRKRITAGPGRVLYTTGQIIGDIRRAHC	35
FJ798547	B	CXCR4	CTRPNNNTRKRITMGPGRVYYTTGQIIGNIRQAHC	35
FJ798429	B	CXCR4	CTRPNNNTRNRISIGPGRAFYTTRQVIGDIRQAHC	35
JN001990	B	CXCR4	CTRPNNNTKKGIYVGPGRKVYTTDRIIGDIRQAHC	35
KF770420	C	CXCR4	CTRPSNNTRRRVRIGRGQAFDATGQIIGDIRQAHC	35
AF355735	B	CXCR4	CTRPGNKTIKSISLGPGRAFSATRQIIGDIRKAYC	35
AY736819	B	CXCR4	CTRPNYNKRKRIHIGPGRAFYTTKNIIGTIRQAHC	35
FJ541293	C	CXCR4	CTRPNNNTRKSVRIGIGRGQAIYAKKAIIGDIRQAH	37
AY842828	B	CXCR4	CARPNNNTRKRIYMGTGRYMSATEKITGDIRQAHC	35
AF258981	B	CXCR4	CTRPNKNIRKGLRLGPGRAFYTMGGIVGYIRQAHC	35
KF770417	C	CXCR4	CVRPNNNTRKSVRIGRGQTFYANRIIGDIRQAHC	34
FJ846659	C	CXCR4	CIRPGNNTRKRVRLGIGPGQTFYATGRVIRDIRQAH	37
FJ798399	B	CXCR4	CIRPGNNTSKRISIGPGRAFRATKIIGDIRKAHC	34
AF180903	B	CXCR4	CTRPNNNTRRRIYIGQGRAVYTTKQIVGDIRKAYC	35
AF259052	B	CXCR4	CTRPGNNTRKRISIGPGRAFYTTEQIIGNIRQAHC	35
AJ810483	B	CXCR4	CTRHHEIIKRRKLHIGPGRPFYTAEIGDRRKAYC	34
FJ846645	C	CXCR4	CTRPNNNTRKSIRTGIGRGQTFYVTGQIIGDVRQAH	37
FJ798362	B	CXCR4	CTRPNNNTRKGIHIGLGRRVYVTRQIIGDTKRAHC	35
L31963	B	CXCR4	CTRPNNNTRKKFRIQRGPGRAFVTIGKIGNMRQAHC	36
AF034384	B	CXCR4	CTRPNNYKRKRITTGPGRVLYTTGQIIGDIRRAYC	35
FJ798460	B	CXCR4	CTRPNNNTRRGVYIGPGKAFYTTDRIIGDIRQAHC	35
AF258999	B	CXCR4	CTRPNKTTRKGLRLGPGRAFYTLGGIVGYIRQAHC	35
JN001993	B	CXCR4	CTRPNNNTKRGIYVGPGRKVYTTDRIIGDIRQAHC	35
AY265949	C	CXCR4	CGRPNNHRIKGLRIGPGRAFFAMGAIGGGEIRQAHC	36
U48207	B	CXCR4	CTRPNNNTRRSIPIGPGRAFYATGDIIGDIRQAHC	35
FJ846634	C	CXCR4	CTRPNNNTRKSMRIGIGRGQTFYAMGRIIGDIRQAH	37
AF034378	B	CXCR4	CTRPNNYKKKRITIGPGRVLYTTGQIIGDIRRAYC	35
FJ846655	C	CXCR4	CTRPNNNTRKNVRIGIGRGQTFNAMGRIIGNIRQAH	37

FJ798540	B	CXCR4	CTRPNNNTRGRLSIGPGRAFYATRDIIGDIRRAHC	35
FJ846640	C	CXCR4	CARPGNNTIKRIRIGPRYAFYAKETIIGDIRQAHC	35
AM156922	B	CXCR4	CTRPHNTIKRRIHIGPGRAFYTTKGIQGDLRQAHC	35
FJ846628	C	CXCR4	CTRPDNKINMKRIKIGPGRAFVATKGIRGDIRQAHC	36
FJ653155	B	CXCR4	CKRPNNNARRHIHIGPGRAFYATDIIGNIRQAYC	34
FJ541294	C	CXCR4	CTRPNNNTRKRVRIGHRHLVYAHGEIIGNIRQAHC	35
AY842819	B	CXCR4	CTRPYNLKKSITRGPGRVIYSTGDIMGDIRKAHC	34
DQ869028	B	CXCR4	CTRPGNSTRRGILVGTRRFYTTRNIIGDIRKAHC	34
EF688436	B	CXCR4	CTRPHNKAIRHIHIGQGRAFTTGSIEGNIRQAHC	34
AF033819	B	CXCR4	CTRPNNNTRKRIRIQRGPGRAFVTIGKIGNMRQAHC	36
AF075721	B	CXCR4	CTRPNYNKRKRIHIGPGRAFYTTKNIKGTIRQAHC	35
DQ904343	C	CXCR4	CTRPGNKTIRSIRIGPGRTFYTNKGDIRQAYC	32
AF021669	B	CXCR4	CTRPNNNTRNRIYIGQGRAVYTTKQIVGDIRKAYC	35
FJ653151	B	CXCR4	CKRPNNNMRRHIHIGPGRAFYTTDIIGNMRRAYC	34
KF770430	C	CXCR4	CTRPGNNTGRSVRIGLRTFYTTRKIIGDIRAAHC	34
U04904	B	CXCR4	CTRPNNNTRRSVHSGHIGGGRTLFTTHIVGDIRKAH	37
FJ846657	C	CXCR4	CTRPNNNTRKNVRIGIGRGQTFYANGRIIGNIRQAH	37
AF258985	B	CXCR4	CTRPNKNIRKGLRLGPGRAFYTMGGIVGNIRQTHC	35
AF258988	B	CXCR4	CTRPNKTTRKGLRLGPGRAFYTMGGIVGYIRQAHC	35
FJ798539	B	CXCR4	CTRPNNNTRRRLSIGPGRAFYATRDIIGDIRQAHC	35
AY736821	B	CXCR4	CTRPNNYTRKRITMGPGRVYYTTGEIIGDIRAHC	34
HQ678267	B	CXCR4	CTRPNTKTRKRIHIGPGRAFYTTKTVRDIRQAHC	34
AF021622	B	CXCR4	CTRPNNNTRRRIYIGQGRAVYTTRQIIGDIRKAYC	35
DQ382362	C	CXCR4	CSRPGNNTRKSVRIGIGRGQTFYATGKVIGDIRQAH	37
FJ798402	B	CXCR4	CIRPGNNTSKRVSIGPGRAFRATKVIGDIRKAHC	34
EU578431	B	CXCR4	CTRPNNYTRKHIHLGARKAFYTGEIVGDIRQAHC	34
AF021647	B	CXCR4	CTRPNNNTRKRIYIGQGRAVYTTRQIIGDIRKAYC	35
FJ846630	C	CXCR4	CTRPDNKINMKRIKIGPGRAFVATKGIKGDIRQAYC	36
GU647196	B	CXCR4	CTRPNNNTRKRVSIGPGRAWYTTKQIVGDIRQAHC	35
DQ286958	B	CXCR4	CTRPDNNIKRRIIHIGPGRAFHATKTGDIRQAYC	34
AF034377	B	CXCR4	CTRPNNYKKKRITVGPGRVLYTTGQIIGDIRRAHC	35
FJ798432	B	CXCR4	CTRPNNNTRKRISIGPGRAFYTTRQVIGDIRQAHC	35
AY173956	B	CXCR4	CTRPNNKARRRIRIGPGRTFYTGKIVGDIRQAYC	34
M14100	B	CXCR4	CTRPNNNTRKKIRIQRGPGRAFVTIGKIGNMRQAHC	36
AY230878	C	CXCR4	CMRPGNNTRKRVRIGIGPRQTFYAPGGINKDIRQAH	37
FJ798323	B	CXCR4	CTRPNNNTRKGIHIGLGRRFYVTQIIGDVKRAHC	34
AY842826	B	CXCR4	CARPNNNTRKGIHMGPGRAMYATEKITGDIRQAHC	35
AF189159	B	CXCR4	CTKPNNNTRKRIRIQRGPGRAFVTVGKIGNMRQAHC	36
AF355744	B	CXCR4	CTRPGNKTIRSISLGPGRAFSVTRQIIGDIRKAYC	35
AF259049	B	CXCR4	CTRPGNNTRRRISIGPGRAFYTTEQIIGNIRQAHC	35
FJ798404	B	CXCR4	CIRPGNNTSKRISIGPGRAFRATKVIGDIRKAHC	34
AY189526	B	CXCR4	CTRVSKNIRQKRIKIGPGRAFVATGDIGDIRKAHC	35
AY842833	B	CXCR4	CARPNNNTRKRIYMGTGRYMSATEKITGDIRQARC	35
FJ798570	B	CXCR4	CTRPNNNTRRGIHIGLGRRFYVTQVIGDVKRAHC	34
FJ375983	C	CXCR4	CTRPNRNTKKRITLGPGRVVYTTNEIVGDIRQHC	34
AF021670	B	CXCR4	CTRPNNNTRNRIYIGQGRAVYTTKQIIGDIRKAYC	35
FJ798576	B	CXCR4	CTRPNNNTRKGIHIGPGRAFYATGQIIGDIRQAHC	35
AF355743	B	CXCR4	CTRPGNKTIRSISLGPGRAFSATRQIIGDIRKAYC	35
AY529679	C	CXCR4	CTRPYYNKRRSMRIGIGRGQALYATKEITGDIRRAY	37
DQ286957	B	CXCR4	CQRPNNHTRKRITMSPGRVVYTTGEVIGDIRRAHC	35
FJ541295	C	CXCR4	CGRPNNHRIKGLRIGPGRAFFAMGAIRGGEIRQAHC	36
FJ541290	C	CXCR4	CTRPNNNTRKSVRIGIGRGQTFYATGEIVGDIRQAH	37
AF021630	B	CXCR4	CTRPNNNTRKRIYIGQGRAVYTTKQIVGDIRKAYC	35
KF384805	B	CXCR4	CTRPSNNTRKGIHIGPGRAFFATGDIIGDIRRAHC	35
AF411966	C	CXCR4	CTRPGSNKQRIRNIRIGPGRAFHTNGVIGDIRKAYC	36
KF770413	C	CXCR4	CTRPNINRERNVRIGPGRAFFRTGQMTGDIRQASC	35
KF384801	B	CXCR4	CTRPNNNIRKRIIHIGPGRPFYATGDIGNIRRAQC	35
AF021639	B	CXCR4	CTRPNNNTRKRIYIGQGRAVYTTRQIVGDIRKAYC	35
AY265948	C	CXCR4	CMRPNNNTRKSVRIGPGQTFFATGAIIGNIRQAHC	35
FJ541296	C	CXCR4	CTRPGNKTRQSIRIGIGRGQSFHATGAIIGDIRKAY	37
KF384799	B	CXCR4	CTRHNNNKKIQRIHIGPGRAFVATKGITGDIRQAHC	36
KF384802	B	CXCR4	CTRPNYSTRKSIHIGPGRAFYTTKQIRGNIIQAHC	35
DQ904342	C	CXCR4	CTRPGNKTIRSIRLGPGQAFYTNKGDIRQASC	32
AM156916	B	CXCR4	CTRPNNYTRKGIRIGPGRAVYAAEKIVGNIRQAHC	35
FJ798430	B	CXCR4	CTRPSNNTRRRISIGPGRAFYTTRQVIGDIRQAHC	35
KF384800	B	CXCR4	CTRPYRVITKRIMHIGPGRTFHTTGTIGNIRHAYC	35
L22956	C	CXCR4	CARPGNNTRKSIRIGPGQTFFATGAIIGDIRQAHC	35
FJ653156	B	CXCR4	CKRPNNNMRRHIHIGPGRAFYATDIIGNIRQAYC	34
EU578395	B	CXCR4	CTRPNNNTIKTIRMGIRRAFYTKEIIGDIRQAHC	34
HM215420	B	CXCR4	CTRPNNNTRKRVTLGPGRVWYTTGQIIGDIRKAHC	35

KF766540	C	CXCR4	CTRPYNNTRKSIGIGPGQAFYATGDIIGDIRQAHC	35
AF259009	B	CXCR4	CTRPNKTIRKGLRLGPGRAFYTMGGIEGYIRQAHC	35
KF770415	C	CXCR4	CIRPGNNTRRRVRIGPGQTFYATGNIIGDIRQAHC	35
FJ798527	B	CXCR4	CTRPNNNTRRRISIGPGRAFTTRDIIGDIRQAHC	34
FJ798416	B	CXCR4	CTRPNNNTRQRISIGPGRAFYTTRQVIGDIRQAHC	35
AY173951	B	CXCR4	CTRPNNYTRKRITMGPGRVYYTTGEIIGDIRRAHC	35
FJ653150	B	CXCR4	CKRPNNNMRRHIHIGPGRAFYTTDIIGNIRRAYC	34
KF770411	C	CXCR4	CTRPNINRERKVRIGPGRAFFRTGQMTGDIRQASC	35
FJ798426	B	CXCR4	CTRPNNNTRQRISIGPGRAFYTTRQVVGDIRQAHC	35
AF034375	B	CXCR4	CTRPNNYKRKRITTGPGKVLYTTGQIIGDIRRAHC	35
AF035534	B	CXCR4	CTRPNNNIRKRIHIGPGRAFYTTRQIIGNIRQAHC	35
EF688451	B	CXCR4	CTRPNNKKIEGIRIGPGSAYFTRQIKEHMRQTHC	34
JN001995	B	CXCR4	CTRPNNNTKRGIYVGPGRKVYTTDRIIGNIRQAHC	35
FJ798554	B	CXCR4	CTRPNNNTMKSITIGPGRAFYTTGQIIGDIRQAHC	35
FJ375980	C	CXCR4	CTRPGNNTRKNVIGGRGQTYAHGIGDIRQAHC	32
EU604561	B	CXCR4	CTRPNNHTRKRVTLGPSRVYYTTGEITGDIRRAHC	35
AY529678	C	CXCR4	CARPGNNTRKMMRIGIGRGQTFYANGQVIGDIRQAH	37
AF034376	B	CXCR4	CTRPNNYKKKRITTGPGRVLYTTGQIIGDIRRAYC	35
FJ798356	B	CXCR4	CTRPNNNTRKGIHIGLGRRFYVTQVIGDVKRAHC	34
KF384806	B	CXCR4	CTRPGKKLSRIIHIGPGRAFYASDGRDIRQAYC	33
FJ376006	C	CXCR4	CRRPGNATRKSVRIGIGRGHTFYATGKIIGDIRKAY	37
AF034385	B	CXCR4	CTRPYNYKKKKITTGPGRVLYTTEEIIGDIRRAHC	35
DQ990880	B	CXCR4	CTRPNNNTRKRITMGPGRVLYTTGQIVGDIRKAHC	35
DQ177202	B	CXCR4	CTRPNNNTRKGIRIGPGRAFIATDKIIGDIRQAHC	35
EF688433	B	CXCR4	CIRPNNNTRRSIHIGPGRAFYATGRVIGDVRRAYC	35
DQ382372	C	CXCR4	CTRPANTRIKRLGIGPGQAFRTVKQIIGDIRQSHC	35
