# Supplementary material for: Covariation of Amino Acid Substitutions in the HIV-1 Envelope Glycoprotein gp120 and the Antisense Protein ASP Associated with Coreceptor Usage
Source: Viruses. 2025 Feb 26;17(3):323. doi: 10.3390/v17030323 (PMC11946160; doi:10.3390/v17030323)
Supplement: Supplementary file 1 [file viruses-17-00323-s001.zip › Supplementary File S2.pdf]

Supplementary File S2.

LDA#1

First Linear discriminant analysis (LDA#1) on a training dataset of 1838 V3 sequences with known tropism (1701 R5-tropic and 137 X4-tropic) using the following 7 amino acid properties:

KYTJ820101 Hydropathy index (Kyte-Doolittle, 1982)
KRIW790103 Side chain volume (Krigbaum-Komoriya, 1979)
GOLD730102 Residue volume (Goldsack-Chalifoux, 1973)
DAYM780201 Relative mutability (Dayhoff et al., 1978b)
HUTJ700103 Entropy of formation (Hutchens, 1970)
OOBM850102 Optimized propensity to form reverse turn (Oobatake et al., 1985)
ANDN920101 Alpha-CH chemical shifts (Andersen et al., 1992)

Numerical scale of the 7 amino acid properties.

A.acid	KYTJ820101	KRIW790103	GOLD730102	DAYM780201	HUTJ700103	OOBM850102	ANDN920101
A	1.8	27.5	88.3	100	154.33	1.34	4.35
R	-4.5	105.0	181.2	65	341.01	0.95	4.38
N	-3.5	58.7	125.1	134	207.90	2.49	4.75
D	-3.5	40.0	110.8	106	194.91	3.32	4.76
C	2.5	44.6	112.4	20	219.79	1.07	4.65
Q	-3.5	80.7	148.7	93	235.51	1.49	4.37
E	-3.5	62.0	140.5	102	223.16	2.20	4.29
G	-0.4	0.0	60.0	49	127.90	2.07	3.97
H	-3.2	79.0	152.6	66	242.54	1.27	4.63
I	4.5	93.5	168.5	96	233.21	0.66	3.95
L	3.8	93.5	168.5	40	232.30	0.54	4.17
K	-3.9	100.0	175.6	56	300.46	0.61	4.36
M	1.9	94.1	162.2	94	202.65	0.70	4.52
F	2.8	115.5	189.0	41	204.74	0.80	4.66
P	-1.6	41.9	122.2	56	179.93	2.12	4.44
S	-0.8	29.3	88.7	120	174.06	0.94	4.50
T	-0.7	51.3	118.2	97	205.80	1.09	4.35
W	-0.9	145.5	227.0	18	237.01	-4.65	4.70
Y	-1.3	117.3	193.0	41	229.15	-0.17	4.60
V	4.2	71.5	141.4	74	207.60	1.32	3.95

Input data: a matrix of R5-tropic sequences (1701 rows and 7 columns) and a matrix of X4-tropic sequences (137 rows and 7 columns). The numerical descriptor of each V3 sequence is a vector of seven components, the first is the arithmetic mean of the hydropathy index and the others are the arithmetic mean of the six indices of physicochemical properties. LDA#1 yields a linear function with the following 7 coefficients:

1) 0.4921 for index KYTJ820101, Hydropathy index
2) 7.6455 for index KRIW790103, Side chain volume
3) -7.3117 for index GOLD730102, Residue volume
4) -0.9267 for index DAYM780201, Relative mutability
5) 1.2554 for index HUTJ700103, Entropy of formation
6) 10.3877 for index OOBM850102, Optimized propensity to form reverse turn
7) -33.3877 for index ANDN920101, Alpha-CH chemical shifts

Mean LDA score = -411.18 (sd = 4.50) in 137 X4-tropic V3 sequences
Mean LDA score = -421.81 (sd = 3.21) in 1701 R5-tropic V3 sequences
Cut-off discriminant value =-417.3824

With a cut-off score of -417.38, 1608 out of 1701 sequences R5-tropic (94.5%) are predicted as R5 (score below the cut-off), and 129 out of 137 sequences X4-tropic (94.2%) as X4 (score above the cut-off). The accuracy of LDA#1 is 94.5%. Accuracy is the number of R5-tropic sequences predicted correctly plus the number of X4-tropic sequences predicted correctly, divided by the total number of sequences and multiplied by 100. The percent frequency distribution of the LDA#1 score is shown in Figure 1A of the text.

LDA#2

Second Linear discriminant analysis (LDA#2) on a training dataset of 1838 V3 sequences with known tropism (1701 R5-tropic and 137 X4-tropic) using the following 7 amino acid properties:

KYTJ820101 Hydropathy index (Kyte-Doolittle, 1982)
KRIW790103 Side chain volume (Krigbaum-Komoriya, 1979)
HUTJ700103 Entropy of formation (Hutchens, 1970)
JOND920102 Relative mutability (Jones et al., 1992)
GARJ730101 Partition coefficient (Garel et al., 1973)
FAUJ880104 Length of the side chain (Fauchere et al., 1988)

BIGC670101 Residue volume (Bigelow, 1967)

Numerical scale of the 7 amino acid properties.

A.acid	KYTJ820101	KRIW790103	HUTJ700103	JOND920102	GARJ730101	FAUJ880104	BIGC670101
A	1.8	27.5	154.33	100	0.28	2.87	52.6
R	-4.5	105.0	341.01	83	0.10	7.82	109.1
N	-3.5	58.7	207.90	104	0.25	4.58	75.7
D	-3.5	40.0	194.91	86	0.21	4.74	68.4
C	2.5	44.6	219.79	44	0.28	4.47	68.3
Q	-3.5	80.7	235.51	84	0.35	6.11	89.7
E	-3.5	62.0	223.16	77	0.33	5.97	84.7
G	-0.4	0.0	127.90	50	0.17	2.06	36.3
H	-3.2	79.0	242.54	91	0.21	5.23	91.9
I	4.5	93.5	233.21	103	0.82	4.92	102.0
L	3.8	93.5	232.30	54	1.00	4.92	102.0
K	-3.9	100.0	300.46	72	0.09	6.89	105.1
M	1.9	94.1	202.65	93	0.74	6.36	97.7
F	2.8	115.5	204.74	51	2.18	4.62	113.9
P	-1.6	41.9	179.93	58	0.39	4.11	73.6
S	-0.8	29.3	174.06	117	0.12	3.97	54.9
T	-0.7	51.3	205.80	107	0.21	4.11	71.2
W	-0.9	145.5	237.01	25	5.70	7.68	135.4
Y	-1.3	117.3	229.15	50	1.26	4.73	116.2
V	4.2	71.5	207.60	98	0.60	4.11	85.1

Input data: a matrix of R5-tropic sequences (1701 rows and 7 columns) and a matrix of X4-tropic sequences (137 rows and 7 columns). The numerical descriptor of each V3 sequence is a vector of seven components, the first is the arithmetic mean of the hydropathy index and the others are the arithmetic mean of the six indices of physicochemical properties. LDA#2 yields a linear function with the following 7 coefficients:

1) 9.8573 for index KYTJ820101, Hydropathy index
2) 6.29021 for index KRIW790103, Side chain volume
3) 1.1429 for index HUTJ700103, Entropy of formation
4) -1.4462 for index JOND920102, Relative mutability
5) -34.5005 for index GARJ730101, Partition coefficient
6) 24.8239 for index FAUJ880104, Length of the side chain
7) -11.0246 for index BIGC670101, Residue volume

Mean LDA score = -251.72 (sd = 4.71) in 137 X4-tropic V3 sequences
Mean LDA score = -262.80 (sd = 3.19) in 1701 R5-tropic V3 sequences
Cut-off discriminant value =-258.3288

With a cut-off score of -258.33, 1603 out of 1701 sequences R5-tropic (94.2%) are predicted as R5 (score below the cut-off), and 129 out of 137 sequences X4-tropic (94.2%) as X4 (score above the cut-off). The accuracy of LDA#2 is 94.2%. Accuracy is the number of R5-tropic sequences predicted correctly plus the number of X4-tropic sequences predicted correctly, divided by the total number of sequences and multiplied by 100. The percent frequency distribution of the LDA#2 score is shown in Figure 1B of the text.

LDA#3

Third Linear discriminant analysis (LDA#3) on a training dataset of 1838 V3 sequences with known tropism (1701 R5-tropic and 137 X4-tropic) using the following 7 amino acid properties:

KYTJ820101 Hydropathy index (Kyte-Doolittle, 1982)
KRIW790103 Side chain volume (Krigbaum-Komoriya, 1979)
HUTJ700103 Entropy of formation (Hutchens, 1970)
DAYM780201 Relative mutability (Dayhoff et al., 1978b)
BIGC670101 Residue volume (Bigelow, 1967),
CHAM830105 Number of atoms in the side chain labelled 3+1 (Charton, 1983)
OOBM770102 Short and medium range non-bonded energy per atom (Oobatake-Ooi, 1977)

Numerical scale of the 7 amino acid properties.

A.acid	KYTJ820101	KRIW790103	HUTJ700103	DAYM780201	BIGC670101	CHAM830105	OOBM770102
A	1.8	27.5	154.33	100	52.6	0.0	-1.40
R	-4.5	105.0	341.01	65	109.1	1.0	-0.92
N	-3.5	58.7	207.90	134	75.7	0.0	-1.18
D	-3.5	40.0	194.91	106	68.4	0.0	-1.16
C	2.5	44.6	219.79	20	68.3	0.0	-1.37
Q	-3.5	80.7	235.51	93	89.7	1.0	-1.12
E	-3.5	62.0	223.16	102	84.7	1.0	-1.16

G	-0.4	0.0	127.90	49	36.3	0.0	-1.36
H	-3.2	79.0	242.54	66	91.9	1.0	-1.22
I	4.5	93.5	233.21	96	102.0	0.0	-1.19
L	3.8	93.5	232.30	40	102.0	0.0	-1.32
K	-3.9	100.0	300.46	56	105.1	1.0	-1.07
M	1.9	94.1	202.65	94	97.7	1.0	-1.30
F	2.8	115.5	204.74	41	113.9	1.0	-1.14
P	-1.6	41.9	179.93	56	73.6	0.0	-1.24
S	-0.8	29.3	174.06	120	54.9	0.0	-1.30
T	-0.7	51.3	205.80	97	71.2	0.0	-1.25
W	-0.9	145.5	237.01	18	135.4	1.5	-1.03
Y	-1.3	117.3	229.15	41	116.2	1.0	-1.03
V	4.2	71.5	207.60	74	85.1	0.0	-1.25

Input data: a matrix of R5-tropic sequences (1701 rows and 7 columns) and a matrix of X4-tropic sequences (137 rows and 7 columns). The numerical descriptor of each V3 sequence is a vector of seven components, the first is the arithmetic mean of the hydropathy index and the others are the arithmetic mean of the six indices of physicochemical properties. LDA#3 yields a linear function with the following 7 coefficients:
1) 4.6184 for index KYTJ820101, Hydropathy index
2) 4.9509 for index KRIW790103, Side chain volume
3) 1.3178 for index HUTJ700103, Entropy of formation
4) -0.7525 for index DAYM780201, Relative mutability
5) -8.9141 for index BIGC670101, Residue volume
6) 9.6644 for index CHAM830105, Number of atoms in the side chain labelled 3+1
7) 43.3139 for index OOBM770102, Short and medium range non-bonded energy per atom

Mean LDA score = -218.10 (sd = 4.32) in 137 X4-tropic V3 sequences
Mean LDA score = -228.22 (sd = 3.07) in 1701 R5-tropic V3 sequences
Cut-off discriminant value =-224.0124

With a cut-off score of -224.01, 1610 out of 1701 sequences R5-tropic (94.7%) are predicted as R5 (score below the cut-off), and 129 out of 137 sequences X4-tropic (94.2%) as X4 (score above the cut-off). The accuracy of LDA#3 is 94.6%. Accuracy is the number of R5-tropic sequences predicted correctly plus the number of X4-tropic sequences predicted correctly, divided by the total number of sequences and multiplied by 100. The percent frequency distribution of the LDA#3 score is shown in Figure 1C of the text.
