# Supplementary material for: Covariation of Amino Acid Substitutions in the HIV-1 Envelope Glycoprotein gp120 and the Antisense Protein ASP Associated with Coreceptor Usage
Source: Viruses. 2025 Feb 26;17(3):323. doi: 10.3390/v17030323 (PMC11946160; doi:10.3390/v17030323)
Supplement: Supplementary file 1 [file viruses-17-00323-s001.zip › Supplementary File S3.pdf]

Supplementary File S3

Dataset of 2496 amino acid sequences of the V3 loop domain whose tropism we predicted by using Fisher's linear discriminant analysis: 2261 sequences (1377 of genotype B and 884 of genotype C) were predicted CCR5-tropic (R5) and 235 sequences (180 of genotype B and 55 of genotype C) were predicted CXCR4-tropic (X4).

NCBI ac. Number	Genotype	Tropism predicted	V3 amino acid sequence	length (aa)
KY968401	B	CCR5	CTRPTNNTRKSIHMGWGRAFYATGEIIGDIRQAHC	35
KY968403	B	CCR5	CTRPSNNTRKGIHLAWGRTIYATGEIIGDIRQAHC	35
AY037269	B	CCR5	CTRPNNNTRQGIPIGPLGSFYATTEIIGNIRKAYC	35
KY968394	B	CCR5	CTRPNNNTRKSIHIGPGRAFYATGDIIGDIRQAHC	35
KY968407	B	CCR5	CTRPNNNTRKSIPIGFGRALYATGEIIGDIRQAHC	35
KY968396	B	CCR5	CTRPNNNTRKSINIGPGRAFYATGDIIGDIRQAHC	35
DQ383748	B	CCR5	CTRPHNNTRKSIHIGPGRAFYTTGGIIGDIRQAHC	35
DQ383751	B	CCR5	CTRPNNNTRRSIHIGPGRAFYTTGEIIGDIRDAHC	35
KY658686	B	CCR5	CSRPGNNTRKSIPIGPGRAFFRTGDIIGDIRQAHC	35
KY658683	B	CCR5	CTRPSNNTRKSIHIGPGRTMYATGQIIGDIRQAHC	35
MH078530	B	CCR5	CTRPNNNTRKSIHMGPGAALYATGEIIGDIRQAHC	35
KY658685	B	CCR5	CQRPNNNTRKSINIGPGRAIYATGQIIGNIRQAYC	35
MH078533	B	CCR5	CTRPNNNTRKSIHIGPGRTFYATGDIIGDIRQAHC	35
AY037282	B	CCR5	CTRPNNNTRKSIHIGPGRAFYTTGQVIGDIRQAHC	35
DQ676886	B	CCR5	CTRPNNNTRKSIHMGPGKAFFATGDIIGDIRQAHC	35
AF042101	B	CCR5	CTRPNNNTRKSIHIGPGRAFYATGDIIGNIRQAYC	35
AF538304	B	CCR5	CTRPNNNTRKSIHIAPGRAFYATGEIIGDIRQAYC	35
AF538306	B	CCR5	CTRPNNNTRKSIHIGPGRAFYATGDIIGNIRHAHC	35
AF042104	B	CCR5	CTRPANNTRESIHIGPGRAFYATDIIGDIRQAHC	34
AF042105	B	CCR5	CIRPNNNTRKSMTLGPGKVFYTTGITGDIRKAHC	34
AF538302	B	CCR5	CIRPSNNTRKSINIGPGRAFYATGEIIGDIRQAHC	35
JN002002	B	CCR5	CTRPNNNTRKSIHIAPGRAFYATGEKIGDIRQAHC	35
JN002009	B	CCR5	CTRPHNTRKSIHIGPGRAFYATGDIVGDIREAHC	34
GU367395	B	CCR5	CTRPNNNTRKSIHIGPGKAFYATGEVVGNIRQAFC	35
MN485973	B	CCR5	CVRPNNNTRKSIHMGLGRAFYATGDIIGNIRQAHC	35
MN485981	B	CCR5	CTRPNNNTRKSINIGPGRTFYATGGIIGNIRQAHC	35
MN485993	B	CCR5	CTRLNNNTRRGIHIGPGQAFYTTGDIVGDIRQAHC	35
MN485996	B	CCR5	CTRPNNNTRKSIHMGPGAAFYATGDIIGNIRQAHC	35
MN485998	B	CCR5	CIRPNNNTRRSIHIGPGRAYYAPGEIIGDIRKAHC	35
MN486009	B	CCR5	CTRPNNNTRKSIHIGPGSTFYATGEIIGDIRQAHC	35
MN486013	B	CCR5	CTRPNNNTRQGIHLGPGKAFYVTGDIIGDIRQAHC	35
MN486014	B	CCR5	CTRPGNNTRKSIHMGPGKAFFATGEIIGDIRQAHC	35
MN486022	B	CCR5	CTRPNNNTRKGIHMGPGQAFYATGAIIGDIRQAHC	35
DQ313249	B	CCR5	CTRPNNNTRKSIPIGPGRAFYTTGEIIGDIRQAHC	35
DQ313250	B	CCR5	CTRPSNNTRKSIHMGPGGAIYATGKIIGDIRQAHC	35
DQ313252	B	CCR5	CTRPGNNTRKSIHIGPGRAFYATGEIIGDIRQAHC	35
EU191616	B	CCR5	CTRPNNNTRKGIHIGPGRAFYATTEITGDIRQAHC	35
MH746266	B	CCR5	CTRPNNNTRKSINIGPGRAFYATGAITGNIRQAHC	35
MH746265	B	CCR5	CTRPNNNTRKSIHIGPGRAFYATGDIIGDIRQAHC	35
MH746264	B	CCR5	CIRPSNNTRKSIPIGPGRAFYTTGDIIGDIRQAHC	35
MH746254	B	CCR5	CTRPSNNTSKSIHLGSGRALYATERIIGDTRQAHC	35
DQ358809	B	CCR5	CSRPNNNTRKSIHIGPGRAFYATGDIIGDIRQAHC	35
DQ358810	B	CCR5	CTRPNNNTRRSIHMGPGRAWYTTGEIIGDIRQAYC	35
JN692435	B	CCR5	CTRPNNNTRRSINIGPGRAFYATGAVIGNIRQAHC	35
JN692444	B	CCR5	CSRPNNNTRKSIHIAPGRAFYATGGIIGDIRQAHC	35
JN692445	B	CCR5	CTRPHNNTRKSIRIGPGAAFYATGDIIGDIRQAHC	35
JN692447	B	CCR5	CSRPGNNTRKSIHLGPGSVFYTGEIIGDIRRAYC	34
EF637056	B	CCR5	CTRPNNNTRKSIHFAPGQAFYATGDIIGDIRQAHC	35
EF637054	B	CCR5	CTRPNNNTRKSINMGPGRAFYATGDIIGDIRQAHC	35
EF637051	B	CCR5	CIRPGNNTSKSIHMGPGRAFYATGEIIGDIRQAHC	35
EF637050	B	CCR5	CTRPNNNTRKSIHMGPGRAWFTTGEIIGNIREAHC	35
EF637048	B	CCR5	CTRPNNNTRKSIPMGPGRAMYATGGIVGNIRLAHC	35
EF637046	B	CCR5	CTRPNNNTRKSIHMGWGRAFYATGEIIGNIRQAHC	35
JN692450	B	CCR5	CTRPNNNTRKSIPMGFGRAFYATGDIIGDIRQAHC	35
JN692452	B	CCR5	CTRPNNNTRKGIHIGPGRTFYATGQIIGDIRQAHC	35
JN692453	B	CCR5	CTRPNNNTRRSIPIGPGRALYTTGEIIGDIRQAHC	35
JN692457	B	CCR5	CTRPGNNTRRSISMGPGRAFYTTGDIIGNIRQAHC	35
FJ195090	B	CCR5	CTRPNNNTRKSIHMGWGRAFYATGEIIGDIRQAHC	35
JN692460	B	CCR5	CTRPNNNTQRSINIGPGRAWYTTGAIIGDIRQAYC	35
JN692463	B	CCR5	CTRPGNNTRKSIHIGPGRAFYATGDIIGDIRQAHC	35

JN692475	B	CCR5	CTRPNNNTRKSIHMGPGKSFYATGDIIGDIRQAHC	35
FJ195091	B	CCR5	CTRPNNNTRKSIHMGWGRTFYATGDIIGDIRQAHC	35
FJ195088	B	CCR5	CTRPNNNTRKSIHLGWGRAFYATGEIIGNIRQASC	35
FJ195089	B	CCR5	CIRPHNNTRRSIHMGWGKTFYATGEIIGDIRQAHC	35
JN692479	B	CCR5	CLRPNNNTRKSIHIAPGRAFYATGAIIGDIRRAHC	35
HQ377443	B	CCR5	CTRPNNNTRKSIHIGPGKAFYATGDIIGDIRQAHC	35
HQ377487	B	CCR5	CTRPGNNTRKSIPIGPGRAFYATGDVIGDVRKAHC	35
JN687739	B	CCR5	CTRPSDNTRKSIHMGWGRAFYATGEITGDIRQAHC	35
JF680907	B	CCR5	CTRPNNNTRKSIHIAPGKAFYVTGEIIGDIRQAHC	35
KT427690	B	CCR5	CTRPSNNTRKGIHMGFGRAFYATGEIIGDIRQAHC	35
KT427688	B	CCR5	CTRPGNNTRRSIPLGPGRAFYATGNIIGDIRKAHC	35
KT427686	B	CCR5	CTRPNNNTRKSIHMGPGKAFFATGDIIGDIRQAHC	35
KT427689	B	CCR5	CTRPGNNTRKSIHMGWGRAFYATGDIIGDIRQAHC	35
KT427682	B	CCR5	CTRPHNNTRKSIHLGPGAVLYATGKIIGDIRQAHC	35
KT427680	B	CCR5	CTRPNNNTRKSISIRPGATFYATGDIIGDIRQAYC	35
KT427675	B	CCR5	CTRPNNNTRKSINIGPGRAFYTTGDIIGDIRQAHC	35
KT427745	B	CCR5	CTRPNNNTRKSINMGQGRAWYATGDIIGDIRQAHC	35
KT427740	B	CCR5	CVRPNNNTRKSIHIGPGRAFYATGQIIGDIRKAHC	35
KT427739	B	CCR5	CTRPNNNTRRSIHIGPGRAWYTTGAIIGNIRQAHC	35
KT427738	B	CCR5	CTRPNNNTRKGIHMGPGQTFYATGQIIGDIRKAHC	35
KT427735	B	CCR5	CTRPNNNTIKRIHIGPGRAFYATGQIIGDIRKAHC	35
KT427734	B	CCR5	CTRPGNNTRKSIHIGPGRAFYATGDIIGDIREAHC	35
KT427733	B	CCR5	CTRPNNNTRKSIHIGPGKAFYATGDIIGDIRQAHC	35
KJ849803	B	CCR5	CTRPNNNTRKSINIGPGRAFYATGDITGDIRRAHC	35
KT427730	B	CCR5	CTRPNNNTRKSIHFGPGQALYATGDIIGDIRQAHC	35
KT427728	B	CCR5	CTRPNNNTRKGIHMGPGKTFYATGEIIGDIRQAHC	35
KT427725	B	CCR5	CTRPNNNTRKGIHIGPGRAFYATGEIIGNIRQAHC	35
KT427724	B	CCR5	CTRPGNNTRKSVHMGPGGAIYATGQIIGDIRKAHC	35
KT427723	B	CCR5	CTRPNNNTRKSIPIGPGRAWYATGDIIGDIRQAHC	35
KT427720	B	CCR5	CTRPGNNTSKSINMVGGKFFATGRIIGNIRQAHC	34
KJ849767	B	CCR5	CMRPNNNTRRGIHIGPGRAFYAAGDIIGNIRQAHC	35
KT427847	B	CCR5	CTRPNNNTRKSIHLGSGRAFYATGDIIGDIRQAHC	35
KT427709	B	CCR5	CIRPNNNTRRSITIGPGRAFYGTDIIGDIRQAHC	34
KT427702	B	CCR5	CTRPNNNTRKGIHIGPGRTFYATGEIIGNIRQAHC	35
KT427700	B	CCR5	CTRLSNNTRKSIPIGPGRAFYATGDIIGDIRQAHC	35
KT427692	B	CCR5	CTRPNNNTRKSITIGPGRAWYATGDIIRNIRQAHC	35
KT427792	B	CCR5	CTRPGNNTRRGIHLGPGQAIYATGQIIGDIRQAHC	35
KT427787	B	CCR5	CTRPGNNTRKSIHIGPGKAYYVTGSITGDIRQAHC	35
KT427786	B	CCR5	CTRPGNNTRRSITFGPGKAFYATGNIIGDIRQAHC	35
KT427784	B	CCR5	CTRPGNNTRKSINIGQGGAWYATGAIIGDIRQAHC	35
KT427656	B	CCR5	CIRPGNNTRKSINMGPGRVFYATGQIIGDIRQAHC	35
KT427650	B	CCR5	CTRPNNNTRKSIHMGPGKAFYATGDIIGNIRQAYC	35
KJ849811	B	CCR5	CTRPNNNTRKSIHMGFGRAFYATGEIIGDIRQAHC	35
KJ849825	B	CCR5	CTRPNNNTSKSIHIGQGRAWSATGRIIGDIRQAHC	35
KT427764	B	CCR5	CTRPNNNTRKSIHMGWGRAFYATGEIIGDIRKSHC	35
KT427761	B	CCR5	CTRPNNNTRRGIHVGPGRALYTTDIIGDIRQAHC	34
KT427757	B	CCR5	CTRPGNNTSKGIHFGPGQAIYATERLIGDTRKAHC	35
KT427756	B	CCR5	CTRPSNNTRKSIHFGPGKAFYATDEVIGDIRKAHC	35
KT427755	B	CCR5	CTRPNNNTRKSIHIGPGRAFYATGDIIGDIRQAHC	35
KT427754	B	CCR5	CTRPNNNTSKGIHIGPGRIFYATERIIGDIRQAHC	35
KT427750	B	CCR5	CTRPNNNTRKSIHLGWGRAFYATGEIIGDIRQAYC	35
KT427830	B	CCR5	CTRPNNNTRKSINIGPGRAFYATGDIIGDIRQAHC	35
KT427829	B	CCR5	CTRPNNNTRKSIHIGPGRAFFATDIIGNIRQAHC	34
KT427824	B	CCR5	CVRPNNNTRKSIHIGPGSTYFATGDIIGDIRQAHC	35
KJ849790	B	CCR5	CTRPNNNTRKSIHLGWGRAFYATGEIIGDIRQAHC	35
KT427819	B	CCR5	CTRPNNNTRKGIHMGWGRTFYATGAIIGDIRQAHC	35
KJ849796	B	CCR5	CTRPNNNTRKSIHMGLGRAFYATGDIIGDIRQAHC	35
KT427815	B	CCR5	CTRPNNNTRKSINIGPGAFYTTGSIIGDIRQAYC	34
KJ849808	B	CCR5	CTRPGNNTRRSITIGPGRAFYATGDIIGDIRKAYC	35
KT427799	B	CCR5	CTRPNNNTRKSIHMGWGRAFYATGDIIGDIRQAHC	35
KT427667	B	CCR5	CTRPNNNTRKGIHTAWGRILYATGDIIGDIRQAYC	35
KT427794	B	CCR5	CTRPNNNTRKSIHVAWGRTLYATGDIIGDIRQAHC	35
KU749390	B	CCR5	CTRPNNNTRKSIHIGPGRAFYGTEIIGDIRRAHC	34
KX181906	B	CCR5	CIRPNNNTRKGIHMGPGRTFYARGEIIGNIRSAYC	35
KX181901	B	CCR5	CTRPNNNTRKGIHMGWGKAFYATGEIIGNIRQAHC	35
KX181907	B	CCR5	CTRPNNNTRKSITIGPGAAFFTGEIIGDIRSAYC	34
KX181911	B	CCR5	CTRPNNNTRKSIHMGPGRAFYATGDIIGDIRRAHC	35
KX181897	B	CCR5	CTRPNNNTRKSIHMGWGRAFYATGAIIGDIRQAHC	35
KX181915	B	CCR5	CTRPNNNTRRSINIGPGRAFFTTGQIIGDIRQAYC	35
KX181916	B	CCR5	CIRPSNNTRKSIHIGPGRAFYAIGGIIGDIRQAYC	35

MK041584	B	CCR5	CTRPNNNTRKSIHMGPGKAFYTTGSIIGDIRQAHC	35
MK041586	B	CCR5	CTRPNNNTRKSIHLGWGRAFYASGEIIGDIRQAHC	35
MK041551	B	CCR5	CTRPNNNTSNSISLAGGRALYTTGRLIGNIRQAHC	35
MK041558	B	CCR5	CTRPNNNTRKGIHIGPGRALYTTGEIVGNIRQAHC	35
MK041556	B	CCR5	CTRPNNNTRKSIHLGLGRAFYATGDIIGDIRQAHC	35
MK041586	B	CCR5	CTRPNNNTRKSIHLGWGRAFYASGEIIGDIRQAHC	35
MG571999	B	CCR5	CTRPNNNTRKSIHMGMGRAFYATGEIIGDIRQAHC	35
MG572006	B	CCR5	CTRPNNNTSKSIHLGPGRTFYATGSIIGDIRKAYC	35
AY779552	B	CCR5	CTRPNNNTRRSINIGPGRAFYTTGDIIGDIRKAHC	35
GU562135	B	CCR5	CTRPNNNTRKSIPMGPGRAFYATEDIIGNIRQAHC	35
GU562272	B	CCR5	CTRPSNNTRKGIHIGPGSAFYTTGEIIGDIRQAHC	35
GU562155	B	CCR5	CTRPNNNTRKSIHIGPGKAFYTTDIIGDIRQAHC	34
JF320413	B	CCR5	CMRPNNNTRKSITIGPGRAFYTTGQIIGDIRQAHC	35
GU562266	B	CCR5	CTRPNNNTRRSIPIGPGRAWFATGDITGDIRKAYC	35
AY314061	B	CCR5	CTRPNNNTRKSIHIQPGRAFYATGEIIGDIRQAHC	35
GU562033	B	CCR5	CTRPNNKTRKSIPMGPGRAFYATGDIIGDIRQAHC	35
GU562058	B	CCR5	CTRPNNNTRKSIHIAPGSAFYATGAIIGDIRQAHC	35
GU562001	B	CCR5	CTRPNNNTRQGIHMGPGRSFYTAGAIIGDIRQAHC	35
GU562080	B	CCR5	CIRPNNNTRKSIHIGPGRAFYTTGEIIGDIRQAHC	35
DQ322227	B	CCR5	CTRPNNNTRKSITMGPGKAFFTTGDIIGDIRQAHC	35
AY857144	B	CCR5	CTRPNNNTRKSIPIGPGRAFYATGDIIGDIRQAHC	35
DQ322239	B	CCR5	CTRPNNNTRRDIHIGPGRAFYAGEIIGNIRKAHC	34
JQ403022	B	CCR5	CTRPNNNTRKSIHLGPGRAIYATGEIIGNIRKAYC	35
JQ403023	B	CCR5	CTRPNNNTRKGIHIGPGRAFYATGDIIGDIRQAHC	35
KC797171	B	CCR5	CTRPNNNTLKSIHIGPGRAFYTTGQIIGDIRQAHC	35
JQ403025	B	CCR5	CTRPNNNTRKSINMGPGRAFYATGDIIGDIRQAHC	35
JQ403026	B	CCR5	CTRPNNNTRKSIPIGPGRAFYTTGDIIGDIRQAHC	35
EU023916	B	CCR5	CTRPSNNTRKSITIGPGRAFYATGEIIGDIRKAHC	35
JQ403019	B	CCR5	CTRPNNNTRKSVRIGPGQALYTTDIIGDIRQAHC	34
JQ403029	B	CCR5	CTRPNNNTRKSIHIGPGRAFYTTGQIIGDIRQAHC	35
JQ403044	B	CCR5	CTRPNNNTRKSITIGPGAAFFATGGIIGDIRKAYC	35
KX792621	B	CCR5	CTRPNNNTRKGIHIGPGRAFYATGSITGDIRKAHC	35
JQ403043	B	CCR5	CTRPNNNTRKSIHIGLGRTFYATGDIIGDIRQAHC	35
JQ403045	B	CCR5	CARPNNNTRKGIHIGPGRAFYTTGEIIGDIRQAHC	35
JQ403046	B	CCR5	CTRPNNNTRRSIQIGPGRSFFATGDIIGDIRRAHC	35
EU023919	B	CCR5	CTRLNNNTRRSINIGPGRAWYTTGEVIGDIRKANC	35
EU023922	B	CCR5	CTRPNNNTRRSIPIGPGRAFYATDIIGDIRQAHC	34
KX792557	B	CCR5	CTRPNNNTRRSIHIAPGRAFYATGEIIGNIKKAHC	35
KX792831	B	CCR5	CIRPNNNTRRSVPIGPGRAIYATGQIIGDIRQAHC	35
JQ403042	B	CCR5	CTRPNNNTRKSIHIGPGRAFYATGDIIGDIRQAHC	35
KX792688	B	CCR5	CTRPNNNTRKSIQLGPGAAFYATGDIIGDIRQAHC	35
KX792771	B	CCR5	CTRPSNNTRKGIHIGPGRAFYTTGSIIGDIRQAHC	35
KX792725	B	CCR5	CTRPNNNTRKSITIGPGRAFYATGQVIGDIRKAHC	35
KX792808	B	CCR5	CTRPSNNTRKSINVGPGRAIYTTGQIIGDIRQAHC	35
EU023929	B	CCR5	CTRPNNNTRKSIRIGPGSAFYTTGEIIGDIRKAHC	35
EU023933	B	CCR5	CTRPNNNTRKSIPIGPGRAFYATGDIIGDIRQAHC	35
EU023927	B	CCR5	CTRPNNNTRRSISIGPGRAFYATGEVIGDIRQAHC	35
MH714351	B	CCR5	CTRPNNNTRKSINIGPGRAFYAAGDIIGDIRQAHC	35
MH714324	B	CCR5	CTRPNNNTRKSINIGPGRAFYATGDIIGDIRQAYC	35
MH714343	B	CCR5	CTRPNNNTRKSLNIGPGRAVYATGEIIGDIRQAHC	35
MH714329	B	CCR5	CVRPNNNTRRGINMGPGRAFYTMGDIIGDIRQAHC	35
KU600814	B	CCR5	CIRPGNNTRKSITIGPGRAFYTTGEVIGNIRQAHC	35
KU600818	B	CCR5	CTRPGNNTRRSIHIGPGRAFYTGEIIGDIRQAHC	34
KU600815	B	CCR5	CTRPGNNIRKSIPIGPGRAFFATGDIIGDIRQAHC	35
EU363827	B	CCR5	CTRPSNNTRKSINIGPGRAWYATGQIIGDIRQAHC	35
AY905493	B	CCR5	CIRPNNNTRKSIHMGPGQAWFATGDIIGDIRQAHC	35
AY905497	B	CCR5	CTRPNNNTRKSINIGPGQAWYATGQIIGDIRQAHC	35
AY905495	B	CCR5	CTRPNNNTRKSIHLGPGQAWYTTGEIIGDIRQAHC	35
HM215397	B	CCR5	CTRPNNNTRKSISLGPGRAWYATGQIIGNIRQAHC	35
HQ699996	B	CCR5	CTRPNNNTRKSINLGPGQAWYTTGQIIGDIRQAHC	35
EU131790	B	CCR5	CTRPNNNTRKSINIGPGRSWYTTGQIIGDIGQAHC	35
EU131788	B	CCR5	CTRPNNNTRKSIHLGPGHAWHTTERIIGNIRQAHC	35
EU131789	B	CCR5	CTRPNNNTRKSIHLGPGQALYATGQIIGDIRQAHC	35
MK053935	B	CCR5	CTRPNNNTRKSIHLGPGRAWYATGEIIGDIRQAHC	35
MF591612	B	CCR5	CTRPNNNTRKSIHLGPGRAWYATGEIIGNIRQAHC	35
JF932482	B	CCR5	CTRPNNNTRKSIHLGPGQAWYTTGQIIGDIRQAHC	35
HQ699949	B	CCR5	CIRPNNNTRKSIPLGPGKAWYTTGEIIGDIRKAHC	35
HM215423	B	CCR5	CTRPNNNTLKSIQLGLGRAWHATGQIIGDIRQAHC	35
EU131805	B	CCR5	CTRPNNNTRKSISIGPGQAMYATGQIIGDIRQAHC	35
EU131809	B	CCR5	CIRPSNNTRKSIPMGPGRAIYATGEIIGDIRQAHC	35

JF932468	B	CCR5	CTRPNNNTRKSISLGPGRAWYTTGEIIGDIRQAHC	35
JF932470	B	CCR5	CTRPNNNTRKSINIGPGRAWYTTGQIIGDIRQAHC	35
JF932471	B	CCR5	CERTNNNTRKSIPLGPGRAWYTTGQVIGNIRQASC	35
JF932473	B	CCR5	CIRPNNNTRKSISLGPGKAWYTTGQIIGDIRQAHC	35
KM217584	B	CCR5	CTRPTNNTRKSIHIGPGSSLYATGAIIGDIRQAHC	35
KM217662	B	CCR5	CSRPNNNTRKSINFGPGGAIYTTGDIIGDIRQAYC	35
KM217802	B	CCR5	CTRPNNNTRKGIHFGPGGPLFTTDIIGDIRRAHC	34
KM217995	B	CCR5	CTRPSNNTRKSIHIGWGRSLYATGDITGDIRQAHC	35
JF932477	B	CCR5	CTRPNNNTRKSIHLGQGRAWYTTGEIIGDIRQAHC	35
JF932483	B	CCR5	CTRPNNNTRKSIHLGPGRAWYATGQIIGDIRQAHC	35
JF932484	B	CCR5	CTRPNNNTRKSINIGPGRAWYATGEIIGNIRQAYC	35
JF932485	B	CCR5	CIRPNNNTRKSIHLGPGQAWYTTGQIIGDIRQAHC	35
JF932486	B	CCR5	CTRPNNNTRKSISMGPGRAVYATGQIIGDIRQAHC	35
MH479957	B	CCR5	CIRPNNNTRKSIHLGPGRALYTTGIIGDIRQAHC	34
EU131798	B	CCR5	CTRPNNNTRKSIHLGPGQAWYTTGQIIGNIRQAHC	35
JF932490	B	CCR5	CTRPNNNTRKSINIGPGQAWYTTGQIIGDIRQAHC	35
JF932491	B	CCR5	CTRPNNNTRKSITLGPGRVWYTTGQIIGDIRQAHC	35
JF932500	B	CCR5	CTRPNNNTRKSIPLGPGRAWFATGQIIGDIRQAHC	35
KM218168	B	CCR5	CTRPNNNTRKSIHIGPGRSFYATGDIIGDIRQAHC	35
KM218228	B	CCR5	CIRPNNNTRKSIHISPGRAFYATGDIIGDIRQAHC	35
JF932480	B	CCR5	CTRPNNNTRKSIPLGPGKAWYTTGQIIGDIRQAHC	35
JF932481	B	CCR5	IPLGQGRAWYTTGQIIGDIRQAHC	24
JF346919	B	CCR5	CTRPNNNTRKSIPLGQGRAWYTTENIIGDIRQAHC	35
JX960597	B	CCR5	CIRPNNNTRKSIPIGPGRAFYATGEITGDIRQAHC	35
JX960598	B	CCR5	CIRPNNNTRKSINIGPGRAFYATGEIIGDIRKANC	35
KC899011	B	CCR5	CTRPNNNTRKSIPLGPGKAWYTTGQIIGDIRKAHC	35
JF932494	B	CCR5	CTRPNNNTRKSIHIGPGRAWYATGNIIGDIRQARC	35
JX140658	B	CCR5	CTRPSNNTRKGISLGQGGVFYTTGDIIGNIRQAHC	35
KX692929	B	CCR5	CTRPNNNTRKSINIGPGRAWYTTGQIIGSIRQAHC	35
KX692941	B	CCR5	CTRPNNNTRKSINIGPGRAWYTTGEIIGNIRQAHC	35
KX692973	B	CCR5	CTRPNNNTRKSIPLGLGRAWYATGQIIGDIRQAHC	35
KX693001	B	CCR5	CTRPNNNTRKSISLGPGQAWYTTGEIIGNIRQAHC	35
KX693029	B	CCR5	CTRPNNNTRKSIPLGPGQAWYTTGDIIGNIRQAHC	35
KX693575	B	CCR5	CTRPNNNTRKSISLGPGQAWFATGQIIGDIRQAHC	35
KX693068	B	CCR5	CTRPNNNTRKSIQLGQGRAWYTTGEIIGDIRQAHC	35
KX693114	B	CCR5	CTRPNNNTRKSINIGPGRAWYTTGEIIGNIRQAHC	35
KX693689	B	CCR5	CTRPNNNTRKSIHLGQGRAWYATGEIIGDIRQAHC	35
KX693720	B	CCR5	CERPNNNTRKSIHLGPGRAWYATGQIIGNIRQAHC	35
KX693770	B	CCR5	CTRPNNNTRKSINLGPGRAWYATGQIIGDIRQAHC	35
KX693223	B	CCR5	CTRPNNNTRKSIPIGPGRAWYATGEIIGDIRQAHC	35
KX693238	B	CCR5	CTRPNNNTRKSIPLGPGRAWYTSGQIIGDIRKAHC	35
KX693247	B	CCR5	CTRPNNNTSKSIHLGPGQAWYTTGRIIGDIRQAHC	35
KX693268	B	CCR5	CTRPNNNTRKSISLGPGQAWYAAGEIIGDIRQAHC	35
KX693886	B	CCR5	CTRPNNNTRKSIPLGQGRAWYTTGDIIGDIRQAHC	35
KX693314	B	CCR5	CTRPNNNTRKSIQLGPGKAWYTTGQIIGEIRQAHC	35
KX693339	B	CCR5	CTRPNNNTRKGIPIGPGRAWYTTKDIIGDIRQAHC	35
KX693374	B	CCR5	CTRPNNNTRKSIPLGPGRAWYATGQIVGDIRQAHC	35
KX693390	B	CCR5	CTRPNNNTRKSIPLGPGRAWFATGEVIGDIRRAHC	35
KX693437	B	CCR5	CTRPNNNTRKGIHIGPGQALYATGEIIGDIRQAHC	35
KX693455	B	CCR5	CIRPNNNTRKSIPLGQGRAWYTTGQIIGDIRQAHC	35
KX693469	B	CCR5	CTRPNNNTRKSINIGPGQAWYTTGQIIGDIRQAHC	35
KX693475	B	CCR5	CTRPNNNTRKSVSLGPGQAWFTTGQIIGDIRKAHC	35
KX693489	B	CCR5	CTRPNNNTRKSIHLGPGQAWYATGQIIGDIRQAHC	35
KX693491	B	CCR5	CTRPNNNTRKSITIGPGRTWYTTGQIIGDIRQAHC	35
KX693496	B	CCR5	CTRPNNNTRKSIPLGPGKAWYTTGQIIGDIRQAHC	35
JF932495	B	CCR5	CTRPNNNTRKSIPLGPGRAWYTTGEIIGDIRQAHC	35
JF932496	B	CCR5	CTRPNNNTRKSINLGLGRAWFTTGQIIGDIRQAHC	35
EU363830	B	CCR5	CTRPNNNTRKGIHIGPGKTWYATGQIIGDIRQAYC	35
AY561236	B	CCR5	CTRPNNNTRKGIHLGPGKAFYASGDIVGNIRQAHC	35
HQ377377	B	CCR5	CTRPNNNTRKGIHIGPGRAFYTTGDIIGDIRQAHC	35
AY586542	B	CCR5	CTRPTNNTRKGIHIGPGRAVYATEEIIGDIRQAHC	35
EU668963	B	CCR5	CVRPGNNTRKSIHIAPGRAFYATGNIIGDIRQAHC	35
FJ388890	B	CCR5	CTRPNNNTRRSIHIGPGRAFYATGEITGDIRAAHC	35
FJ388898	B	CCR5	CTRPSNNTRKSIHLGPGSAFYTTGQIIGDIRRAHC	35
FJ388905	B	CCR5	CTRPNNNTRKSIHIAPGRAFYATDDVIGDIRRAHC	35
FJ388910	B	CCR5	CTRPNNNTRKGVHMGPGKVFYTTDIIGDIRQAHC	34
FJ388912	B	CCR5	CTRPNNNTRKSIHIGPGRAFYATGDIIGDIRQAHC	35
FJ388960	B	CCR5	CTRPNNNTRKSINIGPGRAFYATGEIIGDIRQAHC	35
FJ388914	B	CCR5	CTRPHNNTRRSINIGPGRAFYATGQIVGDIRQAHC	35
FJ388915	B	CCR5	CMRPNNNTRKSIPIGPGRAFYATGDIIGDIRQAHC	35

FJ388918	B	CCR5	CTRPSNNTRRSIHIGPGRAFYATDQIIGDIRQAHC	35
FJ388920	B	CCR5	CTRPNNNTRRSIHIAPGRAFYGTDIIGDIRQAHC	34
FJ388923	B	CCR5	CTRPNNNTRKSISIGPGRAFYATGEIIGNIRQAHC	35
FJ388963	B	CCR5	CIRPNNNTRKSIQIGPGRALYTTGEIIGDIRQAHC	35
FJ388924	B	CCR5	CTRPNNNTRRSINIGPGRAFYATGEIIGDIRQAHC	35
FJ388964	B	CCR5	CTRPNNNTRKGIHMGPGAFYATGKIIGDIRKAHC	34
FJ388927	B	CCR5	CTRPNNNTRKSIHIGPGRAFYATGQIIGDIRQAHC	35
FJ388933	B	CCR5	CTRPNNNTIKSIHMGPGKAFYTTGQIIGNIRQAHC	35
FJ388935	B	CCR5	CIRPNNNTRKSIRIGPGAAFYATDIIGDIRQAHC	34
FJ388936	B	CCR5	CTRPNNNTRRSIHMGPGKAYYATGEIIGDIREAHC	35
FJ388937	B	CCR5	CTRPNNNTRKSIHIAPGRAFYATGGIIGDIRQAYC	35
FJ388939	B	CCR5	CTRPNNNTRKSISIGPGRAFYAHGEIIGDIRKASC	35
FJ388955	B	CCR5	CTRPSNNTRKSISIGPGRAFYTTGEIIGDIRQAHC	35
JF683736	B	CCR5	CTRPNNNTRKSITIGPGRAFYATGDIIGDIRRAHC	35
JF683741	B	CCR5	CTRPGNNTRKSISIGPGRAFYATGDIIGDIRQAHC	35
JF683743	B	CCR5	CTRPNNNTRKSIPMGPGKFYTTGDIIGDIRKAYC	34
JF683747	B	CCR5	CTRPNNNTRKGITIGPGRAFYTTGEIIGNIRQAYC	35
JF683751	B	CCR5	CERPNNNTRKSVHIGPGRAFYATGDIIGNIRQAHC	35
JF683753	B	CCR5	CTRPGNNTRRSIPIGPGKAFYATGDIIGNIRQAHC	35
JF683754	B	CCR5	CTRPNNNTRKGVHIGPGSAIYATGNIIGDIRSAHC	35
JF683769	B	CCR5	CIRPNNNTRKSIHIGPGRAFYATGDIIGDIRQAHC	35
JF683775	B	CCR5	CTRPGNNTRRSIRIGPGSAFFETGDIIGDIRQAHC	35
JF683778	B	CCR5	CTRPNNNTRKSIHIAPGRAFYATGEIIGDIRKAHC	35
JF683785	B	CCR5	CLRPNNNTRKSVPIVPGGAFYATGDITGDIRKAHC	35
JF683787	B	CCR5	CTRPNNNTRKGIHIGPGRAFYATGQVIGDIRQAHC	35
JF683788	B	CCR5	CTRPNNNTRKSITIGPGRAFYATGDIIGDIRKAHC	35
JF683790	B	CCR5	CTRPSNNTRRSIHIGPGRAFYATGQITGDIRSAYC	35
JF683794	B	CCR5	CIRPNNNTRKSIHIAPGRAFYTTGQIIGDIRQAHC	35
JF683801	B	CCR5	CTRPNNNTRKGIHMGWGRAFYATGEIVGDIRQAHC	35
JQ403048	B	CCR5	CTRPNNNTRKSIHIAPGRAFYATGEIIGNIRQAHC	35
KT124812	B	CCR5	CTRPNNNTSKSIPIGPGGAFYTTGSIIGDIRQAHC	35
KT124752	B	CCR5	CTRPNNNTRKGIHIAPGRAFYATGDIIGDIRKAHC	35
KT124755	B	CCR5	CIRPNNNTRKSINIGPGRAFYATGEIIGDIRQAHC	35
KT124757	B	CCR5	CTRPNNNTKKSVNIGPGRAFDATGKIIGDIRQAHC	35
KT124794	B	CCR5	CTRPNNNTRKSINIGPGRAFYATDIIGDIRQAYC	34
KT124800	B	CCR5	CTRPNNNTRKSIHIAPGRAFYATGQIIGDIRKAHC	35
KT124810	B	CCR5	CTGPNNNTRKSIHIGPGKTFYATGEIIGDIRQAHC	35
KT124749	B	CCR5	CTRPNNNTRKSIHIGPGRAFYATGDIIGDIRQAHC	35
KT124753	B	CCR5	CTRPNNNTRKSIPIGPGRALYTTEIIGDIRKAYC	34
KT124754	B	CCR5	CTRPYNNTRKGIHMGPGRALYITGEVTGNIRQAHC	35
KC247955	B	CCR5	CIRPNNNTRKSIPIGPGKVFYATGEIIGDIRQAHC	35
KT124772	B	CCR5	CTRPNNNTRKSIPIGPGRAMFTAGEIIGDIRKAYC	35
KT124797	B	CCR5	CTRPGNNTRKSIHIGPGRAFYATDIIGDIRQAHC	34
KC248026	B	CCR5	CTRPNNNTRKGIHIGPGRTFYATGAIIGDIRQAHC	35
KY968394	B	CCR5	CTRPNNNTRKSIHIGPGRAFYATGDIIGDIRQAHC	35
KT124765	B	CCR5	CTRPNNNTRRSITMGPGKAFFGTEVIGDIRQAHC	34
KT124811	B	CCR5	CTRPGNNTRKGIHMGPGRVFYATGEITGDIRKAHC	35
KT124767	B	CCR5	CVRPGNNTRKSIHIGPGRAFYATGEIIGNIRQAHC	35
KY324301	B	CCR5	CTRPNNNTRKSIHIGPGRAFYATGEVIGDIRQAHC	35
KY324379	B	CCR5	CIKNNNNTRKSINIGPGRAWYTTGQIIGEIRQAYC	35
KY324630	B	CCR5	CTRPNNNTRKSINIGPGRAFYATGDIIGDIRQAHC	35
KY324641	B	CCR5	CTRPSNNTRKSINIGPGRAFYATGDIIGDIRQAHC	35
KY324761	B	CCR5	CIRPNNNTRKSINIGPGRAFYATGAIIGNIRQAHC	35
KY324808	B	CCR5	CTRPGNNTRKSIHIAPGRAFYATGNIIGEIRQAHC	35
MK115683	B	CCR5	CTRPNNNTRKSIHIGPGRAWYATGDIIGDIRKAYC	35
MK115918	B	CCR5	CTRPSNNTRKGIHTGPGSVIYATGEIIGDIRQAHC	35
MK116058	B	CCR5	CTRPNNNTRRGIHIGPGRAFYAASDIIGNIRQAHC	35
MK116212	B	CCR5	CIRPNNNTRKSIPAGPGKVIYATGEIIGDIRQAHC	35
MN090868	B	CCR5	CTRPNNNTRKSIHAGPGKVIYATGEIIGDIRQAHC	35
AF490512	B	CCR5	CTRPNNNTRRSIHIGPGRAIYATGDIIGDIRQAHC	35
EF514704	B	CCR5	CTRPGNNTRKSIHIGPGRAFYTTGDIIGDIRKAYC	35
EF514705	B	CCR5	CTRPNNNTRKGIHIGPGRAFYATGEIIGDIRQAHC	35
EF514706	B	CCR5	CTRPSNNTRKSIHMGPGGAFYATGAIIGDIRQAHC	35
EF514707	B	CCR5	CTRPSNNTRRSIHIAPGRAFYTTGAIIGDIRQAHC	35
EF514709	B	CCR5	CTRPNNNTRKSVHIGPGRAFYATGDIIGNIRQAHC	35
EF514710	B	CCR5	CIRPNNNTRKSIPIGPGRAFYTTGDIIGDIRHAYC	35
EF514711	B	CCR5	CTRPNNNTRKSIPMGPGKAFYTTGQIIGDIRQAHC	35
EF514697	B	CCR5	CTRPNNNTRRGIHIGPGRAFYTTGDVIGDIRQAHC	35
EU839597	B	CCR5	CTRPGNNTRKGIHIGPGSTWYTTGEIVGDIRQAHC	35
FJ817365	B	CCR5	CTRPNNNTRKGIHMGSGRAFYTTGDIIGDIRKAHC	35

KY658702	B	CCR5	CTRPGNNTRKSVHIGPGRAFYATGEIIGDIRQAHC	35
AY173960	B	CCR5	CTRPSNNTSKSIQLVGGSALYATNRIIGDIRRAYC	35
KC595150	B	CCR5	CTRPNNNTRKSINIGPGRAFYTTGDIIGDIRQAHC	35
KC595152	B	CCR5	CTRPNNNTRRSINIGPGRAFYATGKIIGDIRQAHC	35
KC595154	B	CCR5	CTRPSNNTRKSIHIGPGRAFYATGEITGDIRRAYC	35
KC595157	B	CCR5	CTRPNNNTRKGIHIGPGSAFYATGAIIGDIRQAHC	35
KC595160	B	CCR5	CTRPNNNTRKSIHIGPGRAFYTTGEIIGDIRQAHC	35
KC595161	B	CCR5	CTRPNNNTRRSINIGPGRAFYATGAIIGNIRQAHC	35
KC595162	B	CCR5	CTRPNNNTRKSINIGPGRAFYATGEIIGNIRQAHC	35
KC595163	B	CCR5	CTRPSNNTRRSIHMGPGRAFYTTGDIIGDIRQAHC	35
KC595165	B	CCR5	CTRPGNNTRKSIHMGPGRAWYATGDIIGDIRQAHC	35
KC595197	B	CCR5	CTRPNNNTRKSIHIGPGKAFYAAREIIGDIRQAFC	35
KC595202	B	CCR5	CTRPNNNTRKSIPIGPGRTFYATGDIIGDIRQAHC	35
KC595203	B	CCR5	CTRPNNNTRKGIHMGPGKVFYTTGEIIGDIRQAHC	35
KC595158	B	CCR5	CTRPNNNTRRGIHIGPGRAFYATDIIGDIRQAHC	34
AY857144	B	CCR5	CTRPNNNTRKSIPIGPGRAFYATGDIIGDIRQAHC	35
KC595190	B	CCR5	CTRPNNNTRKSIHIGPGKAFYATGEIIGDIRQAYC	35
KT200351	B	CCR5	CTRPNNNTRKGIHIGPGKTWYATGEIIGDIRQAHC	35
EU786676	B	CCR5	CTRPNNNTRKSITIGPGRAFYATGDVIGDIRQAHC	35
KC595196	B	CCR5	CTRPNNNTRKSIPIGPGRAFYTTGAIIGDIRQAHC	35
EU786678	B	CCR5	CIRPNNNTRKSINIGPGRAFYTTGAITGDIRQAHC	35
EU786680	B	CCR5	CTRPNNNTRKSIHIGPGRALYAQDIIGDIRQAHC	34
FJ817366	B	CCR5	CTRPNNNTRRSIPIGPGRTFYATGDIIGDIRKAYC	35
FJ670531	B	CCR5	CIRPNNNTRKSIHMGPGRAFYATGDVIGDIRKAYC	35
FJ853620	B	CCR5	CIRPNNNTRKSINIGPGRAFYAAGEIIGDIRQAHC	35
KT200349	B	CCR5	CTRPNNNTRRSIHIGPGSAFYTSNIIGDIRQAHC	34
KT200354	B	CCR5	CTRPNNNTRKSIPIGPGRAFYATDKIIGDIRKAYC	35
KT200358	B	CCR5	CTRPSNNTRKGIHIGPGRAFYATDIIGDIRQAHC	34
FJ853622	B	CCR5	CTRPNNNTRKSIHLGAGKAIYTTGAIIGNIRQAHC	35
GU362883	B	CCR5	CTRPNNNTRRSIHIAPGRAFYATGEIIGDIRQAHC	35
GU362886	B	CCR5	CTRPNNNTRKGIHIGPGKTFFATDIIGDIRQAHC	34
JX140656	B	CCR5	CTRPNNNTRKGIQMGPGRAFYATGDIIGDIRQAHC	35
GU362881	B	CCR5	CTRPNNNTRKGIHIGPGRTLYATGEIIGDIRKAHC	35
KC473842	B	CCR5	CTRPGNNTRKSISLGPGRVFYATGDIIGDIRQAYC	35
KT200353	B	CCR5	CTRPNNNTRKSIHIGPGRAFYATGDIIGDIRQAHC	35
KT200355	B	CCR5	CVRPGNNTRRSIHIGPGKAFYTTGEIIGDIRQAHC	35
KT200356	B	CCR5	CTRPNNNTRKSINIGPGRAFYATGAIIGDIRQAYC	35
KY465967	B	CCR5	CIRPSNNTRKSISIGPGRAFYATGDIIGDIRQAHC	35
KT276263	B	CCR5	CTRPNNNTRKSVPIGPGAAIYATGAIIGDIRQAHC	35
KT276266	B	CCR5	CMRPNNNTRKSVSLGPGRAIYATGEVGDIRQAHC	34
KT276267	B	CCR5	CTRPNNNTRKSIHLGPGKTFYATGAIIGDIRQAHC	35
KU685585	B	CCR5	CTRPGNNTRKGFHIGPGQTLYATGQIIGDIRQAHC	35
KU685589	B	CCR5	CTRPGNNTRKSIHIAPGRALYATGDIIGDIRQAHC	35
KY989949	B	CCR5	CTRPNNNTRKSIHIGPGRAFYATGDIIGDIRQAHC	35
KY989954	B	CCR5	CTRPNNNTSKSIPIGPGRAFYATGRVIGDIRQAHC	35
KY989956	B	CCR5	CVRPNNNTRKSVHMGPGSVWYATGDIIGDIRQAHC	35
KC595171	B	CCR5	CIRPNNNTRKSIHIGPGRALYTTEVVGNIRQAYC	34
KC595174	B	CCR5	CTRPNNNTRTSIPLGPGRAFYTTGDIIGDIRKAYC	35
KC595177	B	CCR5	CTRPNNNTRKSIHIGPGSTLYATEIIGDIRQAHC	34
KC595178	B	CCR5	CTRPNNNTRKSIHIGPGRAFYTTGEIIGDIRQAHC	35
KC595195	B	CCR5	CTRPNNNTRRSIHLGPGGAFYAAGEIIGDIRQAHC	35
KC595182	B	CCR5	CTRPNNNTRKSITIGPGRAFYATGEIIGDIRQAHC	35
KC595159	B	CCR5	CTRPNNNTRKSIHIGPGRAFYTTGEIIGDIRQAHC	35
KC595225	B	CCR5	CTRPNNNTRKSINIGPGRAFYAHGEIIGDIRQAHC	35
KC595206	B	CCR5	CTRPSNNTRKGIHIGPGRAFYATGEIIGDIRQAHC	35
DQ141341	B	CCR5	CTRPGNNTRRSIHIGPGRAFYTTGQIIGDIRQAHC	35
EF531330	B	CCR5	CTRPNNNTRRGIHIGPGRAFYATGIIGDIRQAHC	34
EF531333	B	CCR5	CTRPNNNTRKGIHMGPGRAFYATGDIIGNIRQAHC	35
JX422195	B	CCR5	CTRPNNNTRRSIHIGPGRAFYTTGSIIGDIRQAYC	35
AY535455	B	CCR5	CTRPNNNTRRGIHLGPGRAFYATGDIIGDIRQAHC	35
KC699018	B	CCR5	CTRPSNNTRKSISIGPGRAFYATGDIIGDIRQAHC	35
KU168261	B	CCR5	CTRPNNNTRRSIRIGPGSTLYAEGDIIGDIRQAHC	35
JX140652	B	CCR5	CTRPSNNTRKSIHIGPGRAFYATGEIIGNIRQAHC	35
KC699029	B	CCR5	CTRPGNNTRRSIHFGPGKAWYTGEIIGDIRQAHC	34
KC699030	B	CCR5	CIRPNNNTRKGINIGPGRAWYATTDIIGDIRKAHC	35
KC699028	B	CCR5	CMRPNNNTRKGIHIGPGGAIYATGAIIGNIRQAHC	35
KC699039	B	CCR5	CVRPNNNTRKSINMGPGRAFYTMGDIIGNIRQAHC	35
KC699035	B	CCR5	CTRPNNNTRKSIHLGLGKAFYATDIIGDIRQAHC	34
KC699036	B	CCR5	CTRPNNNTRRSIHIGPGRAFYATQDIIGNIRQAYC	35
KF716494	B	CCR5	CIRPNNNTRKGIHIGPGRVFYATGDIIGDIRQAHC	35

KF716495	B	CCR5	CTRPSNNTMKSIHIGPGRAFYTTEQVIGDIRKAHC	35
KC699031	B	CCR5	CIRPGNNTRKSIHIGPGRAFYATGSIIGDIRQAHC	35
KC699034	B	CCR5	CTRPNNNTRRSISIGPGRAFYTTGQVIGDIRQAHC	35
KF716496	B	CCR5	CTRPSNNTRKSINIGPGRAFYTTGEIIGDIRQAHC	35
MH000302	B	CCR5	CVRPNNNTRKDIHIGPGRAFYATGEIIGNIRQAHC	35
MH000296	B	CCR5	CTRPNNNTRKSIHLGPGKAFYTTGEVIGDIRQAHC	35
MH000299	B	CCR5	CVRYNNNTRKSIHIGPGRALYATGEIVGDIRQAHC	35
KC699002	B	CCR5	CIRPNNNTRKGIHIGPGRAFYTTGDIIGDIRQAHC	35
KC699011	B	CCR5	CIRPNNNTRKSIHIGPGRAFYTTGQIIGDIRQAHC	35
KC699010	B	CCR5	CTRPNNNTRKSIHIGPGRAFYATGKIIGDIRQAHC	35
KC699009	B	CCR5	CTRPNNNTRKSIHIGPGRAFYTTGNIIGDIRQAHC	35
KC699008	B	CCR5	CTRPNNNTRKSIHIGPGRAFYATGEIIGDIRQAHC	35
KC699003	B	CCR5	CTRPNNNTRKSISIGPGRAFYTTGEIIGNIRQAHC	35
KC699004	B	CCR5	CTRPNNNTRKSIHIGPGKAFYATGDIIGDIKQAHC	35
KC699005	B	CCR5	CTRPNNNTRKSISIGPGRAFYTTGDIIGDIRQAYC	35
KC699001	B	CCR5	CTRPYNNTRKSIHIGPGKAFYATGDIIGDPRQAYC	35
AY535425	B	CCR5	CIRPNNNTRRSIHIGPGSAFYTTGQIIGDIRQAHC	35
AY713411	B	CCR5	CTRPNNNTRKSIHIGPGRAFYTTGDIIGDIRQAHC	35
AF041125	B	CCR5	CTRPNNNTRKSIHMGPGKAFYTTGEIIGDIRQAHC	35
AY535471	B	CCR5	CTRPNNNTRKGIHIGPGSAFYTTGEIIGDIRQAHC	35
AF041130	B	CCR5	CTRPNNNTRKSIHIGPGQAFYATGEIIGDIRQAHC	35
AF041131	B	CCR5	CTRPNNNTRKSISIGPGRAFYATGDIIGNIRQAYC	35
AF041134	B	CCR5	CTRPNNNTRKGIHIGPGRAFYATGEIIGDIRQAHC	35
AF041133	B	CCR5	CTRPNNNTRKSIHIGPGRAFYATGQIIGDIRQAHC	35
KC699014	B	CCR5	CIRPNNNTRRGIHIGPGQTLYATGEIIGDIRKAHC	35
KC699016	B	CCR5	CTRPNNNTRKSIPIGPGRAFYATDIIGDIRQAHC	34
KC699017	B	CCR5	CTRPNNNTRKSIPIGPGRAFYATGEIIGDIRKAYC	35
KC699012	B	CCR5	CTRPYNNTRKSIHIGPGRAFYATGDIIGDIRQASC	35
KC699020	B	CCR5	CTRPNNNTRKSIPIGPGKAFYATGEIIGDIRQAQC	35
KC699026	B	CCR5	CTRPNNNTRKSLAIGPGRAFYATGDIIGDIRKAHC	35
KC699015	B	CCR5	CTRPNNNTLKSIRFGPGSAFYTTGAIIGDIRQAHC	35
KC699021	B	CCR5	CTRPGNNTRKGIQMGPGKVFFATGEIIGDIRQAHC	35
AF321080	B	CCR5	CIRPNNNTRKSIHIAPGRAFYATGNIIGDIRQAHC	35
JN034158	B	CCR5	CTRPNNNTRRGIHLGPGGAFYTTGDIIGDIRKAHC	35
MG902199	B	CCR5	CTRPNNNTIKSIHIGPGRAFYATDIIGDIRQAHC	34
MG902157	B	CCR5	CTRPNNNTRKGIPIGPGRAFYATGDIIGNIRQAHC	35
HM586193	B	CCR5	CIRPNNNTRKSISMGPGRAFFNTGQIIGDIRQAHC	35
HM586198	B	CCR5	CTRPNNNTRKSIHIAPGRTFYATGDIIGDIRKAYC	35
HM586210	B	CCR5	CTRPNNNTRKSIHMGPGQALYTTNIIGDTRQAHC	34
FJ653437	B	CCR5	CTRPNNNTRRGIHIGPGRALYTTDIIGDIRQAHC	34
HQ595768	B	CCR5	CTRPNNNTRQGIHIGPGGAFFARGDIIGDPRQAHC	35
HQ595771	B	CCR5	CTRLNNNTRKSVRIGPGATFFATGDIIGDIRQAHC	35
HQ595772	B	CCR5	CIRPNNNTRRSIHMGPGKTFFAADIIGDIRQAHC	34
HQ595774	B	CCR5	CTRPGNNTRRSINIAPGRAWYTTGEVIGDIRQAHC	35
HM204626	B	CCR5	CTRPNNNTRKSINIGPGRAFYATGEVIGDIRKAHC	35
HM204627	B	CCR5	CTRPNNNTRKSIPIGPGRAFYATGDIIGDIRQAHC	35
HM204628	B	CCR5	CTRPNNNTRKGIHMGPGQAIYGYKTGEIIGNIRQAHC	37
HM204629	B	CCR5	CTRPNNNTRKSITIGPGRAFYATGDIIGDIRQAYC	35
HM204632	B	CCR5	CTRPSNNTRKSIHIGPGRAFYATGAIIGDIRQAHC	35
HM204633	B	CCR5	CTRPNNNTRKSIGIGPGSAIFATGDIIGDIRKAYC	35
HM204638	B	CCR5	CTRPNNNTRKSINLGPGRAFFATGEIIGDIRQAHC	35
HM204640	B	CCR5	CTRPNNNTIKGIHIGPGKAFYTTGAIIGNIRQAHC	35
HM204641	B	CCR5	CTRPNNNTRKSIHIAPGRAFYATGEIIGDIRNAHC	35
HM204642	B	CCR5	CTRPNNNTRRGIHVGPGRAIYTTGIIGDIRQAHC	34
HM204643	B	CCR5	CTRPNNNTRRSIPIGPGRAFYATGQIIGDIRKAYC	35
HM204646	B	CCR5	CTRPSNNTRKGIHMGPGRAFYATGDIIGDIRQAHC	35
KY658697	B	CCR5	CTRVNNNTRKSIHMGLGGAWHTTGNIIGDIRKAHC	35
HQ595776	B	CCR5	CTRPSNNTRKGIHIGPGRAFYATGQITGDIRQAHC	35
HQ595778	B	CCR5	CTRPNNNTRKSIHLGPGRAFYATGDIIGDIRQAHC	35
HQ595779	B	CCR5	CTRPHNNTRKSISTGPGRAIYATGHIIGDIRQAHC	35
HQ595788	B	CCR5	CTRPNNNTRRGIHIGPGQAFYTTDIIGDIRQAHC	34
HQ595792	B	CCR5	CTRPNNNTRKGITIGPGRAFYTTGGIIGNIRQAHC	35
HQ595794	B	CCR5	CTRPNNNTRKSIPLGPGRAFYTTGAIIGDIRQAYC	35
HQ595803	B	CCR5	CTRPNNNTRKSIHMGPGKAFYATDIIGDIRQAHC	34
JF680906	B	CCR5	CSRPNNNTRKSINIAQGRAIYATGEIIGDIRQAHC	35
HQ595769	B	CCR5	CTRPNNNTRKSIHIGPGKAFYTTGEVIGNIRQAHC	35
JF680910	B	CCR5	CTRPNNNTRKSIHIAPGRTFYATGDIIGDIRQAHC	35
JF680911	B	CCR5	CTRPGNNTRKSIPIGPGRAFFTTGAIIGDIRQAHC	35
JF680913	B	CCR5	CTRPSNNTRRSIHLGPGGTFYATGDIIGDIRQAHC	35
HQ595783	B	CCR5	CTRPNNNTRKSIPIGPGRAFYATGDIIGDIRQAHC	35

JF680919	B	CCR5	CTRHNNSTRKSIPLGPGRAIFATGDIIGDIRQAHC	35
JF680920	B	CCR5	CTRPNNNTRTSITLGPGRAYFATGDIIGNIRQAHC	35
HQ595786	B	CCR5	CTRPNNNTRKSIHIGPGRAFFATGEIIGEIRQAHC	35
JF680928	B	CCR5	CTRPNNNTRKSITIGPGRAFYATGDIIGDIRQAHC	35
JF680929	B	CCR5	CTRPGNNTRKSIHMGPGKAFYATGDIIGDIRQAHC	35
JF680934	B	CCR5	CVRPHNNTRKSIHIGPGSAFYATGAIIGDIRQAHC	35
HQ595801	B	CCR5	CTRPNNNTRKSIHIGPGRAFYAAEKIIGNIRQAHC	35
HQ595802	B	CCR5	CTRPNNNTRKGIHIGPGRAFYATGDIIGDIRQAHC	35
HQ595804	B	CCR5	CTRPNNNTRRGIHIAPGRAFYATGEIIGDIRQAHC	35
HQ595806	B	CCR5	CTRPSNNTRKGIHIGPGRAFYATGQVIGDIRQAHC	35
JF680937	B	CCR5	CTRPGNNTRRSIHIGPGRAFYATGAIIGDIRQAHC	35
JF706393	B	CCR5	CTRPGNNTRKFIRIGPGGPGSAFYTTKAVIGDIRQAH	38
JN786861	B	CCR5	CTRPNNNTRKSIHIGPGRAFYATGEITGDIRQAHC	35
AJ535594	B	CCR5	CTRPNNNTRKGIHIGPGRAFYATGGIIGDIRQAHC	35
AJ535607	B	CCR5	CTRPNNNTRKSIHMGPGRALYATGDIIGDIRQAHC	35
AJ535612	B	CCR5	CTRPNNNTRKSLNVGPGRAVYTTGDIIGDIRQAHC	35
AJ418531	B	CCR5	CTRPHNNTRKSIHIGPGRAFYATGEIIGDIRQAHC	35
AJ418494	B	CCR5	CTRPNNNTRKSISFGPGSAMYATGAIIGDIRQAHC	35
AJ418495	B	CCR5	CTRLNNNTRKSIHMGPGRAFFATGEIIGDIRQAHC	35
AJ418519	B	CCR5	CTRPNNNTRKSIHIGPGRAFYTTGGIIGNIRQAHC	35
AJ418521	B	CCR5	CTRPSNNTRKSIPIGPGRAFYTTGEIIGDIRQAHC	35
U36869	B	CCR5	CIRPNNNTRKGIPMGPGRAFYTTGQIIGDIRQAHC	35
U36872	B	CCR5	CTRPNNNTRKSIPIGPGRAFYATGDNIGDIRQAHC	35
U36879	B	CCR5	CTRPNNNTRKSIHIGPGEALFTTDIIGNIRQAHC	34
U36880	B	CCR5	CTRPSNNTRKSIPIGPGRAWYATGEIIGDIRKAHC	35
Y13719	B	CCR5	CTRPNNNTRKSINIGPGRAFYATGDIIGDIRQAHC	35
AJ286330	B	CCR5	CSRPSNNTSKSIHMGPGRAFYATERVIGNIRQAHC	35
AJ286337	B	CCR5	CTRPSNNTRKSIRIGPGSAFYATGEVIGDIRQAHC	35
AF491740	B	CCR5	CTRPNNNTRKSIPMGPGKAFYATGDIIGDIRQAHC	35
HQ122397	B	CCR5	CTRPNNNTRRSISIGPGRAFYATGDIIGDIRQAHC	35
JN002029	B	CCR5	CTRPNNNTRKSIPIGPGRAIYTTGGIIGDIRQAHC	35
DQ207940	B	CCR5	CVRPNNNTRKSISIGPGRAFYATGSIIGDIRKAHC	35
HQ385456	B	CCR5	CTRPNNNTRKGIPIGPGSAFYATGDIIGDIRQAHC	35
EU839603	B	CCR5	CTRFSNNTRRSIHIGPGRAFFATTNIIGDIRQAHC	35
EU839601	B	CCR5	CTRPNNNTRTSIHLGPGKTFFAGGVIGDIRQAHC	34
MH078551	B	CCR5	CTRPNNNTRRGIHIGPGRVFFATEVIGDIRQAYC	34
MK303352	B	CCR5	CVRPNNNTRKSIHIGPGKAFYATGAIIGNIRQAYC	35
MK303348	B	CCR5	CTRPNNNTRKGVPMGPGKTFFTTDVIGNIRQAHC	34
MK303350	B	CCR5	CTRPNNNTRKNIHMGPGRAWYATGEIIGDIRRAYC	35
MK303351	B	CCR5	CVRPNNNTRKSVHIGPGQAFFATGDIIGDIRQAHC	35
MK303353	B	CCR5	CIRPNNNTRRSIHMGPGAAMFATGEIIGDIRRAYC	35
MK303354	B	CCR5	CTRPNNNTRKSIHIGPGRAFYATGEIIGNIRQAHC	35
MK303355	B	CCR5	CTRPNNNTRKSIHMGPGRAFFAAGDIIGNIRKAHC	35
GU191372	B	CCR5	CTRPNNNTRRSINIQPGRAFFATGDIIGNIRQAYC	35
JF680936	B	CCR5	CTRPNNNTRKSIPIGPGRAFYATGEIIGDIRRAHC	35
KF061032	B	CCR5	CTRPNNNTRRSIHIGPGKAFYTGEIIGNIRQAHC	34
KF061031	B	CCR5	CIRPNNNTRKSIPMGPGKAFYATGDIIGNIRQAHC	35
AY835445	B	CCR5	CTRPNNNTRRSIHIGPGRAFYATGDIIGDIRQAHC	35
AY835444	B	CCR5	CTRPNNNTRKSISIGPGRAFYATGDIIGDIRQAHC	35
EU839605	B	CCR5	CVRPNNNTRTTSVHIGPGRAIYATGDIIGDIRRAYC	36
JF680925	B	CCR5	CTRPNNNTRKGIHMGPGGAFYATDQIIGDIRRAYC	35
HM030559	B	CCR5	CTRPNNNTRKSVPIGPGRAFYATGDIIGNIRKAYC	35
HM030562	B	CCR5	CERPNNNTRKGIHIGPGRAFYATGDIIGDIRQAHC	35
HM030565	B	CCR5	CTRPGNNTRKSIGIGPGRAFYTTGAIIGNIRQAHC	35
AB289590	B	CCR5	CTRPNNNTRKGIHIGPGRAFYTTGEIVGNIRQAHC	35
AB428558	B	CCR5	CTRPNNNTRKGVHLGPGQAIYTTGAIIGDIRQAYC	35
AB221126	B	CCR5	CTRPNNNTRKGIHIGAGRAIYATGAIIGDIRQAHC	35
AB480697	B	CCR5	CIRPNNNTIKGIHIGPGRAFYATGKITGDIRQAHC	35
AB588283	B	CCR5	CTRPNNNTRKSIQMGPGKSIYATGNIIGDIRQAHC	35
AB287367	B	CCR5	CTRPNNNTRKSIHIGPGRAFYATGDIIGNIRQAHC	35
AB287368	B	CCR5	CTRPNNNTRKSIPMGPGSAIYATGAIIGDIRQAHC	35
AB588311	B	CCR5	CTRPNNNTRKGIHMGPGRAYYTTGQIIGDIRQAHC	35
AB731663	B	CCR5	CTRPNNNTRKGIHIGPGRAFYTTGQIVGDIRQAHC	35
AB731667	B	CCR5	CTRPSNNTRKGIQIGPGRAFYTTGEIIGDIRQAHC	35
KF716497	B	CCR5	CTRPNNNTRNSVHIGPGRALYTTNIIGDIRRAHC	34
KF716498	B	CCR5	CTRPNNNTRRSIHLGPGQTLYATGDIIGDIRQAHC	35
AB588207	B	CCR5	CTRPNNNTRKGIHMGPGGAFYTTGEITGDIRQAHC	35
AB588209	B	CCR5	CTRPNNNTRKGIHMGWGRAFYATGAIIGNIRQAHC	35
AB588211	B	CCR5	CTRPNNNTRKSIPMGPGRAFYATGDIIGDIRQAHC	35
AB588233	B	CCR5	CTRPNNNTRKGIHIGPGRALFYATDIIGDIRQAHC	35

AB480698	B	CCR5	CTRPHNNTRRSISFTPGSAFVATENIIGDIRQAHC	35
AB604946	B	CCR5	CTRPNNNTRKSIHIAPGRAWYATGDIIGDIRQAHC	35
AB565497	B	CCR5	CTRPNNNTRKGIHMGPGGALFTTEITGNIRQAHC	34
AB565502	B	CCR5	CTRPNNNTRKSIPMGPGQAWFATGDIIGDIRQAHC	35
KT961002	B	CCR5	CTRPNNNTRKSIHIGPGSAFYTTGQIIGNIRQAYC	35
AB742153	B	CCR5	CTRPNNNTRKGIHMGPGRAFYGTDIIGDIRQAHC	34
AB641836	B	CCR5	CTRPNNNTRRSIHIGPGRTIFATDIIGDIRQAHC	34
KU869602	B	CCR5	CTRPNNNTRKGIHLGPGKTFFATGEVIGDIRQAHC	35
KU869602	B	CCR5	CTRPNNNTRKGIHLGPGKTFFATGEVIGDIRQAHC	35
MN043603	B	CCR5	CTRPNNNSRKGIGIGPGSTFYATGDIIGNIRQAHC	35
JQ316128	B	CCR5	CTRPNNNTRKSIHMGPGRTYYATGAIIGNIRQAHC	35
JQ316132	B	CCR5	CIRPNNNTRKGIHIGPGSTLYATGEIIGDIRQAHC	35
JQ316135	B	CCR5	CTRPGNNTRKSIRIGPGSTFYATGDIIGDIRQTHC	35
JQ316130	B	CCR5	CTRPNNNTRRSIRIGPGSAFYTTGEVIGNIRLAYC	35
DQ295195	B	CCR5	CTRPNNNTRKSISFAPGSTIYATGGIIGDIRQAHC	35
DQ295193	B	CCR5	CTRPNNNTRKSIRIGPGSTFYATGDIIGDIRQAHC	35
AY839827	B	CCR5	CTRPNNNTRKSIPMGPGGMIYATGAIIGDIRQAHC	35
DQ837381	B	CCR5	CTRPNNNTRKSIHIGPGKAFYAAGDIIGNIRQAHC	35
JQ316134	B	CCR5	CTRPHNNTRKSIHLGPGSTFFATGAIIGNIRQAHC	35
MN043605	B	CCR5	CIRPSNNTRRSISFAPGSTFYATGDIIGDIRQAHC	35
KJ140263	B	CCR5	CIRPNNNTRKSIHLGPGSTIYATGDIIGDIRQAHC	35
KT878030	B	CCR5	CTRPNNNTRKSITLGPGRAFYATGDIIGDIRQAHC	35
KT878031	B	CCR5	CTRPNNNTRKSIHIAPGRAFYATGGIIGDIRQAHC	35
KT878032	B	CCR5	CTRPNNNTRKSIGFVPGASFIATGAIIGDIRQAHC	35
KT878033	B	CCR5	CTRPNNNTRRSVHIAPGSAIFATGDIIGDIRQAYC	35
KT878036	B	CCR5	CVRPNNNTRKSITLGPGSAFYATGDIIGDIRQAHC	35
KT878037	B	CCR5	CTRPGNNTIRSIHVGPGRAFYTPGTIIGDIRKAYC	35
KT878038	B	CCR5	CTRPNNNTRKGIHFGPGRTIYTTGEIVGDIRQAHC	35
KT878022	B	CCR5	CTRPNNNTRRSIHVGPGSIMYATGAIIGDIRQAHC	35
KT878023	B	CCR5	CTRPNNNTRKSISIGPGRAVYATGQIIGNIRQAHC	35
KT878024	B	CCR5	CIRPNNNTRKGIPIGPGRAIYASGDIIGDIRQAHC	35
KT878025	B	CCR5	CIRPNNNTRRSIHIGPGRAFYATDIIGDIRQAYC	34
KT878027	B	CCR5	CTRPSNNTRKSIRFGPGSAFFAHGDVTGDIRKAYC	35
MK871374	B	CCR5	CTRPNNNTRTSITLGPGRAFFATGDIIGNIRYAYC	35
MN043576	B	CCR5	CTRPNNNTRKSIRIGPGSTFYATGGIIGDIRQAHC	35
KU896118	B	CCR5	CTRPNNNTRKGIGLGPGSTFYATEAIIGDIRQAHC	35
KU896118	B	CCR5	CTRPNNNTRKGIGLGPGSTFYATEAIIGDIRQAHC	35
MN043588	B	CCR5	CTRPNNNTRKSTSIGPGRTFYATGDIIGNIRQAHC	35
KU869580	B	CCR5	CTRPNNNTRKSIHLGPGSTFYATGDIIGDIRQAHC	35
MN043589	B	CCR5	CTRPNNNTRTSISIGPGRTFYATGDIIGDIRQAHC	35
KU869596	B	CCR5	CTRPGNNTSKSIRIGPGSTFYATERIIGDIRQAHC	35
KX960971	B	CCR5	CIRPNNNTRKSISFAPGSTFYATGDIIGDIRQAHC	35
KJ140267	B	CCR5	CTRPNNNTRKSIHLGPGSTFFATGEIIGDIRQAHC	35
KJ140248	B	CCR5	CTRPNNNTRKSIDIGPGRAIYATGDIIGDIRQAHC	35
KJ140252	B	CCR5	CTRPNNNTRKSISIGPGRTFYATGEIIGDIRQAHC	35
AJ417425	B	CCR5	CTRPNNNTRKSINIGPGRAFYATGDIIGDIRQAHC	35
KY820529	B	CCR5	CIRPNNNTRKSIRIGPGSTFYATGGIIGNIRQAHC	35
KU869580	B	CCR5	CTRPNNNTRKSIHLGPGSTFYATGDIIGDIRQAHC	35
MN043589	B	CCR5	CTRPNNNTRTSISIGPGRTFYATGDIIGDIRQAHC	35
KU869596	B	CCR5	CTRPGNNTSKSIRIGPGSTFYATERIIGDIRQAHC	35
KX960978	B	CCR5	CTRPNNNTRKSIHLGPGRTFYATGDIIGDIRQAHC	35
AJ417415	B	CCR5	CTRPNNNTRKGIHMGPGRTFYATGDIIGDIRQAHC	35
AJ417431	B	CCR5	CTRPNNNTRQSISIGPGRALYTTGQIIGDIRQAYC	35
AJ417410	B	CCR5	CTRPGNNTRRSISIGPGRAFYATGSIIGNIRQAHC	35
AJ417413	B	CCR5	CTRPNNNTRKSIHIGPGSTIYATGDIIGDIRQAHC	35
AJ417419	B	CCR5	CTRPNNNTRKSIRIGPGSAFYANGDIIGDIRQAHC	35
AF224507	B	CCR5	CTRLNNNTRKSIRIGPGSTFYATGAIIGDIRQAHC	35
AB097870	B	CCR5	CIRPNNNTRKSINLGPGQAWYTTGDIIGDIRQAHC	35
AY423386	B	CCR5	CTRPNNNTRKSIHIGPGRAFYATGEIIGDIRQAHC	35
HQ386146	B	CCR5	CTRPSNNTSKGIHMGPGRAFYATGQITGDIRKAYC	35
AY970946	B	CCR5	CTRPNNNTRKSIHIGPGRAFYATGEIIGDIRQAHC	35
GU455480	B	CCR5	CTRPNNNTRKGIHIGPGRAFYATGEIIGDIRQAHC	35
EU744159	B	CCR5	CTRPNNNTRRSIHIGPGRAFYATGDIIGDIRQAHC	35
GU455505	B	CCR5	CIRPNNNTRKSIHIGPGRALYTTEIIGDIRKAYC	34
GU455448	B	CCR5	CTRPNNNTRKSIPIGPGRAFYATGDIIGDIRQAHC	35
DQ178989	B	CCR5	CTRPNNNTRRSIPIGPGRAFYATGDIIGDIRKAHC	35
EU744007	B	CCR5	CTRPNNNTRKSISIGPGRAFYATGDIVGDIRKAHC	35
GU455525	B	CCR5	CIRPNNNTRKGIHIGPGRAFYTTGQIIGDIRQAHC	35
EU744052	B	CCR5	CTRPSNNTRRGIHIGPGRAFYTTGEIIGDIRQAHC	35
JF320008	B	CCR5	CTRPNNNTRKSINIGPGRAFYATEAIVGNIRQAHC	35

JF320215	B	CCR5	CTRPNNNTRKSINIGPGRAFYATGDIIGDIRQAHC	35
JF320208	B	CCR5	CTRPNNNTRKSIHIAPGGAFYATGDIIGDIRQAHC	35
JF320230	B	CCR5	CIRPNNNTRKSIPIGPGRAFYTTGEIIGNIRQAHC	35
JF320013	B	CCR5	CTRPNNNTRKSINIGPGRAWYATGDIIGDIRQAHC	35
JF320018	B	CCR5	CIRPNNNTRKSIHLGQGRTWYATGEIIGDIRQAHC	35
JF320028	B	CCR5	CTRPNNNTRKSVHIGPGQVLYTGDIIGNIRQAHC	34
JF320019	B	CCR5	CTRPNNNTRRSIHVGPGKALYTTDIIGDIRQAYC	34
MH078552	B	CCR5	CTRPSNNTRKGIHIGPGRAFYATGDIIGDIRQAHC	35
MH078553	B	CCR5	CTRPNNNTRKSIHIGPGRAFYATGEIIGNIRQAHC	35
MH078555	B	CCR5	CIRPNNNTIKEVRIGPGAYFRAGQIIGDIRKAYC	34
EF210726	B	CCR5	CTRPNNNTRKSIHIGPGRAFYATGDIIGDIRQAHC	35
EF210730	B	CCR5	CTRPNNNTRKSIPIGPGRAFYTTGDIIGDIRQAHC	35
EF210732	B	CCR5	CIRPYNNTRKSIHMGPGRAFYATGEIIGDIRKAHC	35
KC596069	B	CCR5	CTRPNNNTRKSINIGPGRAFYTTGEIIGDIRQAHC	35
JN251901	B	CCR5	CVRPSNNTRRSINIGPGRAFYTTGEIIGDIRQAHC	35
AY819715	B	CCR5	CTRPSNNTRKGIPIGPGRAWYATGDITGDIRKAHC	35
HQ896488	B	CCR5	CTRPNNNTRKSIHIGPGRAFYATGQIIGDIRQAHC	35
JX500709	B	CCR5	CERTNNNTRTDIHIGPGRAFHAIGDIKGDIRVAHC	35
JX500707	B	CCR5	CTRPNNNTRKSINIGPGRAFYATGDIIGDIRQAHC	35
JX500708	B	CCR5	CTRPNNNTRKGIHMGPGKAFYATGDIIGDIRQAHC	35
MF373142	B	CCR5	CTRPNNNTAKSIPMGPGRAFYTTGRIIGDIRQAHC	35
MF373144	B	CCR5	CTRPNNNTRKSIHIGPGRAFYATGDIIGDIRQAHC	35
MF373161	B	CCR5	CTRPNNNTRKSIHIGPGRAFYATGDIIGDIRQAYC	35
KP411828	B	CCR5	CIRPNNNTRKGIHIGPGRAFYATGDIIGDIRKAHC	35
MF373204	B	CCR5	CTRPGNNTRKSIHFGPGKTFYATGDIIGDIRQAHC	35
GU204920	B	CCR5	CARPNNNTRKSIHIAPGRAFHTTGSIIGDIRKAYC	35
JN251856	B	CCR5	CTRPNNNTRKGIHIGPGRTLYATGGIIGNIRQAHC	35
KY213741	B	CCR5	CTRPNNNTRKSIHMGQGKAWYTTGEIIGDIRQAYC	35
KY213740	B	CCR5	CTRPSNNTRKSIHIGPGSAFYATGDIIGDIRQAHC	35
KY213747	B	CCR5	CTRPNNNTRKSIHLGPGKAWYATDIIGDIRQAHC	34
KY213748	B	CCR5	CTRPNNNTRKSIHLGPGKAWYATDIIGDIRQAHC	34
AY945710	B	CCR5	CTRPNNNTIKGIHIGPGQAWYTTGQIIGDIRKAHC	35
KJ952535	B	CCR5	CTRPNNNTRKSIPLGPGRAWYTTGQIIGDIRHAHC	35
JN248321	B	CCR5	CTRPNNNTRKSIHIAPGRAFYTTGEIIGNIRQAHC	35
JN248333	B	CCR5	CTRPNNNTRKGIHFAPGSTFYATGEIIGDIRQAYC	35
JN248335	B	CCR5	CTRPNNNTRKSIPLGPGKAWFATGEIIGDIRQAHC	35
JN248337	B	CCR5	CTRPNNNTRKSIHLGPGRALFTTDIIGDIRKAYC	34
JN248343	B	CCR5	CTRPNNNTRRSIPVGPGRVLYTTGIIGDIRQAHC	34
JN248344	B	CCR5	CTRPNNNTRKSISFTPGSTFYATDIIGNIRQAHC	34
JN248347	B	CCR5	CTRPNNNTRKSIHLGQGRAWYATGDIIGDIRQAHC	35
JN248353	B	CCR5	CTRPNNNTRRGIHIGPGRGMFYATDIIGDIRKAHC	35
JF297222	B	CCR5	CTRPNNNTRQGIHLGPGRAFYSTGDIIGDIRQAYC	35
JX446800	B	CCR5	CTRPNNNTRKSIHIGPGRTFYATGDIIGDIRKAYC	35
JX446818	B	CCR5	CTRPNNNTRKSIHIGPGRAFYAAGDIIGDIRQAHC	35
JX447795	B	CCR5	CSRPSNNTRKGINIGPGQAWYTTDQVVGNIRQAHC	35
JX447156	B	CCR5	CTRPNNNTRKSIPLGPGKAWYATGDIIGEIRQAHC	35
MG989620	B	CCR5	CIRPGNNTRRSITIGPGRAFYTGEIIGDIRQAHC	34
MG989578	B	CCR5	CTRPNNNTRKSIHIAPGRAFYTTGEIIGNIRQAYC	35
MG989587	B	CCR5	CTRPNNNTRKSIPIGPGRVFYATGDIIGDIRQAHC	35
MG989506	B	CCR5	CTRPYNNTRKSIHIGPGRTWYATGEVIGDIRAAYC	35
MG989541	B	CCR5	CTRPNNNTRRGIHIGPGGPIYTTGDIIGDIRKAHC	35
MG989610	B	CCR5	CTRPNNNTRKSIHMGPGRTIYATGSIIGDIRQAHC	35
MG989551	B	CCR5	CTRPNNNTRRSIHIAPGQAFYATGSIIGNIRQAHC	35
U39258	B	CCR5	CTRPNNNTRKSIPLGPGQAWYTTGQIIGDIRQAHC	35
DQ354116	B	CCR5	CTRPNNNTRKSITIGPGRAWFATGQIIGNIRQAHC	35
DQ354119	B	CCR5	CTRPNNNTRKSINIGPGRAFWATGGIIGDIRQAHC	35
HM215431	B	CCR5	CTRPNNNTRRSIHIGPGRAFYTTDIIGNIRAAHC	34
HM215435	B	CCR5	CTRPNNNTRKSISIGPGRAWYATGDIVGDIRQAHC	35
EU839606	B	CCR5	CTRPNNNTRRSITIGPGSVFYTGEIIGDIRQAHC	34
EU839607	B	CCR5	CTRPNNNTRKSVHIGPGRVFYAGEIIGNIRQAHC	34
HQ217662	B	CCR5	CTRPNNNTRRGITIGPGRVFYTGEIIGNIRQAHC	34
EU839608	B	CCR5	CTRPNNNTRRSVHIGPGSVFYTGEIIGDIRKAYC	34
EF593260	B	CCR5	CTRPNNNTRKSIHMGPGKAFYTGDIIGDIRQAHC	34
EU578580	B	CCR5	CTRPNNNTRRSIHLGPLSAFYTGEIIGDIRQAHC	34
EU578603	B	CCR5	CTRPNNNTRKSIHLGAGRALYTNGEIIGNIRHAHC	35
AF277060	B	CCR5	CTRPNNNTRKGITIGPGSVFYTGEIIGDIRQAHC	34
AF277055	B	CCR5	CTRPNNNTRRSIHMGPGKVFYTGEIIGDIRQAHC	34
EF593245	B	CCR5	CTRPNNNTRRGIHMGPGKAYFTGEIIGDIRQAHC	34
AF277070	B	CCR5	CTRPNNNTRRSIHMGPGKALFTGEIIGDIRQAHC	34
AF277072	B	CCR5	CTRPNNNTRRGVTIGPGRVFYTGEVTGDIRQAHC	34

AF277073	B	CCR5	CIRPNNNTRKSIHIGPGRAFYTGDIIGDIRQAHC	34
EU578429	B	CCR5	CTRPNNNTRKSIHLGPGSVFYTGEIIGNIRQAHC	34
EU577148	B	CCR5	CTRPNNNTRRSITFGPGASFYTGEIIGDIRKAFC	34
EF593264	B	CCR5	CTRPNNNTRKSIHIGQGAAFFTGEIIGDIRQAHC	34
EF593268	B	CCR5	CSRPNNNTRRSVHIGPGRAWYTGEIIGDIRQAHC	34
U32396	B	CCR5	CTRPNNNTRKSIHIGPGRAFYTGEIIGDIRQAHC	34
DQ823362	B	CCR5	CTRPNNNTRKGIHIGPGGTFFATGAIIGNIRQAHC	35
DQ823364	B	CCR5	CTRPNNNTRKSIHFGPGGAIYATGDIIGDIRQAHC	35
JN562768	B	CCR5	CTRPNNNTRKGVHMGPGKVFFATGAIIGDIREAHC	35
DQ869026	B	CCR5	CTRPNNNTRKSIHIAPGRPFYATGDIIGDIRQAHC	35
EF363123	B	CCR5	CTRPNNNTRKSIHIAPGRAFYATGEIIGDIRQAHC	35
EU289199	B	CCR5	CTRPNNNTRKGIHIGPGKTLYATGEIIGDIRQAHC	35
JN944930	B	CCR5	CTRPNNNTSKSIHMGPGGAFFATGRIIGDIRKAYC	35
JN944938	B	CCR5	CTRPGNNTRRSINIGPGRAFYATGAIIGDIRKAHC	35
EF593288	B	CCR5	CMRPNNNTRKSIHLAPGRALYTTGDIIGDIRQAHC	35
EF593289	B	CCR5	CTRPNNNTRRSINMGPGRAFFTTGDVIGDIRQAHC	35
HM769944	B	CCR5	CTRPNNNTRKGVHMGPGGALFATDVIGDIRKAHC	34
JQ609746	B	CCR5	CTRPNNNTRRSIPMGPGKAFYTTGAIIGDIRQAHC	35
DQ869017	B	CCR5	CIRPNNNTRRSIPIGPGRAFYATGDIIGDIRQAHC	35
DQ869019	B	CCR5	CTRPSNNTRKGIHIGPGRAFYTTGDIIGDIRQAHC	35
DQ869014	B	CCR5	CTRPNNNTRKSIPIGPGRAFFATDIIGDIRQAHC	34
FJ469738	B	CCR5	CTRPNNNTRKSIHIGPGRAFYATGDIIGDIRQAYC	35
KT283937	B	CCR5	CTRPHNNTRKSIHIAPGRAFYATGDIIGDIRQAHC	35
EF593254	B	CCR5	CTRPNNNTRRSINIGPGRAFYAHGDIIGDIRQAHC	35
JN944936	B	CCR5	CTRPHNNTRKSIHIGPGRAWYATGDIIGDIRKAYC	35
EF593291	B	CCR5	CTRPNNNTRKGVHFGPGKAFFLTGDIIGDIRQAHC	35
EF593293	B	CCR5	CTRPNNNTRKSIPMGPGRAWYATGNIIGDIRQAHC	35
EF593294	B	CCR5	CTRYNNNTRKSIHMGPGAAFYATGDIIGDIRQSHC	35
GU330249	B	CCR5	CTRPGNNTRKSIHPGPGRAIYATEAIIGDIRRAHC	35
GU330333	B	CCR5	CTRPNNNTRKSIPIGPGRALYTTGEVIGDIRKAYC	35
KT124777	B	CCR5	CTRPNNNTRKSISIGPGRVFYATGGIIGNIRQAYC	35
KT124795	B	CCR5	CTRPGNNTRKSIPLGPGRAFYATGDIIGDIRQAHC	35
DQ869020	B	CCR5	CTRPSNNTRKSITIGPGRAFYATGDIIGDIRQAHC	35
DQ869021	B	CCR5	CTRPGNNTRRSIPMGPGRAWYAIGEITGNIRKAHC	35
DQ869022	B	CCR5	CTRPGNNTRKSIHIAPGRAFYATGDIIGDIRQAHC	35
FJ469687	B	CCR5	CTRPNNNTRRSIHIGPGSAFYATGDIIGNIRQAHC	35
FJ469688	B	CCR5	CTRPNNNTRKSIHIAPGRAFYATGDVIGDIRQAYC	35
FJ469693	B	CCR5	CTRPNNNTRKSISIGPGRAFYATGDIIGDIRQAHC	35
HQ217201	B	CCR5	CTRPNNNTRKSIHITPGRAFFTTGEVIGDIRRAHC	35
FJ469730	B	CCR5	CTRPNNNTSKSIHLGPGRAFHVTGEIIGDIRQAHC	35
JQ403102	B	CCR5	CTRVSNNTRKSISIGPGKAFYTTGEVIGDIRKAHC	35
FJ469741	B	CCR5	CTRPNNNTRKSIHIGPGKAFYATGDIIGDIRQAHC	35
FJ469760	B	CCR5	CTRPNNNTRRSIHIAPGSALYTTDIIGDIRQAHC	34
FJ469762	B	CCR5	CTRPNNNTRKGINIGPGRALYATGAIIGNIRQAHC	35
EU578460	B	CCR5	CTRPNNNTRKGIHMGPGGAIYATGAIIGNIRQAHC	35
EF593296	B	CCR5	CTRPGNNTSKSIHMGAGKAFYATGRIIGDIRQAHC	35
EF593297	B	CCR5	CTRPNNNTRKSIHMGPGKTFYAAGDIIGDIRQAHC	35
GU330460	B	CCR5	CTRPNNNTRKSIPIGPGRAFYATGEIIGDIRQAHC	35
HM234503	B	CCR5	CTRPNNNTRKGIHIGPGRAFYATGEIIGDIRKAHC	35
KC312470	B	CCR5	CTRPNNNTRKGIHIGPGRTFYATGEIIGNIRQAHC	35
KT124807	B	CCR5	CTRPNNNTRRGINIGPGRAWYTTGIIGDIRQAHC	34
KT124813	B	CCR5	CTRPNNNTRRSVHMGPGRAFFTTDIIGDIRQAHC	34
DQ869016	B	CCR5	CTRPNNNTRKSISIAPGRAWYATGDIIGDIRQAHC	35
DQ869024	B	CCR5	CTRPNNNTRKGIHMGPGKTLYATGEIIGDIRQAHC	35
DQ869025	B	CCR5	CTRPNNNTRKGIHIGPGRAFYATGEIIGNIRQAYC	35
DQ869029	B	CCR5	CTRPNNNTRKSIHMGPGKAFYATGEIIGDIRKAYC	35
DQ869030	B	CCR5	CTRPNNNTRKGIHIGPGRAFYATGEIIGDIRQAHC	35
DQ869031	B	CCR5	CTRPNNNTRKGIHMGPGRTFYATGEIIGDIRQAHC	35
FJ469701	B	CCR5	CTRPGNNTRKSITIGPGRAFYATGEIIGDIRKAHC	35
KC473846	B	CCR5	CTRPSNNTRRGIHIGPGQAFYTTGQIIGDIRQAYC	35
EF593222	B	CCR5	CTRPNNNTRKSIPIGPGRAFYATGDIIGDIRQAHC	35
DQ410465	B	CCR5	CTRPGNNTRKSIHIGPGRAFYTTGEIIGNIRQAHC	35
FJ469743	B	CCR5	CTRPNNNTRKSIHIGPGQAWYATGEIIGNIRQAHC	35
EF593228	B	CCR5	CTRPNNNTRRSIHIGPGRAWFATGDVIGPIRKAYC	35
FJ469755	B	CCR5	CTRPNNNTRKSINIGPGRAFYATGDIIGDIRQAHC	35
FJ469765	B	CCR5	CTRPNNNTRRSISVGPGRAIYATGDIIGDIRQAHC	35
FJ469770	B	CCR5	CTRPNNNTRTSVHLGPGRAFYTTGDIIGDIRQAHC	35
EF593299	B	CCR5	CTRPNNNTRKSIHIGPGRTFYAPGEIIGNIRQAHC	35
HQ216370	B	CCR5	CSRPNNNTRKGIHIGPGRTFYATGEIIGDIRQAHC	35
HQ216504	B	CCR5	CTRPNNNTRKSIHIAPGRTFYATGEIIGDIRQAHC	35

JX863969	B	CCR5	CVRPNNNTRKSIHFGPGSVFYATEIIGNIRQAYC	34
JN562798	B	CCR5	CTRPNNNTRRSIPIGPGRAFYATDIIGDIRQAHC	34
EU577811	B	CCR5	CTRPNNNTRRGITIGPGRAFYATGEIIGDIRQAHC	35
EU577862	B	CCR5	CTRLNNNTRKSITIGPGRAFYATGEITGDIRKAHC	35
EU577947	B	CCR5	CTRPNNNTRKSITIGPGRAFYATGDIIGDIRQAHC	35
EU577989	B	CCR5	CMRPNNNTRRGIHMGPGKVFYATGAIIGDIRQAHC	35
KX156438	B	CCR5	CTRPNNNTRRSIHIGPGRAFYTTGDIIGNIRQAHC	35
EU578032	B	CCR5	CTRPNNNTRKSIHIAPGSAFYTTGQIIGDIRQAHC	35
KX156451	B	CCR5	CTRPHNNTRKGIHMGPGRAFYTTGEIVGDIRQAHC	35
JN562778	B	CCR5	CTRPNNNTRKSITIGPGRAFYGTDIIGDIRQAHC	34
JN562811	B	CCR5	CTRPNNNTRRSIHIGPGKAFYGTNVIGDIRQAHC	34
DQ869015	B	CCR5	CTRPNNNTRRSIPIGPGRAFYATDIIGDIRQAHC	34
DQ869033	B	CCR5	CTRPNNNTRKGVHIGPGRAFYATGEIIGDIRKAHC	35
KM259097	B	CCR5	CTRPNNNTRKSIHMGPGGAFYATGDIIGNIRQAHC	35
DQ410535	B	CCR5	CTRPANNTRKSIHIAPGRAFHTTGGIIGDIRQAHC	35
AY835452	B	CCR5	CTRPNNNTSKSITIGPGRAFYATGRIIGDIRKAHC	35
KX156493	B	CCR5	CTRPNNNTRKGIHIGPGRAFYTTGEVVGNIRQAYC	35
KX156542	B	CCR5	FTRPNNNTRKSIPIGPGRAFYATGDITGDIRQAHC	35
KX156380	B	CCR5	CTRPSNNTRKSINIGPGRALYATGEITGDIRQAHC	35
FJ469697	B	CCR5	CTRPSNNTRRSIHIGPGRAFYTTGEIIGDIRQAHC	35
HQ217185	B	CCR5	CTRPNNNTRKSINIGPGRAWYAMGQVIGNIRQAHC	35
KC935957	B	CCR5	CTRPNNNTRKSISIGPGRAFYTTGEIIGDIRQAHC	35
DQ410068	B	CCR5	CTRPNNNTRKSIHIGPGRAFYTTGDIIGDIRQAHC	35
EF363124	B	CCR5	CTRPNNNTRKSIHIGPGRAFYTTGEIIGDIRQAHC	35
EF363126	B	CCR5	CTRPNNNTRKSIPIGPGRAFYATGNIIGDIRQAHC	35
DQ410260	B	CCR5	CIRPNNNTRKSIHIGPGRAFYATGDIIGDIRQAHC	35
DQ410292	B	CCR5	CTRPSNNTRKSIHIGPGRAFYATEKIIGNIRQAHC	35
DQ410356	B	CCR5	CTRPNNNTRKSLHITPGSAIYATGDIIGDIRQAHC	35
JQ403103	B	CCR5	CTRPNNNTRKSIHIAPGRAFFATGEIIGEIRKAHC	35
FJ469745	B	CCR5	CTRPNNNTRRSIHIGPGRSFYATDIIGDIRQAHC	34
MK147530	B	CCR5	CTRPGNNTRKGIHIGPGGAFYTRGQITGDIRQAHC	35
FJ469772	B	CCR5	CSRPNNNTRKSIHMGPGSAFYATGDIIGDIRQAHC	35
EF593269	B	CCR5	CTRPNNNTIKGIPIGPGQAFYATETIVGEIRKAYC	35
EF593271	B	CCR5	CTRPNNNTRKGIHIGPGRTFFATGGIVGDIRQAHC	35
EF593273	B	CCR5	CTRPNNNTRKGINIGPGRAFYTTGEIIGDIRQAHC	35
EF593274	B	CCR5	CTRPNNNTRKGIHIGPGRAFYATGAIIGDIRKAYC	35
EF593276	B	CCR5	CTRPNNNTRKSVHIGPGSAFYATGDIIGDIRXAHC	35
JN024210	B	CCR5	CTRPNNNTRKSIHIAPGRAFYATGEIIGDIRQAHC	35
FJ469683	B	CCR5	CTRPNNNTRKSIHIAPGRAFYATGAIIGNIRQAYC	35
HQ238279	B	CCR5	CVRPNNNTRKGISFGPGKAIYTTGEIIGNIRQAHC	35
FJ496085	B	CCR5	CTRPNNNTRKGIHIGPGGAFFTTGQIIGDIRQAHC	35
KC595159	B	CCR5	CTRPNNNTRKSIHIGPGRAFYTTGEIIGDIRQAHC	35
JF689856	B	CCR5	CTRLSNNTRRSIHIGPGRAFYATGEIIGDIRQAHC	35
JF689857	B	CCR5	CTRPNNNTRKSIHIGPGRAFYTTGDIIGDVRQAHC	35
JF689859	B	CCR5	CTRPNNNTRKSIHIGPGKAFYATGEIIGDIRQAHC	35
JF689860	B	CCR5	CTRPGNNTKQSITMGPGRAFYTTGKVIGDIRQAHC	35
HQ216575	B	CCR5	CIRPNNNTRRGINIGPGRAFYATGAIIGDIRRAYC	35
HQ216584	B	CCR5	CTRPNNNTRRSVHMGPGSAFYTTGGIIGDIRQAHC	35
HQ216686	B	CCR5	CIRPNNNTRKSIHIGPGRAFYATDIIGDIRQAHC	34
HQ216727	B	CCR5	CTRPNNNTMKSIHIGPGRAFYTTGKIIGDIRQAHC	35
HQ216841	B	CCR5	CTRPNNNTRKSIAMGPGRAFWTTGDIIGDIRQAHC	35
JX863966	B	CCR5	CIRPNNNTRRSIHMGPGRAFSTTGDIIGDIRQAHC	35
JX863971	B	CCR5	CTRPGNNTRRSIHMGPGKVFYATGDIIGDIRQAHC	35
JX863972	B	CCR5	CTRPGNNTRRSIHMGPGKVFFAQDIIGDIRQAHC	34
JX863919	B	CCR5	CTRPNNNTRKSITIGPGRAFYATGAIIGDIRQAHC	35
JX863983	B	CCR5	CTRPNNNTRKSISIGPGRAFYATGEIIGDIRQAHC	35
JX863984	B	CCR5	CTRPSNNTRKSIHIAPGRAFYATGDITGDIRLAYC	35
JX863985	B	CCR5	CTRPNNNTRRSIPIGPGRAFYATGDIIGNIRKAYC	35
JX863986	B	CCR5	CIRPHNNTRKGIHIGPGRAFYATGEITGDIRKAHC	35
JF320059	B	CCR5	CTRPNNNTRKSIHIVPGRAFYATGDIIGDIRKAYC	35
JF320361	B	CCR5	CTRSNNNTKKSIHMGPERAFYTTGGIIGDIRQAHC	35
JF320054	B	CCR5	CIRPNNNTRKSVRIGPGSAFYATGGIIGDIRQAHC	35
EU577917	B	CCR5	CTRPGNNTRKGIHMGPGKVFYTTDVIGDIRKAHC	34
EU578004	B	CCR5	CERPNNNTRKGIHIGPGRYAFYATGNIIGDIRQAHC	36
KM258984	B	CCR5	CIRPNNNTRKSIHIGPGRALFDATDIIGDIRQAYC	35
KM259000	B	CCR5	CTRPNNNTSKGIHMGPGRAFYATGRIIGDIRQAHC	35
KM259100	B	CCR5	CERPNNNTRKSIHMGPGQALYATGNIIGNIRQAHC	35
DQ410616	B	CCR5	CTRPNNNTRTGINIGPGRAWYTTKDIIGNIRNAYC	35
DQ410553	B	CCR5	CTRPNNNTRKGIHLGPGGAFFTTGQIIGKIRQAHC	35
DQ410585	B	CCR5	CIRPNNNTRKSTPMGPGGAWFTTGEIIGDIRKAYC	35

DQ410596	B	CCR5	CTRPNNNTRTSIPMGPGKVFFATEIIGNIRQAHC	34
KX156472	B	CCR5	CTRLNNNTRKSIPIGPGRAFYATGDIIGDIRKAHC	35
KX156483	B	CCR5	CTRPSNNTRKSIHMGPGRAFYTTGEIIGDIRQAHC	35
KX156523	B	CCR5	CTRPNNNTRKGIHIGPGRAFYTTGEIIGDIRKAHC	35
KX156374	B	CCR5	CTRPNNNTRKSIHMGPGRAFYATGDIIGDIRQAYC	35
FJ469703	B	CCR5	CTRPNNNTRKSIHIAPGRAFYATGEIIGDIRQAHC	35
FJ469706	B	CCR5	CMRPSNNTRKSIHIGPGRAFYTTGEIIGDIRQAHC	35
FJ469718	B	CCR5	CTRPNNNTRKSIHIGPGRAFHATGDIIGNIRQAHC	35
DQ410115	B	CCR5	CTRPNNNTRKSIPIGPGRAFYTTGAIIGDIRQAHC	35
DQ886037	B	CCR5	CTRPNNNTRKGIHIGPGRAFYATGEIIGNIRQAHC	35
FJ653236	B	CCR5	CTRPSNNTSQSIHIGPGRAFDATKTITGDIRQAHC	35
FJ653196	B	CCR5	CTRPNNNTRKGIHIGPGRAFYATEKITGDIRQAHC	35
FJ653597	B	CCR5	CTRPNNNTRKSIHIGPGRAFYATGEIIGDIRQAHC	35
FJ653474	B	CCR5	CTRPNNNTRKGIHIGPGRAFYATQDIIGDIRKAYC	35
FJ653390	B	CCR5	CTRPNNNTRKSIHIAPGRAFYATGDIIGDIRQAHC	35
FJ653478	B	CCR5	CIRPNNNTRKSISLGPGRAVFTTGDIIGDIRQAHC	35
FJ653547	B	CCR5	CIRPNNNTRKSIHIGPGRAWYATGEIIGDIRQAHC	35
HQ217476	B	CCR5	CTRPNNNTRKSIHMGPGRAFYATGGIIGDIRRAHC	35
HQ217818	B	CCR5	CTRPSNNTRKGIHIGPGRAFYTTGEVIGDIRQAHC	35
GU330736	B	CCR5	CTRPNNNTRKGIHIAPGGAIYATGDIIGDIRQAHC	35
GU330809	B	CCR5	CVRPNNNTRKSIHMGPGKSFLATGDIIGDIRQAYC	35
GU330839	B	CCR5	CTRPNNNTRKSIHIGPGRAFYATEGIIGDIRQAHC	35
JF689862	B	CCR5	CTRPNNNTRKGIHMGLGKAFYATGDIIGDIRQAHC	35
JF689863	B	CCR5	CTRPDNNTRKSINIGPGRAFVTTGDIIGNIRLAHC	35
JF689866	B	CCR5	CTRPNNNTRKSINIGPGRAFYAQGEVIGDIRQAHC	35
JF689870	B	CCR5	CTRPNNNTRKSIPIGPGRAFYTTGEIIGDIRRAHC	35
JF689871	B	CCR5	CTRPNNNTRKGVHIGPGRTFYATGDIIGDIRQAHC	35
JF689872	B	CCR5	CTRPNNNTRKSIPIGPGRAWYATGNIIGDIRQAYC	35
JF689873	B	CCR5	CTRPSNNTRKSIHMGPGKAFYTTGDIIGNIRQAYC	35
JF689876	B	CCR5	CMRPNNNTRKSINMGPGRAFYATGDIIGDIRQAHC	35
HQ216908	B	CCR5	CTRPSNNTRKSIHIGPGRAFFATGEIIGDIRQAHC	35
HQ216925	B	CCR5	CTRPNNNTRRSIPIGPGSAFYTADIIGDIRQAHC	34
FJ152546	B	CCR5	CTRPANNTRKSIPMGPGRAFFATGDIIGNIRKAFC	35
FJ152547	B	CCR5	CIRPNNNTRRSVHIGPGSAFYTTGEIIGNIRQAHC	35
JX863987	B	CCR5	CTRPNNNTRKGIHIGPGSAFYATGDIIGDIRQAHC	35
JX863988	B	CCR5	CTRPNNNTRKGIHMGPGKTFFTTDIIGDIRQAHC	34
JX863991	B	CCR5	CTRPNNNTRKGINIGPGRAVYAIGEIIGDIRQAHC	35
JX863992	B	CCR5	CIRPNNNTRQGIHIGPGRAFYATGDIVGDIRQAHC	35
JN562781	B	CCR5	CTRPNNNTRRGINLGQGRAWYATTDIVGDIRQAHC	35
JF320615	B	CCR5	CTRPNNNTRKSIHIGPGKAFYTTGDIIGDIRQSHC	35
JF320613	B	CCR5	CTRPNNNTRKSISIGPGRAFYTTGNIIGDIRQAHC	35
JF320048	B	CCR5	CMRPSNNTRKSITIGPGRAFYTTGEVTGDIRQAHC	35
JF320308	B	CCR5	CTRPNNNTRRSITIGPGKAFYATNIVGDIRQAYC	34
JF320003	B	CCR5	CTRPNNNTRRSIPIGPGRAFYATGDIIGDIRQAHC	35
JF320097	B	CCR5	CTRPNNNTRKSIPIGPGKAFFATGEIIGDIRQAHC	35
JF320038	B	CCR5	CTRPNNNTRKSIPLGPGRAFFTTGEIIGDIRKAHC	35
JF320526	B	CCR5	CTRPSNNTRKSITIGPGRAFYTTGDIIGDIRRAHC	35
JF320160	B	CCR5	CTRPNNNTRRSIHMGPGRAFYATGDIIGDIRQAHC	35
JF320169	B	CCR5	CTRPNNNTRKSIHIAPGRTFYATGEIIGDIRQAHC	35
JF320053	B	CCR5	CTRPNNNTRKSIHIGPGRAFYATGEIIGDIRQAHC	35
JF320356	B	CCR5	CIRPNNNTRKGIHMGPGRAFYTTGDIIGDIRQAHC	35
JF320592	B	CCR5	CTRPNNNTRKSIHMGWGRAFYTTGEIIGNIRQAHC	35
KT124791	B	CCR5	CIRPNNNTRKSIPIGPGRAFYATGGIIGDIRQAHC	35
KR423026	B	CCR5	CTRPNNNTRKSINIGPGRAFYATGDIIGDIRQAHC	35
GU331039	B	CCR5	CTRPSNNTRKSIHIGPGRAFYATGEITGDIRQAHC	35
GU331147	B	CCR5	CIRPNNNTRRSIHIGPGRAFYGTGDIIGNIRQAYC	35
KM353701	B	CCR5	CMRPNNNTRKGIHIGPGRAFYATGEIIGDIRQAHC	35
JN687749	B	CCR5	CTRPNNNTRKGIHIGPGRAFYATGEIIGDIKRAHC	35
JN687758	B	CCR5	CIRPGNNTRKSIPIGPGRAFFATGDVIGDIRKAHC	35
HQ908116	B	CCR5	CTRPNNNTRKSIHIAPGSAFFATGEIIGDIRQAYC	35
JQ251004	B	CCR5	CTRPNNNTRKSIHIGPGRAFYATGGVIGDPRQAHC	35
JN944897	B	CCR5	CTRPNNNTRKSIHIGPGRAFYATGNIIGDIRKAYC	35
JN944907	B	CCR5	CTRPNNNTRKSITIGPGRAFYATGDIIGDIRQAHC	35
FJ469691	B	CCR5	CIRPNNNTRRSINMGPGRAFYATGEIIGDIRQAHC	35
FJ469696	B	CCR5	CTRPNNNTSKSISIGPGRAFYAREKIIGPIRQAHC	35
FJ469700	B	CCR5	CTRPNNNTRKSIPMGPGKFYATGDIIGDIRKAHC	34
FJ469708	B	CCR5	CTRPNNNTRKSIHIASGRAFYATGEIIGDIRQAHC	35
FJ469709	B	CCR5	CSRPNNNTRKSTHIGPGAALYTTGEIIGDIRQAHC	35
JQ250880	B	CCR5	CTRPNNNTRKSIHIGPGRAFDATGEIIGDIRQAHC	35
HQ217266	B	CCR5	CTRLNNNTRKSIPIGPGKALYATGEIIGDIRQAHC	35

JQ403068	B	CCR5	CTRPNNNTRKSIHIGPGRAFYATGDIIGDIRQAHC	35
JQ403095	B	CCR5	CTRPNNNTRRSINIGPGRAFYATGQIIGDIRQAHC	35
KM259364	B	CCR5	CTRPHNNTLKGIHIGPGRAFYATGSIIGNIRQAHC	35
KM259479	B	CCR5	CTRPNNNTRKSITIGPGSAFYATGEVIGNIRQAYC	35
EU578667	B	CCR5	CTRPNNNTRKSIPIGPGRAFYATGDIIGDIRRAYC	35
HQ217967	B	CCR5	CTRPYNNTRKSIPMGPGKAFYATGEVIGDIRQAQC	35
HQ218020	B	CCR5	CTRPNNNTRRSIPIGPGRAFYGMGDIIGDIRQAHC	35
HQ218034	B	CCR5	CTRPNNNTRKSIHIAPGRAFYATGEIIGDIRQAHC	35
GU330865	B	CCR5	CIRPGNNTRKSITIGPGSAFYTTDIIGDIRRAHC	34
JF689877	B	CCR5	CERPNNNTRKGIHIGPGRAFYATGEIIGDIRQAHC	35
JF689883	B	CCR5	CTRPNNNTRKSIHIGPGGAFYATGAIIGNIRQAHC	35
JF689885	B	CCR5	CVRPNNNTRKSIHIGPGRAFFATESIIGNIRKAHC	35
JF689890	B	CCR5	CTRPGNNTRKSINIGPGRAFYATGDIIGDIRKAHC	35
JF689893	B	CCR5	CIRPNNNTRKSIHLGPGGAFYTTGQIIGNIRQAHC	35
MH746265	B	CCR5	CTRPNNNTRKSIHIGPGRAFYATGDIIGDIRQAHC	35
JX863995	B	CCR5	CTRPSNNTSTGIHIGPGRAFYATERIIGDIRQAHC	35
JX863921	B	CCR5	CTRPANNTRKSIRIGPGSTFYAHGDIIGDIRQAHC	35
JX864007	B	CCR5	CTRPNNNTRKGINIGPGRAFYTIGEVIGDIRQAHC	35
JX864008	B	CCR5	CTRPNNNTRKSISIGPGRAIYATGDIIGDIRQAHC	35
JX864020	B	CCR5	CVRPGNNTRTSIHMVPGKAFYARKAFYPTSGIIGDTR	41
JF320375	B	CCR5	CTRPNNNTRKSIHIGPGSAFYATGDIIGDIRQAHC	35
JF320563	B	CCR5	CTRPGNNTRKSIHIGPGRAFYATGDIIGDIRKAHC	35
JF320045	B	CCR5	CTRPNNNTRKSIHITPGRSFFATGEIIGDIRRAYC	35
JF320173	B	CCR5	CTRPNNNTRKSIHIGPGRAFYTTGEIIGDIREAHC	35
JF320387	B	CCR5	CTRPNNNTRRSVPVGPGKALYTTDIIGDIRQAHC	34
JF320117	B	CCR5	CTRPNNNTRKSINIGPGRAFYATGDIIGDIRQAHC	35
JF320182	B	CCR5	CTRFNNNTRKSIPIGPGRAFYATGDIIGDIRVAHC	35
KX156431	B	CCR5	CIRPNNNTRKSIPIGPGRAIYATGEIIGDIRQAHC	35
HQ908150	B	CCR5	CTRPSNNTRKSIHIAPGRAFYATDIIGDIRQAHC	34
HQ217045	B	CCR5	CIRPNNNTRKSVRIGPGAAFYTTGEIIGDIRQAHC	35
HQ908184	B	CCR5	CTRPNNNTRKGIHIGPGRAFYATGDIIGNIRQAYC	35
KM353733	B	CCR5	CTRPNNNTRKSINIGPGRAFYATGDIIGDIRQAHC	35
KM354114	B	CCR5	CTRPGNNTGRGIPIGPGGAFYNTESIIGDIRKAHC	35
HQ217096	B	CCR5	CTRPNNNTRRSITIGPGRAFYGTDIIGDIRKAHC	34
HQ217102	B	CCR5	CVRPNNNTRKGIHIGPGEAIYTTGDIIGDIRQAHC	35
JN687761	B	CCR5	CIRPSNNTRKSIHMGPGRVLYATGEIIGDIRQAHC	35
JN687762	B	CCR5	CTRPNNNTRKSIHMGPGRAFYTTGDIIGDIRQANC	35
HQ217115	B	CCR5	CTRPNNNTRKSMHIGPGSAFYATGAIIGDIRQAHC	35
KM259082	B	CCR5	CTRPNNNTRRGIHLGPGGAIYATGDIIGNIRQAHC	35
HQ908139	B	CCR5	CTRPNNNTRKGISIGPGRAFYATGDIIGDIRQAHC	35
HQ217160	B	CCR5	CTRPNNNTRRGIPIGPGRVFYTSEIIGDIRQAHC	34
FJ469689	B	CCR5	CTRPSNNTRTSINIGPGRAWYTTGQITGDIRLAYC	35
FJ469707	B	CCR5	CTRPNNNTRKGINIGPGRAWYATGEITGDIRKAHC	35
FJ469721	B	CCR5	CTRPNNNTRKSIHIGPGRAFYATGDIIGNIRQAHC	35
MN516423	B	CCR5	CTRPSNNTRKSIHIQPGGAFYATGEIIGDIRKAYC	35
HQ217308	B	CCR5	CTRPNNNTRKSIHTGPLRAIHLTGNIIGDIRQAHC	35
JQ403058	B	CCR5	CVRPNNNTRKSIPMGPGRSFYATRNITGDTRNAHC	35
JQ403059	B	CCR5	CTRPSNNTRKSIHIGPGRAWFATGEITGDIRQAHC	35
JQ403060	B	CCR5	CTRPNNNTRKSIPMGPGRAFYAMGEIIGDIRQAHC	35
JQ403064	B	CCR5	CTRPNNNTRRGIHFGPGKTFYATGEIIGNIRQAHC	35
JQ403077	B	CCR5	CTRPNNNTRKGIHIGPGRAFYATESISGDIRQAHC	35
JQ403092	B	CCR5	CTRPSNNTRKGIHLGPGQAIWATGQVVGDIRQAHC	35
JQ403093	B	CCR5	CTRPNNNTRKSVNIGPGRAWFATGEVIGDIRQAHC	35
HQ217347	B	CCR5	CTRPTNNTRTSVHIGPGRAFFATGDIIGDIRDAHC	35
HQ217386	B	CCR5	CTRPNNNTRKGIHIGPGIAFYATQDIIGDIRQAHC	35
KC312583	B	CCR5	CVRPNNNTRKSIRIGPGSAFYAAGEIIGNIRQAHC	35
HQ217430	B	CCR5	CTRPNNNTRQGIHIGPGGALYTTKVIGNIRQAYC	34
KM259566	B	CCR5	CIRPGNNTRRSIQMGPGAAIYATGQIIGDIRQAHC	35
HQ217509	B	CCR5	CTRPNNNTRRSITIGPGKAFYTTDIIGDIRQAHC	34
HQ217601	B	CCR5	CTRPNNNTRKSVHMVPGGAIYATGDIIGDIRQAHC	35
GQ256646	B	CCR5	CTRPNNNTREGINIGPGRAWYATTDVIGDIRQAHC	35
JF689895	B	CCR5	CTRPNNNTRRGIHIGPGKAFYATGDIIGDIRQAHC	35
JF689896	B	CCR5	CSRPNNNTRKSIHIGPGRAFYTTGEIIGDIRQAHC	35
JX864023	B	CCR5	CTRPNNNTRKSIHMGPGSTIYATGDIIGDIRQAHC	35
JX864024	B	CCR5	CTRPNNNTRKSITIGPGQAWYATGDIIGDIRQAHC	35
JX864025	B	CCR5	CTRPNNNTRKGIHIGPGRVFYAAGEIIGDIRQAHC	35
HM638517	B	CCR5	CTRPNNNTRKSITIGPGRAFYATGDIIGDIRQAHC	35
MF499166	B	CCR5	CTRPNNNTRKSIPIGPGRAWYATGEIIGDIRQAHC	35
HM638584	B	CCR5	CTRPNNNTRKGIHLGPGSAIYATGAIIGNIRQAHC	35
KM354155	B	CCR5	CTRPNNNTRKSIQMGPGKAFFTTGDIIGDIRQAHC	35

KM354232	B	CCR5	CTRPNNNTRKSIHIAPGRAFYATGDIIGDIRQAHC	35
KM354355	B	CCR5	CTRPNNNTRKSIHMGPGGAFYATGEVIGDIRQAYC	35
JN687773	B	CCR5	CTRPYNNTRRSIPIGPGRAFYATGEVIGNIRKAYC	35
HQ908219	B	CCR5	CTRPNNNTRKSIHLGPGSAIYATGQIIGDIRQAHC	35
KY112149	B	CCR5	CTRPNNNSRKSITIGPGKAFYATGGIVGDIRQAHC	35
KY112490	B	CCR5	CTRPNNNTRTSITIGPGRAFYATGDIVGDIRQAHC	35
JQ403067	B	CCR5	CTRPGNNTRKSIHIGPGKAFYTTGAIIGDIRQAHC	35
JQ403069	B	CCR5	CMRPSNNTRRGIHIGPGGALYTTGEITGDIRRAHC	35
JQ403082	B	CCR5	CARPNNNTRKGIHIGPGGALYATGQIIGNIRQAHC	35
JQ403035	B	CCR5	CTRPNNNTRKGIHLGPGQTFYATGAIIGDIRQAHC	35
JQ403084	B	CCR5	CTRPHNNTRRSIHMGPGRALFATGDITGDIRQAHC	35
JQ403086	B	CCR5	CTRPGNNTRRGIHLGPGRTIYATGAIIGDIRQAHC	35
JQ403087	B	CCR5	CTRPNNNTRRSISIGPGRAFFTTGDVIGDIRKAQC	35
JQ403088	B	CCR5	CTRPSNNTRKSITIGPGRAFYATGQIIGDIRKAHC	35
JQ403089	B	CCR5	CTRPNNNTRKSITVGPGRAFYTTGDIIGDIRQAHC	35
JQ403091	B	CCR5	CSRPNNNTRKSIPLGPGKAFYTTGDIIGDIRQAHC	35
JQ610123	B	CCR5	CTRPNNNTRKSINIGPGRAFYATGEIIGNIRQAHC	35
KM081848	B	CCR5	CTRPGNNTRRSIHIGPGRAFYTTGEIIGDIKQAHC	35
KM081861	B	CCR5	CTRPNNNTRKSIHIAPGRAFFATGEIIGNIRQAYC	35
KM081893	B	CCR5	CIRPNNNTRRGIHIGPGGAFYTTGQIIGDIRQAHC	35
KM081963	B	CCR5	CTRPNNNTRKSIHIAPGRAFYAHTNIIGDIRQAHC	35
MH666175	B	CCR5	CIRPGNNTRKSIHIGPGRAFYATGEIIGNIRQAHC	35
MH666186	B	CCR5	CTRPSNNTSKGIHIGPGRTFYATGRITGDIRQAHC	35
MH666216	B	CCR5	CTRPNNNTRKSIHIAPGRAFYATEAVIGNIRKAYC	35
MH666227	B	CCR5	CTRPNNNTRKSIHIGPGRAYFRTGDIIGDIRQAHC	35
MH666240	B	CCR5	CTRPNNNTRKGIHIGPGRAFYATGEVIGDIRQAHC	35
MH666253	B	CCR5	CTRPGNNTRRSINIGPGRAFYATGDIIGDIRQAHC	35
MH666262	B	CCR5	CMRPGNNTRRSISIGPGRAFYATGDIIGDIRQAHC	35
KM354431	B	CCR5	CTRPNNNTRKGIGIGPGSIFYATGAIIGDIRQAHC	35
KM354585	B	CCR5	CIRPNNNTRTSIPIGPGRTIYATGEIIGDIRQAHC	35
KM354670	B	CCR5	CTRLNNNTRKSIHIGPGRAFYATGQIIGDIREAHC	35
KX587204	B	CCR5	CTRPNNNTRKSINIGPGRAWYATGQIIGDIRQAHC	35
KC473824	B	CCR5	CIRPNNNTRKSIHLGPGRAFYATGEIIGDIRKAHC	35
MK164663	B	CCR5	CTRPNNNTRKSIHIGPGRAFYATGEIIGNIRKAHC	35
MK164670	B	CCR5	CIRPNNNTRKSIPIGPGRALYATGEIIGNIRQAHC	35
KC935958	B	CCR5	CTRPNNNTRKSIHLGPGRAFYATEGIIGNIRQAHC	35
JF680914	B	CCR5	CTRPNNNTRKSIHIGPGRAFYTTGDIIGDIRQAHC	35
JF680916	B	CCR5	CTRPNNNTRKGIHIGPGRAFYATGDIIGDIRQAHC	35
KF526141	B	CCR5	CTRPNNNTRKSISIGPGRAFYATGDVIGDIRQAHC	35
KM081852	B	CCR5	CTRPNNNTRKSINIGPGRAWHISEPIIGNIRQAHC	35
KM082074	B	CCR5	CTRPGNNTRKSIHIGPGRAFYTTGDIIGDIRQAHC	35
MG196787	B	CCR5	CTRLNNNTRKSIHMGPGRAFYATGDIIGDIRQAHC	35
MG196997	B	CCR5	CVRPNNNTRKSIPIGPGRAFYATDIIGDIRQAHC	34
KM354703	B	CCR5	CTRPNNNTRKSIHIAPGKAFYATDRIIGDIRQAHC	35
KM354781	B	CCR5	CTRPNNNTIKGIHMGPGRAFYATGSIIGDIRQAHC	35
JN397365	B	CCR5	CVRPNNNTRRSMTIGPGRAFYATGDIIGDIRQAHC	35
HQ846901	B	CCR5	CTRPNNNTRKSIPIGPGRAFYTTGEIIGDIRQAHC	35
KC473825	B	CCR5	CTRPGNNTRKSIHIGPGRAFYATGDIIGDIRKAHC	35
KC473827	B	CCR5	CTRPGNNTRKSIHLGQGRAWYATGDIIGDIRQAHC	35
KU678074	B	CCR5	CTRPNNNTRKSITIGPGRAFYTTGEIIGDIRQAHC	35
KM082156	B	CCR5	CTRPNNNTRKGIHIGPGRAFYATDIIGDIRQAHC	34
KF526228	B	CCR5	CIRPQNNTRKGIHIGPGRAFYTTGEIIGDIRQAHC	35
KF526265	B	CCR5	CTRPNNNTSKSIHIGPGRAFHTTGRIIGDIRQAHC	35
KT124766	B	CCR5	CTRPNNNTRKSIRIGPGSAFYATGEIIGKIRQAHC	35
KT124773	B	CCR5	CTRPGNNTRKSIPIGPGRAFYATGDIIGDIRQAHC	35
KT124775	B	CCR5	CTRPNNNTRKSIHIGPGRAWFATGDIIGDIRQAHC	35
MG196689	B	CCR5	CTRPNNNTRKSINIGPGRAFYATGDIIGDIRQAHC	35
MG196815	B	CCR5	CIRPSNNTRKSIHIGVPGAAFYATEDIIGDIRQAHC	36
MG196840	B	CCR5	CTRPSNNTRKGFHAGPGKILYATGEIIGDIRQAHC	35
MG196846	B	CCR5	CIRPNNNTRKSINMGPGRAFYATGDIIGDIRQAHC	35
MG196942	B	CCR5	CTRPNNNARKSVHIGPGKAFFATGEIIGDIRQAHC	35
MG197084	B	CCR5	CTRPGNNTRKSIQIGPGRAFYATGDIIGDIRQAHC	35
KM354470	B	CCR5	CTRPGNNTRRSIHIGPGRAFYATGGIIGDIRQAHC	35
KM354904	B	CCR5	CTRPGNNTRKSIHIGPGRAFYATGDIIGDIRKAHC	35
KM355003	B	CCR5	CIRPNNNTRKSIHIGPGRAFYTTGDIIGNIRQAHC	35
KM355044	B	CCR5	CTRPNNNTRRSINMGPGRAFYTTGDIIGDIRQAHC	35
KU901727	B	CCR5	CTRPNNNTRKSIPMGPGKAIYATEQIIGDIRQAHC	35
KF384805	B	CCR5	CTRPSNNTRKGIHIGPGRAFFATGDIIGDIRRAHC	35
KC473831	B	CCR5	CTRPNNNTRRSIHLGPGKAIYTTGEIIGDIRRAHC	35
KC473833	B	CCR5	CVRPNNNTRQGIHMGPGRTFYTTGGIIGDIRQAYC	35

KC473834	B	CCR5	CTRPNNNTRKSINIGPGRAFYTTGDIIGDIRQAHC	35
JN397362	B	CCR5	CTRPSNNTRTSISMGPGRAFYATGSIIGDIRQAHC	35
KF384810	B	CCR5	CTRHNNNTRKSIHIGPGSAFYATGAIIGDIRQAHC	35
KM516891	B	CCR5	CTRPNNNTRKDIHIGPGRAMYATGAIIGNIRQAHC	35
KF384812	B	CCR5	CTRPNNNTRRSIHIGPGRAFYATGEIIGDIRQAHC	35
KF384813	B	CCR5	CTRPNNNTRKSISIGPGRAFYTTGEVIGDIRQAHC	35
MK383400	B	CCR5	CTRPGNNTRKSISLGPGSAIYTTGQIIGDIRQAHC	35
KY778473	B	CCR5	CERPNNNTRKSIHIGPGRAFYATGEIIGNIRQAHC	35
KT124756	B	CCR5	CIRPNNNTRKSIHIGPGRAFYATDIIGDIRKAHC	34
MH897911	B	CCR5	CTRPSNNTRRSINIGPGRAFYATGQVIGDIRQAHC	35
MH897913	B	CCR5	CTRPNNNTRKSISIGLGRAFYTTGGIIGDIRQAHC	35
MH897916	B	CCR5	CTRPSNNTIKGIHMGPGKAFYTTGKVIGDIRQAHC	35
MG196702	B	CCR5	CTRPNNNTRKSVPIGFGTWYATGDIIGDIRQAHC	34
MG196732	B	CCR5	CTRPNNNTRKSIHMGPGKAFFATGDIIGDIRKAYC	35
MG196767	B	CCR5	CTRPNNNTRKSIHIGLGRAFYATGDIIGDIRQAHC	35
MG196819	B	CCR5	CTRPNNNTRRSIHIGPGRAFYATGDIIGDIRQAYC	35
MG196902	B	CCR5	CTRPNNNTRKSIHIAPGKAFYATGGIVGDIRQAHC	35
MG196952	B	CCR5	CTRPNNNTRKSIRIGPGASFYTEEIIGDIRKAYC	34
MG197164	B	CCR5	CIRPNNNTMKSIHIGPGRAFYATGEIIGDIRQAHC	35
MG197187	B	CCR5	CTRPNTNTRKSIPIGPGRAFYATGDIIGDIRQAHC	35
KT124785	B	CCR5	CKRPNNNTRKGIHTGPGKVVYATGDIIGDIRQAHC	35
KT124790	B	CCR5	CTRPNNNTRKSVHIGPGRAFYATDIIGDIRKAHC	34
KM355085	B	CCR5	CTRPNNNTRKGIPIGPGRAFYATGDIIGDIRQAHC	35
KX587079	B	CCR5	CTRPNNNTRKSIPMGPGRAFYATGDIIGDIRQAHC	35
KU901792	B	CCR5	CTRPGNNTRKSIHIGPGQAFYATGDIIGDIRQAHC	35
KU901816	B	CCR5	CTRPFNNTNKSINIGPGRVFYATERIVGDIRQAHC	35
KM986883	B	CCR5	CTRPNNNTRKSINIGPGRAFYATGDIIGDIRQAHC	35
KU901976	B	CCR5	CTRPNNNTRKGIHMGPGKAFFVTDIIGDIRQAHC	34
KY778395	B	CCR5	CTRPNNNTRKGINMGPGRAFYTTGEIIGDIRQAHC	35
MH897918	B	CCR5	CVRPNNNTRKSIHIGPGRAFYATGDIIGNIRQAHC	35
MG196711	B	CCR5	CTRPGNNTRKSIPMGPGRALYATGEIIGDIRQAHC	35
MG197127	B	CCR5	CTRPNNNTRKSISIGPGRAFYATGDIIGDIRQAHC	35
KT124796	B	CCR5	CTRPNNNTRKSIHIGPGRAFFATGDIIGDIRQAHC	35
KX505652	B	CCR5	CTRPNNNTRKSIHIGPGRTFYAAGEIIGDIRRAYC	35
KX505686	B	CCR5	CTRPNNNTRKSIHIAPGRTFYATGQIIGDIRQAHC	35
KX505707	B	CCR5	CTRPNNNTRKSINMGPGRAFFTTGEIIGDIRQAHC	35
KY057587	B	CCR5	CTRPGNNTRKGIHMGPGKVFFATDITGDIRQAHC	34
KY748513	B	CCR5	CTRPGNNTRKSITMGPGRAMYATGDIIGDIRQAHC	35
KY748576	B	CCR5	CTRPNNNTRQSIHIGPGRAFYATGDIIGDIRQAYC	35
KT284371	B	CCR5	CTRPNNNTRKSIPMGPGRAFYTTGQIIGDIRQAHC	35
KR182173	B	CCR5	CTRPNNNTRKSIPMGPGRVLYATGQVIGDIRQAHC	35
KR182183	B	CCR5	CTRPGNNTRKGIHLGPGGAFYATGGIIGNIRQAHC	35
KR182187	B	CCR5	CIRPNNNTRKGIHLGPGGSFYATGAIIGNIRQAHC	35
KR182191	B	CCR5	CTRPSNNTRKGIHLGPGGSFYATGEIIGDIRQAHC	35
KR182196	B	CCR5	CTRPNNNTRKGIHIGPGRAFYATGDIIGDIRQAHC	35
KR182341	B	CCR5	CTRPNNNTRKSIHIGPGRAFYATGEVIGNIRQAHC	35
KR182409	B	CCR5	CIRPNNNTRRSIHIGPGGAFYATGGIIGDIRKAHC	35
KR182465	B	CCR5	CTRPNNNTRKSIPIGPGSAFYATGEIIGDIRQAHC	35
KY778299	B	CCR5	CTRPNNNTRKGVHIGPGSVWYTTGEIIGDIRQAHC	35
MK384347	B	CCR5	CIRPNNNTRKSIPIGPGRAFIATGDIIGNIRQAHC	35
KX505396	B	CCR5	CIRPNNNTRKSVPIGPGRAWYATGDIIGDIRQAHC	35
KX505419	B	CCR5	CTRPNNNTRKGIHIGPGRAFYATGDIIGKIRQAYC	35
KX505435	B	CCR5	CTRPNNNTRKSIHIGPGGAFYATGDIIGNIRQAHC	35
KM354781	B	CCR5	CTRPNNNTIKGIHMGPGRAFYATGSIIGDIRQAHC	35
KX028091	B	CCR5	CTRPGNNTRRSIHIAPGRAFYANDIIGDIRQAHC	34
KX028321	B	CCR5	CTRPNNNTRKSIHIGPGQAFYATGDIIGDIRQAHC	35
KU678060	B	CCR5	CTRPGNNTGRGIHIGPGRAWYSTGNIIGNIRQAHC	35
KX595124	B	CCR5	CTRPNNNTIRDIRIGPGSFYTTGQIIGDIRKAYC	34
KY323732	B	CCR5	CIRPNNNTRKGIHIGPGGGMFYATDIIGDIRQAHC	35
KY323909	B	CCR5	CTRPNNNTRRGIHIGPGRAFYATGTIIGNIRQAHC	35
KY324122	B	CCR5	CTRPGNNTRRSITFGPGSSFYTSDIIGDIRQAHC	34
KX028240	B	CCR5	CIRPNNNTRKSITVGPGKAFYTTGDIIGDIRQAHC	35
KX505555	B	CCR5	CIRPNNNTRKSIHMGPGKAFFATDIIGDIRQAHC	34
KX587137	B	CCR5	CTRPNNNTRKGIHIGPGRAFYATGDIIGDIRQAHC	35
KY113738	B	CCR5	CMRPNNNTRRSINIGPGRAFYAAGEIIGNIRQAHC	35
KY113566	B	CCR5	CTRPNNNTRKGISIGPGAAIYATGQIIGDIRQAYC	35
KY113379	B	CCR5	CTRPGNNTRKSIHIGPGRAFYATGVIIGDIRQAHC	35
KY057390	B	CCR5	CIRPNNNTRRGIHIGPGRAFYATGDIIGDIRKAYC	35
KY748402	B	CCR5	CTRPNNNTSKSIHIGPGRAWSATGSIIGNIRQAHC	35
KY324010	B	CCR5	CTRPNNNTRKSVRIGPGASLFTTGEIIGDIRQAHC	35

KY324473	B	CCR5	CTRPNNNTRKSITIGPGRAFYATGDIIGKIRQAHC	35
MK148554	B	CCR5	CTRPNNNTRKSIHIGPGRAFYTTGEIIGNIRQAHC	35
MK114983	B	CCR5	CTRPNNNTRKSISVAGRAIYATGQIIGDIRQAHC	34
MK115065	B	CCR5	CTRPNNNTRRSIHIGPGRAFYGTDIIGDIRQAHC	34
MK115195	B	CCR5	CTRPNNNTRKSIHIGPGRAFYATGDIIGDIRKAHC	35
MK115360	B	CCR5	CTRPNNNTRKSIHIGPGKAFYATGAIIGDIRQAHC	35
MK115488	B	CCR5	CTRPNNNTMKSIPLGPGRAFYATGAIIGDIRQAHC	35
MK115745	B	CCR5	CTRPNNNTRKSIHIAPGRAFYATGEIIGDIRQAHC	35
KX587450	B	CCR5	CTRPNNNTSKSIHIGPGRAFHTLGGIIGDIRQAHC	35
MK499378	B	CCR5	CTRPGNNTRKSIHIGPGRAFYATGDIIGDIKKAHC	35
MK169493	B	CCR5	CTRPNNNTRKSIPIGPGRAFYATGDIIGDIRQAHC	35
MK169546	B	CCR5	CTRPSNNTRKSIHITPGRAFYATGEIIGDIRQAHC	35
MK169551	B	CCR5	CTRPSNNTRKSIHITPGRAFYATGEIIGDIRQAHC	35
MK169581	B	CCR5	CTRPSNNTRKSINIVPGRAIYATGEIIGDIRQAHC	35
MK169545	B	CCR5	CTRPNNNTRKGIHIGPGRAFYATGDIIGDIRQAHC	35
MK169677	B	CCR5	CTRPNNNTRRGIHIGPGRAFYTTGDIIGDIRQAHC	35
MN090376	B	CCR5	CTRPNNNTRRSIHIGPGRAFYATGDIIGDIRQAHC	35
MN090376	B	CCR5	CTRPNNNTRRSIHIGPGRAFYATGDIIGDIRQAHC	35
AY835781	B	CCR5	CTRPNNNTRKSIHIGPGRAFYTTGQIIGDIRQAHC	35
U90935	B	CCR5	CTRPNNNTRKSIHIGPGQALYATGAIIGDIRQAHC	35
M17450	B	CCR5	CTRPNNNTTRSIHIGPGRAFYATGDIIGDIRQAHC	35
KR182196	B	CCR5	CTRPNNNTRKGIHIGPGRAFYATGDIIGDIRQAHC	35
AB221005	B	CCR5	CTRPNNNTRKSINIGPGRAFYTTGEIIGDIRQAHC	35
AY357406	B	CCR5	CTRPNNNTRRSIPIGPGRAFYTTGDIIGDIRQAHC	35
AF025758	B	CCR5	CTRPNNNTRKGIHIGPGGAFYTTKIIGDIRQAHC	34
AF025764	B	CCR5	CTRPNNNTRKSIHIQPGRAFYTTGDIIGDIRQAHC	35
AF025751	B	CCR5	CTRPNNNTRKSIPIGPGRAFYTTGEIIGEIRQAHC	35
U69584	B	CCR5	CTRPNNNTRKSIHIGPGRAFYTTGEIIGDIRQAHC	35
AY835775	B	CCR5	CIRPNNNTIKGIHIGPGRAFYTTGQIVGDIRQAHC	35
KC595159	B	CCR5	CTRPNNNTRKSIHIGPGRAFYTTGEIIGDIRQAHC	35
AY426119	B	CCR5	CTRPNNNTRKSIHIGPGRAFYTTGEIIGDIRQAHC	35
AY426125	B	CCR5	CTRPSNNTRKSIHIGPGRAFYTTGEIIGDIRQAHC	35
AF025759	B	CCR5	CTRPNNNTRKSIHIGPGRAFYTTGEIIGDIKQAHC	35
AF025761	B	CCR5	CTRPNNNTGKSINIGPGGAFYTTGKIIGDIRQAYC	35
AF025749	B	CCR5	RTRPNNNTRRSINMGPGKTLYTTDIIGDIRQAHC	34
KC595159	B	CCR5	CTRPNNNTRKSIHIGPGRAFYTTGEIIGDIRQAHC	35
AF025752	B	CCR5	CTRPNNNTRKSINIGPGKALYTTGEIIGDIRQAHC	35
AF025756	B	CCR5	CIRPNNNTRKSINIGPGRAFYTTGEIIGDIRQAHC	35
M93258	B	CCR5	CTRPNNNTRKSINIGPGRALYTTGEIIGDIRQAHC	35
AY835758	B	CCR5	CTRPNNNTRKGIHIGPGRAFYTTGEVIGNIRQAHC	35
AF025755	B	CCR5	CTRPNNNTRKGIHMGPGRAFYTTGDIIGDIRQAHC	35
AF025757	B	CCR5	CTRPNNNTRKSITIGPGKAFYTTGEIIGDIRQAHC	35
AY835763	B	CCR5	CTRPNNNSRKSIHIGPGRAFYTTGEIIGDIRKAHC	35
AF025762	B	CCR5	CIRPNNNTRNSIPIGPGKAIYTTGKIIGDIRQAYC	35
AF025754	B	CCR5	CTRPNNNTRKSIPIGPGRAFYTTGEIIGNIRQAHC	35
AF128126	B	CCR5	CSRPNNNTRKSIPMGPGRAFYTTGQIIGDIRQAHC	35
AF025763	B	CCR5	CTRPNNNTRKSIPIGPGRAFYATGEIIGDIRKAYC	35
MH012793	B	CCR5	CTRPNNNTRRSIHIGPGRAFYTTGDIIGDVRKAHC	35
EU576292	B	CCR5	CTRPNNNTRKSIHIGPGRTFYTTGDIIGDIRQAYC	35
KT283697	B	CCR5	CTRPNNNTRRGIHIGPGGAFYSTGDIIGDIRQAHC	35
AY173952	B	CCR5	CTRPSNNTRKSIHIGPGRAFYTTGNIIGDIRQAHC	35
AY173953	B	CCR5	CIRPNNNTRKSIHIGPGRAIYATGGIIGDIRRAYC	35
AY173954	B	CCR5	CTRPNNNTRKSIPIGPGRAFYATGDIIGDIRQAHC	35
MH012589	B	CCR5	CTRPNNNTRRSIHIAPGRSFYATGEVIGDIRQAHC	35
MH012650	B	CCR5	CTRPNNNTRKGINIGPGRAFYAQGEIIGDIRRAYC	35
MH012925	B	CCR5	CTRPNNNTRKGVHIGPGGAFYATGEIIGDIRQAHC	35
AY835761	B	CCR5	CTRPNNNTRKSIHIHRGPGRAFYASGGIIGDIRQAHS	37
GU331649	B	CCR5	CTRPNNNTRKSITIGPGRAFYTTGQIIGDIRQAYC	35
JN944928	B	CCR5	CTRPNNNTRKSIPIGPGRAFYTTGEIIGDIRQAHC	35
MH013129	B	CCR5	CTRPNNNTRKSIHLGPGSALYTTEIIGNIRQAHC	34
U04908	B	CCR5	CTRPNNNTRKGIHIGPGRAFYTTGEVIGNIRQAHC	35
KT283706	B	CCR5	CTRPNNNTRKSIHIGPGRAFYTTGEIIGDIRQAHC	35
MH012332	B	CCR5	CTRPNNNTRKSIHIGPGRAFYATGEIIGDIRQAHC	35
MH012400	B	CCR5	CTRPNNNTRKSIHIGPGSALYTTEIIGNIRQAHC	34
MH012257	B	CCR5	CTRPNNNTRRSIHIGPGRAFYATDIIGDIRQAHC	34
MH012478	B	CCR5	CTRPNNNTRKSINIGPGSAFYATGDVIGDIRKAYC	35
MH013027	B	CCR5	CTRPNNNTSKSVHIGPGRALYATGRIIGDIRQAHC	35
DQ487190	B	CCR5	CSRPNNNTRKGIHIGPGRAFYTTGEIIGDIRKAYC	35
AY612855	B	CCR5	CTRPNNNTRKSIHMGPGAAFYARGEVIGDIRQAHC	35
AY835437	B	CCR5	CTRPNNNTRKGIHIGPGRAFYTTGQIIGDIRQAHC	35

KT283729	B	CCR5	CTRPNNNTRKSIHIAPGRTFYTTGEIIGDIRQAHC	35
DQ313246	B	CCR5	CTRPNNNTRKSINMGPGRAFYTTGEIVGDIRQAHC	35
EU289197	B	CCR5	CTRPNNNTRKSIPIGPGRAFYTTGEIIGDIRQAHC	35
JQ609870	B	CCR5	CIRPNNNTRKSIHFGPGSAFYTTGAIIGDIRQAHC	35
AY835436	B	CCR5	CTRPNNNTRKSINIGPGRAFVATGAIIGDIRQAHC	35
AY835438	B	CCR5	CTRPNNNTRKSINLGPGRAFYATGDIIGDIRQAHC	35
KT283753	B	CCR5	CIRPHNNTRKSIHIGPGRTFYATGDIIGDIRKAHC	35
EU289198	B	CCR5	CTRPNNNTRKGIHIGPGKAFYTTGEIIGNIRQAHC	35
KU678164	B	CCR5	CTRPNNNTRKSIHIGPGRAFFGTDIIGDIRKAYC	34
JN024303	B	CCR5	CTRPNNNTAKGIHIGPGRAFYTTDRIIGDIRQAHC	35
AY332237	B	CCR5	CVRPNNNTRKSINIGPGRAFYTTGEIIGDIRQAHC	35
AY308762	B	CCR5	CTRPNNNTRKSIHIGPGRAFYTTGEVIGNIRQAYC	35
AY835753	B	CCR5	CTRPNNNTRESITMGPGKAFYATGDIIGNIRKAYC	35
EU575474	B	CCR5	CTRPNNNTRKSITIGPGRAFWTTGGIIGDIRQAHC	35
JN024428	B	CCR5	CERPNNNTIKSIHLGPGRAWHATGQIIGDIRKAFC	35
AY331284	B	CCR5	CTRPNNNTRKGIHIGPGRAFYATGQIIGDIKRAYC	35
JF680930	B	CCR5	CTRPNNNTRKSIHIGPGRAFYATGEIIGNIRQAHC	35
KT124745	B	CCR5	CTRPSNNTSKSIPIGPGRAFYTTDRIVGDIRQAHC	35
JN562755	B	CCR5	CIRPNNNTRKSIHITPGKAFYATGDIIGNIRQAHC	35
JN562794	B	CCR5	CTRPNNNTRRSIHIGPGAAIYATGQIIGNIRQAHC	35
JN562773	B	CCR5	CARPNNNTRRSINIGPGRAFYTTGAIIGDIRQAHC	35
EU289189	B	CCR5	CTRPNNNTRKSIHIGPGQAFYTTGAIIGDIRQAYC	35
EF593201	B	CCR5	CTRPNNNTRKSVRIGPGQAFYATGIIGDIRQAHC	34
EU289192	B	CCR5	CMRPNNNTRKGIHIGPGGAFYATGDIIGNIRQAHC	35
EF593204	B	CCR5	CTRPNNNTRKGIHIGPGRAFYATGDIIGNIRQAHC	35
EU289195	B	CCR5	CMRPNNNTRKSINIGPGRAFYATGDIIGDIRQAHC	35
EU575815	B	CCR5	CTRPGNNTRKSIHIAPGRAFYTTGDIIGDIRQAHC	35
EU575850	B	CCR5	CTRPSNNTRKSITIGPGRAFYTTDIIGDIRQAHC	34
EF593232	B	CCR5	CTRPNNNTRKSIHIGPGRAFYATGEIIGDIRQAHC	35
JN400469	B	CCR5	CTRPNNNTRKGVHIGPEKVYFTTSIIGDIRQAHC	34
EF593304	B	CCR5	CTRPSNNTRKSISIGPGRAFYTTGEVIGDIRQAHC	35
KT124746	B	CCR5	CTRPNNNTRKSITFGPGRAFYTTGDIIGDIRKAYC	35
KT124747	B	CCR5	CTRPNNNTRKSIHIAPGRAFYATGEIIGDIRQAHC	35
EF593208	B	CCR5	CSRPNNNTRRSIHIGPGRAFYATGDITGDIRKAHC	35
AY560107	B	CCR5	CTRPNNNTRRGIHMGPGKFYATGDIIGDIRQAHC	34
AY560109	B	CCR5	CTRPNNNTRKSIHMGPGRAFFTTGDIIGDIRKAHC	35
AY560110	B	CCR5	CIRPNNNTRKSIHIAPGRAFFATGEVIGNIREAHC	35
EF175212	B	CCR5	CIRPNNNTRKSIPMGPGKAFYATGDIIGNIRLAYC	35
EU577426	B	CCR5	CTRPGNNTRRSIHMGPGKAFYTGEIIGDIRKAHC	34
EF593284	B	CCR5	CTRPNNNTRKSIHIGPGRAFYGTDIIGDIRQAHC	34
EF593310	B	CCR5	CIRPNNNTRRSITIGPGKAFYATDIIGDIRQAYC	34
EF593312	B	CCR5	CTRPNNNTRKSTPIGPGRAFYATGEVIGDIRRAHC	35
EF593314	B	CCR5	CTRPNNNTRRSINIGPGRAFYATGEIIGDIRQAHC	35
EF593316	B	CCR5	CTRPNNNTRKSISIGPGRAFYTTGDIIGNIRQAHC	35
EF593318	B	CCR5	CTRPNNNTRKSINIGPGRAFYATGDIIGDIRQAHC	35
KT124759	B	CCR5	CTRPSNNTRKSIPIGPGRAFYATGDIIGNIRQAHC	35
JN562762	B	CCR5	CTRPNNNTRKGIHMGPGGAFYTTGQIIGDIRQAHC	35
KT124776	B	CCR5	CTRPNNNTRKSISIGPGRAFYATGQIIGNIRQAHC	35
GU331247	B	CCR5	CTRLNNNTRKSIHIGPGRAFYTTGDIIGDIRQAHC	35
JN786831	B	CCR5	CTRPNNNTRKSINMGPGRAWYATGGIIGDIRQAHC	35
JQ403100	B	CCR5	CTRPTNNTSKGIHIGPGRAFYATGQIKGDIRQAHC	35
EU576617	B	CCR5	CIRPYNNTRKSIHIGPGRAFYTTGEIIGNIRQAHC	35
AY331296	B	CCR5	CTRPNNNTRKSINIGPGRAFYATGDIIGDIRQAHC	35
EF593286	B	CCR5	CTRPNNNTRKGIHIGPGGAFYGTDIIGDIRQAHC	34
EU577525	B	CCR5	CTRPNNNTRRSINIGPGRAFYATDIIGDIRQAHC	34
JQ085297	B	CCR5	CTRPNNNTRRSISIGPGRAFYATGDIIGDIRQAHC	35
JQ085295	B	CCR5	CTRPNNNTRKSIHIGPGRAFYATGDIIGDIRQAYC	35
FJ798397	B	CCR5	CTRPNNNTRKGISIGPGRAFYATGDIIGDIRKAHC	35
FJ469685	B	CCR5	CTRPNNNTRKSIHIGPGRAFYTTGDIIGDIRQAHC	35
JQ085289	B	CCR5	CTRPNNNTRKSVHIGPGRAFYATGEIIGNIRQAHC	35
JQ085293	B	CCR5	CTRPSNNTRRGIHMGPGKAFYTTGDIIGDIRKAHC	35
AF217150	B	CCR5	CTRPNNNTRRSINIGPGRALYTTGAIIGDIRKAYC	35
MH263144	B	CCR5	CTRPSNNTRKGIHIGPGRAWYTTGEIIGDIRKAHC	35
MH263362	B	CCR5	CTRPGNNTRKSIRIGPGQTFYATGDIIGDIRQAHC	35
MH263630	B	CCR5	CTRPNNNTRNSVHMGPGKAFYTTDIVGDIRQAHC	34
KX984368	B	CCR5	CTRPNNNTRKSIPIGPGRAFYATGDIIGDIRQAHC	35
KX984537	B	CCR5	CTRPNNNTRKGIHIGPGRAFYATDIIGDIRQAHC	34
KX984588	B	CCR5	CTRPGNNTRKSINIGPGRAFYATGDIIGDIRQAHC	35
AY842786	B	CCR5	CTRPNNNTRKSIHMGPGRVLYTTGGITGDIRQAHC	35
AY842808	B	CCR5	CTRPNNNTRKSIPIGPGRAMYATGDIIGDIRQAHC	35

AY842824	B	CCR5	CIRPNNNTRKSINIGPGRAMYATEQITGDIRQAHC	35
MH632956	B	CCR5	CIRPNNNTRKSIQMGPGKTFYATGTIIGDIRQAHC	35
MH632822	B	CCR5	CTRPNNNTRKSITIGPGSAFFATGDIIGDIRQAHC	35
MK114983	B	CCR5	CTRPNNNTRKSISVAGRAIYATGQIIGDIRQAHC	34
MH576262	B	CCR5	CTRPNNNTRRSIHIGPGRAFYGTDIIGDIRQAHC	34
MH576152	B	CCR5	CTRPNNNTRRSIHIGPGRAFFATGDIIGDIRQAHC	35
MK115488	B	CCR5	CTRPNNNTMKSIPLGPGRAFYATGAIIGDIRQAHC	35
MH575857	B	CCR5	CIRPNNNTRTSIHIAPGRTFYATGEIIGDIRQAHC	35
MH575796	B	CCR5	CSRPNNNTRKSIPIGPGRALYATGQIIGDIRQAHC	35
MH575728	B	CCR5	CTRPGNNTRKGIHMGPGKTLYVTGDIIGDIRQAHC	35
MH575694	B	CCR5	CSRPNNNTRKSIGIGPGRAWFATGEIIGDIRQAHC	35
MK115745	B	CCR5	CTRPNNNTRKSIHIAPGRAFYATGEIIGDIRQAHC	35
MK116058	B	CCR5	CTRPNNNTRRGIHIGPGRAFYAASDIIGNIRQAHC	35
MK116212	B	CCR5	CIRPNNNTRKSIPAGPGKVIYATGEIIGDIRQAHC	35
MH633031	B	CCR5	CTRPNNNTRKSIHIGPGRAFYATGEIIGDIRQAHC	35
MH633155	B	CCR5	CTRPNNNTRRSIHIGPGRAFYATGEIIGNIRQAHC	35
DQ127537	B	CCR5	CTRPSNNTRKSIHIGPGRTFFATEIIGDIRRAHC	34
DQ127548	B	CCR5	CTRPNNNTRKSIHIAPGRAFYATGDIIGDIRKAYC	35
GU728244	B	CCR5	CTRPGNNTRKSIHIGPGRAWYATGDIIGDIRQAHC	35
GU728376	B	CCR5	CTRPGNNTRKSIPMGPGRAFYATGEIIGDIRQAHC	35
FJ469690	B	CCR5	CTRPNNNTRKSITIGPGRAFYATGDIIGDIRKAHC	35
FJ469694	B	CCR5	CTRPNNNTRKGIHIGPGRAFYATGDIIGDIRQAHC	35
FJ469699	B	CCR5	CTRPGNNTRKSITIGPGKAFYTTEIVGDIRQAHC	34
FJ469702	B	CCR5	CTRPNNNTRKSIHIGPGSAFYAAGEIIGDIRQAHC	35
FJ469705	B	CCR5	CTRPGNNTRKSIHIGPGRAFYATGDIVGDIRQAHC	35
FJ469712	B	CCR5	CTRPNNNTRKSIPIGPGRAWYATGDIIGDIRQAHC	35
FJ469719	B	CCR5	CTRPNNNTRKGIHMGPGKAFFTTGQIIGDIRQAYC	35
FJ469726	B	CCR5	CTRPNNNTRKGIHIGPGRAFYGTDIIGNIRQAHC	34
GU728225	B	CCR5	CTRVNNNTRKSIHIGPGRTLYATGEIIGNIRLAHC	35
MN515912	B	CCR5	CTRPNNNTRKGIHMGLGRTFYATGDIIGDIRQAHC	35
MN515943	B	CCR5	CTRPNNNTRKSIHIGPGSAFYTTGGIIGDIRQAHC	35
MN515787	B	CCR5	CIRPNNNTRKSIHLGPGRVFYATGDIIGDIRQAHC	35
KC595160	B	CCR5	CTRPNNNTRKSIHIGPGRAFYTTGEIIGDIRQAHC	35
GU728304	B	CCR5	CTRPNNNTRKSIHMGPGKAFFATDIIGDIRQAHC	34
GU728410	B	CCR5	CTRPNNNTRKSIPIGPGKAIYVTGQIIGDIRQAHC	35
GU728295	B	CCR5	CTRPNNNTRRSIPMGPGKVFYATEIIGDIRQAHC	34
GU728383	B	CCR5	CTRPNNNTRKGIHIGPGKSFYATGDIIGDIRQAHC	35
DQ886033	B	CCR5	CTRPNNNTRKGINIGPGRAWYATTNIIGDIRQAHC	35
GU728280	B	CCR5	CTRPNNNTRRSIHIGPGRSFYTADIIGDIRQAHC	34
GU728339	B	CCR5	CTRPNNNTRRGIHIGPGSAFFATGEVIGDIRKAHC	35
DQ222211	B	CCR5	CTRPNNNTSKSIPLGPGRAFHTTGRIIGDIRQAHC	35
GU367407	B	CCR5	CTRPNNNTRKSINMGPGRAFYATGDIIGDIRQAYC	35
GU367408	B	CCR5	CTRPNNNTRKSIPIGPGRAFYATGHIIGDIRQAHC	35
KP754463	B	CCR5	CTRPNNNTRKSINMGPGRAFWATGDIIGDIRQAHC	35
KP754465	B	CCR5	CVRPNNNTRKSIHLGPGSTWYATGEIIGNIRQAHC	35
KP754468	B	CCR5	CTRPNNNTRKSIHIGPGRAFYTTGEIIGDIRQAHC	35
KP754470	B	CCR5	CTRPNNNTRKSVHMGPGGALYTTDIIGDIRKAYC	34
KP754472	B	CCR5	CTRPNNNTRKSIPMGPGRAFFTTDIIGDIRQAHC	34
KP754473	B	CCR5	CIRPHNNTRKSITFGPGRAFYAADQITGDIRKAHC	35
DQ444258	B	CCR5	CTRPNNNTRKGIHMGPGGALYTTGSIIGNIRQAHC	35
GU728371	B	CCR5	CTRPNNNTRKSIHIGPGRAFYTTGEIIGDIRRAHC	35
GU728322	B	CCR5	CTRPGNNTRRSINIGPGRAFYTTGAIIGDIRKAHC	35
EF440778	B	CCR5	CTRPNNNTRKDIPLAGPGRFWTTGKIIGDIRQAHC	35
GU728392	B	CCR5	CTRPNNNTRKSIHIGPGRAFYTTGDIIGDIRQAHC	35
DQ886034	B	CCR5	CTRPNNNTRKSIHMGPGRWSFHGTDIIGDIRRAHC	35
DQ886035	B	CCR5	CTRPSNNTRKSIHIAPGRVFHATGEIIGDIRKAHC	35
FJ469750	B	CCR5	CTRPNNNTRKSITIGPGRAFYATDIIGNIRQAHC	34
GU728413	B	CCR5	CTRPNNNTRRSIHIGPGRAFYATGDIIGDIRQAHC	35
GU728179	B	CCR5	CTRPNNNTRKSIHIAPGRAFYATGDIIGDIRQAHC	35
AF491737	B	CCR5	CTRPNNNTRKSIHMGPGKAFYTTGVIGDIRQAHC	34
JN002043	B	CCR5	CTRPNNNTRKSIPIGPGRAFYATGDIIGNIRQAHC	35
FJ469752	B	CCR5	CTRPYNNTRKSISLGPGRAIFATGDIIGDIRQAHC	35
GU728189	B	CCR5	CERPNNNTRKSIPIGPGRALFMQQEIIGDIRQAYC	35
KU252650	B	CCR5	CTRPNNNTRKSIHIQPGRAFYATDIIGDIRQAHC	34
GU728294	B	CCR5	CTRPNNNTRRGIPIGPGAAFYTTDIIGDIRQAHC	34
FJ469763	B	CCR5	CTRPNNNTRKGIHIGPGRAFYTTGEIIGNIRQAHC	35
FJ469766	B	CCR5	CIRPSNNTRKSIPMGPGKAFYATGDVIGDIRQAHC	35
GU728073	B	CCR5	CIRPNNNTIKSIHLGPGRAFHTTGEIRGDIRKAYC	35
GU727875	B	CCR5	CTRPNNNTRKSVTIGPGRVWYTTGQIIGNIRQAHC	35
GU728234	B	CCR5	CTRPNNNTRKSIHIGPGRAFYATGEIIGNIRQAYC	35

M95292	B	CCR5	CTRPNNNTRKSINIGPGRAIYTTGAIIGDIRQAHC	35
FJ469767	B	CCR5	CTRPNNNTRKSIPIGPGRAFYATGDIIGNIRQAHC	35
FJ469768	B	CCR5	CTRPSNNTRKSIPIGPGRAFYATGAIIGDIRQAHC	35
FJ469769	B	CCR5	CTRPNNNTRKGVHIGPGRAFYATGQIVGDIRQAHC	35
FJ469771	B	CCR5	CTRPNNNTRRGIHIGPGRAFYATGEIIGDIRQAHC	35
GU728204	B	CCR5	CTRPSNNTRKGIHMVPGGAFYATDQIIGDIRQAHC	35
GU728030	B	CCR5	CTRPNNNTRRSISFSPGKAFYATGDIIGDIRKAHC	35
GU728370	B	CCR5	CTRPNNNTRKGIHIGPGRAFYATGEIIGNIRQAHC	35
GU728321	B	CCR5	CTRPNNNTRKSIHIGPGRAFYTTGQIVGDIRQAYC	35
GU727980	B	CCR5	CIRPNNNTRKSISIGPGRAFYTTGEIIGDIRQAHC	35
GU728181	B	CCR5	CTRPNNNTRKSIPIGPGRAFYATGDIIGDIRQAYC	35
EU604556	B	CCR5	CTRPSNNTRKSINMGPGRAFYTTGEIIGDIRQAHC	35
JX503075	B	CCR5	CTRPNNNTRKGIHIGPGKVLYATGDIIGDIRQAHC	35
AY781127	B	CCR5	CTRPSNNTRKSIHIGVGRALYATGDIIGDIRQAHC	35
JN235958	B	CCR5	CTRPNNNTRKSIHIGPGRAFFTTGAIIGDIRQAHC	35
AY795905	B	CCR5	CTRPNNNTRRSISIGPGKAFYATGDIIGNIRQAYC	35
HQ595755	B	CCR5	CTRPSNNTRKSIHMGPGGAFYTTGAVIGDIRQAHC	35
HQ595755	B	CCR5	CTRPSNNTRKSIHMGPGGAFYTTGAVIGDIRQAHC	35
KF725918	B	CCR5	CTRHNNNTRKSIPMGPGKAFFATGDIIGDIRQAQC	35
KF725977	B	CCR5	CTRPSNNTRKSINIGPGRAFYATGDIIGDIRQAHC	35
KF726002	B	CCR5	CTRPSNNTIRGIHMGPGRAFYTTGTIVGDIRRAHC	35
KF726004	B	CCR5	CTRPNNNTRKGIHLGPGKTFYATGDIIGDIRQAHC	35
KF726016	B	CCR5	CTRPNNNTRKGIHIGPGKTWFATGEIIGDIRQAHC	35
KF726021	B	CCR5	CTRPNNNTRKSIHIGPGRAFYATDITGDIRKAYC	34
FJ647145	B	CCR5	CTRPNNNTRKSIHIGPGRAFYTTGEIIGDIRQAYC	35
MH234640	B	CCR5	CTRPNNNTRKSIHLGPGKAFYATGEIIGDIRQAHC	35
AY505010	B	CCR5	CTRPNNNTRKSIHIGPGRAFYTTGQIIGDIRQAHC	35
KU168257	B	CCR5	CTRPNNNTRKSISIGPGRAFYTTGDIIGDIRQAHC	35
JF680931	B	CCR5	CTRPNNNTRKSIPIGPGRAFYATGEVIGDIRQAHC	35
KC834604	B	CCR5	CTRPGNNTRKSIHMGPGSSIYATGAIIGDIRQAHC	35
KC834602	B	CCR5	CTRPNNNTRKSISIGAPGRAFYATGDIIGDIRQAHC	36
KT452273	B	CCR5	CIRPNNNTRKGIHIGPGRSFYATGDIIGNIRQAHC	35
KT452314	B	CCR5	CTRPNNNTRKSIPVGPGSSFYATGDIIGDIRQAHC	35
KT452341	B	CCR5	CTRPNNNTRKSISFGPGSTIYATGDIIGDIRQAHC	35
KT452551	B	CCR5	CTRPNNNTRKSIHIGPGRTFYATGDVIGDIRQAHC	35
KT452612	B	CCR5	CTRPNNNTRKSIHIGPGRAFYATGEVIGNIRQAHC	35
KT452359	B	CCR5	CTRPNNNTRKGIHMGPGKAFYTTGEIIGDIRQAHC	35
KT452109	B	CCR5	CTRPNNNTRKSINLGPGSAFYATGDIIGDIRQAHC	35
KT452090	B	CCR5	CTRPGNNTRRSIPMGPGKAFYATGDIIGDIRQAHC	35
KT452407	B	CCR5	CVRPNNNTRRSINIGPGRAFYGTDIIGDIRQAHC	34
KT452218	B	CCR5	CTRPGNNTRKSIHIGPGRAFYTTGDIIGDIRQAHC	35
KT452245	B	CCR5	CSRPNNNTRKSINIGPGRAFYATDIIGDIRQAHC	34
FJ952154	B	CCR5	CTRPNNNTRXSIXIGPGRAFYATGQIIGDIRQAHC	35
AF042103	B	CCR5	CTRPNNNTRKSITIGPGKAFYATXEIIGDIRQAHC	35
EF536386	B	CCR5	CTRPNNNTRRSIXXGPGXAFYATGDIIGDIRQAHC	35
KF247097	B	CCR5	CTRPNNNTSKGIHXGPXXSFYATGAIIXDIRKAYC	35
DQ322223	B	CCR5	CTRPGNNTRRSIPMGPGRAFYTTGXIIGDIRQAHC	35
KC247971	B	CCR5	CTRPNNNTRKSIPMGPGKAFYTTGDIIGDIRQAHX	35
JN251896	B	CCR5	CTRPNNNTRKGIHMGFGRTFYATGEIIGDIRQAYX	35
JX864027	B	CCR5	CTRPNXNTRKSIHLGPGKAFYATGEIIGDIRQAHC	35
MG196874	B	CCR5	CTRPNNXTRKSITMGPGRAFYATDIIGNIRQAHC	34
GU728216	B	CCR5	CTRPNNNTRRGIHIGPGAFYATGXIIGDIRQAHC	34
GU728409	B	CCR5	CTRPGNNTRRGIHLGPGKTFFFTTDIXGDIRQAHC	34
GU728054	B	CCR5	CTRLNNNTIKGIHLGPGRAFYATEAIIGDIRQAHC	35
GU728313	B	CCR5	CTRPNNNTRKSIHLGXGXAFYATGDIIGDIRQAHC	35
KJ948657	B	CCR5	CTRPNNNTRKGIHIGPGRALYTTEITGDIRQAXC	34
GU728223	B	CCR5	CTRPNNNTRRGIHIAPGAAFYATXGGIIGDIRQAHC	36
MN486011	C	CCR5	CIRPGNNTRKSIRIGPGQAFYATGDIIGDIRQAHC	35
HQ912708	C	CCR5	CTRPNNNTRESIRIGPGQAFYATGDIIGDIRQAHC	35
EU191613	C	CCR5	CERPNNNTRESVRIGPGQAFYATGDIIGDIRQAHC	35
MH746230	C	CCR5	CTRPNNNTIKSMRIGPGQTFYATGEVVGNIRQAHC	35
MH746248	C	CCR5	CTRPNNNTRKSIRIGPGQTFYATGDIIGDIRQAHC	35
U39233	C	CCR5	CTRPNNNTRKSIRIGPGQILYATGDIIGDIRQAHC	35
U39237	C	CCR5	CIRPNNNTRESIRIGPGQTFYAHGAIIGDIRQAYC	35
U39239	C	CCR5	CTRPNNNTRRSIGIGPGQTFYATGDIIGDIRKAHC	35
U39246	C	CCR5	CTRPNNNTRESIRIGPGQTFYATGDIIGDIRQAHC	35
U39245	C	CCR5	CTRPNNNTRKGIGIGPGQTFFATEDIIGDIRQAHC	35
U39251	C	CCR5	CTRPNNNTRTSIRIGPGQAFYATGEIIGDIRQAHC	35
JN692434	C	CCR5	CTRPGNNTRKSIGIGPGRAFYATEDIIGDIRQAHC	35
AY727522	C	CCR5	CTRPNNNTRESIRIGPGQTFYATGDIIGDIRQAHC	35

AY727523	C	CCR5	CTRPNNNTRESIRIGPGQTFYATGDIIGDIRQAYC	35
AY727524	C	CCR5	CVRPNNNTRESIRIGPGQTFYATGDIIGNIRQAYC	35
AY727525	C	CCR5	CTRPNNNTRKSIRIGPGQTFYATGDIIGDIRQAHC	35
JX140663	C	CCR5	CTRPNNNTRKSIRIGPGQAFYATGDIVGDIRQAHC	35
KX181929	C	CCR5	CTRPNNNTRTSIRIGPGQTYYATGDIIGDIRQAYC	35
KX181930	C	CCR5	CTRFNNNTRKSVRIGPGQTFYATGEIIGNIRQASC	35
KX181931	C	CCR5	CTRPNNNTRKSIRIGPGQTFYATGDIIGDIREAHC	35
KX181932	C	CCR5	CTRPNNNTRKSIRIGPGQTFYATGDIIGDIRQAHC	35
KX181933	C	CCR5	CTRPNNNTRTSIRIGPGQAFYATGDIIGDIRQAHC	35
KX181934	C	CCR5	CTRPNNNTRKSVRIGPGQTFYATGDIIGDIRQAHC	35
KX181935	C	CCR5	CTRFNNNTRESIRIGPGQIFYATGDIIGDIRRAHC	35
KX181938	C	CCR5	CTRPNNNTRKSIRIGPGQAFYATGEIIGDIRQAHC	35
KX181939	C	CCR5	CTRPNNNTRTSIRIGPGQSFFATGDIIGDIRQAHC	35
KX181940	C	CCR5	CTRPNNNTRKSIRIGPGQTFYATGDIIGDIRQAHC	35
KU749391	C	CCR5	CTRPNNNTREGIGIGPGQTFYASRSIIGDIRQAYC	35
KT427678	C	CCR5	CTRPNNNTRTSVRIGPGQTFYATGEIIGDIRQAYC	35
KT427736	C	CCR5	CTRPNNNTRQSIRIGPGQAFYATGAIIGDIRRAHC	35
KT427800	C	CCR5	CTRPNNNTRTSIRIGPGQTFYATGDIIGDIRQAHC	35
KU749392	C	CCR5	CTRPNNNTRKSIRIGPGQAFYATNDIIGDIRQAYC	35
KU749393	C	CCR5	CTRPNNNTRESIRIGPGQVFYATGDIIGDIRSAYC	35
MK041570	C	CCR5	CTRPGNNTRTSIRIGPGQAFYATGDIIRDIRQAHC	35
KX181931	C	CCR5	CTRPNNNTRKSIRIGPGQTFYATGDIIGDIREAHC	35
MK041550	C	CCR5	CTRPNNNTRKSIRIGPGQTFYATGEIIGNIRQASC	35
MK041553	C	CCR5	CTRPNNNTRKSIRIGPGQTFYATGDIIGDIRQAHC	35
MK041561	C	CCR5	CTRPNNNTRTSIRIGPGQTFYATGDIIGDIRQAHC	35
MK041563	C	CCR5	CTRPNNNTRESIRIGPGQTFYATGDIIGDIRKAYC	35
MK041564	C	CCR5	CIRPNNNTRKSIRIGPGQAFYATGGIIGDIRQAHC	35
U52953	C	CCR5	CTRPNNNTRKSIRIGPGQAFYATGEIIGDIRQAHC	35
AF443088	C	CCR5	CTRPGNNTRRSVRIGPGQTFYATGDIIGNIRQAHC	35
AF443089	C	CCR5	CTRPNNNTRKSIRIGPGQTFYATGDIIGDIREAHC	35
AF443092	C	CCR5	CTRPNNNTRKSMRIGPGQTFYATGDIIGNIREAHC	35
AF443093	C	CCR5	CTRPNNNTRKSIRIGPGQTFYATGAIIGDIRQAYC	35
AF443095	C	CCR5	CTRPNNNTRKSIRIGPGQTFYATGDIIGDIRQAHC	35
AF443096	C	CCR5	CTRPGNNTRKSVRIGPGQTFYATGDIIGDIRQAHC	35
AF443098	C	CCR5	CTRPGNNTRKSIRIGPGQAFFATGEIIGDIRQAHC	35
AF443101	C	CCR5	CTRPNNNTRKSIRIGPGQTFYATGDIIGNTRQAHC	35
AF443104	C	CCR5	CTRPNNNTRKSIRIGPGQAFYATDAIIGDIRQAHC	35
AF443110	C	CCR5	CTRPNNNTRESIRIGPGQTFYATGDIIGDIRKAYC	35
AF443111	C	CCR5	CTRPNNNIRKSVRIGPGQAFYATGDIIGDIREAYC	35
AF443112	C	CCR5	CVRPNNNTRKSIRIGPGQTFYATGEIIGNIRQAYC	35
AF443113	C	CCR5	CTRPNNNTRRSIRIGPGQTFYATGEIIGDIRQAYC	35
KY658704	C	CCR5	CIRPNNNTRKSIRIGPGQTFYATGDIIGNIREAHC	35
KY658705	C	CCR5	CTRPGNNTRKSMRIGPGQTFYATGGIIGDIRQAHC	35
KY658706	C	CCR5	CTRPGNNTRKSVRIGPGQTFYATGEIIGDIRQAHC	35
KC154013	C	CCR5	CTRPGNNTRTSIRIGPGQTFYATGDIIGDIRKAHC	35
KC154015	C	CCR5	CTRPGNNTRKSVRIGPGQTFYATGDIIGNIRQAHC	35
KF114882	C	CCR5	CTRPNNNTRKSIRIGPGQTFYATGDIIGNIREAHC	35
KR861313	C	CCR5	CTRPGNNTRKSIRIGPGQAFYATGDIIGDIRRAYC	35
KR861315	C	CCR5	CTRPNNNTRKSMRIGPGQTFYATGEIIGDIRHAHC	35
KR861316	C	CCR5	CTRPNNNTRKSIRIGPGQTFFAEGGIIGDIRQAHC	35
KR861325	C	CCR5	CTRPGNNIRTSIRIGPGQTFFATGDIIGDIRQAYC	35
KR861326	C	CCR5	CTRPGNNTRKSIRIGPGQTFYATGDIIGDIRKAHC	35
KR861337	C	CCR5	CTRPNNNTRKSIRIGPGQAFYATGDIIGDIRQAHC	35
KR861345	C	CCR5	CMRPGNNTRKSVRIGPGQTFYATGDIIGDIREAHC	35
KR861312	C	CCR5	CIRPNNNTRKSMRIGPGQTFYATGDIIGDIREAHC	35
KR861322	C	CCR5	CARPGNNTRTSMRIGPGQTFYATGEIIGDIRQAHC	35
KR861324	C	CCR5	CTRPGNNKRKSVRIGIGPGHAFYATGDIIGDIRQAHC	37
KR861330	C	CCR5	CTRPNNNKRTSIRIGPGQTFYATGDIIGDIRQAYC	35
KR861335	C	CCR5	CMRPGNNTRKSMRIGPGQTFYATGDIIGDIREAHC	35
KR861343	C	CCR5	CTRPNNNTRKSIRIGPGQTFYATGDITGDIRQAHC	35
KR861311	C	CCR5	CTRPSNNTRKSVRIGPGQTFYATGDIIGDIRQAHC	35
KR861327	C	CCR5	CTRPGNNTRTSIRIGPGQTFYATNDIIGDIRQAHC	35
KR861344	C	CCR5	CIRPNNNTRKSIRIGPGQTFFATGEVIGEIRKAFC	35
KR861279	C	CCR5	CTRPGNNTRKSVRIGPGQTFYATGDIIGDIRAAHC	35
KR861282	C	CCR5	CTRPNNNTRKSIRIGPGQTFFATNDIIGDIRQAYC	35
KR861304	C	CCR5	CTRPGNNTRKSIRIGPGQVFYATGEVIGDIRQAHC	35
AF290028	C	CCR5	CTRPNNNTRKSIRIGPGQTFYATDIIGDIRQAYC	34
AF443075	C	CCR5	CTRPGNNTRKSVRIGPGQAFYATGDIIGDIRKAYC	35
AF443074	C	CCR5	CTRPGNNTRRSVRIGPGQAFYATGDIIGDIRAAHC	35
KY658709	C	CCR5	CIRPNNNTRRSVRIGPGQTFYATGEIIGNIREAHC	35

KY658711	C	CCR5	CTRPNNNTRKSIRIGPGQTFFATGDIIGDIRQAYC	35
AF443076	C	CCR5	CTRPNNNTRKSMRIGPGQIFYATGDIIGDIREAHC	35
AF443078	C	CCR5	CTRPNNNTRKSIRIGPGQTFYATGDIIGDIRQAHC	35
AF443079	C	CCR5	CTRPGNNTRTSIRIGPGQTFYATGDIIGDIRQAHC	35
AF443080	C	CCR5	CVRPSNNTRKSVRIGPGQTFYATGDIIGDIRQAHC	35
AF443082	C	CCR5	CTRPSNNTRKSIRIGPGQAFYATNDIIGDIRQAHC	35
AF443083	C	CCR5	CIRPNNNTRKSIRIGPGQTFYATGAIIGNIREAYC	35
AF443085	C	CCR5	CIRPNNNTRKSIRIGPGQTFYATGEIIGDIRKAHC	35
AF443086	C	CCR5	CTRPNNNTRKSMRIGPGQTFYATGEIIGDIRQAHC	35
HM215421	C	CCR5	CTRPNNNTRKSIRIGPGQTFYATGEIIGDIRQAHC	35
HQ699992	C	CCR5	CTRPNNNTRKSIRIGPGQTFYATGEIIGDIRQAHC	35
KF835515	C	CCR5	CIRPGNNTRQSIRIGPGQTFYAPGEIIGNIRQAHC	35
HQ699985	C	CCR5	CTRPNNNTRKSIRIGPGQTFYATGDIIGNIRQAYC	35
HM215411	C	CCR5	CTRPNNNTRRSIRIGPGQTFFAHGEIIGDIRQAHC	35
KC898995	C	CCR5	CTRPNNNTRKSIRIGPGQTFYATGDIIGDIRQAHC	35
KC898996	C	CCR5	CTRPNNNTRKSIRIGPGQTFYATGEIIGDIRQAHC	35
KF250403	C	CCR5	CTRPGNNTRKSIRIGPGQTFYATGDIIGDIRQAHC	35
KF250404	C	CCR5	CTRPDNNTRKSIRIGPGQMFYAAGDIIGDIRQAHC	35
KC870038	C	CCR5	CTRPSNNTRKSIRIGPGQTFYATGDIIGDIRQAYC	35
FJ388913	C	CCR5	CTRPNNNTRKSIRIGPGQAFYATGDIIGNIRQAHC	35
FJ388948	C	CCR5	CTRPGNNTRKSIRIGPGQAFYTNDIIGDIRQAYC	34
FJ388952	C	CCR5	CTRPSNNTRKSMRIGPGQTFYATGEVIGDIRQAHC	35
JF683740	C	CCR5	CIRPGNNTRKSIRIGPGQAFYATGDIIGNIRQAHC	35
JF683755	C	CCR5	CTRPGNNTRTSVRIGPGQTFYATGDIIGDIRQAHC	35
KT124786	C	CCR5	CTRPNNNTRKSVTLGPGQAIYATGDIIGDIRQAHC	35
KC473844	C	CCR5	CTRPGNNTRKSMRIGPGQTFFATGDIIGDIRQAHC	35
HQ236594	C	CCR5	CTRPNNNTRESIRIGPGQTFYATGDIIGDIRQAHC	35
HQ236601	C	CCR5	CTRPNNNTRKSIRIGPGQTFYATGAIIGDIRQAYC	35
KY496624	C	CCR5	CTRPNNNTRKSIGIGPGRAFYATGDIIGNIRQAHC	35
AY713417	C	CCR5	CTRPNNNTRQSIRIGPGQTFYATGEIIGDIRQAHC	35
KU319531	C	CCR5	CTRPGNNTRKSVRIGPGQTFYATGDIIGDIRQAHC	35
KU319532	C	CCR5	CERPKNNTRGSMRVGPGQTFYATGDIIGDIRQDHS	35
KU319534	C	CCR5	CTRPNNNTRKSIRIGPGQAFPATATIEGDIRQAHC	35
KU319541	C	CCR5	CTRPNNNTRKSVRIGPGQTFYAAGDIIGDIRQAYC	35
KU319544	C	CCR5	CTRTNNNTRISMRIRPGPTFYATGHIIRDIWNAHC	35
KU319546	C	CCR5	CTRPGNNTRKSMRIGPGQTFYATGDITGDIRQAHC	35
KU319551	C	CCR5	CMRTGNNTRESIRIGPGRAIYATGDIIGDIRQAFC	35
U46016	C	CCR5	CTRPSNNTRESIRIGPGQTFYATGDIIGDIRQAHC	35
AF219262	C	CCR5	CTRPSNNTRESIRIGPGQTFYATGAIIGDIRQAHC	35
AF219263	C	CCR5	CTRPNNNTRRSIRIGPGQVFYATGEIIGDIRQAHC	35
AF219269	C	CCR5	CIRPGNNTRKSMRIGPGQTFYATGAIIGDIRQAHC	35
AF219274	C	CCR5	CTRPNNNTRKSIRIGPGQAFYATNDIIGNIRQAHC	35
FJ977094	C	CCR5	CARPGNNTRKSVRIGPGQAFYATGDIIGDIRQAHC	35
HQ595789	C	CCR5	CIRPGNNTRKSIRIGPGQAFYATGEIIGNIRHAHC	35
JF680927	C	CCR5	CTRPGNNTRTSIRIGPGQSFHATGAIIGDIRQAHC	35
HQ385458	C	CCR5	CTRPDNNIRKSIRIGPGQAFYATGDVIGDIRQAYC	35
AF286233	C	CCR5	CTRPSNNTRKSMRIGPGQTFYATGAIIGDIRQAHC	35
AY255824	C	CCR5	CTRPNNNTRKSMRIGPGQTFYATGSIIGDIRQAYC	35
KP109481	C	CCR5	CTRPNNNTRKSIRIGPGQTFYATGDIIGNIREAHC	35
KP109484	C	CCR5	CTRPNNNTRKSVRIGPGQTFYATGEIIGDIRQAHC	35
EF469243	C	CCR5	CTRPNNNTRKSIRIGPGQTFYATGDIIGNIRQAHC	35
KF766537	C	CCR5	CIRPNNNTRKSIRIGPGQTFYATGEIIGDIRQAHC	35
KF766540	C	CCR5	CTRPYNNTRKSIGIGPGQAFYATGDIIGDIRQAHC	35
EU908218	C	CCR5	CTRPNNNTRQSIRIGPGQVFYATGAIIGDIRQAYC	35
EU521727	C	CCR5	CTRPNNNTRKSIRIGPGQTLYATGDIIGDIRQAHC	35
KC156210	C	CCR5	CTRPNNNTRKSVRIGPGQTFYATGEIIGDIRKAYC	35
KY929361	C	CCR5	CTRPNNNTRKSIRIGPGQTFYATGEIIGDIRQAHC	35
KY929362	C	CCR5	CTRPSNNTRRSVRIGPGQAFYTTGEIIGDIRQAYC	35
KY929366	C	CCR5	CTRPSNNTRKSIRIGPGQTFYAAGDVIGDIRQAQC	35
KY929368	C	CCR5	CIRPGNNTRKSIRIGPGQTFYATGEVIGDIRQAHC	35
KX069227	C	CCR5	CTRPNNNTRKSIRIGPGQTFYATGGIIGDIRQAHC	35
KX069228	C	CCR5	CVRPNNNTRKSVRIGPGQTFYATGEIIGDIREAHC	35
KX069222	C	CCR5	CTRPNNNTRKSVRIGPGQTFYATGDIIGDIRQAYC	35
KX756601	C	CCR5	CIRPNNNTRKSIRIGPGQTFYATGDIIGDIRQAHC	35
KX069219	C	CCR5	CTRPSNNTRESIRIGPGQVFYATKGIIGDIRQAHC	35
KX069221	C	CCR5	CTRPNNNTRKSIPIGPGKTFYATGEIIGDIRQAHC	35
KX069223	C	CCR5	CTRPNNNTRKSIRIGPGQAFYAMGDIIGDIREAHC	35
KX069224	C	CCR5	CTRPGNNTRKSVRIGPGQTFYATGDIIGDIRQAHC	35
KX069226	C	CCR5	CTRPSNNTRKSIRIGPGQTFFATGEVIGDIRKAHC	35
KY713230	C	CCR5	CTRPSNNTRTSIRIGPGQTFYATGDIIGDIRQAHC	35

KY713236	C	CCR5	CARPNNNTRKSIRIGPGQAFYTTGEIIGDIRQAHC	35
MN703367	C	CCR5	CTRPGNNTRQSIRIGPGQAFYATGDIIGNIREAHC	35
MN703351	C	CCR5	CERPNNNTRKSIRIGPGQTFYATGNIIGDIRQAYC	35
MN703360	C	CCR5	CIRPNNNTRKSVRIGPGQTFYATGDIIGNIREAYC	35
MN703391	C	CCR5	CTRPNNNTRTSIRIGPGQAFYATGEIIGPIRRAYC	35
AF067154	C	CCR5	CVRPNNNTRESIRIGPGQTFYATGEIIGDIRQAHC	35
JQ715368	C	CCR5	CTRPNNNTRKSIRIGPGQTFYATGDIIGDIRQAHC	35
AF286223	C	CCR5	CTRPNNNTRKSIRIGPGQAFYATNGIIGDIRQAHC	35
AF067155	C	CCR5	CTRPDNNTRKSIRIGPGQTFYATGDIIGDIRQAHC	35
AF286231	C	CCR5	CTRPNNNTRKSIRIGPGQTFYATGGIIGNIRQAHC	35
AF286232	C	CCR5	CTRPNNNTRKSIRIGPGQTFYATGDIIGDIREAHC	35
KP109486	C	CCR5	CTRPNNNTRKSIRIGPGQTFYATGDIIGNIRQTYC	35
KP109487	C	CCR5	CARPSNNTRTSIRIGPGQTFYATGAITGDIRQAHC	35
KP109488	C	CCR5	CIRPNNNTRKSIRIGPGQTFYATGDIVGDIRQAYC	35
KP109489	C	CCR5	CTRPNNNTRKSIRIGPGQTFYATGAIIGNIREAHC	35
AJ276221	C	CCR5	CTRPGNNTRQGIRIGPGQTFYATGEIIGFIRKAHC	35
EU521728	C	CCR5	CARPSNNTRKSIRIGPGQTFYATGEIIGDIRQAHC	35
EF694033	C	CCR5	CTRPNNNTRKSIRIGPGQTFYATGDIIGDIRQAYC	35
AF457054	C	CCR5	CIRPGNNTRKSVRIGPGQTFYATGDIIGDIRKAYC	35
FJ396028	C	CCR5	CTRPGNNTRTSVRIGPGQTFFATGDIIGDIRQAHC	35
KT022366	C	CCR5	CTRPNNNTRKSVRIGPGQTFYATGDIIGDIREAHC	35
KT022371	C	CCR5	CTRPNNNTRRGIGIGPGQMFFATDIIGDIREAHC	34
KU921723	C	CCR5	CIRPGNNTRRSVRIGPGQTFYATGDIIGDIRQAHC	35
KU921743	C	CCR5	CIRPGNNTRKSVPMGPGQVIYATGSIIGEIRQAHC	35
KU921790	C	CCR5	CIRPNNNTRKSVRIGPGQTFFATDIIGDIREAHC	34
KU921807	C	CCR5	CIRPGNNRRTSMRIGPGQTFFATGDIIGDIRKAYC	35
FJ866132	C	CCR5	CMRPGNNTRKSMRIGPGQTFYATGDIIGDTRQAHC	35
JN983803	C	CCR5	CIRPGNNTRKSMRIGPGQTFYATGDIIGDIRQAHC	35
JN983804	C	CCR5	CTRPNNNTRKSIRIGPGQTFYATGDIIGDIRQAQC	35
HM215360	C	CCR5	CIRPGNNTRKSVRIGPGQTFYATGEIIGDTRQAYC	35
KC894075	C	CCR5	CTRPNNNTRKSIRIGPGQTFYATGDIIGDIREAHC	35
KC894077	C	CCR5	CTRPNNNTRKSVRIGPGQTFYATGEITGDIRQAHC	35
FJ444184	C	CCR5	CTRPGNNTRRSIRIGPGQTFYATGDIIGDIRQAHC	35
FJ444251	C	CCR5	CTRPSNNTRQSIRIGPGQAFYATGDIIGDIRQAHC	35
FJ444277	C	CCR5	CARPGNNRRESVRIGPGQAFYATGDIIGDIRLAYC	35
FJ444476	C	CCR5	CIRPNNNTRKSMRIGPGQTFYATGDIIENIRQAHC	35
KC894083	C	CCR5	CIRPGNNTRKSVRIGPGQAFYATGDIIGNIRQAHC	35
KC894085	C	CCR5	CTRPGNNTRRSVRIGPGQTFYATGEIIGNIRQAYC	35
KC894087	C	CCR5	CTRPGNNTRRSVRIGPGQTFYATGEIIGNIRQAHC	35
KC894088	C	CCR5	CTRPNNNTRKGIGIGPGQTFYATGDIIGDIRQAHC	35
KC894090	C	CCR5	CIRPGNNTRKSVRIGPGQTFFATGDIIGDIRQAHC	35
KC894091	C	CCR5	CTRPSNNTRRSIRIGPGQTFYATGDIIGDIRQAHC	35
KC894093	C	CCR5	CIRPGNNTRQSIRIGPGQTFYATGDIIGNIREAHC	35
KC894094	C	CCR5	CTRPNNNTRKSVRIGPGQTFYATDIIGNIRQAHC	34
KC894095	C	CCR5	CTRPNNNTRRSIRIGPGQTFYATDIIGNIRQAYC	34
KC862818	C	CCR5	CTRPQNNTRKSVRIGPGQTFYATGEVIGDIREAHC	35
KC894098	C	CCR5	CTRPSNNTRESVRIGPGQTFYATGDIIGDIRQAHC	35
KC862876	C	CCR5	CTRPNNNTRKSMRIGPGQTFYATGDIIGDIRQAYC	35
KC894100	C	CCR5	CTRPGNNTRTSVRIGPGQTFYATGDIIGDIRQAHC	35
KC862949	C	CCR5	CVRPNNNTRTSIRLGPGQTFYATGGIIGDIRQAHC	35
KC894385	C	CCR5	CARPGNNTRKSIRIGPGQSFFATGDIIGDIRQAHC	35
KC156212	C	CCR5	CIRPNNNTRKSVRIGPGQTFYATGEIIGDIREAYC	35
KC156211	C	CCR5	CTRPGNNTRKSVRIGPGQTFYATGDIIGDIRQAHC	35
KC156213	C	CCR5	CIRPGNNTRKSIRIGPGQTYFSTGEIIGNIRQAHC	35
KC156119	C	CCR5	CTRPSNNTRKSVRIGPGQTFYATGRIIGDIREAHC	35
KC894109	C	CCR5	CTRPGNNTRKSMRIGPGQTFYATGDIIGDIRRAYC	35
KC148599	C	CCR5	CIRPNNNTRKSIRIGPGQTFYATNAIIGDIRQAYC	35
KC148663	C	CCR5	CTRPNNNTRKSIRIGPGQTFYATGDIIGDIRQAHC	35
HM204583	C	CCR5	CTRPNNNTRKSVRIGPGQTFYATGEIIGDIRQAYC	35
HM204591	C	CCR5	CIRPNNNTRKSVRIGPGQTFYATGEIIGDIREAYC	35
FJ443615	C	CCR5	CTRPGNNTRKSIRIGPGQTFYAHGIIGDIKQAHC	34
KP109520	C	CCR5	CTRPGNNTRKSMRIGPGQTFYATGDIIGDIRKAHC	35
HM070539	C	CCR5	CTRPGNNTRKSVRIGPGQTFYATGDVIGDIRQAHC	35
HQ595984	C	CCR5	CTRPGNNTRKSIRIGPGQAFYAAGEIIGDIRRAHC	35
HQ596030	C	CCR5	CIRPNNNTRKSVRIGPGQTFYATDIIGDIRQAHC	34
HM070639	C	CCR5	CTRPNNNTRESVRIGPGQTFYATGDIIGDIRQAHC	35
KF527172	C	CCR5	CTRPGNNTRKSIRIGPGQTFYAPNGIIGNIRKAHC	35
HM070807	C	CCR5	CTRPNNNTRKGIGIGPGQTLYATGEIIGDIRQAYC	35
KC156218	C	CCR5	CIRPGNNTRKSIRIGPGQVFFTATDIIGNIREAHC	35
HQ615962	C	CCR5	CIRPNNNTRTSIRIGPGQAFFATNDIIGNIRQAYC	35

KY112213	C	CCR5	CTRPSNNTRKSMRIGPGQTFYATGDIIGDIRQAHC	35
KP109521	C	CCR5	CIRPGNNTRKSIRIGPGQVFFAATDIIGNIREAHC	35
KP109522	C	CCR5	CTRPGNNTRKSIRIGPGQTFFATGEVIGDIRQAHC	35
KC634164	C	CCR5	CTRPGNNTRNSIRIGPGQSFYATEKIIGDIRQAHC	35
KC186899	C	CCR5	CIRPGNNTRKSIRIGPGQIFYATGEIIGDIRQAHC	35
KC186242	C	CCR5	CIRPNNNTRKSIRIGPGQTFYATGEIIGNIRKAYC	35
KC187207	C	CCR5	CVRPGNNTRRSIRIGPGQTFYATGEIIGDIRQAHC	35
KC186306	C	CCR5	CTRPNNNTRKSVRIGPGQTFYATGDIIGDIRQAHC	35
KC187437	C	CCR5	CTRPNNNTRKSIRIGPGQTFYATGSIIGDIREAHC	35
KC186647	C	CCR5	CTRPGNNTRRSVRIGPGQTFYATGDIIGNIRQAHC	35
KC187550	C	CCR5	CTRPNNNTRQSIRIGPGQVFYATKGIIGNIRQAHC	35
KC187376	C	CCR5	CTRPGNNTRKSMRIGPGQTFYATGDIIGDIRQAHC	35
KC187723	C	CCR5	CTRPNNNTRKSIRIGPGQTFYANDIIGDIRQAHC	34
KC186458	C	CCR5	CVRPNNNTRTSIRIGPGQTFYATGDIIGDIRQAHC	35
KC187049	C	CCR5	CTRPNNNTRKSIRIGPGQAFYATGDIIGNIRQAHC	35
KC186320	C	CCR5	CTRPNNNTRKSIRIGPGQTFYANNDIIGDIRQAHC	35
KC187482	C	CCR5	CTRPNNNTRRSVRIGPGQAFYATGDIIGDIRQAHC	35
KC186143	C	CCR5	CTRLNNNTRTSVRIGPGQTFYASEAVIGNIRQAHC	35
KC156214	C	CCR5	CTRPGNNTRKSVRIGPGQTFFATGDIIGDIRQAYC	35
KP109523	C	CCR5	CTRPNNNTRKSIRIGPGQAFFATGEIIGNIREAHC	35
KP109524	C	CCR5	CTRPYNNTRKSVRIGPGQTFYATGDIIGDIREAHC	35
KP109527	C	CCR5	CTRPSNNTRKSVRIGPGQTLFTTGEVIGNIRNAYC	35
KC634109	C	CCR5	CIRPGNNTRQSIRIGPGQTFYATGDIIGDIRQAHC	35
KC187500	C	CCR5	CARPGNNTRKSVRIGPGQTFYATGDIIGDIRQAHC	35
KC187630	C	CCR5	CTRPNNNTRQSVRIGPGRAFYTTGDIIGDTRKAHC	35
KC186203	C	CCR5	CTRPGNNTRQSIRIGPGKVFYATGGIMGNIRQAHC	35
KC186594	C	CCR5	CTRPGNNTRKSVRIGPGQTFYATGEIIGDIRQAHC	35
KC186169	C	CCR5	CTRPNNNTRKSVRIGPGQTFYATGDIIGNIRQAHC	35
KC186446	C	CCR5	CIRPNNNTRKSIRIGPGQTFYANDIIGDIRQAHC	34
KT896551	C	CCR5	CTRPGNNTRKSIRIGPGQVFYATGEITGDIRQAHC	35
MG898649	C	CCR5	CTRPGNNTRQSVRIGPGQTFYTTGDIIGNIRQAHC	35
AY713413	C	CCR5	CTRPNNNTRKSVRIGPGQTFYATGAIIGDIRQAHC	35
HM638662	C	CCR5	CTRPNNNTRKSVRIGPGQVFYATGDIIGDIRQAHC	35
HM638713	C	CCR5	CTRPSNNTRKSMRIGPGQTFYATGDIIGNIRQAHC	35
HM638770	C	CCR5	CTRPNNNTRKSIRIGPGQTFYATGGIIGNIREAHC	35
HM638848	C	CCR5	CTRPNNNTRKSVRIGPGQAFYATNEIIGDIRQAHC	35
HM638911	C	CCR5	CTRPSNNTRKSVRIGPGQTFYATGEIIGDIRQAHC	35
KC894126	C	CCR5	CTRPGNNTRKSMRIGPGQTFYATGDIIGDIRQAYC	35
HM638986	C	CCR5	CTRPNNNTRKSMRIGPGQTFFATGDIIGDIRQAHC	35
KC894128	C	CCR5	CTRPNNNTRKSMRIGPGQTFYATGEIIGDIRQAYC	35
KC863127	C	CCR5	CTRPSNNTRKSVRIGPGQTFYATGAITGDIRQAHC	35
KC894129	C	CCR5	CTRPNNNTRKSMRIGPGQTFYATGEIIGNIRQAHC	35
HM639015	C	CCR5	CTRPGNNTRKSMRIGPGQTFYATGEIIGDIRQAHC	35
HM639196	C	CCR5	CTRPNNNTRKSIRIGPGQTFYATNEIIGDIRQAYC	35
KC894132	C	CCR5	CTRPSNNTRKSIRIGPGQVFYANDIIGDIRQAYC	34
HM215361	C	CCR5	CTRPSNNTRKSIRIGPGQTFYATNAIIGDIREAHC	35
HM215362	C	CCR5	CTRPGNNTRTSMRIGPGQAFYATGDVIRDIRQAYC	35
KU341731	C	CCR5	CIRPNNNTRKSIRIGPGQTFYATGDIIGDIRQAHC	35
KU341730	C	CCR5	CARPNNNTRRSVRIGPGQTFYATGEIIGNIRQAHC	35
KU341726	C	CCR5	CIRPNNNTRKSIRIGPGQTFYATGDIIGDIRQAHC	35
KU341725	C	CCR5	CTRPNNNTRRSIRIGPGQTFYATGDIIGNIKQAHC	35
KU341723	C	CCR5	CTRPNNNTRKSIRIGPGQIFYATGDIIGDIRQAHC	35
KU341724	C	CCR5	CTRPNNNTRKSIRIGPGQTFYATGDIIGDIRQAHC	35
KJ158430	C	CCR5	CTRPSNNTRKSIRIGPGQTFFATGEIIGNIRQAHC	35
KJ158433	C	CCR5	CTRPGNNTRKSIRIGAGQTFYATGDIIGDIRQVPF	35
KJ541840	C	CCR5	CIRPGNNTRRSIRIGPGQTFYATGDITGDTRQAHC	35
KJ158435	C	CCR5	CTRPNNNTRKSIRIGPGQTFYATEDIIGDIREAHC	35
KJ541849	C	CCR5	CARPNNNTRKSVRIGPGQTFYAAGDIIGDIRQAHC	35
MF373126	C	CCR5	CIRPNNNTRKSVRIGPGQTFFATGAIIGEIRQAYC	35
MF373131	C	CCR5	CTRPTRNTRESIRIGPGQTFYATGEIIGDIRQAHC	35
KP411832	C	CCR5	CTRPNNNIRKSVRIGPGQTFYATGGIIGDIRQAHC	35
KP411833	C	CCR5	CTRPNNNTRTSVGIGPGKTFYATGDRIRNIKLAHC	35
MF373145	C	CCR5	CTRPGNNTRESIRIGPGQTFYATGAVIGDIRQAHC	35
MF373146	C	CCR5	CTRPNNNTRKGIGIGPGQTFYATNIIGDIRQAHC	34
MF373154	C	CCR5	CTRPNNNTRKSIRIGPGQTFYATGDIIGDIRQAYC	35
KP411837	C	CCR5	CIRPNNNTRKSMRIGPGQTFYATGDIVGDIRHAYC	35
MF373156	C	CCR5	CIRPNNNTRKSVRIGPGQTFYATGAIIGDIRKAYC	35
MF373157	C	CCR5	CTRPGNNTRKSVRIGPGQTLYATGAIIGDIRQAHC	35
MF373170	C	CCR5	CTRPNNNTRKSVRIGPGQTFYATGDIIGDIRQAHC	35
MF373171	C	CCR5	CTRPNNNTRKSVRIGPGQTFYATGDIIGNIRQAHC	35

MF373177	C	CCR5	CTRPNNNTRRSVRIGPGQTFYTTGDIIGDIRQAHC	35
MF373193	C	CCR5	CIRPGNNTRKSIRIGPGQAFYATGDVIGNIREAHC	35
MF373199	C	CCR5	CTRPNNNTRKSVRIGPGQAFYATGDIIGDIRRAYC	35
MH705137	C	CCR5	CTRPGNNTRKSVRIGPGQTFYATGDIIGDIRQAHC	35
MG989567	C	CCR5	CARPNNNTRKSIRIGPGQTFYAPGEIIGDIREAHC	35
HM215433	C	CCR5	CTRPGNNTRKSIRIGPGQVFYANDIIGNIRQAYC	34
HQ659585	C	CCR5	CTRPNNNTRQSVRIGPGQTFYATDSIIGDIRQAHC	35
HQ697934	C	CCR5	CTRPNNNTRKSIRIGPGQAFYATGDIIGDIRQAHC	35
HQ697983	C	CCR5	CTRPNNNTRRSMRIGPGQTFYATGDIIGNIRKAHC	35
HQ698009	C	CCR5	CIRPSNNTRKSMRIGPGQAFYATGDIIGDIRQAHC	35
HM215286	C	CCR5	CTRPNNNTRKSMRIGPGQTFYATGAIIGDIRQAYC	35
AY253304	C	CCR5	CVRPGNNTRKSVRIGPGQTFYATGDIIGDIRQAHC	35
AY253312	C	CCR5	CTRPSNNTRKSMRIGPGQTFYATGDIIGDIRQAHC	35
AY253303	C	CCR5	CKRPNNNTRKSVRIGPGQTFYATGDIIGNIREAHC	35
AY253313	C	CCR5	CTRPNNNTRKSMRIGPGQTFYATGEIIGDIRQAHC	35
AY253322	C	CCR5	CTRPGNNTRKSIRIGPGQAFFATGEVIGDIRQAHC	35
MK501601	C	CCR5	CTRPNNNTRKSIRIGPGQTFYATDIIGDIRQAYC	34
AY734550	C	CCR5	CTRPNNNTRKSIRIGPGQAFYATNGIIGDIRQAHC	35
AY734551	C	CCR5	CIRPGNNTRKSVRIGPGQAFYATGGIIGDIRQAHC	35
AY734558	C	CCR5	CTRPNNNTRKSIRIGPGQTFYATGDIIGDIRQAHC	35
AY734559	C	CCR5	CIRPNNNTIKSVRIGPGQAFYAPGDIVGDIRQAHC	35
AY734560	C	CCR5	CVRPGNNTRKSVRIGPGQTFYATGDIIGDIRQAHC	35
KX907407	C	CCR5	CTRPGNNTRQSIRIGPGQTFYATGDIIGDIRQAYC	35
KX907424	C	CCR5	CIRPNNNTRESIRIGPGQAFYATRDIIGDIRRAYC	35
KX907426	C	CCR5	CTRPNNNTRKSIRIGPGQTFYATEVIGDIRQAYC	34
KX907345	C	CCR5	CTRPNNNTRKSVRIGPGQTFYATGDIIGDIRQAHC	35
KX907384	C	CCR5	CTRPNNNTRKSIRIGPGQTFYATGGIIGNIRQAYC	35
KX907392	C	CCR5	CIRPGNNTRKSIMIGPGQAFFAHTNIIGDIRQAHC	35
KX907394	C	CCR5	CMRPGNNTRKSVRIGPGQTFYATGDIIGDIRQAHC	35
KX907395	C	CCR5	CTRPGNNTRRSVRIGPGQTFYATGAIIGDIRQAHC	35
KX907405	C	CCR5	CIRPGNNTRQSIRIGPGQAFYAYTNIIGDIRKAHC	35
KX907413	C	CCR5	CTRPNNNTRKGIRIGPGQTFYATGEIIGNIREAHC	35
KX907422	C	CCR5	CIRPNNNTRKSIRIGPGQTFYATGDIIGNIRQAYC	35
KX907338	C	CCR5	CTRPNNNTRRSIRIGPGQAFYTTGEIIGNIRKAYC	35
KX907363	C	CCR5	CTGPGNNTRKSMRIGPGQTFYATGAIIGNIRQAHC	35
KX907374	C	CCR5	CIRPNNNTRKSVRIGPGQTFYATGDIIGDIRQAHC	35
KX907416	C	CCR5	CTRPGNNTRRSIRIGPGQTFYATEVIGNIRQAHC	34
KX907375	C	CCR5	CVRPNNNTRKSIRIGPGQTFYANDIIGDIRQAHC	34
KX907390	C	CCR5	CTRPGNNTRKSIRIGPGQAFYGNDIIGNIRQAHC	34
KX907391	C	CCR5	CTRPGNNTRRSVRIGPGQTFYATGEIIGDIRQAYC	35
KX907399	C	CCR5	CTRPYNNTRKSIRIGPGQAFYATGDIIGDIRQAHC	35
KC156220	C	CCR5	CTRPGNNTRKSVRIGPGQTFYATGEIIGDIRKAHC	35
AF361874	C	CCR5	CTRPNNNTRESIRIGPGQTFYATGDIIGDIRQAHC	35
AF286234	C	CCR5	CTRPGNNTRKSVRIGPGQTFYTNDIIGDIRQAYC	34
AF286235	C	CCR5	CTRPGNNTRKSIRIGPGQTFYATGEIIGDIRQAHC	35
L22948	C	CCR5	CARPNNNTRESIRIGPGQTFYATGDIIGDIRQAYC	35
EU663619	C	CCR5	CTRPGNNTRRSTRIGPGQTFFATGDIIGDIRQAHC	35
AY444800	C	CCR5	CTRPNNNKRKSIRIGPGQTFYATGEIIGDIRQAHC	35
AY444801	C	CCR5	CTRPGNNTRKSVRIGPGQAFYATNDIIGDIRQAYC	35
AY563169	C	CCR5	CTRPNNNTRTSIRIGPGQTFYATGEIIGDIRQAHC	35
AY795906	C	CCR5	CTRPNNNTRKSMRIGPGQTFYATGSIIGDIRQAHC	35
AY529667	C	CCR5	CTRPNNNTRKSIRIGPGQTFYATGDIIGDIRQAHC	35
AY463217	C	CCR5	CTRPNNNTRKSIRIGPGQTFYTNDIIGDIRQAYC	34
AY463230	C	CCR5	CTRPGNNTRKSIRIGPGQTFYTTGEIIGDIRQAHC	35
AY463231	C	CCR5	CTRPNNNTRKSVRIGPGQTFYATGDIIGDIRQAHC	35
AY463225	C	CCR5	CTRPGNNTRKSIRIGPGQTFYATNDIIGNIRQAHC	35
AY463232	C	CCR5	CTRPNNNTRKSIRIGPGQTFFANNDIIGDIRQAYC	35
AY463218	C	CCR5	CIRPGNNTRKSVRIGPGPGQTFYATDIVGDIRKAHC	36
AY463233	C	CCR5	CTRPGNNTRKSMRIGPGQTFYATGDIIGDIRQAHC	35
AY463219	C	CCR5	CTRPNNNTRKSIRIGPGQAFYATNEIIGDIRKAYC	35
AY463220	C	CCR5	CTRPNNNTRKGIGIGPGQTFFATNAIIGDIRQAHC	35
AY463234	C	CCR5	CTRPNNNTRKSIRIGPGQAFYATNEIIGDIRQAYC	35
AY463236	C	CCR5	CIRPGNNTRTSVRIGPGQTFYATGDIIGDIRQAHC	35
AY463226	C	CCR5	CTRPNNNTRKSIRIGPGQTFYANDIIGNIREAHC	34
AY463222	C	CCR5	CVRPNNNTRKSIRIGPGQAFYTTGEIIGDIRQASC	35
JX845606	C	CCR5	CTRPGNNTRRSIRIGPGQVFYANSPIIGDIRQAHC	35
KF146947	C	CCR5	CTRPNNNTRKSVRIGPGQAFFATGEIIGDIRQAHC	35
AY838568	C	CCR5	CIRPGNNTRKSIRIGPGQTFFATNDIIGDIRQAHC	35
AY463228	C	CCR5	CTRPNNNTRKSIRIGPGQTFYATDIIGNIREAYC	34
EU161645	C	CCR5	CTRPNNNTRTSTRIGPGQAFYATGDIIGDIRQAHC	35

AY463237	C	CCR5	CTRPGNNTRKSIRIGPGQTFYANDIIGDIRQAHC	34
KC863203	C	CCR5	CTRPNNNTRKSVRIGPGQTFYATGDIIGDIRQAYC	35
AY463223	C	CCR5	CTRPNNNTRKGIGIGPGQTFFATNAIIGDIRQAHC	35
DQ351235	C	CCR5	CTRPNNNTRKSMRIGPGQTFYATGDIIGNIRLAHC	35
DQ351222	C	CCR5	CTRPNNSTRKSIRIGPGQTFYATNEIIGDIRQAHC	35
DQ351218	C	CCR5	CTRPNNNTRKSIRIGPGQTFFANSIIGDIRQAHC	34
DQ369978	C	CCR5	CTRPGNNTRKSIRIGPGQTFYANDVIGNIRQAYC	34
DQ351225	C	CCR5	CTRPNSSTRKSVRIGPGQTFYATGEIIGDIRQAYC	35
DQ396367	C	CCR5	CTRPNNNTRKSVRIGPGQAFYATKDIIGDIRQAHC	35
DQ351226	C	CCR5	CIRPYNNTRKSMRIGPGQAWFYTNDIIGDIRQAHC	35
DQ369985	C	CCR5	CTRPNNNTRKSVRIGPGQTFYATGSIIGDIRQAHC	35
DQ369996	C	CCR5	CARPGNNTRKSVRIGPGQTFYATGEIIGNIREAHC	35
DQ445633	C	CCR5	CARPGNNTRKSVRIGPGQAFYATNDIIGDIRQAHC	35
DQ369979	C	CCR5	CTRPSNNTLKSIRIGPGQVFYATGKIIGDIREAHC	35
DQ369984	C	CCR5	CTRPGNNTRKSVGIGPGQTFFATNDIIGDIRQAHC	35
DQ396364	C	CCR5	CTRPNNNTRKSIRIGPGQTFYAAGDIIGNIRQAHC	35
DQ369986	C	CCR5	CTRPHNNTRKSVRIGPGQAFYATDDIIGDIRQAHC	35
DQ369980	C	CCR5	CIRPGNNTRKSVRIGPGQTFYATGEIIGNIRLAHC	35
DQ396385	C	CCR5	CTRPNNNTRKSIRIGPGQTFYATGAIIGEIRQAHC	35
DQ369987	C	CCR5	CTRPNNNTRKSVRIGPGQTFYANTNIIGDIRQAYC	35
DQ369988	C	CCR5	CTRPNNNIRKSIRIGPGQTFYANDIIGDIRQAHC	34
DQ396373	C	CCR5	CKRPNNNTRQSIRIGPGQTFYATGAIIGDIRQAHC	35
DQ369989	C	CCR5	CIRPGNNTRKSIRIGPGQTFYATGDITRDIRNAHC	35
DQ396375	C	CCR5	CTRPNNNTRRSIRIGPGQTFYANNDIIGDIRQAHC	35
DQ396389	C	CCR5	CTRPGNNTRKSIRIGPGQTFYATGDITGDIRQAHC	35
DQ396376	C	CCR5	CTRPNNNTRKSIRIGPGQTFYATEVIGDIRQAYC	34
DQ093591	C	CCR5	CTRPNNNTRKSIRIGPGQTFFATNSIIGDIRQAHC	35
DQ351219	C	CCR5	CTRPNNNIRKSIRIGPGQTFYATGEIIGDIRQAYC	35
DQ351229	C	CCR5	CVRPNNNTRKSVRIGPGQTFYATGIIGNIRQAHC	34
DQ275654	C	CCR5	CTRPGNNTRKSIRIGPGQTFYATGDIIGDIRAAHC	35
DQ351216	C	CCR5	CTRPSNNTRKSIRIGPGQAFYATDIIGDIRQAYC	34
DQ275645	C	CCR5	CTRPGNNTRRSIRIGPGQAFYTNNIIGDIRQAHC	34
DQ351230	C	CCR5	CTRPNNNTRKSIRIGPGQAFYATGEIIGDIRQAHC	35
DQ275656	C	CCR5	CTRPNNNTRKSIGIGPGRAFYATGDIIGDIRQAHC	35
DQ445632	C	CCR5	CTRPNNNTRKGVRIGPGQTFYATGDIIGDIRQAYC	35
DQ396378	C	CCR5	CIRPGNNTRKSIRIGPGQTFYANDIIGNIREAHC	34
DQ396386	C	CCR5	CTRPGNNTRKSVRIGPGQTFYATGDIIGDIRQAHC	35
DQ396365	C	CCR5	CTRPSNNTRKSIRIGPGQTFYATNDIIGNIRQAYC	35
DQ445635	C	CCR5	CTRPNNNTRKSVRIGPGQTFYATGEIIGNIREAHC	35
DQ396368	C	CCR5	CTRPNNNTRKSIRIGPGQAFYATNDIIGDIREAHC	35
DQ396370	C	CCR5	CTRPSNNTRKSIRIGPGQTFYATDGIIGDIRQAHC	35
DQ396369	C	CCR5	CTRPGNNTRKSIRIGPGQAFYAANGIIGDIRQAHC	35
DQ369976	C	CCR5	CTRPGNNTRKSIRIGPGQTFYATGAIIGDIRQAHC	35
DQ396390	C	CCR5	CTRPNNNTRKSVRIGPGQVFYATNAIIGDPRQAHC	35
DQ275657	C	CCR5	CTRPNNNTRKSIRIGPGQTFYATNDIIGNIRQAHC	35
DQ396380	C	CCR5	CIRPNNNTRKSMRIGPGQTFYATGAIIGNIRQAHC	35
DQ275646	C	CCR5	CTRPGNNTRKSIRIGPGQTFYANNNIIGNIRQAHC	35
DQ351231	C	CCR5	CSRPSNNTRKSIRIGPGQAFYATGDIIGDIRQAHC	35
DQ369981	C	CCR5	CERPNNNTRKSIRIGPGQTFYATNEIIGDIRQAYC	35
DQ396392	C	CCR5	CIRPNNNTRTSIRIGPGQTFYATGDIIGDIRQAHC	35
DQ396399	C	CCR5	CTRPGNNTRKSIRIGPGQTFYATGGIIGDIRKAYC	35
AY901965	C	CCR5	CIRPGNNTRGSVRIGPGQTLFFTNDMIGDIRQAYC	34
DQ275660	C	CCR5	CTRPNNNTRKSVRIGPGQTFYATGDIIGDIRQAHC	35
DQ351233	C	CCR5	CVRPNNSTRKSIRIGPGQVFYATGDIIGDIRQAHC	35
AY878063	C	CCR5	CIRPGNNTRQSIRIGPGQTFFTNDIIGDIRQAHC	34
AY878065	C	CCR5	CTRPNNNTRKSIRIGPGQAFYATGAIIGDIRQAHC	35
AY878068	C	CCR5	CTRPNNNTRKSIRIGPGQTFYANNDIIGDIRQAHC	35
AY878069	C	CCR5	CTRPGNNTRKSVRIGPGQVFYATGDIIGDIRQAHC	35
AY901967	C	CCR5	CTRPNNNTRTSIRIGPGQTFYATNDIIGDIREAHC	35
AY901968	C	CCR5	CVRPNNNTRKSIRIGPGQTFFATGDIIGDIRQAHC	35
DQ164113	C	CCR5	CTRPGNNTRKSIRIGPGQTLFANDIIGDIRKAYC	34
AY901969	C	CCR5	CMRPGNNTRKSIRIGPGQTFYATGDIIGDIRKAHC	35
DQ093593	C	CCR5	CSRPNNNTRKSIRIGPGQTFFATGEIIGDIRQAHC	35
AY901970	C	CCR5	CVRPNNNTRKSIRIGPGQAFYATGDIIGDIRQAHC	35
AY901981	C	CCR5	CIRPNNNTSKGVRIGPGQTFFATDRIIGDIRQAYC	35
AY878057	C	CCR5	CTRPNNNTRKSIRIGPGQTFYATNDIIGDIRQAYC	35
AY878060	C	CCR5	CTRPGNNTRKSIRIGPGQTFYATGDIIGDIREAHC	35
DQ396395	C	CCR5	CTRPNNNTRRSIRIGPGQTFYTNDIIGDIRKAYC	34
DQ056410	C	CCR5	CIRPGNNTRKSIRIGPGQAFYANDIIGDIRKAHC	34
DQ056411	C	CCR5	CVRPNNNTRRGIGIGPGQTFFATDAIIGDIRKAYC	35

DQ056404	C	CCR5	CTRPNNNTRKSIRIGPGQTFYATGDIIGNIRQAHC	35
DQ351237	C	CCR5	CIRPGNNTRKSMRIGPGQTFYATGDIIGDTRQAHC	35
DQ056408	C	CCR5	CTRPGNNTRQSIRIGPGQAFYATGAIIGDIRRAHC	35
DQ011169	C	CCR5	CTRPGNNIRRSIRIGPGQTFFATGDITGDIRQAYC	35
DQ011176	C	CCR5	CTRPNNNTRKSVRIGPGQAFYATGDIIGNIRQAHC	35
DQ093601	C	CCR5	CTRPNNNTRKSIRIGPGQAFYANDIIGDIRQAHC	34
DQ093596	C	CCR5	CTRPGNNTRKSVRIGPGQTFYATGDIIGDIRQAHC	35
DQ093607	C	CCR5	CTRPNNNTRKSIRIGPGQTFYATGEIIGNIREAHC	35
DQ164108	C	CCR5	CTRPNNNTRKSIRIGPGQTFYATNIIGDIRQAHC	34
DQ351234	C	CCR5	CTRPGNNTRKSIRIGPGQTFYATGEIIGDIRQAHC	35
DQ275650	C	CCR5	CTRPGNNTRKSIRIGPGQTFYATGEVIGDIRQAHC	35
KT183301	C	CCR5	CTRPNNNTRKSVRIGPGQTFYATGEIVGDIRQAHC	35
KC863229	C	CCR5	CTRPGNNTRKSIRIGPGQTFYATGDIIGDIRQAHC	35
AY772690	C	CCR5	CTRPNNNTRKSVRIGPGQAFYAMGDIIGNIRQAHC	35
AY772691	C	CCR5	CTRPSNNTRKSVGIGPGQTIYATGQIIGDIRQAHC	35
AY703908	C	CCR5	CTRPHNNTREGIGIGPGQTFFVTKKIIGNIRQAHC	35
AY772693	C	CCR5	CTRPSNNTRKSIRIGPGQTFYANEIIGDIRQAHC	34
AY772700	C	CCR5	CTRPSNNTRKSIRIGPGQTFYATGAIIGDIRQAHC	35
AY772694	C	CCR5	CTRPNNNTRKSIRIGPGQTFFANNDIIGDIRQAHC	35
MK041572	C	CCR5	CTRPNNNTRKSIRIGPGQTFYATGAIIGDIRQAYC	35
AY772695	C	CCR5	CIRTGNNTRKSIRIGPGQTFFATDAIIGDIRKAYC	35
AY772696	C	CCR5	CTRPGNNTRRSIRIGPGQAFFATNAIIGDIRQAHC	35
DQ164115	C	CCR5	CTRPNNNTRKSIRIGPGQAFYATNGIIGDIREAHC	35
DQ164121	C	CCR5	CERPNNNTRRSIRIGPGQTFYATGDIIGNIRQAHC	35
DQ396393	C	CCR5	CTRPGNNTRKSIRIGPGQAFYATGDIIGDIRQAHC	35
DQ396394	C	CCR5	CTRPNNDTRKSIRIGPGQAFYATNSIIGNIRQAHC	35
DQ164122	C	CCR5	CTRPNNNTRKSIRIGPGQTFYATNIIGDIRQAHC	34
DQ164117	C	CCR5	CTRPNNNTRRSIRIGPGQAFYATNGIIGDIRQAHC	35
DQ164118	C	CCR5	CTRPNNNTRKSVRIGPGQAFYATGDIIGDIRQAHC	35
DQ445637	C	CCR5	CVRPNNNTRRSLRIGPGQTFYTNDIIGDIRQAHC	34
DQ164110	C	CCR5	CTRPNNNTRKSIRIGPGQTFYANNDIIGDIRQAHC	35
DQ093598	C	CCR5	CTRPGNNTRKSVRIGPGQTFYATGDIIGDIRQAHC	35
DQ093599	C	CCR5	CTRPGNNTRKSIRIGPGQTFYATGDIIGNIRQAHC	35
DQ164126	C	CCR5	CTRPNNNTRRSIRIGPGQAFYTTNIIGDIRKAYC	34
DQ093588	C	CCR5	CTRPGNNTRKSIRIGPGQTFYANDIIGDIRRAYC	34
DQ164119	C	CCR5	CTRPGNNTRQSTRIGPGQTFYARGDIIGDIRQAHC	35
DQ093585	C	CCR5	CTRPSNNTRRSVRIGPGQVFYTNDIIGDIRQAHC	34
DQ056412	C	CCR5	CTRPGNNTRKSIRIGPGQSFYATGDIIGDIRKAHC	35
AY878072	C	CCR5	CTRPGNNTRKSIRIGPGQAFYATNDIIGDIRQAHC	35
AY772699	C	CCR5	CTRPNNNTRKSIRIGPGQVFYTNEIIGNIRQAHC	34
DQ164114	C	CCR5	CTRPNNNSRKSIRIGPGQTFYATNAIIGDIREAYC	35
AY901977	C	CCR5	CTRPNNNTRKSIRIGPGQAFYATDGIIGNIRQASC	35
AY901978	C	CCR5	CIRPGNNTRKSIRIGPGQAFYATNDIIGDIRQAHC	35
DQ011171	C	CCR5	CTRPGNNTRKSMRIGPGQTFYATGDIIGDIRKAHC	35
DQ011179	C	CCR5	CTRPGNNTRKSIRIGPGQTFYTNDVIGDIGEAHC	34
DQ011173	C	CCR5	CIRPNNNTRKSIRIGPGQTFFATGGIIGDIRQAHC	35
DQ011170	C	CCR5	CIRPGNNTRKSIRIGPGQTFYATNDIIGDIRNAYC	35
AY901979	C	CCR5	CTRPDNNTRKSVRIGPGQVFYAKGDIIGDIRQAHC	35
DQ396387	C	CCR5	CTRPGNNTRKSIRIGPGQTFYATGDIIGDIRQAHC	35
DQ164127	C	CCR5	CTRPGNNTRRSIRIGPGQAFYATGDIIGDIRQAHC	35
AY878058	C	CCR5	CTRPNNNTRKSIRIGPGQAFYATNGIIGDIKQAHC	35
DQ396381	C	CCR5	CVRPNNNTRKSIRIGPGQAFFATTDIIGNIREAHC	35
DQ093595	C	CCR5	CTRPSNNTRKSIRIGPGQAFYATNDIIGDIRQAHC	35
DQ164129	C	CCR5	CTRPNNNTRKSIRIGPGQTFYATGKIIGDIRQAYC	35
DQ056416	C	CCR5	CTRPGNNTRKSVRIGPGQTFYPTGAIIGDIRQAHC	35
AY878062	C	CCR5	CTRPNNNTRKSIRIGPGQTFYATGDIIGDIRQAHC	35
DQ056409	C	CCR5	CTRPNNNTRKSVRIGPGQTFYATGGIIGNIREAHC	35
DQ369993	C	CCR5	CTRPGNNTRKSMRIGPGQTFYATGDIIGDIRQAHC	35
DQ396382	C	CCR5	CERPNNNTRQSVRIGPGQTFYATGDIIGDIRQAYC	35
DQ396396	C	CCR5	CIRPGNNTRQSIRIGPGQTFYATGDIIGDIRKAHC	35
DQ056413	C	CCR5	CTRPNNNTRKSIRIGPGQAFYATNDIIGNIRQAHC	35
DQ396383	C	CCR5	CIRPSNNTRKSIRIGPGQTFYATGEIIGDIRQAYC	35
DQ396397	C	CCR5	CSRPNNNTRKSIRIGPGQTFYATGEIIGNIRQAYC	35
DQ011180	C	CCR5	CTRPNNNTRKSIRIGPGQTFYATGDIIGDIREAHC	35
DQ056415	C	CCR5	CIRPGNNTRKSVRIGPGQTFYATGDIIGDIRQAHC	35
DQ056406	C	CCR5	CTRPNNNTRKSVRIGPGQTFYATNDIIGDIRQAHC	35
DQ056417	C	CCR5	CKRPGNNTRKSIGLGPGRAFFAHGDIIGDIRQAHC	35
DQ093605	C	CCR5	CTRPSNNTRKSVRIGPGQTFYATGEIIGDIRQAHC	35
GQ999973	C	CCR5	CTRPNNNTRKSIRIGPGQAFYATNDIIGDIRRAYC	35
GQ999975	C	CCR5	CVRPGNNTRKSVRIGPGQTFYATGDIIGDIRKAHC	35

AY703909	C	CCR5	CTRPGNNTRQSIRIGPGQTFYATGDIIGDIRQAYC	35
AY703910	C	CCR5	CTRPNNNTRTSVRIGPGQTFYATGDIIGNIRQAHC	35
AY703911	C	CCR5	CTRPNNNTRKSMRIGPGQTFYATGAIIGDIRQAHC	35
DQ382369	C	CCR5	CIRPGNNTRKSVRIGPGQAFYATGDIIGDIRQAHC	35
DQ382370	C	CCR5	CTRPNNNTRKSVRIGPGQTFYATGDIIGDIRQAHC	35
DQ382371	C	CCR5	CARPGNNTRRSVRIGPGQAFYATGEIIGDIRKAHC	35
DQ382375	C	CCR5	CTRPNNNTRRSIRIGPGQTFYTNDIIGDIRQAYC	34
DQ382377	C	CCR5	CTRPNNNTRKSVRIGPGQTFYATGEIIGDIRQAHC	35
DQ382361	C	CCR5	CTRPNNNTRESIRIGPGQTFFATGDIIGDIRQAYC	35
DQ382364	C	CCR5	CARPGNNTRKSVRIGPGQTFFATGDIIGDIRKAHC	35
DQ382365	C	CCR5	CTRPGNNTRKSVRFGPGQAFYATGDIIGDIRQAHC	35
DQ382366	C	CCR5	CTRHNNNTRKSVRIGPGQTFYATGDIIGDIRQAHC	35
DQ382367	C	CCR5	CTRPSNNTRKSVRIGPGQTFFATGEIIGDIRQAHC	35
DQ396372	C	CCR5	CTRPGNNTRKSVRIGPGQAFFATGEIIGDIRQAHC	35
DQ369992	C	CCR5	CTRPNNNTRKSVRIGPGQAFYTNDVIGDIRKAHC	34
DQ369982	C	CCR5	CTRPNNNTRKSVRIGPGQTFYATGDIIGDIRQAHC	35
DQ369983	C	CCR5	CTRPGNNTRKSIRIGPGQTFFTHDIIGDIRKAYC	34
DQ369994	C	CCR5	CTRHSNNTRKSIRIGPGQTFYATGDIIGDIRLAHC	35
HQ615948	C	CCR5	CTRPSNNTRESIRIGPGQTFYATGNIIGDIRQAHC	35
JQ754193	C	CCR5	CTRPNNNTRKSVRIGPGQTFYATGDIIGNIRQAHC	35
GQ999981	C	CCR5	CTRPGNNTRKSIRIGPGQTFYANNDIIGDIRQAHC	35
KC894135	C	CCR5	CTRPNNNTRGSIRIGPGQTFYATKDIIGDIRQAHC	35
GQ999983	C	CCR5	CIRPGNNTRRSIRIGPGQAFYAMGDIIGNIREAHC	35
GQ999984	C	CCR5	CTRPNNNTRKSIRIGPGQAFYTNEIIGDIRQAHC	34
GQ999989	C	CCR5	CTRPNNNTRKSVRIGPGQSFYATGDIIGNIRQAHC	35
GQ999990	C	CCR5	CTRPNNNTRKSIRIGPGQTFYATGDIIGDIRQAYC	35
JN681227	C	CCR5	CTRPGNNTRKSIRIGPGQAFYATNDIIGDIRQAHC	35
GQ999974	C	CCR5	CRRPNNNTRKSIRIGPGQAFYATNDIIGDIRQAHC	35
GQ999976	C	CCR5	CARPHNNTRKSIRIGPGQAFFATNDIIGDIRQAHC	35
GQ999978	C	CCR5	CTRPNNNTRKSIRIGPGQTFFATNEIIGNIRQAHC	35
GQ999979	C	CCR5	CTRPGNNTRKSIRIGPGQTFYATGDIIGDIRQAHC	35
GQ999980	C	CCR5	CIRPSNNTRKSIRIGPGQAFYATNAITGDLRQAHC	35
FJ443982	C	CCR5	CIRPGNNTRKSVRIGPGQTFYATGDIIGDIRKAYC	35
HQ595744	C	CCR5	CTRPNNNTRRSIRIGPGQTFYTNDIIGDIRQAHC	34
HQ595745	C	CCR5	CTRPNNNTRKSVRIGPGQTFYATGDIIGNIREAHC	35
HQ615959	C	CCR5	CIRPGNNTRKSIRIGPGQTFYATGDIIGDIRKAHC	35
KC894111	C	CCR5	CTRPGNNTRKSVRIGPGQTFYATDIIGNIRQAHC	34
KC863324	C	CCR5	CTRPGNNTRRSVRIGPGQTFYAMGDIIGDIRQAHC	35
KC863361	C	CCR5	CTRPDNNTRKSIRIGPGQTFYANSIIGDIRQAHC	34
KC894133	C	CCR5	CIRPNNNTRKSIRIGPGQTFYAINGIIGDIRQAYC	35
FJ443417	C	CCR5	CTRPNNNTRKSVRIGPGQTFYATGEIIGQIRQAHC	35
FJ443426	C	CCR5	CIRPNNNTRKSIRIGPGQTFYANNDIIGDIRQAHC	35
FJ443473	C	CCR5	CTRPNNNTRTSIRIGPGQAFYATNGIIGDIRQAHC	35
FJ443488	C	CCR5	CVRPGNNTRKSIRIGPGQTFYANNIIGDIRQAHC	34
FJ443498	C	CCR5	CTRPGNNTRKSIRIGPGQAFYATNSIIGDIRKAHC	35
FJ443515	C	CCR5	CTRPGNNTRKSIRIGPGQTFYATGDIIGDIRQAHC	35
HQ615950	C	CCR5	CTRPGNNTRKSVRIGPGQTFYATGDIIGDIRQAHC	35
HQ615944	C	CCR5	CTRPGNNTRQSVRIGPGQTFYATGAITGDIRQAHC	35
HQ595746	C	CCR5	CTRPNNNTRKSIRIGPGQTFYATGEIIGNIRDAHC	35
HQ595766	C	CCR5	CTRPGNNTRKSIRIGPGQTFYAPGDIIGDIRKAHC	35
KC894386	C	CCR5	CMRPGNNTRKSIRIGPGQTFYATGAIIGDIRQAHC	35
HQ595759	C	CCR5	CTRPGNNTRKSIRIGPGQTFYATGAIIGDIRQAHC	35
HQ615942	C	CCR5	CTRPNNNTRRSIRIGPGQTFYANTIIGDIRQAYC	34
KT183087	C	CCR5	CTRPNNNTRKSVRIGPGQTFYATGEIIGNIRQAYC	35
KT183090	C	CCR5	CVRPNNNTRKSIRIGPGQAFYATNDIIGNIREAHC	35
KT183211	C	CCR5	CIRPNNNTRKSIRIGPGQTFYANNEIIGDIRQAHC	35
KT183216	C	CCR5	CTRPGNNTRQSIRIGPGQTFYATGDIIGNIRQAHC	35
KT183229	C	CCR5	CTRPGNNTRKSVRIGPGQTFYATGDIIGDIRNAHC	35
KT183253	C	CCR5	CTRPNNNTRKSIRIGPGQAFFATGEVIGDIRKANC	35
KT183271	C	CCR5	CTRPNNNTRKSVRIGPGQTFYATGDIIGDIRQAHC	35
KT183279	C	CCR5	CIRPNNNTRKSIRIGPGQAFYATGEIIGDIRKAYC	35
HQ595761	C	CCR5	CVRPGNNTRKSIRIGPGQAFYAHNDIIGDIRQAYC	35
KC894116	C	CCR5	CMRPGNNTRRSVRIGPGQTFYATGDIIGDIRQAHC	35
KC894119	C	CCR5	CTRPNNNTRRSITIGPGQAFYAQTNIIGDIRKAHC	35
KC156130	C	CCR5	CTRPGNNTRKSIRIGPGQTFFATNDIIGDIRQAYC	35
KC894115	C	CCR5	CTRPSNNTRKSIRIGPGQAFYANDIIGDIRQAHC	34
JN687704	C	CCR5	CTRPNNNTRKSVRIGPGQTFYATGGIIGDIKQAHC	35
JN687706	C	CCR5	CTRPGNNTRKSIRIGPGQTFYATGEIIGDIRQAHC	35
JN687718	C	CCR5	CTRPNNNTRKSVRIGPGQAFYATNGIVGDIRQAHC	35
JN687735	C	CCR5	CTRPGNNTRKSIRIGPGQVFYANDIIGDIREAHC	34

JN687736	C	CCR5	CTRPNNNTRKSVRIGPGQTFYATGAIIGDIRQAHC	35
JX976681	C	CCR5	CTRPSNNTRKSIRIGPGQAFYATGEVTGDIRQAHC	35
KC863587	C	CCR5	CTRPNNNTRKSVRIGPGQTFYYANDIIGDIRKAYC	35
KC863599	C	CCR5	CTRPNNNTRKSMRIGPGQTFYATGGIIGDIRQAHC	35
HM204585	C	CCR5	CVRPNNNTRSSIRIGPGQAFYAMNDIIGDIRQAYC	35
HM204594	C	CCR5	CTRPGNNTRKSMRIGPGQTFYATGEVIGDIREAHC	35
HM204595	C	CCR5	CIRPNNNTRKSIRIGPGQTFYANNAIIGDIREAHC	35
HM204600	C	CCR5	CTRPNNNTRKSISMGPGQAFYATGDIIGDIRQAHC	35
HM204602	C	CCR5	CTRPNNNTRKSIRIGPGQAFYATGGIIGDIRQAHC	35
HM204604	C	CCR5	CIRPNNNTRKSVRIGPGQTFYATNDIIGNIREAHC	35
HM204606	C	CCR5	CTRPNNNTRKSVRIGPGQAFYATGEIIGKIREAHC	35
HM204607	C	CCR5	CIRPNNNTRKSMRIGPGQTFYAYGEIVGDIRQAHC	35
HM204608	C	CCR5	CTRPNNNTRQSVRIGPGQAFYATGDIIGDIRQAHC	35
HM204609	C	CCR5	CTRPNNNTRKSVRIGPGQTFYATGDIIGDIRQAHC	35
HM204611	C	CCR5	CVRPNNNTRRSMRIGPGQTFYATGDIIGNIRQAHC	35
HM204616	C	CCR5	CTRPNNNTRRSMRIGPGQTFYATGDIIGDIRQAYC	35
FJ443732	C	CCR5	CTRPNNNTRKSIRIGPGQTFYATGEIIGDIRQAHC	35
FJ443905	C	CCR5	CTRPGNNTRKSMRIGPGQTFYATGNIIGDIRQAHC	35
FJ443988	C	CCR5	CTRPNNNTRKSIRIGPGQTFYANNIIGDIRQAYC	34
MN202471	C	CCR5	CTRPNNNTRKGIRIGPGQTFYATNDIIGDIRAAHC	35
HQ595747	C	CCR5	CTRPNNNTRQSIRIGPGQTFYATGDIIGDIRQAHC	35
HQ615943	C	CCR5	CTRPNNNTRKSIRIGPGQTFYATDIIGDIRQAHC	34
HQ595749	C	CCR5	CTRPGNNTRKSVRIGPGQAFYTTGEIIGDIRQAHC	35
HQ595750	C	CCR5	CMRPGNNTRTSVRIGPGQTFFANNNIIGNIRNAHC	35
HQ615946	C	CCR5	CARPGNNTRTSIRIGPGQAFYTTGDIIGDIRAAHC	35
KY713231	C	CCR5	CTRPNNNTRKSIRIGPGQTFYATGDIIGDIRQAHC	35
KT183053	C	CCR5	CTRPNNNTRKSVRIGPGQTFYATGAIIGDIRQAYC	35
KT183056	C	CCR5	CTRPNNNTRESVRIGPGQVFYATKGIIGDIRQAYC	35
KT183125	C	CCR5	CIRPNNNTRKSIRIGPGQTFFATDIIGDIRQAYC	34
KT183128	C	CCR5	CTRPNNNTRRSIRIGPGQTFYATDIIGDIRQAYC	34
KT183153	C	CCR5	CTRPNNNTRKSIRIGPGQTFYATNIIGDIRQAHR	34
KT183172	C	CCR5	CVRPSNNTRKSIRIGPGQTFYATGDIIGDIKQAHC	35
KT183183	C	CCR5	CTRPNNNTRKSIRIGPGQTFYANNIIGDIRQAHC	34
KT183201	C	CCR5	CTRPGNNTRKSIRIGPGQTFYATNGIIGDIRQAHC	35
KT183243	C	CCR5	CTRPGNNTRRSIRIGPGQAFYTTGEIIGDIRQAHC	35
KT183245	C	CCR5	CTRPNNNTRKSIRIGPGQTFYATDIIGNIREAYC	34
KT183250	C	CCR5	CTRPGNNTRKSIRIGPGQAFFATNDIIGDIRQAHC	35
KT183258	C	CCR5	CARPNNNTRKSMRIGPGQTFYATGAIIGNIRQAHC	35
KT183274	C	CCR5	CTRPGNNTRKSIRIGPGQTFYATGEIIGDIRQAHC	35
HQ615983	C	CCR5	CTRPNNNTRKSIRIGPGQTFFATNEIIGDIRQAHC	35
HQ615966	C	CCR5	CTRPDNNTRKSIRIGPGQAFYAMGDIIGDIRQAHC	35
KC156221	C	CCR5	CTRVGNNTRKSVRIGPGQTFYATGDIIGDIREAHC	35
HQ615967	C	CCR5	CTRPGNNTRESIRIGPGQTFYATGDIIGNIRQAHC	35
HM623551	C	CCR5	CTRPSNNTRKSIRIGPGQAFYATNDIIGDIRQAHC	35
HM623553	C	CCR5	CTRPNNNTRKSVRIGPGQAFYATNDIIGNIREAHC	35
HM623557	C	CCR5	CVRPNNNTRKSIRIGPGQAFYATNEIIGDIRQAYC	35
HM623563	C	CCR5	CTRPNNNTRKSIRIGPGQAFYATDIIGNIRQAYC	34
HM623564	C	CCR5	CARPGNNTRKSVRIGPGQTFYATGDIIGDIRQAHC	35
HM623569	C	CCR5	CVRPNNNTRTSIRIGPGQVFYATGDIIGDIRQAHC	35
HM623570	C	CCR5	CTRPNNNTRQSIRIGPGQTFYGNDVIGDIRQAHC	34
HM623572	C	CCR5	CVRPNNNTRKSVRIGPGQSLFTTDIIGDIRLAYC	34
HM623574	C	CCR5	CIRPGNNTRKSIRIGPGQTFFANDIIGDIRQAHC	34
HM623579	C	CCR5	CIRPGNNIRKSVRIGPGQTFYATGDIIGDIRKAYC	35
HM623581	C	CCR5	CTRPGNNTRTSIRIGPGQAFFATGAIIGDIRQAHC	35
HM623582	C	CCR5	CTRPNNNTRTSIRIGPGQMFYATGDIIGDIRQAHC	35
JN687728	C	CCR5	CTRPNNNTRKSVRIGPGQAFYATGAIVGEIRQAHC	35
JN687730	C	CCR5	CIRPGNNTRQSIRIGPGQTFYATGEVIGNIRQAHC	35
JN687731	C	CCR5	CTRPNNNTRKSVRIGPGQTFYATGDIIGNIRQAHC	35
KF114884	C	CCR5	CTRPNNNTRKGIRIGPGQVFYTTDIIGDIRQAHC	34
KC154018	C	CCR5	CIRPNNNTRKSVRIGPGQTFYATGGIIGDIRQAYC	35
KC154021	C	CCR5	CIRPGNNTRKSVRIGPGQTFYATGAIIGDIRKAHC	35
KC154022	C	CCR5	CTRPNNNTRKSIRIGPGQAFYANSNIIGDIRKAHC	35
KC154024	C	CCR5	CSRPGNNTRKSIRIGPGQAFYATGDIIGDIRQAHC	35
KF114885	C	CCR5	CTRPDNNTRKSIRIGPGQTFYATNIIGNIRQAHC	34
HM204624	C	CCR5	CTRPGNNTRRSVRIGPGQTFYATGQIIGNIREAHC	35
JN681254	C	CCR5	CTRPNNNTRKSVRIGPGQTFYATGDIIGNIRQAHC	35
HQ615960	C	CCR5	CVRPNNNIRTSVRIGPGQAFFATNDIIGDIRQAYC	35
HQ595751	C	CCR5	CIRPNNNTRRSVRIGPGQSYFTTGEIIGDIRNAFC	35
JQ754244	C	CCR5	CTRPNNNTRKSVRIGPGQTFYATGEIIGNIRQAHC	35
HQ595765	C	CCR5	CVRLNNNTRKSIRIGPGQAFYAHGDIIGDPRQAHC	35

HQ615956	C	CCR5	CTRPNNNTRKSIRIGPGQSFYATGDIIGDIRQASC	35
KY658710	C	CCR5	CTRPNNNTRKSIRIGPGQTFYATGDIIGDIRQAHC	35
KT183068	C	CCR5	CTRPNNNTRKSIRIGPGQTFYAYGEITGNIREAHC	35
KT183114	C	CCR5	CTRPSNNTRRSVRIGPGQAFYTTGDITGDIRQAHC	35
KT183141	C	CCR5	CTRPGNNTRKSVRIGPGQVFFATNDIIGNIRQAHC	35
GU080161	C	CCR5	CIRPGNNTRKSVRIGPGQTFYANDIIGDIRKAHC	34
GU080162	C	CCR5	CTRPNNNTRQSIGFGPGQAFYATGDIIGDIRQAHC	35
GU080164	C	CCR5	CVRPNNNTRTSIRIGPGQTFYATNGIIGDIREAHC	35
GU080165	C	CCR5	CTRPGNNTRRSVRIGPGQTFYATGDIIGDIRQAHC	35
GU080166	C	CCR5	CTRPGNNTRKSVRIGPGQTFYATGDIIGNTRQAHC	35
GU080173	C	CCR5	CTRPNNNTRKSVRIGPGQAFYATGGIIGDIRQAYC	35
GU080178	C	CCR5	CLRPGNNTRKSIRIGPGQTFYATGAIIGDIRQAHC	35
GU080179	C	CCR5	CTRPGNNTRKSVRIGPGQTFYATGDIIGNIRQAHC	35
GU080180	C	CCR5	CTRPNNNITTRIISIGPGRPFYTKNIGRDIKQAYC	35
GU080181	C	CCR5	CTRPGNNIRKSIRIGPGQAFFTTGDIIGDIRQAYC	35
GU080185	C	CCR5	CTRPGNNTRRSIRIGPGQTFYATGDIIGDIRKAHC	35
GU080191	C	CCR5	CTRPGNNTRTSIRIGPGQTFYATGAVTGDIRQAHC	35
GU080192	C	CCR5	CTRPGNNTRKSIRIGPGQTFYATGDIIGNIRQAHC	35
GU080197	C	CCR5	CTRPNNNTRKSIRIGPGQTFYATGDIIGDIRQAHC	35
GU080193	C	CCR5	CTRPGNNTRQSIRIGPGQTFYATGDIIGDIRKAHC	35
HM623592	C	CCR5	CTRPGNNTRKSIRIGPGQTFYATGDVIGDIRKAFC	35
HM623593	C	CCR5	CVRPNNNTRKSVRIGPGQMFYATGDIIGNIRQAHC	35
HM623595	C	CCR5	CIRTGNNTRKSVRIGPGQTFYAANDIIGDIRKAYC	35
HM623597	C	CCR5	CTRPNNNTRKSIRIGPGQTFYATNNIIGNIREAHC	35
HM623599	C	CCR5	CIRPNNNTRKSIRIGPGQTFYATGEIIGNIRQAHC	35
HM623600	C	CCR5	CTRPNNNTRKSIRIGPGQAFYATNDIIGDIRQAYC	35
HM623602	C	CCR5	CTRPNNNTRTSVRIGPGQTFYATGEIIGNIRQAHC	35
HM623605	C	CCR5	CTRPNNNTRKGIRIGPGQTFYATGEIIGDIRKAYC	35
HM623607	C	CCR5	CTRPGNNTRKSIRIGPGQAFYATGDIIGDIRQAHC	35
HM623608	C	CCR5	CTRPGNNTRKSIRIGPGQAFYATGAITGDIRQAHC	35
HM623609	C	CCR5	CTRPGNNTRKSVRIGPGQTFYATGEIIGDIRQAHC	35
HM623610	C	CCR5	CTRPNNNTRKSIRIGPGQTFYAAGDIIGDIRQAHC	35
HM623583	C	CCR5	CVRPNNNTRQSIRMGPGQAFYATGEIIGDIRQAYC	35
HM623586	C	CCR5	CIRPGNNTRKSIRIGPGQTFFATNIIGDIRQAYC	34
KC154026	C	CCR5	CTRPNNNTRRSIRIGPGQTFYANAIIGDIRQAHC	34
KC154027	C	CCR5	CTRPNNNTRKSIRIGPGQTFYATNIIGDIRQAHC	34
MF284922	C	CCR5	CTRPNNNTRKSVRIGPGQTIYATGDIIGDIRQAHC	35
MF284952	C	CCR5	CTRPNNNTRKSVRIGPGQAFYATNDIIGDIRQAHC	35
MF284958	C	CCR5	CARPGNNTRTSVRIGPGQTFYATGDIIGDIRKAHC	35
MF284962	C	CCR5	CTRPGNNTRKSVRIGPGQVFYATNDIIGDIRQAHC	35
MF284977	C	CCR5	CTRPGNNTRQSIRIGPGQTFYAWGDIIGDIRQAHC	35
MF285022	C	CCR5	CTRPGNNTRKSIRIGPGQTFYATGDIIGDIRQAHC	35
MF285058	C	CCR5	CTRPGNNTRKSIRIGPGQVFYATNDVIGDIRQAHC	35
JX140667	C	CCR5	CTRPHNNTRKSVRIGPGQTFYATGEIIGNIRQAHC	35
JX140668	C	CCR5	CIRPNNNTRKSIRIGPGQTFYANNIIGDIRQAYC	34
KF114892	C	CCR5	CTRPNNNTRKSMRIGPGQTFYATGDIIGDIRQAHC	35
KF725883	C	CCR5	CTRPNNNTRKSIRIGPGQVFYATDIIGDIRQAYC	34
KF725889	C	CCR5	CTRPGNNTRKSIRIGPGQAFYAHGDVIGNIRLAHC	35
KF725890	C	CCR5	CTRPGNNTRKSVRIGPGQVFYATNDIIGDIRQAYC	35
KF725891	C	CCR5	CTRPNNNTRKSVRIGPGQTFYATGDIIGDIRQAYC	35
KF725892	C	CCR5	CVRPNNNTRKSIRIGPGQTFYATGAIIGDIRQAHC	35
KF725895	C	CCR5	CTRPNNNTRKSIRIGPGQTFYAHNNIIGDIRQAHC	35
KF725896	C	CCR5	CTRPQNNTRKSIRIGPGQAFYATNAIIGDIRQAHC	35
KF725908	C	CCR5	CIRPGNNTRKSVRIGPGQTFYATGEIIGNIRQAHC	35
KF725912	C	CCR5	CTRPGNNTRKSIRIGPGQAFYATGAITGDIRKAHC	35
KF725914	C	CCR5	CIRPGNNTRRSVRIGPGQAFYATNDIIGDIRQAHC	35
KF725916	C	CCR5	CTRPNNNTRKSVRIGPGQTFFATGDIIGDIRQAYC	35
KF725922	C	CCR5	CTRPNNNTRKSVRIGPGQIFYATGDIIGNIREAHC	35
JN681258	C	CCR5	CTRTGNNTRTSARIGPGQTFFATGGIIGDIRKAHC	35
KF725928	C	CCR5	CTRPNNNTRKSVRIGPGQTFYATGSIIGNIRQAHC	35
KF725929	C	CCR5	CTRTGNNTRKSIRIGPGQAFYATDAIIGDIRQAYC	35
KF725933	C	CCR5	CIRPGNNTRKSVGIGPGQAFYAIGDIIGDIRQAHC	35
KF725935	C	CCR5	CTRPNNNTRKSTRIGPGQTFYTMGQVIGDIRQAYC	35
KF725936	C	CCR5	CIRPGNNTRRSVRIGPGQTFFATGDIIGDIRQAHC	35
KF725938	C	CCR5	CVRPNNNTRKSIRIGPGQAFYATGQIIGDIRAAHC	35
KF725944	C	CCR5	CTRPNNNTRKSIRIGPGQAFYANNDIIGDIRQAYC	35
KF725948	C	CCR5	CARPNNNTRRSVRIGPGQVFYATGEIIGDIRQAHC	35
KF725954	C	CCR5	CSRPNNNTRKGIRIGPGQAFYATNGIIGDIRQAHC	35
KF725957	C	CCR5	CTRPGNNTRKSIRIGPGQAFYATGDIIGDIRQAHC	35
KF725968	C	CCR5	CIRPGNNTRKSIRIGPGQTFYATGDIIGDIRKAHC	35

KF725970	C	CCR5	CTRPNNNTRKSVRIGPGQAFYATNGIIGDIRQAHC	35
KF725972	C	CCR5	CTRPGNNTRKSVRIGPGQTFYATGDIIGDIRQAHC	35
KF725975	C	CCR5	CIRPNNNTRKSIRIGPGQTFYATGDIIGNIRQAYC	35
KF725976	C	CCR5	CIRPNNNTRKSIRIGPGQTFYATGEIIGDIRQAHC	35
KF725983	C	CCR5	CERPNNNTRKSIRIGPGQTFFANEIIGDIRQAHC	34
KF725987	C	CCR5	CTRPGNNTRKSVRIGPGQTFYATGDIIGDIRQAHC	35
KF725990	C	CCR5	CTRPNNNTRKSVRIGPGQTFYATGAIIGDIREAHC	35
KF725995	C	CCR5	CTRPNNNTRKSVRIGPGQAFYANNDIIGDIRQAHC	35
KF725996	C	CCR5	CTRPGNNTRKSIRIGPGQAFYANNDIIGDIRQAHC	35
KF725997	C	CCR5	CTRPNNNTRTSHRIGPGQTFYATGDIIGDIRQAHC	35
KF725999	C	CCR5	CTRPNNNIRKSIRIGPGQAFYATGEIIGDIRQAYC	35
KF726000	C	CCR5	CTRPNNNTRKSIRIGPGQTFYATGDIIGDIRQAHC	35
KF726001	C	CCR5	CTRPSNNTRKSVRIGPGQVFYANSDIIGDIRKAHC	35
KF726011	C	CCR5	CTRPNNNTRTSVRIGPGQAFYATNGIIGDIRQAHC	35
KF726014	C	CCR5	CTRPNNNTRKSIRIGPGQTFYATGDVIGDIRQAHC	35
KF726020	C	CCR5	CTRPNNNTRKSIRIGPGQTFYATGDIIGNIRKAHC	35
KF726022	C	CCR5	CTRPNNNTRKSIRIGPGQAFYATGEIIGDIRQAHC	35
KF726025	C	CCR5	CTRPNNNTRKSIRIGPGQTFYATNIIGDIRQAHC	34
KU749416	C	CCR5	CTRPNNNTRESVRIGPGQTFYATGDIIGDIRQAHC	35
MH933705	C	CCR5	CTRPNNNTRQSIRIGPGQTFYATGDIIGNIRQAHC	35
MK643564	C	CCR5	CTRPGNNTRRSIRIGPGQALFTNNIIGDIRQAHC	34
MK643538	C	CCR5	CIRPGNNTRKGIGIGPGQTFYASEGIIGDIRQAHC	35
MK643537	C	CCR5	CTRPSNNTRKSVRIGPGQTFYATGDIIGDIRQAHC	35
MN097551	C	CCR5	CTRPSNNTRKSIRIGPGQTFYATGAIIGNIREAHC	35
MN097661	C	CCR5	CTRPGNNTRESIRIGPGQTFYATGQIIGDTRQAHC	35
JN188292	C	CCR5	CTRPGNNTRKSVRIGPGQTFFATGEIIGKIREAHC	35
AY118165	C	CCR5	CTRPNNNTRKSIRIGPGQAFYATGDIIGDIRQAYC	35
AY118166	C	CCR5	CTGPNNNTRKSMRIGPGQVFYATNGIIGDIRQAHC	35
AF286227	C	CCR5	CTRPNNNTRKSMRIGPGQTFYATGDIIGDIRQAYC	35
AY158533	C	CCR5	CTRPNNNTRKSMRIGPGQTFFATGDIIGNIRQAHC	35
AY158534	C	CCR5	CTRPHNNTRKSVRIGPGQAFYATGDIIGNIRQAHC	35
AY158535	C	CCR5	CIRPGNNTRQSIRIGPGQTFFATGDIIGDIRQALC	35
AY529659	C	CCR5	CTRPNNNTRKGVRIGPGQTFYATGDIIGDIRQAHC	35
AY043176	C	CCR5	CTRPGNNTRKSIRIGPGQAFYATGDIIGDIRQAHC	35
DQ411850	C	CCR5	CTRPNNNTRKSIRIGPGQTVYATNDIIGDIRQAHC	35
AY529661	C	CCR5	CTRPSNNTRKSVRIGPGQTFYATNDIIGDIRQAHC	35
AY529662	C	CCR5	CARPNNNTRKSIRIGPGQAFYATGAIIGNIREAHC	35
AY162224	C	CCR5	CTRPGNNTRKSVRIGPGQAFYATGDIIGDIRQAHC	35
DQ056405	C	CCR5	CTRPNNNTRKSIRIGPGQTFYATGEIIGDIRQAHC	35
AF391238	C	CCR5	CPRPNHNTRRSIRIGPGQAFYATGDIIGDIRQAHC	35
AF391242	C	CCR5	CTRPNNNTRKSVRIGPGQTFYATGDIIGDIREAHC	35
JX845593	C	CCR5	CTRPNNNTRRSIRIGPGQAFYTNDIIGDIRQAHC	34
AF391247	C	CCR5	CTRPGNNTRKSVRIGPGQTFYATGDIIGDIRQAHC	35
AF391250	C	CCR5	CTRPGNNTRKSIRIGPGQAFFATGAIIGDIRKAYC	35
AY529664	C	CCR5	CTRPSNNTRKSIRIGPGQAFFATNAIIGDIRQAHC	35
AY529665	C	CCR5	CVRPNNNTRKSIRFGPGQAFYATGDILGDIRQAHC	35
EU293450	C	CCR5	CTRPSNNTRKSVRIGPGQAFFATGEIIGDIRQAHC	35
AY529675	C	CCR5	CTRPNNNTRKSVRIGPGQTFYAEGEIIGNIRQAHC	35
AY228556	C	CCR5	CTRPNNNTRKSIRIGPGQTFYATNIIGDIRQAYC	34
AY529676	C	CCR5	CTRPNNNTRQSVRIGPGQVFYATNDIIGDIRQAYC	35
DQ447266	C	CCR5	CTRPNNNTRRSIRIGPGQTFYATGDIIGDIRQAHC	35
AY043174	C	CCR5	CTRPGNNTRKSIRIGPGQAFYTNHIIGDIRQAYC	34
AY043175	C	CCR5	CTRPNNNTRKSVRIGPGQTFYATGEIIGDIREAHC	35
DQ411852	C	CCR5	CTRPNNNTRKSVRIGPGQTFYATGDIIGDIRQAHC	35
AY522722	C	CCR5	CIRPNNNTRKSIRIGPGQAFFANNIIGNIRQAHC	34
AY522723	C	CCR5	CTRPSNNTRQGIGIGPGQAFFATTNIIGDIRQAHC	35
AY522724	C	CCR5	CMRPSNNTRKSMRIGPGQTFFATGDIIGDIRKAHC	35
AY522725	C	CCR5	CTRPNNNTRKSIRIGPGQVFYAHNNIIGNIRDAHC	35
AY522727	C	CCR5	CTRPGNNTRTSIRIGPGQSFYATGEIIGDIRQAHC	35
AY522729	C	CCR5	CIRTGNNTRKSVRIGPGQSFFATGEIIGDIRQAHC	35
AY522730	C	CCR5	CVRPGNNTRKSVRIGPGQTFYATGDIIGDIRKAHC	35
AY522736	C	CCR5	CTRPGNNTRKSIRIGPGQAFFATGDIIGDIRQAHC	35
KU168308	C	CCR5	CTRPNNNTRKSMRIGPGQTFYATGDIIGDIRQAYC	35
GU216805	C	CCR5	CIRPNNNTRKSIRIGPGQTFYATNEIIGDIRQAYC	35
HQ625593	C	CCR5	CTRPPNNTIKSMRIGPGQTFYATGQIIGDIRQAYC	35
HQ625570	C	CCR5	CTRPNNNTRTSIRIGPGQTFYATDIIGNIRQAHC	34
HQ625595	C	CCR5	CTRPNNNTRRSIRIGPGQAFYATGDIIGNIRQAYC	35
HQ625590	C	CCR5	XNTRKSIRIGPGQSFYAVGQAYC	23
HQ625589	C	CCR5	CTRPGNNTRKSVRIGPGQTFYATNEIIGDIRQAHC	35
HQ625572	C	CCR5	CIRPNNNTRKSIRIGPGQAFHATGEIIGDIRQAYC	35

HQ625566	C	CCR5	CTRPSNNTRKSIRIGPGQAFYATNAIIGDIREAHC	35
HQ625573	C	CCR5	CVRPANNTRKSVGIGPGQTIYATGEIIGDIRQAHC	35
HQ625587	C	CCR5	CIRPNNNTRKSIRIGPGQAFYATGDIIGNIRQAHC	35
HQ625574	C	CCR5	CTRPGNNTRKSIRIGPGQAFYATGDIIGDIRQAHC	35
HQ625575	C	CCR5	CMRPGNNTIKSVRIGPGQAFYATGDIIGDIRQAHC	35
HQ625585	C	CCR5	CTRPGNNTRKSIRIGPGQTFYATGDIIGDIRQAHC	35
HQ625576	C	CCR5	CTRPGNNTRKSIRIGPGQAFYATGEIIGDIRQAYC	35
HQ625584	C	CCR5	CTRPNNNTRKSIRIGPGQAFYATNDIIGDIRQAHC	35
HQ625565	C	CCR5	CTRPGNNTRTSVRIGPGQTFYATGDIIGDIRQAHC	35
HQ625583	C	CCR5	CTRPNNNTLKSVRIGPGQAFFTTGSIIGDIRKAYC	35
HQ625578	C	CCR5	CTRPNNNTRKSVRIGPGQAFYATGEIIGNIRQAHC	35
HQ625594	C	CCR5	CVRPNNNTRRSVRIGPGQTFYATGDIIGDIRQAYC	35
HQ625579	C	CCR5	CARPNNNTRNSVRIGPGQVFYTTDIIGDIRQAHC	34
HQ625567	C	CCR5	CTRPSNNTRTSVRIGPGQMFYATGNIIGNIRQAHC	35
KC894134	C	CCR5	CTRPNNNTRKSIRIGPGQTFYATGEIIGNIRKAYC	35
HQ625592	C	CCR5	CTRPGNNTRKSMRIGPGQTFYATGEIIGNIRQAHC	35
HQ625571	C	CCR5	CVRPGNNTRQSIGIGPGRAFYARGDIIGDIRQAHC	35
GU939100	C	CCR5	CTRPGNNTRKGIGIGPGQTFYTNDVIGDIRQAHC	34
HM036825	C	CCR5	CVRPGNNTRQSVRIGPGQTFYATGDIIGDPRAAHC	35
KY229391	C	CCR5	CIRPSNNTRRSIRIGPGQAFYATGDITGDIRKAHC	35
AB254142	C	CCR5	CTRPNNNTRESIRIGPGQTFFATGDIIGNIRQAHC	35
AB254149	C	CCR5	CERPNNNTRTSVRIGPGQTFYATGDIIGNIRQAYC	35
AB254153	C	CCR5	CTRPNNNTRRSIRIGPGQVFYATTDIIGDIRQAHC	35
KY229251	C	CCR5	CIRPSNNTRKSIGIGPGRAFYATGDITGDIRQAYC	35
HM036760	C	CCR5	CTRPNNNTRKSVRIGPGQTFYATGGIIGNIRQAHC	35
KY229414	C	CCR5	CTRPGNNTRKSVRIGPGQTFYATGDIIGDIRQAHC	35
KY229678	C	CCR5	CTRPNNNTRKSVRIGPGQTFYAPGDIIGDIRQAYC	35
KY229337	C	CCR5	CTRPGNNTRQSIRIGPGQAFYATGDIIGDIRQAHC	35
KY229337	C	CCR5	CTRPGNNTRQSIRIGPGQAFYATGDIIGDIRQAHC	35
KY229356	C	CCR5	CVRPNNNTRRSVRIGPGQAFYATGDIVGDIRQAYC	35
KY229374	C	CCR5	CIRPGNNTRRSVRIGPGQAFYTTGEIIGNIRQAHC	35
KY229434	C	CCR5	CTRPNNNTRKSVRIGPGQTFYATGEIIGNIREAYC	35
KY658710	C	CCR5	CTRPNNNTRKSIRIGPGQTFYATGDIIGDIRQAHC	35
HM037006	C	CCR5	CTRPGNNIRKSVRIGPGQTFYATGDITGDIRQAHC	35
EU166385	C	CCR5	CTRPNNNIRKSIRIGPGQSFHATGSIIGDIREAYC	35
DQ388515	C	CCR5	CVRPNNNTRKSVRIGPGQTFFATGEIIGDIRQAHC	35
EU166449	C	CCR5	CTRPNNNTRKSVRIGPGQTFYATGEIIGNIRQAHC	35
EU166594	C	CCR5	CARPGNNTRKSMRIGPGQTFYATGAIIGDIRQAHC	35
DQ388517	C	CCR5	CTRPNNNTRKSIRIGPGQSFYATGEIVGNIREAHC	35
HM036903	C	CCR5	CTRPGKNIRKSVRIGPGQAFFATGDIIGDIRQAYC	35
KY229542	C	CCR5	CTRPNNNTRKSIRIGPGQTFFATNDIIGDIRQAHC	35
GQ485357	C	CCR5	CVRPNNNTRKSVRIGPGQTFYATGEIIGDIRQAYC	35
HM068598	C	CCR5	CTRPNNNTRKSIRIGPGQTFYATDGIIGNIRQAHC	35
EU166715	C	CCR5	CTRPNNNTRKSIRIGPGQAFYATGDIIGNIKQAYC	35
FJ496194	C	CCR5	CTRPNNNIRQSIRIGPGQTFFATGDIIGDIREAHC	35
EU166769	C	CCR5	CTRPNNNTRRSIRIGPGQTFYATGDIIGDIRQAHC	35
JX239338	C	CCR5	CIRPNNNTRKSIRIGPGQVFYAAQDIIGDIRQAHC	35
FJ496214	C	CCR5	CTRPNNNTRKSIRIGPGQTFYATGEIIGKIREAHC	35
KY229633	C	CCR5	CTRPNNNTRKSVRIGPGQTFYATGEIIGDIKQAHC	35
KY229641	C	CCR5	CTRPNNNTRKSMRIGPGQTFYATGDIIGNIRHAYC	35
HM036792	C	CCR5	CTRPNNNTRRSIRIGPGQVFFATNEIIGDIREAHC	35
KY229650	C	CCR5	CTRPNNNTRQSVRIGPGQTFYATGTIVGNIRAAYC	35
GQ485436	C	CCR5	CTRPNNNTSRSVRIGPGQTFFATGRIIGDIRQAYC	35
JX239272	C	CCR5	CTRPSNNTRRSVRIGPGQTFFATGEIIGDIRQAHC	35
JX239362	C	CCR5	CTRPGNNTRRSIRIGPGQTFYATGAIIGDIRQAHC	35
GU329057	C	CCR5	CTRPGNNTRKSVRIGPGQVFYATGDIIGDIRQAHC	35
GU329069	C	CCR5	CTRPNNNTRQSIRIGPGQTFYATGGIIGDIRQAHC	35
GU329080	C	CCR5	CTRPGNNTRKSVRIGPGQAFFATGAIIGDIRQAHC	35
GU329131	C	CCR5	CMRPGNNTRKSVRIGPGQTFYATGDIIGDIRQAHC	35
GU329145	C	CCR5	CTRPNNNTRRSIRIGPGQAFYATGGIIGDIRQAHC	35
GU329161	C	CCR5	CTRPGNNTRKSIRIGPGQTFYATGAIIGDIRQAHC	35
GU329185	C	CCR5	CTRPGNNTRKSTRIGPGQTFYATGEIIGDIRKAHC	35
GU329269	C	CCR5	CTRPGNNTRRSMRIGPGQAFYATGDIIGDIRKAHC	35
GU329303	C	CCR5	CTRPNNNTRKSIRIGPGAAFYAHNNIIGDIRQAYC	35
GU329320	C	CCR5	CTRPGNNTRKSIRIGPGQAFYATGDIIGDIRQAHC	35
GU329359	C	CCR5	CTRPGNNTRKSIRIGPGQTFYAHGDIIGDIRQAHC	35
GU329387	C	CCR5	CTRVGNNTRKGIGIGPGQTFYATDAIIGDIRKAHC	35
GU329411	C	CCR5	CTRPNNNTRKSVRIGPGQTFFATGEIIGDIRQAHC	35
GU329428	C	CCR5	CIRPNNNTRKSMRIGPGQTFFATGDIIGDIRQAHC	35
GU329473	C	CCR5	CIRPNNNTRKSIRIGPGQTFYATGEVIGDIRQAYC	35

GU329489	C	CCR5	CTRPNNNTRQGIRIGPGQTFYARGEVIGDIRQAYC	35
GU329494	C	CCR5	CTRPNNNTRKSVRIGPGQTFYATGEIIGDIRQAYC	35
GU329501	C	CCR5	CIRPGNNTRKSVRIGPGQAFFATGDIIGDIRRAHC	35
KX983797	C	CCR5	CTRPGNNTRRSIRIGPGQAFYATGDIIGDIRQAHC	35
JX213352	C	CCR5	CTRPGNNTRKSIRIGPGQTFYATNAIIGDIRQAYC	35
JX239395	C	CCR5	CTRPGNNTRKSIRIGPGQVFYATGDIIGDIRKAHC	35
JN977604	C	CCR5	CTRPNNNTRKSIRIGPGQEFFATGEIIGNIRQAHC	35
KT252545	C	CCR5	CARPGNNTRQSTRIGPGQTFFITGEIIGDIRQAHC	35
KX983777	C	CCR5	CTRPGNNTRRSVRIGPGQSFYATGDIIGDIRQAYC	35
KX983831	C	CCR5	CVRPNNNTRKSIRIGPGQTFFATGDIIGNIRQAYC	35
JX213464	C	CCR5	CIRPGNNTRKSIRIGPGQAFYATGDIIGQIRQAHC	35
KX983851	C	CCR5	CIRPNTNTRKSVRIGPGQAFYATGDITGDIRKASC	35
KF716466	C	CCR5	CTRPNNNTRKNVRIGPGQAFYATNGIIGDIRQAYC	35
KR820314	C	CCR5	CTRPNNNTRKSVRIGPGQTFYATGGIIGNIRQASC	35
KR820324	C	CCR5	CTRPGNNTRKSIRIGPGQTFYATGEIIGDTRQAHC	35
KR820326	C	CCR5	CVRPSNNTRTSIRIGPGQTFYAKGEIIGDIRQAHC	35
KR820342	C	CCR5	CTRPNNNTRKGIGIGPGQTFYATGAIIGDIRQAHC	35
KR820376	C	CCR5	CIRPGNNTRTSIRIGPGQAFYTTGDIIGDIRKAHC	35
KR820415	C	CCR5	CIRPNNNTRKSMRIGPGQTFYATGEIIGNIRQALC	35
KP109494	C	CCR5	CTRPSNNTRKSIRIGPGQTFYATGDITGDIRQAHC	35
KP109495	C	CCR5	CIRPNNNTRKSIRIGPGQTFYATGGIIGDIRKAYC	35
KF716467	C	CCR5	CVRPNNNTRKSLRIGPGQTFYATGDIIGDIRQAHC	35
KP109496	C	CCR5	CTRPDNNTRKSVRIGPGQTFYATGDIIGDIRQAHC	35
AB485647	C	CCR5	CTRPSNNTRKSIRIGPGQAFYATGDIIGNIRQAHC	35
AY805330	C	CCR5	CTRPNNNTRKSMRIGPGQAFYATGEIIGNIREAHC	35
KU200938	C	CCR5	CSRPNNNTRKSVRIGPGQTFYAMGDIIGDIRAAHC	35
JF680908	C	CCR5	CTRPNNNTRRSIRIGPGQTFFAANGIIGEIRQAHC	35
JF680918	C	CCR5	CVRPNNNTRKSVRIGPGQAFYATGDIIGNIRQAHC	35
KC894102	C	CCR5	CTRPGNNTRKSVRIGPGQTFYATGDIIGDIRKAHC	35
KX907388	C	CCR5	CTXPSNNTRKSIRIGPGQMFFATNEIIGDIRQAHC	35
GU080176	C	CCR5	CIRPGNNTRKSVRIGPGQTFYATGEXIGDIRQAHC	35
K03455	B	CXCR4	CTRPNNNTRKRIRIQRGPGRAFVTIGKIGNMRQAHC	36
DQ383746	B	CXCR4	CTRHGNNTRRSIHMGPGRAFYTTGEVTGDIRKAHC	35
KY968399	B	CXCR4	CTRLNNNTRKRITMGPGRVYYTTGGIIGDIRKAHC	35
AY037268	B	CXCR4	CTRPGNRTRRRITLGPGRVYYTTGEIMGDIRKAHC	35
AF042100	B	CXCR4	CTRPSNSTGQSIRIGQRRAFYATGKIIGDIRHAHC	35
AY624305	B	CXCR4	CTRPNNNTRRGIYIGPGRAIYTTDKIVGDIRKAYC	35
GU367406	B	CXCR4	CTRPNDNRRKSIQVGPGRALYATGQIIGDIRQAHC	35
FJ653258	B	CXCR4	CTRPGNNTRKSIYIGPGRAVHVTDRIIGDIRQAHC	35
MN486010	B	CXCR4	CIRHNNNTRRSIHIGPGRAYYTPGQIIGDIRKAYC	35
L07421	B	CXCR4	CTRPNNSKRKTLHMGPKRAFYATGDIGGYTGYIRQAH	38
MH746261	B	CXCR4	CTRPNNNTRKGIRIGPGRAFLAAEKIIGDIRKAHC	35
JN692451	B	CXCR4	CTRPNNNTRKSIQLVGGRTLYATGKIIGEIRQAHC	35
KU749389	B	CXCR4	CTRPNNNIRKGIHLGPGSVFHVHQKVIGDIRKAYC	35
KT427671	B	CXCR4	CTRLGNNTRRSIHMGPGRAFYTTGEIIGDIRKAYC	35
KT427716	B	CXCR4	YTRPNNNTRKSIHLGARRALYTTDIVGDIRKAYC	34
KT427853	B	CXCR4	CTRPSNNTRRSIHLGPGRVIYATGQITGDIRQAHC	35
KT427781	B	CXCR4	CTRLGNNTRKSIHMGFGRTLYATGEIIGDIRKAHC	35
KT427767	B	CXCR4	CTRPNNNTRKSIRIQIAPGRAFYATGKIGDIRRAHC	36
KX181912	B	CXCR4	CTRPNNNTRRSIHFGPGRTLYATGEIIGDIRRAHC	35
KX181900	B	CXCR4	CSRPNNNTRKSIHMGWGRAFYARGDIIGDIRRAHC	35
KX181910	B	CXCR4	CVRPGNNTRKSIHLGPGRALYATGSIIRDIRKAYC	35
AY173956	B	CXCR4	CTRPNNKARRRIRIGPGRTFYTGKIVGDIRQAYC	34
KX792664	B	CXCR4	CIRPNNNTRKSYPVGTGRVLYTRGIIGDIRQAHC	34
AY180905	B	CXCR4	CTRPSNNTRKRVTLGPGRVWYTTGQIIGDIRRAHC	35
EU131797	B	CXCR4	CTRPNNNTRKSIHLGQGRAWYTTGQIIGDIRQAHC	35
EU131791	B	CXCR4	CTRPNNNTRKSIHLGPGHAWHTTRRIVGNIRQAHC	35
EU131808	B	CXCR4	CTRPSNNTRKSIQLGQGRAWYTTGQIIGDIRRAHC	35
DQ990880	B	CXCR4	CTRPNNNTRKRITMGPGRVLYTTGQIVGDIRKAHC	35
HM215420	B	CXCR4	CTRPNNNTRKRVTLGPGRVWYTTGQIIGDIRKAHC	35
HQ699995	B	CXCR4	CTRPNNNTRKRVTLGPGRVWYSTGQIIGDIRRAYC	35
HM215428	B	CXCR4	CTRPSNNTRKSIHLGQGRAWYTTGKIIGDIRQAHC	35
EU131807	B	CXCR4	CIRPNNNTRKSINLGQGRAWYATGQIIGNIRQAHC	35
MH479968	B	CXCR4	CTRPNNNTRKRITLGPGRVLYATGQIIGDIRRAHC	35
JF932475	B	CXCR4	CTRPNNNTRKSIQLGLGRAWYTTGQIIGNIRKAYC	35
JF932476	B	CXCR4	CTRPNNNTRKRVSLGPGRVWYTTGQIVGDIRQAHC	35
JF932499	B	CXCR4	CTRPNNNTRKSIQLGLGRAWYTTGQIIGDIRQAHC	35
HQ215556	B	CXCR4	CTRPNNKTRKGISLGPGRWRSLYTTRDIIGDKRKAYC	37
KX692916	B	CXCR4	CTRPNNNTRKRVTLGPGRVWYTTGEIVGDIRQAHC	35
KX693091	B	CXCR4	CTRPNNNTRKRVTLGPGRVWYTTGQIIGDIRRAHC	35

KX693093	B	CXCR4	CTRPNNNTRKSVTLGPGRVWYTTGQIVGDIRRAYC	35
KX693539	B	CXCR4	CTRPNNNTRKSIHLGLGRAWYTTGQIIGNIRQAHC	35
KP109512	B	CXCR4	CTRPNNNTRRGIHIGPGRAIYATGAIIGKIRQAHC	35
GU647196	B	CXCR4	CTRPNNNTRKRVSIGPGRAWYTTKQIVGDIRQAHC	35
AY561239	B	CXCR4	CIRPNNNTRRSVHLGPGRGAIYTGDIIGKIRQAYC	35
FJ388895	B	CXCR4	CVRPNNNTRKGIHVGPGRAIYTTGKIIGDIRQAHC	35
JF683804	B	CXCR4	CTRPNRNIRKRITMGPGRVYYTTGGIVGDIRKAYC	35
MK086129	B	CXCR4	CIRPNNYTRKRIYIGPGRAFHTTGQIIGDIRQAHC	35
U43096	B	CXCR4	CTRPNNYTSKRIRIGARRAFYTKGKIIGDIRQAHC	35
U43141	B	CXCR4	CTRPNNNTRKGIHIGPGRAVYTTGRIVGDIRLAHC	35
EF514712	B	CXCR4	CIRPYNKRRERIRIGIGRTFQTTRTVDGDIRKAYC	35
FJ694790	B	CXCR4	CTRPNNNTRKGFRIGPGRAAFAHGKIIGDIRKAYC	35
GQ372988	B	CXCR4	CTRPNNNTRKSIHVGWGRALYTTGQIIGDIRKAHC	35
GU362885	B	CXCR4	CTRPYSYTTIRRIRIGPGRAFTTGSVGDIRQAHC	34
KC595172	B	CXCR4	CTRPNNNTRKGIYIGPGRAVYTTDKIIGDIRKAHC	35
DQ854716	B	CXCR4	CTRLNNNTRRSIHVGHVGPGRAIYTTGIIGKIRQAHC	37
KC595222	B	CXCR4	CTRPNNNTRKGIFIGPGRAFYATRIVGDIRQAHC	34
DQ141345	B	CXCR4	CTRPNNNTRKSIHIGLGRAFYATGQIIGDIRQAHC	35
KC699013	B	CXCR4	CTRPNNNTRRRITLGPGRVFYTTGDIIGDIRRAHC	35
KF695111	B	CXCR4	CIRPNNNTRKSMTLGPGKVFYTTGVTGDIRKAHC	34
EF033658	B	CXCR4	CTRPHMSKSRSIRLGPGRPFYATGNIIGALKQAHC	35
M26727	B	CXCR4	CTRPNNNTRNRISIGPGRAFHTTKQIIGDIRQAHC	35
JN034139	B	CXCR4	CTRPNNNTRRSIHLGPGKTLYATDIIGDIRRAHC	34
HQ595790	B	CXCR4	CTRPNNNTRKRITMGPGRVLYATGEIIGDIRRAHC	35
D10112	B	CXCR4	CTRLNNNTRKSIAIGPGRTVYATDRIIGDIRQAHC	35
Y13716	B	CXCR4	CTRPNNNTRKGIYMGPGRRFYTTGRIIGDIRQAHC	35
AJ286342	B	CXCR4	CTRPGNNTRKRIAIGPGRAVYATRQIIGDIRQAHC	35
U36875	B	CXCR4	CTRPNNKTKRSFHIGPGRAFLTEIVGDIRQAYC	33
U23487	B	CXCR4	CTRPSNNSRKSIYIGPGRRFHVTRAVTGDIRQAHC	35
AJ286332	B	CXCR4	CTRPNNNTRKRISIGPGRSFVTTGQIIGDIRQAHC	35
AJ286334	B	CXCR4	CTRPNNHTRKSIHLGPGRWSVLATKDIIGDIRKAHC	36
AJ286336	B	CXCR4	CTRPNNNTRKGIRIGPGRAFLVTDRIIGDIRQAHC	35
U36882	B	CXCR4	CTRPGNNTRRSITIGPGRAFHATGRIIGDIRRAHC	35
AB289588	B	CXCR4	CTRPNNNTRKSIRIHRGPGRAFVTSGIPGDIRQAHC	36
AB480695	B	CXCR4	CTRPSNNTRKRIHIGPGRAVYTTDQITGDIRKASC	35
AB287363	B	CXCR4	CTRPGYNTIKRVSIGPGRGRAIVATKKIEGDTKKAYC	37
AB287364	B	CXCR4	CTRPNINRRKGLRIGPGRAFFTTGNIKGDIKQAYC	35
AB588296	B	CXCR4	CTRPNNNTRKSIHLGFGRAMYATGQIIGDIRQAHC	35
D12582	B	CXCR4	CIRPNNKTRKRVTMGPGRVYYTTGEIIGDIRQAHC	35
JQ316127	B	CXCR4	CTRPGNNTRRSIRIGPGRAFYTTGDITGDIRRAYC	35
JQ316126	B	CXCR4	CTRPNNNIERRISIGPGRAFRAIKAITGDIRKAHC	35
DQ295196	B	CXCR4	CTRPNNNTRRGIHIGLGRTFYATGEIVGNIRQAHC	35
HQ386218	B	CXCR4	CTRPNNNTRKRVTLGPGRVWYTTGEIIGDIRKAHC	35
U34603	B	CXCR4	CTRPNNNTRKGIHIGPGRAFYAARKIIGDIRQAHC	35
GU455465	B	CXCR4	CTKPNNNQRRRIRLGPGPGSVFYATGEIIGHIRQAQC	37
U15030	B	CXCR4	CTRPNNNIRKRIHIGPGRAFYTTGQIIGNIRQAHC	35
L08655	B	CXCR4	CTRPNNNTRKGIHIGPGRAVYTTGRIIGDIRQAHC	35
KY213751	B	CXCR4	CTRPNNNTRKSIHLGLGRRWYATEIIGDIRQAHC	34
JN248329	B	CXCR4	CTRPNNYTRKRISIGPGRAWITTGQIIGDIRQAHC	35
EU578386	B	CXCR4	CTRPNNNTIKSIRMGIRRAFYTKEIIGDIRQAHC	34
AF086817	B	CXCR4	CTRPNNISKRRSMHIGTGRVFYTANXIGNIRQAHC	35
DQ823363	B	CXCR4	CTRPNRNTIRKIYIGRGRAIHATDLTGDIRKAHC	34
DQ869027	B	CXCR4	CTRPNEYRTRRIHIGPGRAFVTTKSITGDIRQAYC	35
EU578061	B	CXCR4	CTRPNNNTRKGIHIGPGRAVYATGKVIGDIRQAHC	35
FJ469737	B	CXCR4	CTRPNNTTRKGIRIGPGRAVYATEKIIGDIRKAHC	35
FJ469748	B	CXCR4	CTRPSNKLIRRIHIGPGRAFYGTDIKGNIRQAYC	34
DQ410422	B	CXCR4	CTRPGNKTRKSITRGPGRVLYTTGGIIGNIRLAHC	35
FJ469739	B	CXCR4	CTRPNIKKARHIHIGPGRAFYATGSITDIRRAQC	34
FJ469753	B	CXCR4	CTRPYHNAKQRIHIGPGRAFYATKRIKGDIRQAHC	35
EF593256	B	CXCR4	CTRPNNNTRKRMTFGPGKVFYTTGEIVGDIRRAHC	35
FJ469684	B	CXCR4	CTRPNNNTRKRITMGPGRVYYTTGEVIGNIRQAHC	35
FJ469698	B	CXCR4	CTRPIIKIRKGIHMGPGKAFYATGDILGDIRRAHC	35
DQ410498	B	CXCR4	CTRPSNNTKKGIYIGPGRTVHVARKIIGDIRQAHC	35
JQ251132	B	CXCR4	CTRPNNNTRKSINLGQGRAWYTLEKVIGDIRHAYC	35
JQ403104	B	CXCR4	CIRPNNNTRKRITMGPGRVLYTTGEIIGDIRKAHC	35
MK383403	B	CXCR4	CTRPGNNTRRRIRIGLGRAFYATEEITGDTRKAYC	35
HQ216762	B	CXCR4	CTRPNNNTRKRVTLGPGRVFYTTGQVIGDIRKAHC	35
HQ216816	B	CXCR4	CTRPNNNTRKMIHMGHIGGGRAFYATGKIIGDIRQAH	38
HQ217546	B	CXCR4	CTRPNNNTRRGIYIGPGRAVYTTGRIIGDIRKAHC	35
HQ217889	B	CXCR4	CTRPGNNTKKGISIGPGRTVYATERIVGDIRKAQC	35

JN944909	B	CXCR4	CMRPGNNTSKSIHMGALRAFHATSRIIGDTRRAHC	35
JF689879	B	CXCR4	CTRPNNNTRKRITMGPGRVIYTTGEIIGNIRQAHC	35
MK383385	B	CXCR4	CTRPNNNTRKSIRIGPGRTIYATGRIIGDIRKAHC	35
JF320530	B	CXCR4	CTRPNNNTRKSIHIGLGRAFYATGQIIGDIRKAHC	35
JF320631	B	CXCR4	CTRPDNNTRRGIHIGPGRGLFYTGEIIGDIRRAHC	35
JF320577	B	CXCR4	CTRPNNNTRKRISLSPGRVFYTTGEIIGDIRRAHC	35
FJ469716	B	CXCR4	CTRPNNNTRKGIYIGPGRAVYATGRIIGDIRQAHC	35
JQ403096	B	CXCR4	CTRPNNNTRRSIHIGLGRRFYTRQIIGNIKQAHC	34
KR423340	B	CXCR4	CTRPYNGGKIRRIHIGPGKTFYTTNNTYIRQAYC	34
HM204596	B	CXCR4	CSRPNNNTRKRISLGPGRVLYTTGEIIGDIRQAHC	35
KM081913	B	CXCR4	CTRPDNNIKKRILHIGPGRAFYTKVMGDIRKAYC	34
MK383384	B	CXCR4	CTRPHNNIRGRMSIGPGRAFVATREVTGVIRQAHC	35
KF526312	B	CXCR4	CTRPNNNTRRGIHIGLGRAVYVTRKIIGDIRQAHC	35
KF526323	B	CXCR4	CTRTNNYTSKGLSIGPGRAFVAARQIIGDIRQAHC	35
MG197146	B	CXCR4	CTRPNNNTRRGIHMGPGRTFYATDIIGDIRRAHC	34
KF384799	B	CXCR4	CTRHNNNKKIQRIHIGPGRAFVATKGITGDIRQAHC	36
KF384806	B	CXCR4	CTRPGKKLSRIIHIGPGRAFYASDGRDIRQAYC	33
KU901848	B	CXCR4	CTRPYIKTSRRVHIGPGRAWSAMDVTGDIRKAYC	34
MH897917	B	CXCR4	CTRPNNNTRRRSVRLGPGNAYFRTREIIGDIKKAHC	36
MG196922	B	CXCR4	CTRPNNNTRKGIHLGFGRTFYRVGDIIGDIRKAHC	35
MG196979	B	CXCR4	CTRPNNNTRKGIHIGPGRAFYATGQIVGDIRRAHC	35
KY766175	B	CXCR4	CTRPNNNTKQGIYIGPGRRFYTTKRIIGDIRKAFC	35
KX028508	B	CXCR4	CTRPNNNTRKGIHMGPGRALFTERIIGDIRKAHC	34
KX505739	B	CXCR4	CTRPNNNTRKRVTMGPGKVWYTTGEIIGDIRKAHC	35
KX587284	B	CXCR4	CTRPGNNLRRRVHIGPGRAFFTSDIRGDIRRAHC	34
MK114705	B	CXCR4	CTRPNNNTRKRISMGPGRVIYATGQIIGDIRKAHC	35
M17451	B	CXCR4	CTRPNNNTRKSITKGPGRVIYATGQIIGDIRKAHC	35
AY835779	B	CXCR4	CTRPNNKKVRRIHIGPGRAFYTTGQIVGNIRQAHC	35
M38431	B	CXCR4	CTRPNNNTRKGIAIGPGRTLYAREKIIGDIRQAHC	35
AY352275	B	CXCR4	CTRPNNNRRRRITSGPGKVLYTTGEIIGDIRKAYC	35
M38430	B	CXCR4	CTRPNIYRKGRIHIGPGRAFHTTRQIIENIRQAHC	35
AY835778	B	CXCR4	CTRPNNNTRKSIRIGLGRRFYATKIIGDIRKAHC	34
L02317	B	CXCR4	CTRPNKKTRKRITTGPGRVYYTTGEIVGDIRQAHC	35
AF286365	B	CXCR4	CTRPGNKIRRRIHIGPGRAFYTDRVGDIRQAYC	33
U39362	B	CXCR4	CTRPNNNTRRRLSIGPGRAFYARRNIIGDIRQAHC	35
U21135	B	CXCR4	CTRPNNNTRKKITLGPGRVLYTTGEIIGDIRRAHC	35
MH012866	B	CXCR4	CIRPGNNTRKSIHIGLGRALYTTEIIGNIRLAYC	34
AY608577	B	CXCR4	CTRPGNNTRKRVTLGPGRVYYTTGQIIGDIRKAHC	35
MH013075	B	CXCR4	CTRPNSKIRKIIHLGLRRAFYATKRIGNIKQAEC	34
AY835768	B	CXCR4	CTRPHKKIRKRVHMGGGRTFYATGIDGDIRKAYC	34
JN562786	B	CXCR4	CTRPNKYTRKDIHIHPGRAFFATGHIIGDTRRAYC	35
EF593200	B	CXCR4	CTRPNNNTRKGIHLGPGKVLYATDIIGDIRRAYC	34
AY331294	B	CXCR4	CIRPNNNTRKRITMGPGKVYYTTGQIIGDIRQAHC	35
AY560108	B	CXCR4	CTRPYHNIRRWIHIGPGRAFYAAKGIQGTMRQAHC	35
JN786786	B	CXCR4	CTRPNNYTSKNIRFGPGGAFRARGRIIGDIRKAYC	35
JN599165	B	CXCR4	CLRPNKYTRQRIHIGPGRAFYTARDIIGDIRKAYC	35
KT124798	B	CXCR4	CTRPNNNTRRSINIGPGRAFYLRDIIGDIRQAHC	34
FJ798416	B	CXCR4	CTRPNNNTRQRISIGPGRAFYTTRQVIGDIRQAHC	35
MH262735	B	CXCR4	CTRPNNNTRKGIRVGPGRTIYAARRIIGDIRKAHC	35
KX984877	B	CXCR4	CTRPNNNTRSRISLGPGRAFITTKQIIGDIRQAHC	35
MH632949	B	CXCR4	CTRLNNNTRRGIHTAFGRTLFVTNVIGDIRKAFC	34
MK114705	B	CXCR4	CTRPNNNTRKRISMGPGRVIYATGQIIGDIRKAHC	35
DQ127534	B	CXCR4	CTRPSTNKKKKISMGPGRVYYTTGQITGDIRRAYC	35
AF219627	B	CXCR4	CTRPNNNIRKGLRIGPGRAWWYATRGIIGKMRQTHC	36
FJ469692	B	CXCR4	CTRPNRYTKQRIHIGPGRAFYATKSIAGNIRQAHC	35
FJ469711	B	CXCR4	CTRPNNYTRKRVSIGPGRAFRTTGQIIGNIRQAHC	35
MN515956	B	CXCR4	CTRPNNNTRRRVTMGPGRVYYTTGQIIGDIRKAHC	35
FJ469729	B	CXCR4	CTRPNNNTSRRISIGPGRAFYAKERIIGDVRRAYC	35
KP754466	B	CXCR4	CTRPNNNTRRSIHLGFGRALYTTGEITGDIRKAHC	35
AF049495	B	CXCR4	CTRPNYNETKRIRIHRGYGRSFVTVRKLGDRKQAHC	36
FJ469756	B	CXCR4	CARPNNNTRKSIRIGHRSALYATGGIIGDIRQAHC	35
FJ469761	B	CXCR4	CTRPNNNTRRRMYIGQGRAVYTTGQIIGDIRKAHC	35
GU728229	B	CXCR4	CTRPNNNTRKSITMGPGRVVYATGQIIGDIRRAYC	35
AY189526	B	CXCR4	CTRVSKNIRQKRIKIGPGRAFVATGDIGDIRKAHC	35
KJ948660	B	CXCR4	CTRPGNNTRKGIRIGPGRALYTTERIIGDIRQAHC	35
KF726018	B	CXCR4	CTRPNNNTRKRITMGPGRVFYTTGQIIGDIRQAHC	35
MH234643	B	CXCR4	CTRPNNKRTRRIHIGPGRAFYTGDIIGDIRQAHC	34
KU168258	B	CXCR4	CTRPNNTTRKGIAIGPGRKFYTAEKIIGDIRQAHC	35
KU168259	B	CXCR4	CTRPNNNTRQRISIGPGRAFVAGRAIIGNIRQAHC	35
KT452194	B	CXCR4	CTRPGNNTRRGIHIGPGRAMYTNNIVGDIRRAHC	34

MK041562	B	CXCR4	CTRPGNNTIKXISIGPGRAFRTRGQIIGDIRQAHC	35
AF443094	C	CXCR4	CTRPNNNTRRSVRIGPGQTFYARGDIIGNIRQAHC	35
AF443115	C	CXCR4	CRRPNNNTGKSVRIGPGQTFFATGGIIGEIRRAHC	35
KR861336	C	CXCR4	CVRPHNNTRQSIRIGPGQTFYATGRITGDIRRAHC	35
JF683768	C	CXCR4	CIRPGNNTRNRIRIGPGRTFLAHKHIIGDIRQAHC	35
KU319537	C	CXCR4	CTRPNNNTRKRVGIGPGLAFRAHGNIIGDIRQAHC	35
KP109482	C	CXCR4	CTRPNENRRKSIRIGPGQAFYATGDIIGDIRQARC	35
EU521729	C	CXCR4	CIRPNNYTRKGIRIGPGRTVYAARKIIGDIRKVHC	35
MG899863	C	CXCR4	CTRPGNNTRKSVRIGPGRAYLIDRIVGDIRRAYC	34
MF373136	C	CXCR4	CTRPNNNTRRRVLIGPGQAFYATRDILGNIRQAHC	35
MF373187	C	CXCR4	CTRPNNNTRKRIRLGPGRTIYATGAIIGNIRQAQC	35
MF373202	C	CXCR4	CTRPGTNRRRSIRIGPGQAIYTTNEVTGDIRQAHC	35
AY253310	C	CXCR4	CIRVGNNTRKSVRIGPGQVFFANNVIGDIRQAHC	34
KX907356	C	CXCR4	CIRTGNNTRRSIRIGPGQTFYATGDIIGDIRKAYC	35
AY463221	C	CXCR4	CTRPGNNTRRSIRIGPGQAFYTNKIIGDIRKAHC	34
FJ846632	C	CXCR4	CTRPDNKISMKRIKIGPGRAFVATKGIKGDIRQAYC	36
AY529672	C	CXCR4	CTRPNNYTRKRLRIGPGYAFYAKNDIIGDIRKAYC	35
DQ275643	C	CXCR4	CTRPGNNTRRSIRIGPGQTFYTNHIIGDIRKAYC	34
AY878064	C	CXCR4	CTRPNHNKRIRVRIGPGRTFVTRNIVGDIRQAYC	34
DQ056414	C	CXCR4	CIRAGNKTRKSVRIGPGQVFYATGEVTEDIRQAHC	35
DQ382372	C	CXCR4	CTRPANTRIKRLGIGPGQAFRTVKQIIGDIRQSHC	35
DQ382378	C	CXCR4	CTRPGKGKRTRVRIGPGRTFYATGAVTGDIRKAHC	35
DQ382362	C	CXCR4	CSRPGNNTRKSVRIGIGRGQTFYATGKVIGDIRQAHC	37
GQ999985	C	CXCR4	CTRLGNNTRKSIRIGPGQAFYTTNAILGNIRQAYC	35
HQ615955	C	CXCR4	CTRPGNNTRKSIRIGPGQMLYATKIIGDIRQAYC	34
HQ595742	C	CXCR4	CTRLGNNTRRSVRIGPGQTFYATGEIIGDIRKAYC	35
HM623554	C	CXCR4	CTRPGNKTRRSMRIGPGRVFYTNSILGDIRKAYC	34
HM623556	C	CXCR4	CTRPYKIVRRRGIGIGPGQTVQANAQVIGDIRQAHC	36
HM623559	C	CXCR4	CTRPGNITKRSMRIGPGRVFYANNIMRDIRQAYC	34
HM623566	C	CXCR4	CIRPGNNTRKSIRIGPGKTFYATNKIIGDIRKAHC	35
HM623575	C	CXCR4	CTRPGNNTRKSVRIGIGRGHTFYATGAIGKDARQAHC	37
HM623580	C	CXCR4	CTRPGNITRRSFRIGPGRAFITTKRISDIRQAYC	34
GU080168	C	CXCR4	CTRPNINKRQRIRIGPGRAFVAIGKIIGDIRKAHC	35
GU080171	C	CXCR4	CTRPGDHRKRIIRIGPGQAFHARDNIIGDIRKAYC	35
GU080182	C	CXCR4	CTRPGNNTRRSIRVGPGQSIYATNRIIGDIRKAYC	35
GU080184	C	CXCR4	CTRPNNNRRRSMRIGPGQVFYATDAIIGNIRQAYC	35
GU080186	C	CXCR4	CTRPGNNTRRGIRIGPGQTFFATRTIGNIRQAHC	34
GU080190	C	CXCR4	CIRPNSHTRQEGVRIGPGRAFYVRGKIIGDIRKAYC	36
GU080198	C	CXCR4	CTRPGNNIRKRIGIGPGQAFRATSGIIGNIRQAQC	35
HM623591	C	CXCR4	CTRPGNKTRKRIGIGPGQAFHTHTVIGDIRKAYC	34
HM623601	C	CXCR4	CIRTGNNTRKSIRIGPGQVFYATGDVIRNIRVAHC	35
KF725884	C	CXCR4	CTRPGNNTRKSVRIGPGQTFYAKIIGDIRQAHC	33
KF725885	C	CXCR4	CTRPNNNTRKSVRIGPGQTFYATGDIIGRIRQAHC	35
KF114888	C	CXCR4	CTRPNNNTRKSMRIGPGQTFYATGAIIGKIRQAHC	35
KF725959	C	CXCR4	CIRTGNNTRKSIRIGPGQVFFAATSIIGDIRKAHC	35
MH933704	C	CXCR4	CTRIANNTRRSIRIGPGQSFYTHDIIGDIRKAHC	34
MN097654	C	CXCR4	CIRPGNNTRRSIRIGPGQTFYATGDIVGDIRRAYC	35
AF411967	C	CXCR4	CARPGNNTIKRIRIGPRYAFYAKETIIGDIRQAHC	35
AY529673	C	CXCR4	CTRPNNNTRKSVRIGIGRGHAFYTTGKVIGNIRQAHC	37
AY529677	C	CXCR4	CTRPNNNTRKNVRIGIGRGQTFYANGRIIGNIRQAHC	37
AY529678	C	CXCR4	CARPGNNTRKMMRIGIGRGQTFYANGQVIGDIRQAHC	37
AF411966	C	CXCR4	CTRPGSNKQRIRNIRIGPGRAFHTNGVIGDIRKAYC	36
HQ625586	C	CXCR4	CTRLNNNTRKSIRIGPGQTFYATGGIIGDIRKAHC	35
GU329394	C	CXCR4	CMRPGNNTKRRRVRIGHGPVFYTHDIIGDIRQAHC	35
AY494971	C	CXCR4	CTRPGNNTRKAIGIGPGQTLFATNRIVGDIRRAHC	35
KF725955	C	CXCR4	CIRPNNNTRKSIRIGPGQAYXTYNKIIGNIRQAHC	35
