# Supplementary material for: Covariation of Amino Acid Substitutions in the HIV-1 Envelope Glycoprotein gp120 and the Antisense Protein ASP Associated with Coreceptor Usage
Source: Viruses. 2025 Feb 26;17(3):323. doi: 10.3390/v17030323 (PMC11946160; doi:10.3390/v17030323)
Supplement: Supplementary file 1 [file viruses-17-00323-s001.zip › Supplementary Table S1.pdf]

Supplementary Table S1.

A. Percent frequency within genotypes B (1557 strains) and C (939 strains) of the 42 amino acid substitutions in the V3 region of ENV significantly associated to coreceptor tropism (see Figure 2 in the main text).

Amino acid substitution in V3	Percent frequency of substitution in genotype B	Percent frequency of substitution in genotype C	Significance of % difference (P value)
K10R	17.5	10.8	<0.00001
H13R	3.7	95.0	<0.00001
R13H	56.0	0.0	<0.00001
R/H13S	6.7	0.1	<0.00001
R/H13P	12.8	0.2	<0.00001
R/H13T	7.1	0.2	<0.00001
R/H13N	10.0	0.1	<0.00001
I14L	12.9	0.4	<0.00001
R18Q	3.6	96.2	<0.00001
A19T	9.0	65.5	<0.00001
A19V	6.2	5.3	0.37
F20L	7.4	1.2	<0.00001
F20W	10.6	0.1	<0.00001
Y21F	9.1	12.2	0.012
A22T	32.2	6.1	<0.00001
G24D	2.1	5.4	0.00001
D/E25Q	12.7	1.0	<0.00001
D25E	28.7	21.0	0.00003
I26V	6.9	5.2	0.096
Q32K	14.3	10.1	0.003
H34Y	13.6	22.8	<0.00001

B. Percent frequency within genotypes B (1557 strains) and C (939 strains) of the 5 amino acid substitutions in the antisense protein ASP significantly associated to coreceptor tropism (see Figure 3 in the main text).

Amino acid substitution in ASP	Percent frequency of substitution in genotype B	Percent frequency of substitution in genotype C	Statistical significance
L20F	6.4	0.2	<0.00001
K106N	2.1	95.2	<0.00001
L119R	0.5	90.6	<0.00001
H157Q	8.6	3.7	<0.00001
I161M	11.3	70.7	<0.00001

C. Percent frequency within genotypes B (1557 strains) and C (939 strains) of the 13 amino acid substitutions in the V1/V2 region of ENV significantly associated to coreceptor tropism (see Figure 4 in the text).

Amino acid substitution in V1/V2	Percent frequency of substitution in genotype B	Percent frequency of substitution in genotype C	Statistical significance
E38M	11.3	30.6	<0.00001
E52S	38.9	0.0	<0.00001
D55T	5.5	0.0	<0.00001
D55N	14.6	1.9	<0.00001
I/69>L	8.7	5.5	0.004
I/L72M	4.3	0.8	<0.00001
N74E	7.1	29.7	<0.00001
N74K	7.0	9.7	0.016
S83R	5.2	0.8	<0.00001
S83E	1.9	56.0	<0.00001
N/S88H	6.7	3.0	0.00005
