# Supplementary material for: Covariation of Amino Acid Substitutions in the HIV-1 Envelope Glycoprotein gp120 and the Antisense Protein ASP Associated with Coreceptor Usage
Source: Viruses. 2025 Feb 26;17(3):323. doi: 10.3390/v17030323 (PMC11946160; doi:10.3390/v17030323)
Supplement: Supplementary file 1 [file viruses-17-00323-s001.zip › Supplementary Table S2.pdf]

Supplementary Table S2.

A. Percent conservation of the most frequent amino acid in the 20 positions of the V3 loop of gp120 showing a significant difference between R5-tropic and X4-tropic strains (see Figure 2 in the main text).

Amino acid position in V3	Amino acid	Percent conservation in V3 sequences with a predicted tropism R5	Percent conservation in V3 sequences with a predicted tropism X4	Percent conservation in all V3 sequences
7	N	99.7	78.3	97.7
8	T	98.8	79.6	97.0
9	R	96.5	82.6	95.2
10	K	78.5	61.8	76.9
11	S	85.2	37.0	80.6
13	R+H	74.7	57.4	73.0
14	I	79.7	57.9	77.6
16	P	96.7	80.4	95.1
18	R	42.1	75.7	45.3
19	A	60.8	52.3	60.0
20	F	83.6	54.9	80.8
21	Y	87.3	72.3	85.9
22	A	77.6	46.5	74.7
23	T	89.9	71.0	88.2
24	G	84.6	57.9	82.1
25	D+E	74.3	27.6	70.3
26	I	93.9	85.4	93.1
27	I	94.0	71.2	91.9
32	Q	80.2	50.6	77.4
34	H	82.2	71.5	81.2

B. Percent conservation of the most frequent amino acid in the 5 position of the antisense protein ASP showing a significant difference between R5-tropic and X4-tropic strains (see Figure 3 in the main text).

Amino acid position in ASP	Amino acid	Percent conservation in ASP sequences from strains with a predicted tropism R5	Percent conservation in ASP sequences from strains with a predicted tropism X4	Percent conservation in ASP sequences from all strains
20	L	96.0	90.2	95.5
106	K	60.6	77.4	62.2
119	L	62.2	76.6	63.6
157	H	93.3	88.5	92.9
161	I	58.7	68.1	59.6

C. Percent conservation of the most frequent amino acid in the 10 position of the V1/V2 region of ENV showing a significant difference between R5-tropic and X4-tropic strains (see Figure 4 in the main text).

Amino acid position in V1/V2	Amino acid	Percent conservation in V1/V2 sequences with a predicted tropism R5	Percent conservation in V1/V2 sequences with a predicted tropism X4	Percent conservation in all V1/V2 sequences
38	E	30.0	43.7	31.4
52	E	37.8	21.9	36.3
55	D	80.9	68.1	79.7
69	I+V	91.8	88.1	91.5
70	V	81.1	71.5	80.2
72	I+V	94.3	88.9	92.9
74	N	38.0	40.9	38.3
83	S	45.7	54.0	46.4
84	Y	98.1	88.5	97.2
88	A+S	93.2	74.3	91.4
